# RNA-Seq in *Mytilus galloprovincialis*: comparative transcriptomics and expression profiles among different tissues

**DOI:** 10.1186/s12864-015-1817-5

**Published:** 2015-09-24

**Authors:** Rebeca Moreira, Patricia Pereiro, Carlos Canchaya, David Posada, Antonio Figueras, Beatriz Novoa

**Affiliations:** Instituto de Investigaciones Marinas (IIM), Consejo Superior de Investigaciones Científicas (CSIC), Eduardo Cabello, 6, 36208 Vigo, Spain; Departamento de Bioquímica, Genética e Inmunología, Facultad de Biología, Unidad Asociada CSIC, Universidade de Vigo, 36310 Vigo, Spain

**Keywords:** *Mytilus galloprovincialis*, Transcriptome, NGS, RNA-Seq, NOISeq, KEGG, Gene Ontology, Blast2GO

## Abstract

**Background:**

The Mediterranean mussel (*Mytilus galloprovincialis*) is a cosmopolitan, cultured bivalve with worldwide commercial and ecological importance. However, there is a qualitative and quantitative lack of knowledge of the molecular mechanisms involved in the physiology and immune response of this mollusc. In order to start filling this gap, we have studied the transcriptome of mantle, muscle and gills from naïve Mediterranean mussels and hemocytes exposed to distinct stimuli.

**Results:**

A total of 393,316 million raw RNA-Seq reads were obtained and assembled into 151,320 non-redundant transcripts with an average length of 570 bp. Only 55 % of the transcripts were shared across all tissues. Hemocyte and gill transcriptomes shared 60 % of the transcripts while mantle and muscle transcriptomes were most similar, with 77 % shared transcripts. Stimulated hemocytes showed abundant defense and immune-related proteins, in particular, an extremely high amount of antimicrobial peptides. Gills expressed many transcripts assigned to both structure and recognition of non-self patterns, while in mantle many transcripts were related to reproduction and shell formation. Moreover, this tissue presented additional and interesting hematopoietic, antifungal and sensorial functions. Finally, muscle expressed many myofibril and calcium-related proteins and was found to be unexpectedly associated with defense functions. In addition, many metabolic routes related to cancer were represented.

**Conclusions:**

Our analyses indicate that whereas the transcriptomes of these four tissues have characteristic expression profiles in agreement with their biological structures and expected functions, tissue-specific transcriptomes reveal a complex and specialized functions.

**Electronic supplementary material:**

The online version of this article (doi:10.1186/s12864-015-1817-5) contains supplementary material, which is available to authorized users.

## Background

The Mediterranean mussel (*Mytilus galloprovincialis*) is a cultured bivalve species with an important commercial and ecological value worldwide [1, 2]. In contrast to other cultured bivalves such as clams and oysters, where different pathogens may result in massive mortalities and therefore, substantial economic losses [3–5], *M. galloprovincialis* displays an extraordinary resistance to a variety of pathogens [6]. Although molluscs lack a specific immune response, their innate response, which involves circulating hemocytes and a large variety of molecular effectors, constitutes an efficient defense mechanism [7–9]. While a wide range of molecules involved in the bivalve immune system have been described [10–13], particularly for mussels and oysters [14–18], the information is very limited compared, for example, to vertebrates.

Unfortunately, most bivalve genomic resources are not annotated or well described, with the exception of the Pacific oyster, *Crassostrea gigas*, whose genome has been recently published [19] or the pearl oyster, *Pinctada fucata,* in which genome annotation is still at the draft level [20]. Several bivalve transcriptomes are publicly available for *M. galloprovincialis* [21, 22], *Bathymodiolus azoricus* [23], *Patinopecten yessoensis* [24], *Ruditapes philippinarum* [25, 26] *Corbicula fluminea* [27] and *Crassotrea gigas* [19, 28, 29]. There are also 666 entries from the class Bivalvia deposited in the NCBI Short Read Archive (SRA) (25/03/2015). The number of available sequences for *M. galloprovincialis* is constantly increasing [30–33]. As an example, 23 *M. galloprovincialis* entries are publicly available in the SRA database, including whole-body, digestive gland and hemocytes transcriptomes, a *M. galloprovincialis* EST database called Mytibase [16].

In this study we show the results of the first comparative RNA-Seq analysis of gene expression in different *M. galloprovincialis* tissues, including gills, muscle, mantle and hemocytes. The raw data are accessible from the NCBI Short Read Archive (SRA: SRP033481). Additional files 1, 2, 3, 4 and 5 include all the transcripts obtained, together with their expression values, annotation and sequences in FASTA format.

## Results and discussion

### Sequence analysis and functional annotation

**Fig. 1:**
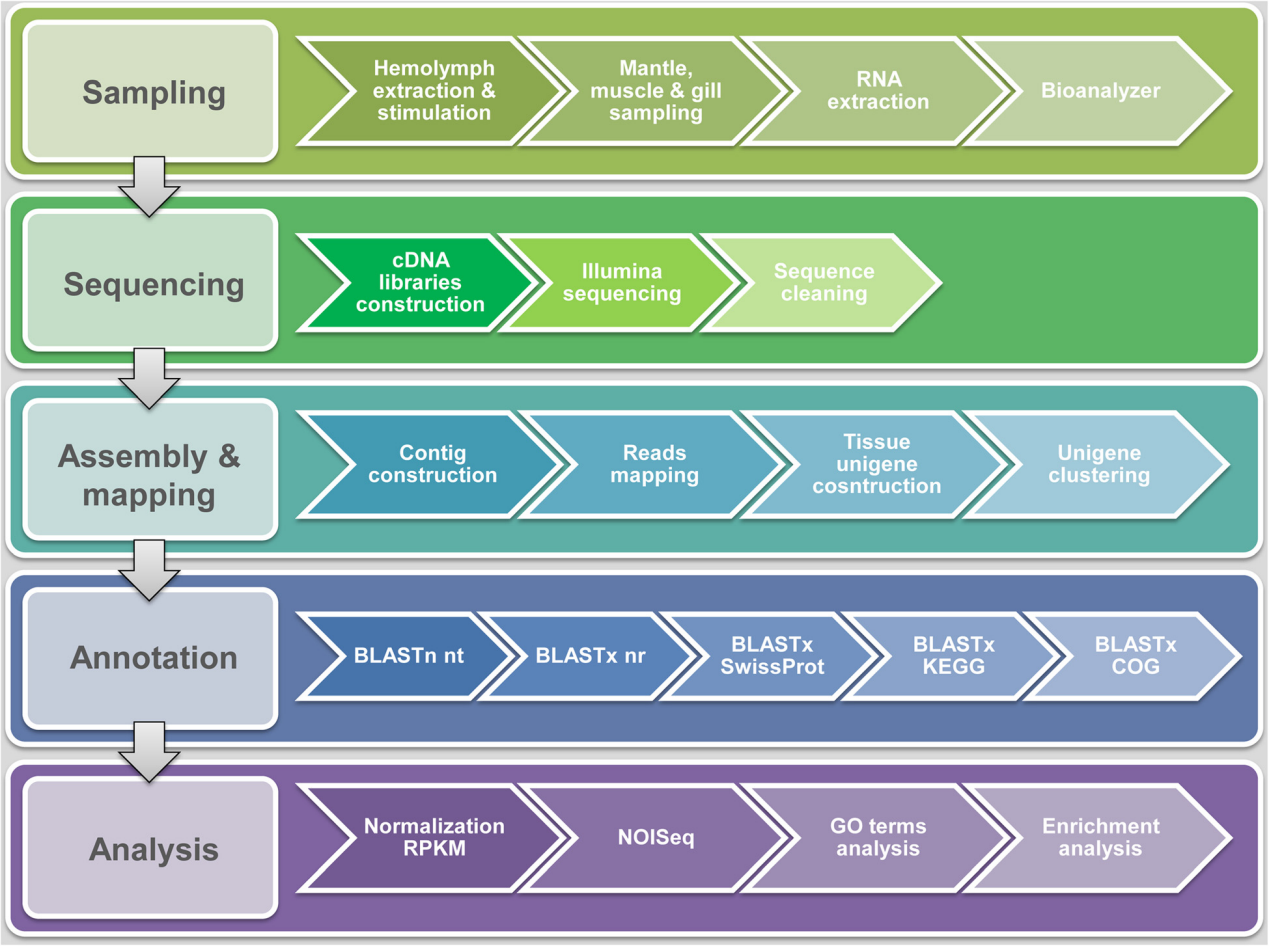
Flow chart summarizing the work tasks and the data processing pipeline

Mussel samples were processed as depicted in Fig. 1. The 7 cDNA libraries obtained (2 from stimulated hemocytes, 2 from mantle, 2 from muscle and 1 from gills) were sequenced on the Illumina HiSeq™ 2000 platform.

The sequencing and assembly statistics are summarized in Table 1. Briefly, we obtained a total of 393.3 million raw reads (with an average of 56.2 million reads per run). Of these, more than 95 % passed the quality standards and were subjected to further analyses. The filtered high-quality reads were assembled in a three-step approach with the Trinity software [34] into 1,242,475 contigs, which after clustering resulted in 479,806 unigenes. Until this point, the assembly protocol was individual for each sample, but the third and last clustering step was performed in common for the 7 samples. A total of 151,320 non-redundant unigenes (“transcripts” hereafter) were obtained, which could represent the *M. galloprovincialis* global transcriptome for these 4 tissues.

**Table 1:** Summary of sequencing and assembly data

Sequencing statistics	Hemocytes	Mantle	Muscle	Gill
Millions of raw reads	112.706	111.322	113.045	56.244
Millions of clean reads	107.386	106.060	107.127	53.335
Total Megabases	9,665	9,545	9,641	4,800
% GC content	38.99 %	38.32 %	38.28 %	37.54 %
Assembly statistics				
Number of contigs	261,332	428,939	313,554	238,650
Tissue unigenes	107,045	131,935	120,572	120,254
	All			
Total number of transcripts	151,320			
Average transcript length	570			
N50 transcript length	774			
Range of transcript lengths	200 – 17,690			
Number of transcripts < 500pb	104,757			
Number of transcripts > 500pb	46,563			
Annotation statistics				
Annotated transcripts by nt	14,207 (9.4 %)			
Annotated transcripts by nr	45,182 (29.8 %)			
Annotated transcripts by SwissProt	36,656 (24.2 %)			
Annotated transcripts by KEGG	31,144 (20.6 %)			
Annotated transcripts by COG	14,503 (9.6 %)			
TOTAL annotated transcripts	50,998 (33.7 %)			
Transcripts with GO terms	18,899 (12.5 %)			

The length of the transcripts ranged from 200 to 17,690 bp, with an average length of 570 bp, a similar size to that obtained with Roche 454 technology in other bivalves, e.g., 582 bp in the Manila clam [26]. Furthermore, when we compared our results with SOLiD and Illumina RNA-Seq analyses conducted in oyster, we obtained larger transcripts than those reported by Gavery and Roberts [28] or Zhao et al. [29], averaging 276 bp (554 using GigasDatabase v8 as a reference for mapping) and 322 bp, respectively.

The NCBI’s nucleotide and non-redundant, SwissProt, KEGG [35] and COG [36] databases were chosen to annotate the transcripts. The percentage of transcripts annotated with an e-value threshold of 1x10e^−5^ was 33.7 %. The annotations and expression values are included in Additional file 1. Our annotation percentage was similar to previous transcriptome studies conducted in bivalves using 454 technology [26], with 45 % of hemocyte transcripts being annotated with an e-value threshold of 1x10e^−3^. Similar approaches applied in oyster using the SOLiD [28] or Illumina [29] sequencing platform resulted in an annotation success of 41 % or 16 %, respectively, while in the *M. galloprovincialis* digestive gland transcriptome, about half (48.1 %) of the transcripts were successfully annotated [22].

The coverage of the whole transcript for each specific tissue sample (calculated as the percentage of base pairs in a transcript covered by reads of a specific sample per transcript length) is summarized in Table 2. The mean coverage was 69.87 %, with an average of 256.55 reads being mapped to each transcript, which is lower than the values reported in the oyster gills study by Gavery and Roberts [28], in which 454 reads per transcript were mapped (376 using GigasDatabase v8 as a reference). Hemocytes were the sample with the lowest coverage, but also showed the highest number of mapped reads per transcript. If this fact is related with the specific immune function of hemocytes, as was previously reported in other bivalves [26], is something that deserves further investigation.

**Table 2:** Coverage, mapping and new discoveries using a 2^nd^ replicate

	Average coverage of transcripts	Average unique mapped reads	New transcripts in the 2^nd^ replicate
Hemocytes1	67.58 %	279.50	8,890 (8.31 %)
Hemocytes2	62.94 %	334.05	
Mantle1	75.76 %	256.79	12,437 (9.43 %)
Mantle2	72.60 %	214.44	
Muscle1	69.48 %	248.45	13,726 (11.38 %)
Muscle2	66.73 %	221.61	
Gill1	73.99 %	241.03	-

We sequenced 2 samples of hemocytes, mantle and muscle, in order to understand whether a second biological replicate would effectively increase the sequencing depth (Table 2). Although this second replicate resulted in an average increase of 9.7 % transcripts for each tissue, it did not result in a significantly higher number of transcripts when all tissues were considered (Additional file 2). However, the use of pools of individuals, different tissues and biological replicates increased the reliability and robustness of the results, as previously reported [37]. For example, we achieved a transcriptome completeness of 88.71 % or 95.16 % (considering whole or partial sequence comparisons, respectively) using the CEGMA package (http://korflab.ucdavis.edu/datasets/cegma/).

### Qualitative description of the *M. galloprovincialis* transcriptome

**Fig. 2:**
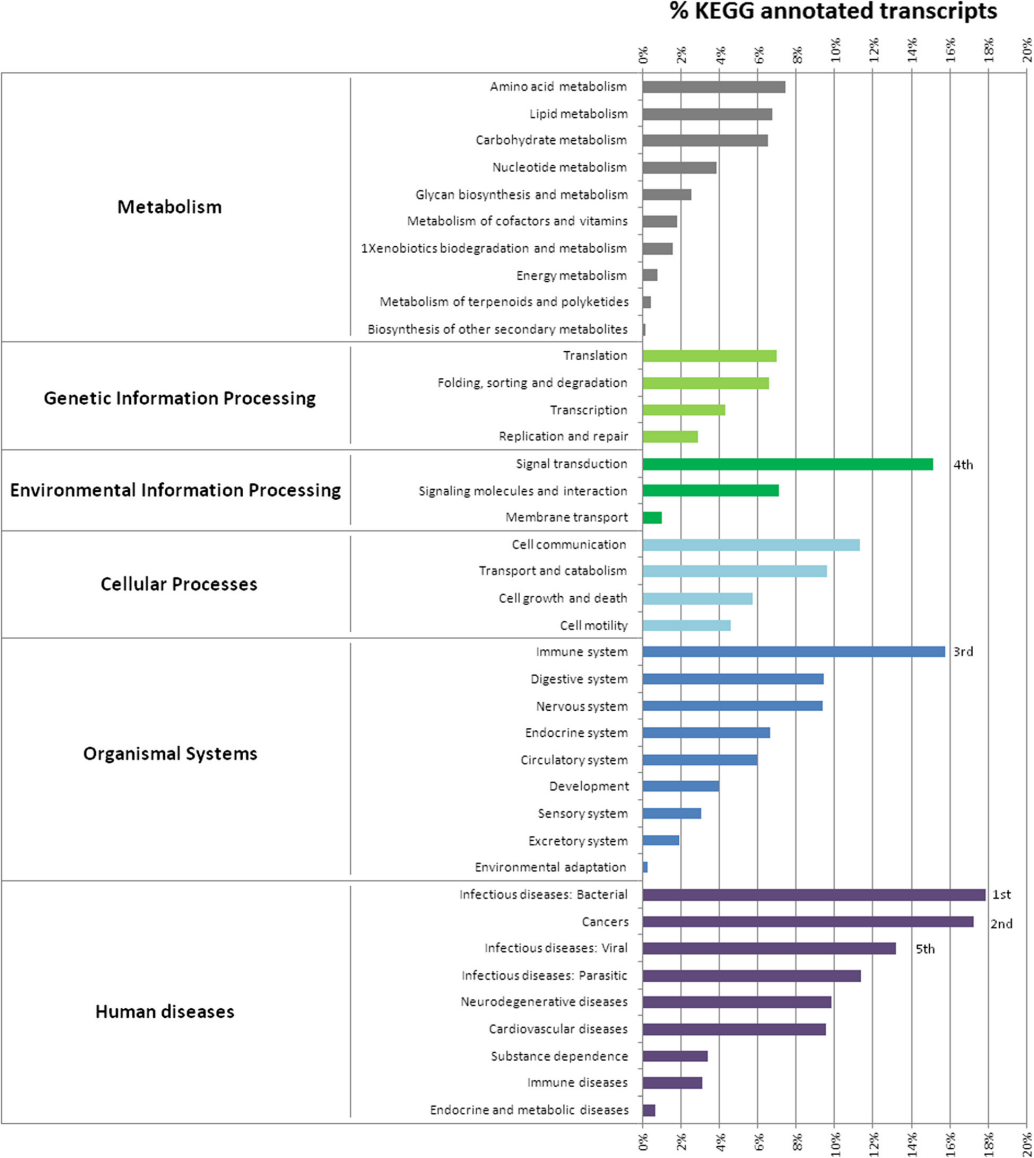
Summary of the KEGG reference pathway results. Bars represent the percentage of the total KEGG-annotated transcripts in the transcriptomes

**Fig. 3:**
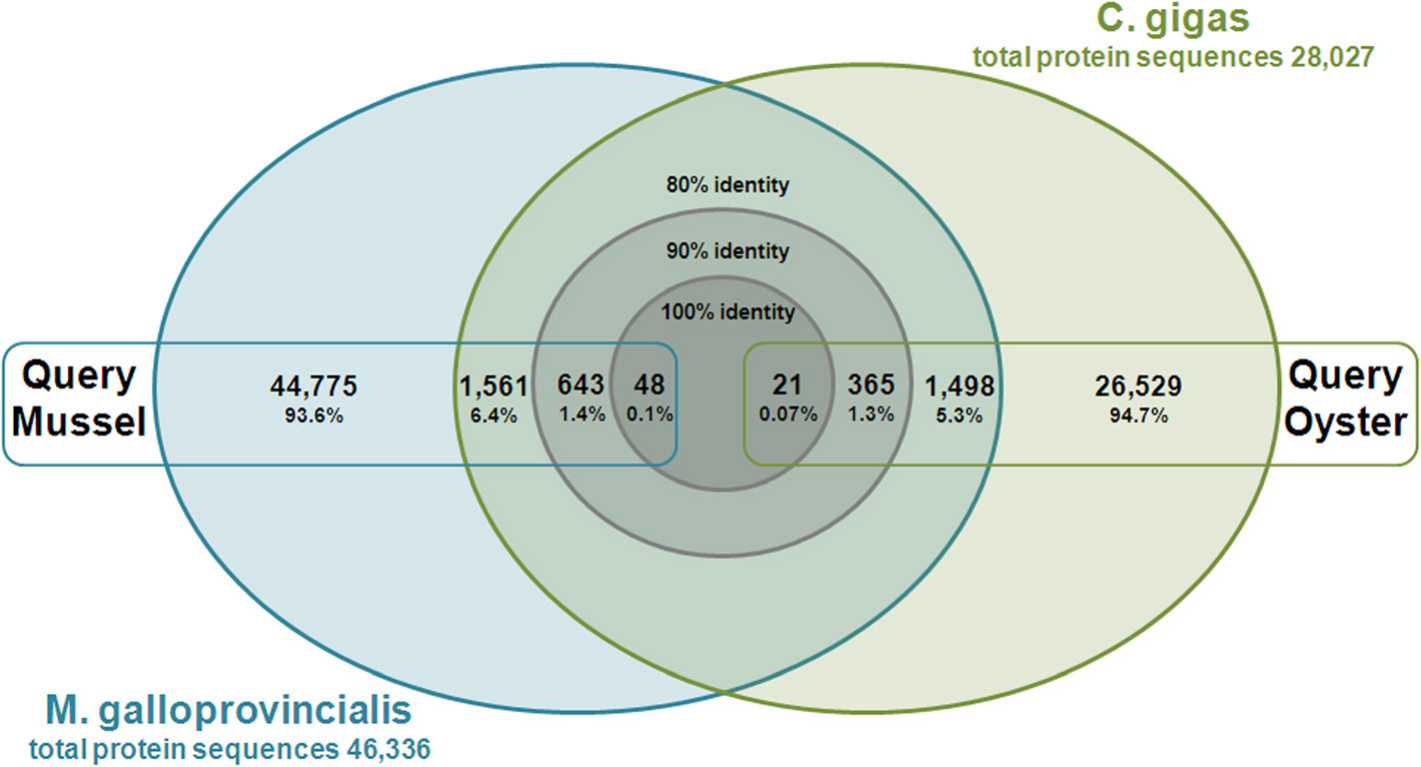
Comparison of the translated *Mytilus galloprovincialis* sequences with the *Crassostrea gigas* proteome downloaded from http://www.oysterdb.com/FrontDownloadAction.do?method=download

Using KEGG, we annotated 31,144 transcripts (20.6 %). This annotation served as a basis for analyzing not only the role of individual transcripts, but also the interaction with other genes. Figure 2 provides a representation of the global functionality of the transcripts and summarizes the 256 molecular pathways found in the transcriptomes. It was interesting that a high number of these transcripts had annotations related to the immune system, signal transduction and infectious diseases (bacterial, viral and parasitic). A possible explanation for this could be that, as a filter feeding animal, *M. galloprovincialis* is permanently in contact with microorganisms and with toxic/pollutant substances in their marine environment [38], and has adapted to become very resistant to these impacts [39, 40]. Another group of disease-associated pathways were those related to cancer, which ranked second among the most represented pathways, like in other studies in oysters [29]. Interestingly, although mussels and oysters shared less than 10 % of proteins with a sequence identity over 80 % (Fig. 3), the response to infectious disease and cancer were highly represented in both transcriptomes [29]. Although these cancer-related genes may have other functions, this subject obviously requires further attention specially taking into account that some bivalves are affected by a disease of the circulatory system closely resembling leukemia [41, 42].

The information about the molecules that were present and absent in each pathway is available in Additional file 3. The specialization and diversification observed throughout the phylogeny of the immune system [43] suggests that the absence of some key molecules in the pathways can be an artifact. It is possible that they were not annotated or that other molecules could play a similar function.

**Fig. 4:**
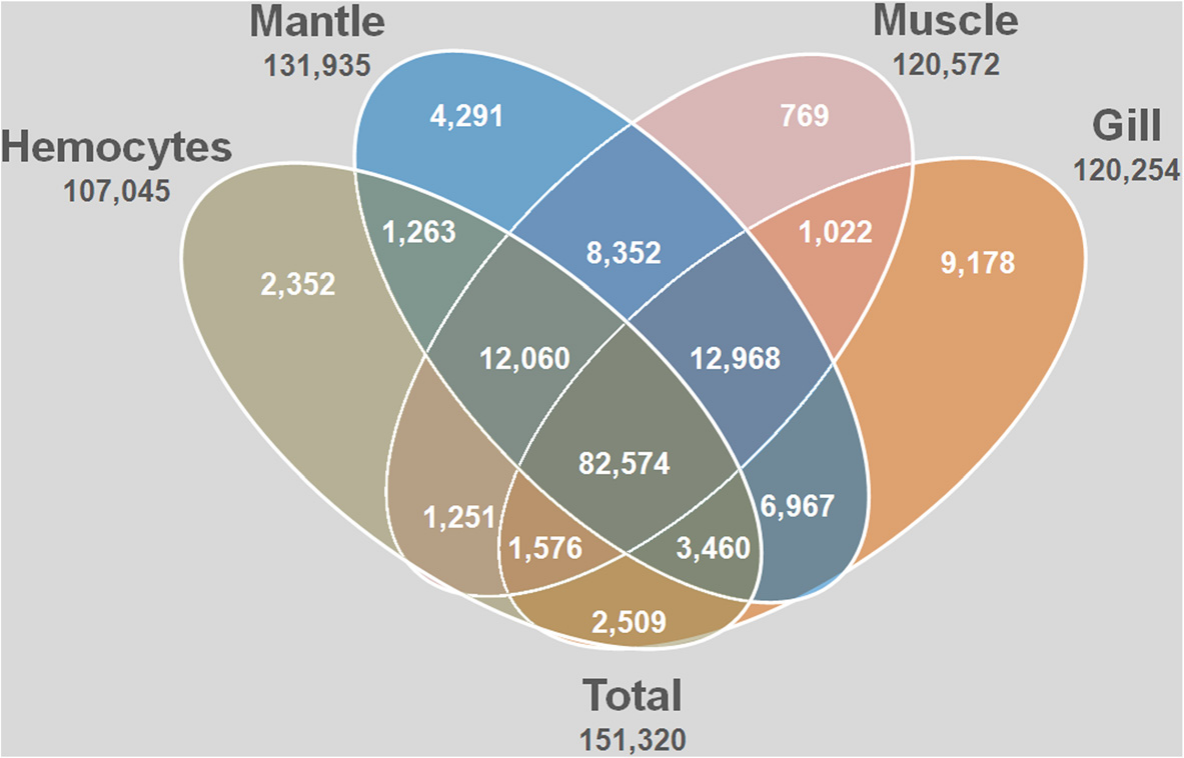
Venn diagram showing a comparison of the *R. philippinarum* tissue transcriptomes: hemocytes, mantle, muscle and gills. Numbers refer to the transcripts that belong to each group

A comparative analysis among the *M. galloprovincialis* transcriptomes was conducted to identify transcripts conserved in the 4 tissues and those unique to each tissue (Fig. 4). Among the total 151,320 transcripts, 54.57 % were shared by all the tissues. The most related pair of tissues, muscle and mantle, had 76.63 % transcripts in common, whereas hemocytes and gills shared only 59.56 % of the transcripts.

The tissue with the fewest private transcripts was muscle, with only 769 unique transcripts (0.51 %), while gills presented the highest number of non-shared transcripts, 9178 (6.07 %). This might be due to the filter feeding behavior of bivalves, where the gills are in constant contact with the surrounding habitat and exposed to more stress factors such as microorganisms, pollutants, pH or salinity changes.

Tissue-specific transcriptome portions are presented in Table 3. A high number of lectins, C1q domain-containing proteins and fibrinogen-related proteins were detected in gills. Their direct contact with the environment could explain the high presence of these putative recognition and immune-triggering molecules [11, 12, 44]. Hemocytes, as key players in the invertebrate immune response [45], showed a high percentage of antimicrobial peptides (AMPs), such as defensin, mytilins, and myticins, as well as other immune-related proteins, such as FREPs, serine protease inhibitors, complement component C4, HSP90 and C1q domain-containing proteins. Hemocyte hematopoiesis is a poorly described process in bivalves, but some studies suggest that heart and mantle could be possible hematopoietic tissues [46, 47]. In our case, peroxidasin transcripts, an early hematopoietic differentiation marker in *Drosophila* [48], were found only in the mantle, reinforcing the hypothesis that mantle could be the main hematopoietic tissue of bivalves.

**Table 3:** Top 25 non-shared transcripts

Reads	Hemocyte top 25 non-shared	Reads	Mantle top 25 non-shared
905.5	Apolipoprotein L	305	Von Willebrand factor D and EGF dom-contprot
501	PugilistDominant	293	Fibroin heavy chain
466	C1q dom-cont prot MgC1q28	283.5	C1q domain containing protein MgC1q95
300	Defensin	245	Nacrein B3
277.5	Toxin CrTX-A	215	ADAM family mig-17
268	Mytilin B	165.5	Fibrocystin L
255	Conodipine-M alpha chain	160.5	Gigasin-6
238.5	DNA ligase 1	152.5	C1q domain containing protein MgC1q69
159	Fibrinogen-related protein	149	ATP-dependent RNA helicase A
146	Mytilin-6	132	GTPase IMAP family member 8-like
145.5	Transcription antiterminator	120.5	Lactase-phlorizin hydrolase
141	Serine protease inhibitor Cvs.i-2	120.5	Processed variable antigen (Fragment)
125	Rossmann fold nucleotide-binding protein	116	TPR repeat
121	Reverse transcriptase-like protein	115.5	Peroxidasin homolog (Drosophila)-like
112.5	Complement component 4	114	Beta-hexosaminidase
107	ATP synthase subunit a	105	Nicotinic acetylcholine receptor alpha subunit
92	Heat shock protein 90 (HSP90-2)	103	LDL receptor-related protein 8 (LRP8)
81.5	Pol-like protein	101	Electrogenic NBC-like protein
73.5	C1q dom-cont prot MgC1q56	99.5	Basic proline-rich protein
72.5	Ribosome-associated protein Y (PSrp-1)	99.5	Inter-alpha-trypsin inhibitor heavy chain H5
70	ATP-dependent RNA helicase ddx41	94.5	Golgi-associated plant pathogenesis-related protein 1
69.5	Myticin C	94	Fatty acid synthase
63	Nephrin	93.5	Myb-related transcription factor, partner of profilin
61.5	Cytosolic phospholipase A2	91.5	C1q domain containing protein MgC1q48
56.5	Fibrinogen-related protein (FREP_G1)	89	RING finger protein 13
Reads	Muscle top 25 non-shared	Reads	Gill top 25 non-shared
107	Ribosomal RNA	6644	Perlucin-like protein
62	Heat shock protein 90 (HSP90-2)	3223	C1q domain containing protein MgC1q71
48	Mammaglobin-A precursor	2935	Yolk ferritin
44.5	Gill symbiont ribosomal RNA	1655	WSC domain-containing protein 2
36.5	28S ribosomal RNA gene, partial sequence	1423	Short-chain collagen C4 (Fragment)
31	Myticin C	1407	Apextrin-like protein
30.5	Angiopoietin-4	1342	Fibroin heavy chain
30	Basal body protein NBP-2	1248	GTPase IMAP family member 4
27	Stress-70 protein, mitochondrial-like	1030	Nicotinic acetylcholine receptor alpha subunit
25.5	Ficolin-2-like, partial	997	Fucolectin
23.5	Collagen alpha-2(I) chain	989	Collagen alpha-1(XII) chain
17.5	16S ribosomal RNA	969	Multiple EGF-like domains protein 6-like
17.5	Rps19	916	C1q domain containing protein MgC1q17
17	ABC protein, subfamily ABCC	897	C1q domain containing protein MgC1q52
17	Catecholamine binding protein	889	C1q domain containing protein MgC1q36
15.5	Oxidoreductase, FAD/FMN-binding family protein	879	Eggshell protein
14.5	Large exoprotein involved in heme utilization or adhesion	875	GTPase IMAP family member 7
14.5	Ribulose-phosphate 3-epimerase,	858	C1q domain containing protein MgC1q81
12.5	C1q domain containing protein MgC1q22	853	Codakine
11.5	Ribosomal protein L32	825	Cathepsin L
11.5	Small heat shock protein hspI, mitochondrial	799	C-type lectin
11	DnaJ homolog dnj-10	795	Fibrinogen-related protein
10.5	GrpE-like protein	772	Fibrinogen C domain-containing protein 1
10.5	Zn-finger domain associated with topoisomerase type I	742	Tetraspanin-CD63 receptor
9.5	Macrophage receptor MARCO	728	Calmodulin

Reads are the averaged read number per tissue library

The lowest number of tissue-specific transcripts was observed in muscle, which could be due to its limited functional diversity (Fig. 4). In this case, the most abundant transcripts corresponded to heat shock proteins (HSP90, HSP70, HSP40, HSP20, HSP24 and GprE) and ribosome-related sequences. Interestingly, some immune-related molecules, such as myticin C, ficolin-2, C1q domain-containing protein and the scavenger receptor MARCO, were also present in the muscle. In mammals, MARCO is a pattern recognition receptor for Gram-positive and negative bacteria expressed mainly in macrophages [49] and it has not been described in invertebrates to date.

Some tissue-specific transcripts presented different variants depending on the tissue. This could mean that the gene is not tissue-exclusive but instead tissue-exclusive variants may exist. This was clearly observed for the C1q domain-containing proteins, a group of molecules that show high variability in *M. galloprovincialis* [12]. The C1q annotation for all the non-shared transcripts did not coincide between tissues (Additional file 4), which could suggest a high specialization of this molecule in each tissue and, possibly, specialization for non-self recognition, as might be inferred from the high abundance of the C1q sequences in gills. Without the *M. galloprovincialis* genome we can not know if the C1q variants are different isoforms or belong to different loci, further research is needed to clarify this issue.

### Quantitative analysis between tissues: RNA-Seq

**Fig. 5:**
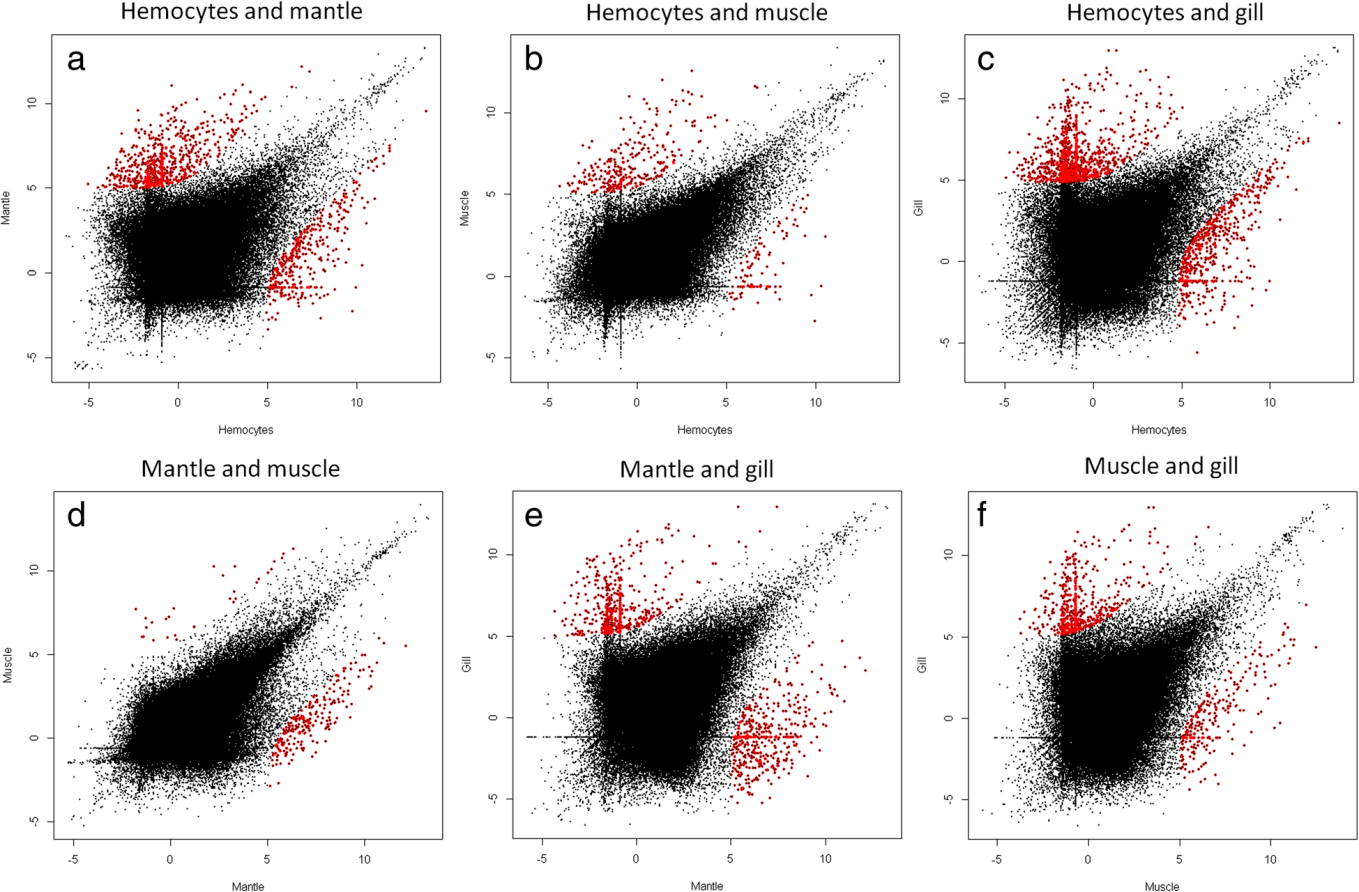
NOISeq log2-transformed expression charts. Red indicates the differentially expressed genes (d.e.g.) with a *p*-value < 0.01. **a**. Hemocytes and Mantle; **b**. Hemocytes and Muscle; **c**. Hemocytes and Gills; **d**. Mantle and Muscle; **e**. Mantle and Gills; **f**. Muscle and Gills

The transcriptomes were also quantitatively analyzed. We first normalized the number of reads that were mapped to each transcript into RPKM units (Reads Per Kilobase of exon model per Million mapped reads). To evaluate differentially expressed genes (d.e.g.) among tissues we used NOISeq [50], a nonparametric statistical approach that presents a low false discovery rate. The expression distribution of all the transcripts is showed in Fig. 5. As it is reflected by the red color intensity in each chart, the most similar tissues are mantle and muscle (d) while the most different are hemocytes and gills (c). The pairwise comparisons between the four tissues are summarized in Table 4. In the tissues that exhibited more transcripts in common (mantle and muscle) only 256 d.e.g. were found. In contrast, the comparison between hemocytes and gill, the tissues with the most dissimilar transcriptomes, revealed almost 2000 significantly different transcripts.

**Table 4:** Number of differentially expressed genes between tissues

Analysis	*p*-value0.01	
	Total	Annotated
Hemocytes vs. Mantle	1,086	707 (238 h + 469 m)
Hemocytes vs. Muscle	399	264 (55 h + 209mu)
Hemocytes vs. Gills	1,928	1,040 (357 h + 683 g)
Mantle vs. Muscle	256	169 (149 m + 20mu)
Mantle vs. Gills	1,016	566 (285 m + 281 g)
Muscle vs. Gills	905	496 (182mu + 314 g)

*h* hemocytes; *m* mantle; *mu* muscle; *g* gills

**Fig. 6:**
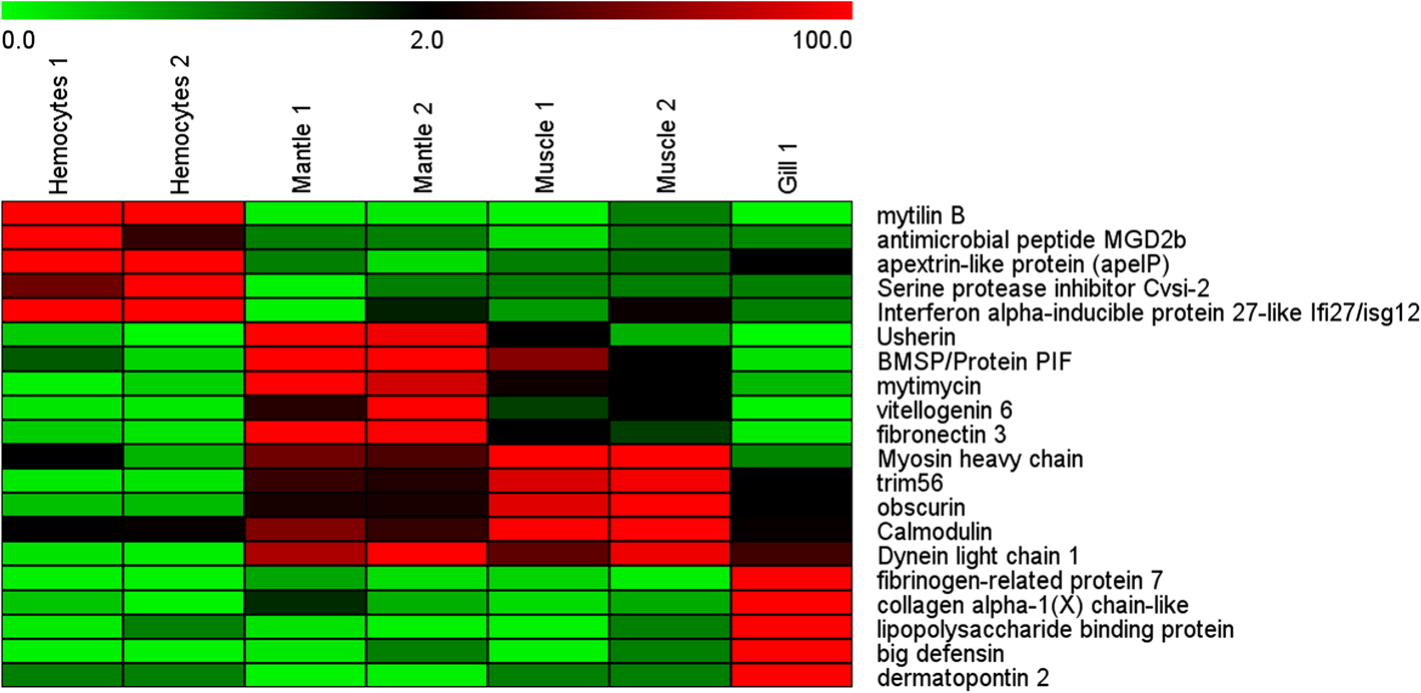
Heatmap of a selection of 5 of the most highly expressed genes by tissue, which shows the expression level of all the biological replicates used in this study. The scale bar is a non-linear representation of the normalized expression: Saturated green: no expression, 0 %. Black: 2 % of maximum expression. Saturated red: maximum expression, 100 %

The heatmap provided in Fig. 6 illustrates, as an example, the quantitative expression of 5 among the top-expressed genes in each tissue, showing also the high reproducibility of the two biological replicates.

Table 5 shows the 25 most highly expressed genes in each tissue compared to the other 3 transcriptomes. The top d.e.g. in hemocytes were immune-related, including AMPs, such as myticin A, mytilin B, mytilin 6 and 7 or defensin 2b; pore-forming molecules, such as apextrin and MAC/perforin; lectins (C-type, nacre protein, macrophage mannose receptor) and many other genes that are directly or indirectly related to the immune system, such as ADAMTS16, a metalloprotease required for remodeling the basement membrane during cell migration [51] (Table 5). This list also included C1q, a serine protease inhibitor that modulates host-pathogen interactions [52] and HSP70, ISG12 and IAP, which play important roles in apoptosis and immunity [53–55]. The expression fold change was relatively high, varying approximately between 200 and 2000. Nevertheless, we have to consider that hemocytes were stimulated with different treatments whereas the other tissues were sampled from unstimulated mussels.

**Table 5:** Top 25 differentially expressed transcripts

FC	Hemocyte top 25 expressed	FC	Mantle top 25 expressed
1,820	Mytilin B	5,008	Usherin
750	Procollagen type VI alpha 4	2,702	Mitochondrial glycine cleavage system H prot
744	Metallothionein MT-20	2,521	BMSP / Protein PIF
709	C-type lectin	1,992	MAM dom-cont glycosylphosphatidylinositol anchor protein 2
685	Disintegrin and metalloproteinase with thrombospondin motifs 16 (ADAMTS16)	1,951	Protocatechuate 3,4-dioxygenase beta subunit
588	Cystatin-A-like	1,795	Collagen alpha-1, IV/III
530	Melatonin receptor-like (1A/1B)	1,758	Keratin, type II cytoskeletal 2 epidermal
508	Nacre protein	1,618	Fibroin heavy chain
478	Defensin 2b (MGD2b)	1,552	ATP-dependent RNA helicase A
465	C1q domain containing protein	1,541	Endo-1,4-mannanase
428	Mytilin-6	1,489	Heterogeneous nuclear ribonucleoprotein A3
407	Spermine oxidase	1,489	Mytimycin
383	Gly, Ala and Asn-rich protein	1,489	Splicing factor 3A subunit 2
357	Apextrin-like protein (apelP)	1,409	L-rhamnose-binding lectin CSL3
320	Mucin-2	1,389	Sarcoplasmic calcium-binding protein
296	Mytilin-7	1,314	Protein diaphanous
269	Macrophage mannose receptor 1-like	1,314	Vitellogenin 6
260	Serine protease inhibitor Cvs.i-2	1,261	Fibronectin 3
258	MAC/perforin- and kringle-dom-cont prot	1,218	Heat shock protein 70
252	Peptide O-xylosyltransferase	1,193	Whey acidic protein-like
251	MAM and LDL-receptor class A dom-cont prot	1,184	Porin-like
246	Heat shock protein 70	1,184	Hornerin / filaggrin
242	Myticin-A	1,168	Chitinase 3
241	Interferon alpha-inducible protein 27 2B (IFI27/ISG12)	1,144	Protein unc-93 homolog A
237	Inhibitor of apoptosis 7B/2/3	1,136	TPA: SCO-spondin protein
FC	Muscle top 25 expressed	FC	Gill top 25 expressed
2,702	Collagen alpha-3/6(VI) chain	3,083	Inner centromere protein A
1,226	Myosin heavy chain	2,837	BMSP / protein PIF
1,128	Heat shock protein 70	2,817	Perlucin
861	Collagen pro alpha-chain	2,592	Caveolin-1/3
787	C1q domain containing protein	2,120	Peptide O-xylosyltransferase
776	Tripartite motif-containing protein 2/56 (TRIM2/56)	2,048	Endonuclease domain-containing 1 protein
695	Proteoglycan 4	1,872	Insulin-like growth factor binding protein 2b
690	Protein LEA-1	1,833	Fibrinogen-related protein 7
662	Sushi, VWF type A, EGF and pentraxin dom-cont prot 1 (SVEP1)	1,771	Synaptotagmin
657	Beta-glucanase/Beta-glucan synthetase	1,734	Collagen alpha-1(X) chain-like
644	Enzymatic glycosylation-regulating-like	1,722	LDL receptor-related protein 8 (LRP8)
553	Fatty acid-binding protein homolog 9/7	1,652	Suppressor of tumorigenicity 14 protein (ST14)
530	Nucleolar protein 12	1,552	Antistasin
488	Forkhead box L2	1,479	Collagen triple helix repeat protein
481	Obscurin	1,458	LPS binding protein / Bactericidal permeability-increasing protein
471	Calmodulin	1,305	Viral A-type inclusion protein repeat
471	Dynein light chain 1	1,269	Big defensin
465	Angiopoietin-4	1,269	Notch gene homolog 3-like
465	GTP-binding protein REM 1	1,261	Alpha 1 type V collagen
461	Synaptopodin 2	1,243	Golgin subfamily A member 4
458	Myosin light chain	1,243	Apextrin-like protein
452	Calpain-5	1,235	Short-chain collagen C4
443	Plasminogen	1,209	Dermatopontin 2
440	Paramyosin	1,184	Stanniocalcin
428	BTB/POZ domain-containing protein KCTD7	1,160	Tolloid-like 1 precursor (TLL1)

*FC* fold change

In mantle the most highly expressed protein was usherin, showing a fold change of 5008 (Table 5). This protein is involved in visual and auditory transduction in mammals [56]. Other bivalves, such as scallops, possess ‘eyes’ at the mantle edge that influence their relationship with the environment [57]. The presence of this highly expressed gene in the mantle suggests that it might play a sensory role, in addition to its shell-forming and reproduction functions, which are also represented by genes such as vitellogenin 6, which is a precursor to egg-yolk proteins during embryonic development [58], or fibronectin 3, which is involved in shell formation in bivalves but also in mammal spermatogenesis [59, 60]. Interestingly, 3 of the 25 most highly expressed genes in mantle were related to antifungal functions or chitin metabolism: the PIF protein, mytimycin and chitinase 3, which showed fold changes of over 1000 compared with the other tissues. The shell of bivalves is a substrate for epibiotic communities, including fungi. Some fungi possess the ability to penetrate into the internal organs of animals and cause mycoses if the host-pathogen relationship is altered [61]. Therefore, the shell and the mantle could represent the first antifungal barrier, which would explain the presence of these d.e.g.

The muscle showed many typical myofibril molecules presenting fold changes of over 400, such as myosin light and heavy chain; paramyosin, which is typical of invertebrates; obscurin, which is involved in myofibrillogenesis [62]; calcium-related proteins, such as calmodulin or calpain, which are linked to muscle remodeling and contraction [63, 64]; and angiogenesis- and migration-related genes, such as angiopoietin-4 [65] and viral response molecules (e.g., TRIM56) [66], which exhibited expression increases of 465 and 776 fold, respectively (Table 5). These results suggest other possible functions of muscle in bivalves, as mentioned above.

The expression profile observed in gills confirmed previous studies showing that collagen is a major compound of this tissue [67]. Collagen was represented at more than 1000 fold the levels found in the other tissues, and showed higher levels than other extracellular matrix-related genes, such as dermatopontin, ST14 and TLL1. Dermatopontin accelerates and stabilizes collagen fibril formation, but this protein also presents other functions that are closely related to immune defense, such as cell adhesion via integrin binding, enhancing Transforming Growth Factor β1 activity or inhibiting cell proliferation [68]. ST14 degrades the extracellular matrix [69] and TLL1 processes procollagen C-propeptides [70]. However, as previously noted, the gills showed a significant expression of some immune-related molecules, such as the PIF protein, perlucin, LPS binding protein, big defensin or apextrin, which displayed fold changes ranging from 1243, in the case of apextrin, to 2837, in the case of PIF.

### Enrichment analyses to compare qualitative and quantitative results

**Fig. 7:**
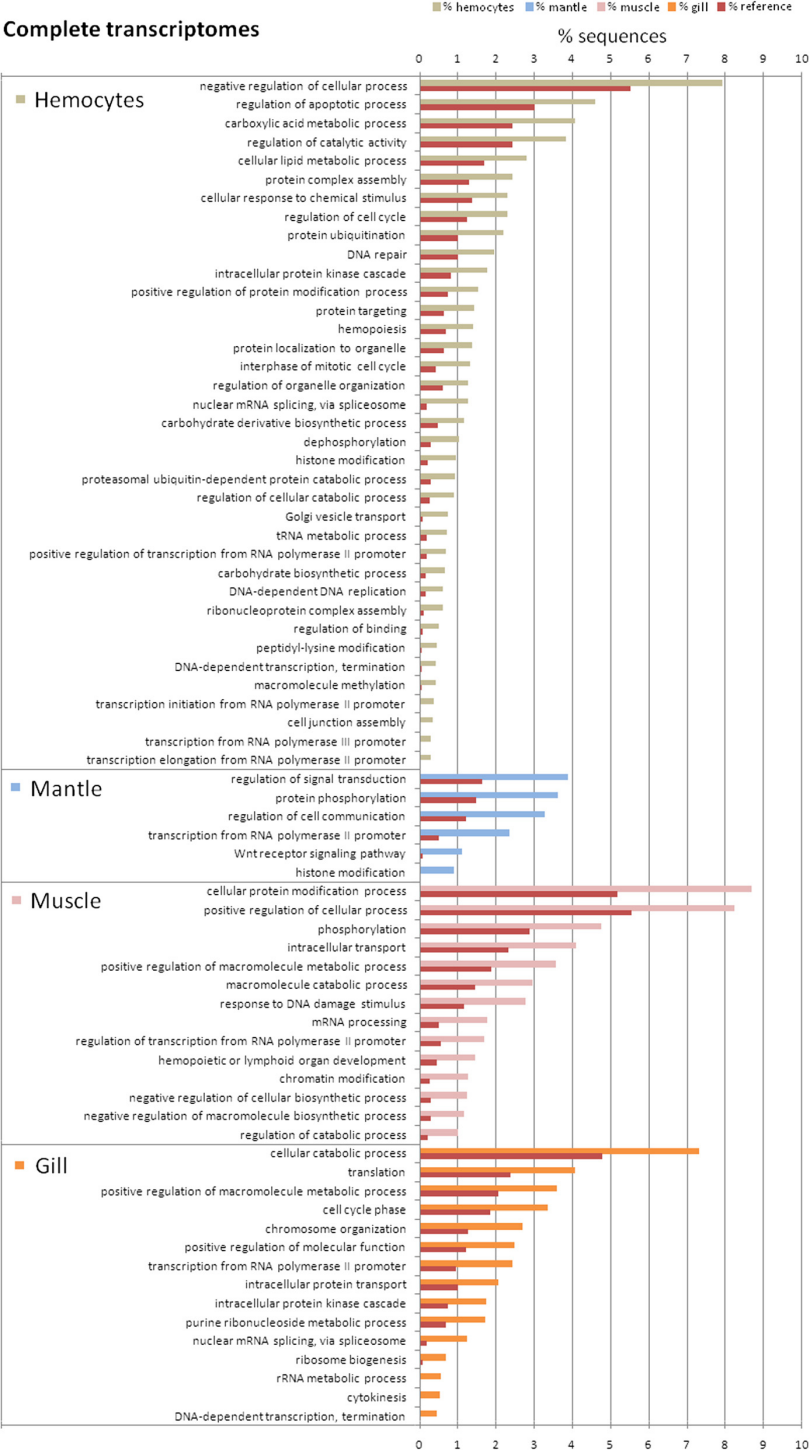
Classification of the complete transcriptomes by tissue type after a Blast2GO Enrichment Analysis. Only overrepresented biological process GO terms are shown

**Fig. 8:**
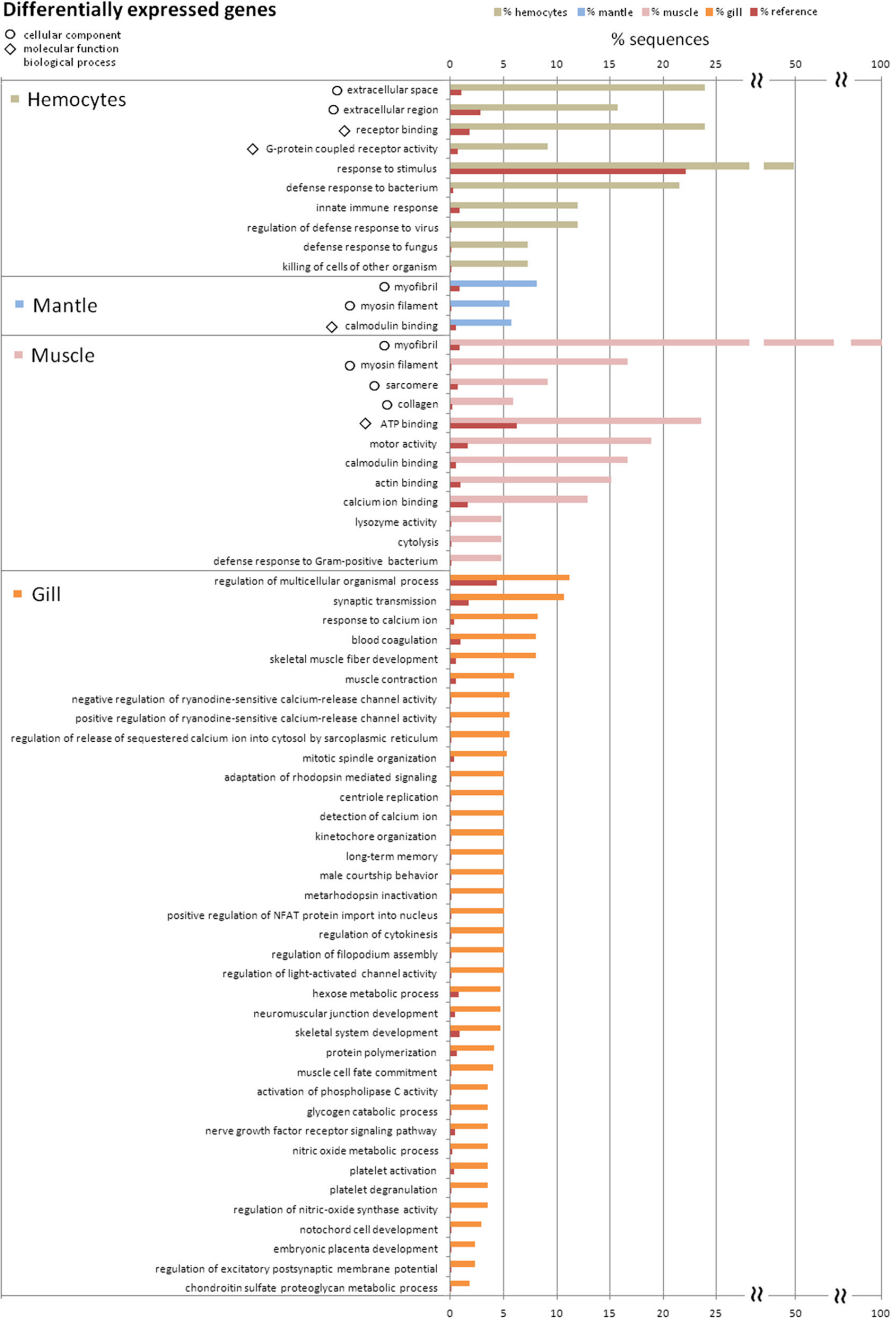
Classification of the d.e.g. by tissue after a Blast2GO Enrichment Analysis. Only overrepresented GO terms are shown and for the gills, only the biological process enriched GO terms are shown

Gene Ontology (GO) terms were assigned to the non-redundant transcripts. A total of 18,899 (12.5 %) transcripts matched at least one GO term, which is twice as much as what was obtained in other reported Illumina transcriptomes (6 % GO annotation) [29]. This GO information was used to identify overrepresented biological processes in each transcriptome and in each group of d.e.g. by tissue. The results are summarized in Fig. 7 for the different transcriptomes and in Fig. 8 for the d.e.g. Figure 8 also shows overrepresented cellular components and molecular functions of hemocytes, mantle and muscle transcriptomes. First, it is important to note the large differences in enriched terms when the whole transcriptomes are compared with those of the d.e.g., which do not present a single term in common. The complete transcriptomes appeared to show more general functions, such as metabolism, transport or transcription, whereas the differentially expressed transcriptomes presented more detailed terms and functions, such as defense or regulation of specific signaling processes.

Mussel hemocytes exhibited the most divergent transcriptome due to the large number of significantly enriched processes found (Fig. 7). These processes included immune-related functions, regulation of the apoptotic process, cellular response to chemical stimulus, intracellular protein kinase cascade and hemopoiesis. Other functions regarding cell proliferation (regulation of cell cycle, DNA-dependent DNA replication, DNA repair) or migration (cell junction assembly) were also found to be overrepresented. In contrast, the hemocyte d.e.g. (Fig. 8) showed a quite different profile, with a high representation of categories related to the immune response, such as innate immune response (12 % of d.e.g.), defense response to bacterium (21 %), regulation of immune defense to virus (12 %) or defense response to fungus (7 %). Figure 8 provides other interesting results as well; for example, almost 50 % of the hemocyte d.e.g. had functions involved in the response to stimulus, and over 20 % of them had ontologies for extracellular proteins and functions related to receptor binding.

The mantle transcriptome showed some remarkable enriched processes (Fig. 7), such as the evolutionarily conserved Wnt receptor signaling pathway, which plays a key role in development, including stem cell proliferation and cancer [71]. This finding is also congruent with the mantle being the hematopoietic tissue in mussels. Moreover, the differentially expressed transcriptome of the mantle confirmed the functional similarity between the mantle and muscle, as it included three GO terms related to muscle contraction: myofibril, myosin filament and calmodulin binding (Fig. 8). The results of the muscle d.e.g. enrichment analysis fully coincide with the mantle analysis, adding more terms related to contraction, such as motor activity, sarcomere, actin binding or calcium binding (Fig. 8). The complete muscle transcriptome, as well as the transcriptomes of the other tissues, presented general processes such as metabolism or transcription, but also two specific processes: response to DNA damage stimulus and hemopoietic or lymphoid organ development, including differentiation of resident and migratory cell types (Fig. 7). The GO terms related to immune response may be due to the normal presence of some hemocytes in the muscle.

The enriched functions of the gill transcriptome showed a similar, but reduced profile compared with that from hemocytes (Fig. 7). The gill transcriptome was not as closely related to immunity as it was to signaling (intracellular protein kinase cascade) and cell proliferation (cell cycle phase, chromosome organization and cytokinesis), which were most likely overrepresented due to the direct contact of this tissue with the environment, as such contact could lead to a regular renewal of the tissue. The enrichment analysis of the gill d.e.g. (Fig. 8) produced the highest number of results among all the analyzed data, with 37 GO biological process categories being overrepresented. All of these processes can be grouped into three main categories: calcium homeostasis, coagulation and defense, which are intimately related to each other. The identified coagulation processes (blood coagulation, platelet activation and degranulation) could also be included in the defense group because coagulation triggers the complement cascade [72] and is critical in immune defense, as well as the production of toxic radicals such as nitric oxide (NO) (represented with the categories nitric oxide metabolic process and regulation of nitric oxide synthase activity), which has been shown to occur in the gills. NO production is known to be up-modulated in bivalves stimulated with bacteria and parasites [73, 74]. Calcium homeostasis processes were clearly represented in the gill d.e.g. (Fig. 8), such as the detection and response to calcium ions, regulation of the release of sequestered calcium and activation of phospholipase C activity. In addition to their role in gas exchange, gills exhibit osmoregulatory, ion transport and homeostasis functions in crustaceans and fish [75, 76]; however, these functions have not been studied in bivalves. The cells involved in these processes in fish are ionocytes, a mitochondria-rich cell (MRC) type. In bivalves, three types of MRCs are present in the gills [67]. These factors suggest that there is calcium homeostasis activity in mussel gills.

## Conclusions

We have shown the value of whole-transcriptome analysis generated via RNA-Seq for accurate quantification of gene expression. Using almost 400 million reads, we described the transcriptome and expression profiles of *M. galloprovincialis* tissues and the generated data has enriched the genomic resources available for this organism.

This study represents the first RNA-Seq approach applied in bivalves to describe and analyze tissue-specific transcriptomes. We identified a high number of transcripts related to the immune system, signal transduction and infectious diseases that highlight immune functions in all the tissues studied, probably as a result of mussel’s open circulatory system. Another group of disease-associated pathways were those related to cancer, which ranked second among the most represented pathways. Moreover, we also found specific and unexpected functions in specific tissues: mussel hemocytes showed the greatest number of antimicrobial and defense proteins; mantle appeared to exhibit a more specific antifungal function and even to be a firm candidate of the hematopoietic tissue; gills presented a large number of putative recognition molecules; and muscle expressed stress- and defense-related proteins.

Our results shed light into the transcriptomics and physiology of the Mediterranean mussel. This species has a great economical and ecological importance, it has been extensively used as pollution sentinel and the present findings related to immunity, hematopoiesis and cancer confirm that *M. galloprovincialis* is a very interesting candidate to be the model species for bivalves and even molluscs. The mussel genome project, that will come soon, will further support this candidature.

## Methods

### Tissue sampling, *in vitro* stimulation of hemocytes and RNA isolation

*M. galloprovincialis* mussels were obtained from a commercial shellfish farm (Vigo, Galicia, Spain) after depuration. The animals were maintained in open-circuit filtered sea water tanks at 15 °C with aeration and were fed daily with *Phaeodactylum tricornutum* and *Isochrysis galbana* until 2 days before sampling. Prior to the experiments, the mussels were acclimatized to aquarium conditions for one week.

The mantle, muscle and gill tissues from 5 mussels were sampled, pooled and conserved in 1 ml of TRIzol (Invitrogen). All samplings were performed as 2 biological replicates from all the tissues, except for the gills (which included only 1 biological replicate).

For hemolymph collection, approximately 50 mussels were notched in the shell and hemolymph (1–3 ml) was withdrawn from the adductor muscle of each mussel with a 0.5-mm-diameter (25G) disposable needle. The hemolymph was pooled and distributed in 6-well plates, with 7 ml per well, in a total of 9 wells, one for each treatment. The hemocytes were allowed to settle to the base of the wells for 30 min at 15 °C in the dark. Then, the hemocytes were stimulated for 3 h at 15 °C with 50 μg/ml polyinosinic:polycytidylic acid (Poly I:C), peptidoglycans (PG), zymosan, *Vibrio anguillarum* DNA (CpG), lipopolysaccharide (LPS), lipoteichoic acid (LTA), 100 ng/ml flagellin and 1 x 10^6^ CFU/ml of heat-inactivated *Vibrio anguillarum* (one stimulus per well). The last group of hemocytes remained unstimulated. All the stimuli were purchased from SIGMA, except for CpG and *V. anguillarum*, which were produced in our laboratory. This procedure was performed twice to obtain 2 biological replicates. Hemolymph was centrifuged at 4 °C at 3000 g for 10 min and the pellet was resuspended in 500 μl of TRIzol (Invitrogen).

From this step onwards the methodology used was the same for all the tissues. Total RNA isolation was conducted following the manufacturer’s protocol using the RNeasy Mini kit (Qiagen) for RNA purification after DNase I treatment. Next, the concentration and purity of the RNA were measured using a *NanoDrop ND1000* spectrophotometer. Finally, RNA integrity was tested on an Agilent 2100 Bioanalyzer (Agilent Technologies) to produce cDNA libraries for Illumina sequencing.

### cDNA production and Illumina sequencing

The mRNA-Seq sample preparation kit from Illumina was used according to the manufacturer’s instructions. Briefly, eukaryotic mRNA was extracted from total RNA using oligo (dT) magnetic beads and cleaved into short fragments using fragmentation buffer. A cDNA library compatible with the Illumina NGS technology was then prepared from the fragmented mRNA via reverse transcription, second-strand synthesis and ligation of specific adapters (paired-ends) after cDNA purification using the QIAquick PCR Purification Kit (Qiagen). The amount of cDNA in each library was quantified through spectrofluorometric analysis using the Qbit system. Next-generation sequencing was performed using Illumina HiSeq™ 2000 technology at the Beijing Genomics Institute (BGI-HongKong Co., Ltd., Tai Po, Hong Kong).

### Bioinformatics workflow

#### Assembly and functional annotation

The image data output from the sequencing apparatus was transformed via base calling into raw data and stored in FASTQ format. The raw data were cleaned with filter_fq software to discard low-quality reads, reads with regions with greater than 5 % unknown bases or reads with adapters.

*De novo* transcriptome assembly was conducted with the short reads assembly program Trinity [34, 77] (minimal contig_length: 100; group_pairs distance: 250; minimal kmer_cov: 2). Trinity first combined overlapping reads to form contigs with at least a 100-bp length and a minimum of 2 reads to be assembled. Then, the contigs were assembled again to obtain longer sequences that could not be further extended, which are unigenes. During this process and before obtaining the final unigenes, the reads were mapped against the contigs to confirm the assembly procedure. When multiple samples from the same species are sequenced (biological replicates or different tissues), unigenes from each sample can be applied together to perform another assembly step. This process detects sequence splicing and redundancy to acquire the longest sequences and group them into clusters. Each cluster is formed by several unigenes with more than 70 % similarity. To simplify the terminology employed in this study, all the non-redundant sequences will be called “transcripts”, regardless of whether they are unique unigenes or belong to a cluster. The completeness of the mussel transcriptome was confirmed with the CEGMA package (http://korflab.ucdavis.edu/datasets/cegma/).

A total of 151,320 transcripts were obtained following this protocol. This number represents all the detectable variability in the mRNAs from the four studied tissues, including splicing variants, non-overlapping fragments of the same mRNA, UTRs or mRNAs in different splicing stages.

The transcripts were first annotated using BLASTx and BLASTn (with an e-value threshold of 10e^−5^) against the NCBI nr, Swiss-Prot, KEGG and COG protein databases and the NCBI nt nucleotide database. The annotation step provided the identity of the transcript with the species harboring the matching sequence, which is useful for detecting possible contaminants in our samples. Using the KEGG database information, the metabolic pathways and functions of the annotated transcripts could be obtained and presented.

The oyster proteome was downloaded from http://www.oysterdb.com/FrontDownloadAction.do?method=download and compared with the translated mussel transcripts.

#### RNA-Seq with NOISeq: Quantitative analysis between tissues

RNA-Seq compares the number of reads that align to a specific transcript in different samples or cDNA libraries. The calculation of expression uses the RPKM (Reads Per Kilobase of exon model per Million mapped reads) normalization, while accounting for the length of the transcript that they belong to, its number of base pairs and the total number of reads in the transcriptome [78]. This normalization can eliminate the influence of different gene lengths and sequencing levels on the calculation of the gene expression. Therefore, the calculated gene expression can be directly used for comparison of the differences in gene expression between tissues in pairwise comparisons. The chosen method for evaluating the d.e.g. between tissues was NOISeq (http://bioinfo.cipf.es/noiseq) [50]. NOISeq is a nonparametric statistical approach that creates an empirical distribution of count changes that are adapted to the available data. This method has been proven to be the most effective in controlling the false discovery rate. The *p*-value threshold used to detect d.e.g. was 0.01.

To present the quantitative results and to facilitate their visualization, the pairwise comparisons (three per tissue) were fused, calculating the average of the three fold change values of the transcripts with the same annotation. Only one table/figure per tissue is presented, rather than all the possible comparisons.

The heatmap shown in Fig. 6 was designed with the software TMeV [79]. The normalized values (RPKM) for each gene by tissue and biological replicate were used to represent their expression in a green/0 – red/100 scale, with green representing the lower expression values and red the higher expression values.

#### GO classification and enrichment analysis

The nr annotation was used to obtain the GO term assignments of the transcripts with the Blast2GO program [80]. Then, enrichment analyses were conducted with the total information from all the tissues, including the reference set and each tissue and expression analysis test set. Next, Fisher’s exact test was run with default values (a two-tailed test that removes double IDs, with a false discovery rate (FDR) cut-off of 0.01). The Blast2GO option to show only the most specific terms (0.01 FDR cut-off) was used once. To reduce the dimensions of Fig. 8, the enrichment analyses of the expression results were combined according to the tissue types. Thus, only one graph per tissue is represented, instead of all the possible comparisons. Non-redundant categories were aggregated. For the coincident categories, the average of the percent representation was calculated.

## Additional files

Additional file 1:
**Table listing Mytilus galloprovincialis transcripts, including the sequence, length, RPKM, description, accession number of the description (Hit ACC) and e-value obtained in each database used for annotation and the GO terms ascribed to each sequence.** (XLSX 38412 kb) Additional file 2:
**Figure representing the number of transcripts obtained after the assembly of increasing number of mapped reads.** (XLSX 743 kb) Additional file 3:
**Pathways found in the annotated portion of the transcriptomes.** (ZIP 4950 kb) Additional file 4:
**Tables and figures showing the non-shared C1q variants found in the hemocytes, mantle, muscle and gills.** (XLSX 17 kb) Additional file 5:
**Mytilus galloprovincialis transcriptome in FASTA format.** (ZIP 25884 kb)

## Abbreviations

ACC Nº: Accession number
ADAMTS16: A disintegrin and metalloproteinase with thrombospondin motifs 16
AMPs: Antimicrobial peptides
BLAST: Basic local alignment search tool
bp: Base pairs
C1q: C1q domain-containing proteins
CEGMA: Core Eukaryotic Genes Mapping Approach
COG: Clusters of orthologous groups of proteins
d.e.g.: Differentially expressed genes
EST: Expressed sequence tags
FC: Fold change
FDR: False discovery rate
FREPs: Fibrinogen-related proteins
GO: Gene Ontology
GrpE: GroP-like gene E
HSP: Heat shock protein
IAP: Inhibitor of apoptosis protein
ID: Identity
IFI27: Interferon alpha-inducible protein 27-like protein
ISG12: Interferon-stimulated gene 12 protein
KEGG: Kyoto encyclopedia of genes and genomes
LPS: Lipopolysaccharide
LTA: Lipoteichoic acid
*M. galloprovincialis*: *Mytilus galloprovincialis*
MAC: Membrane attack complex
MARCO: Macrophage receptor with collagenous structure
MRC: Mitochondria-rich cell
NCBI: National Center for Biotechnology Information
NGS: Next generation sequencing
NO: Nitric oxide
PG: Peptidoglycans
Poly I:C: Polyinosinic:polycytidylic acid
RNA-Seq: RNA sequencing
RPKM: Reads Per Kilobase of exon model per Million mapped reads
SRA: Short read archive
ST14: Suppressor of tumorigenicity 14 protein
TLL1: Tolloid like-1 precursor
TMeV: TIGR Multiexperiment Viewer
TRIM56: Tripartite motif-containing protein 56
*V. anguillarum*: *Vibrio anguillarum*
