# Supplementary figures and images for: RNA-Seq in Mytilus galloprovincialis: comparative transcriptomics and expression profiles among different tissues (part 2 of 2)
Source: BMC Genomics. 2015 Sep 24;16(1):728. doi: 10.1186/s12864-015-1817-5 (PMC4581086; doi:10.1186/s12864-015-1817-5)

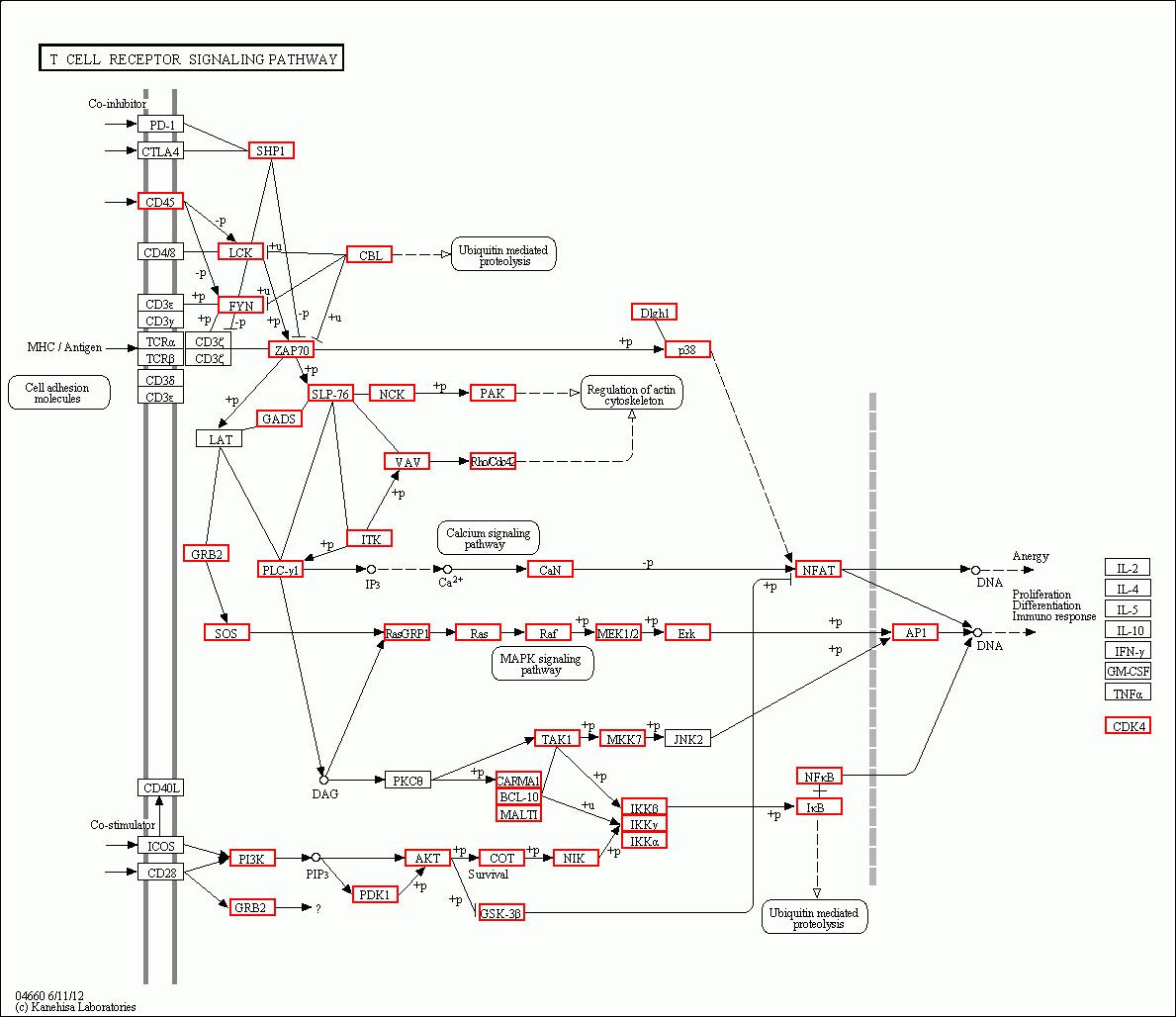

Supplement: Additional file 3: — Pathways found in the annotated portion of the transcriptomes. (ZIP 4950 kb) [file 12864_2015_1817_MOESM3_ESM.zip › map04660.png]

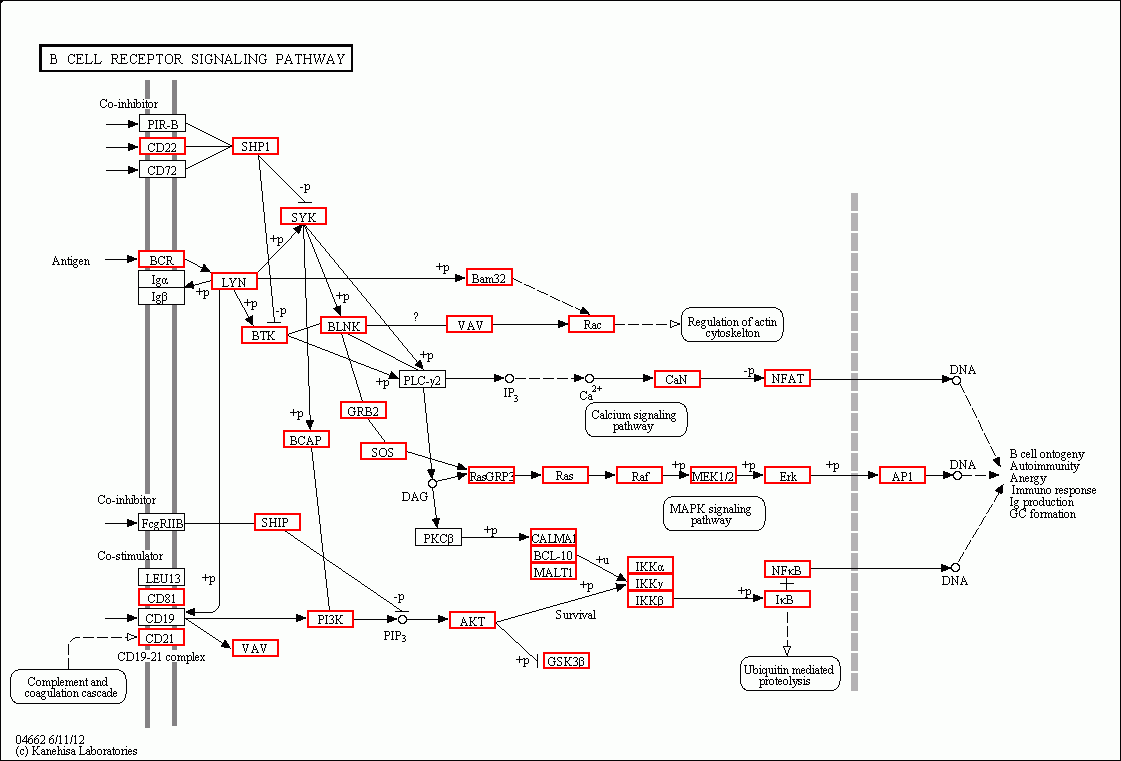

Supplement: Additional file 3: — Pathways found in the annotated portion of the transcriptomes. (ZIP 4950 kb) [file 12864_2015_1817_MOESM3_ESM.zip › map04662.png]

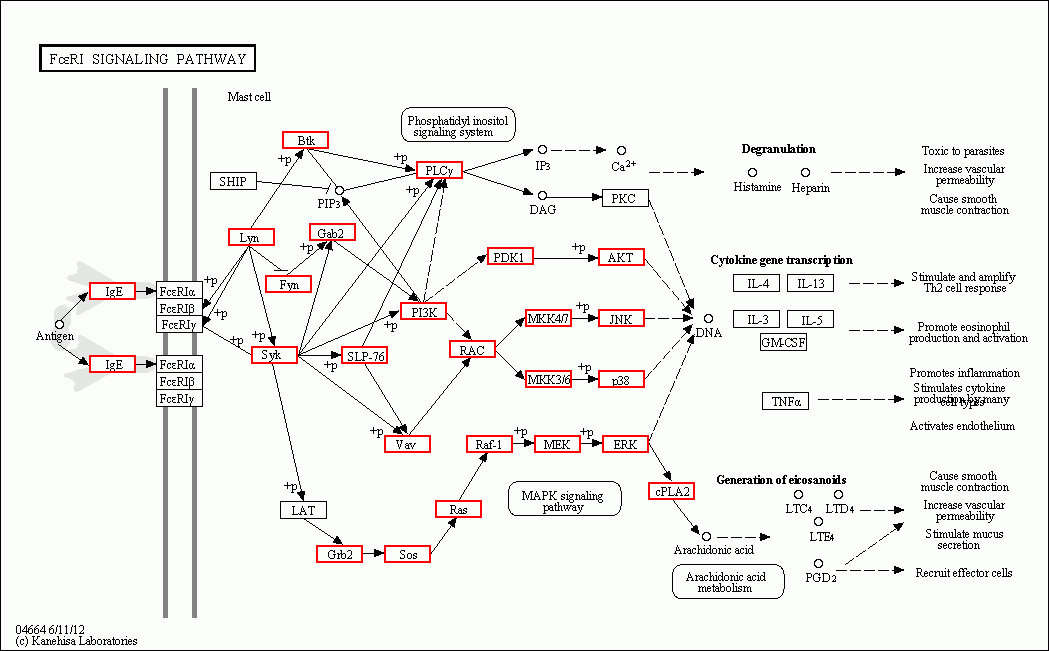

Supplement: Additional file 3: — Pathways found in the annotated portion of the transcriptomes. (ZIP 4950 kb) [file 12864_2015_1817_MOESM3_ESM.zip › map04664.png]

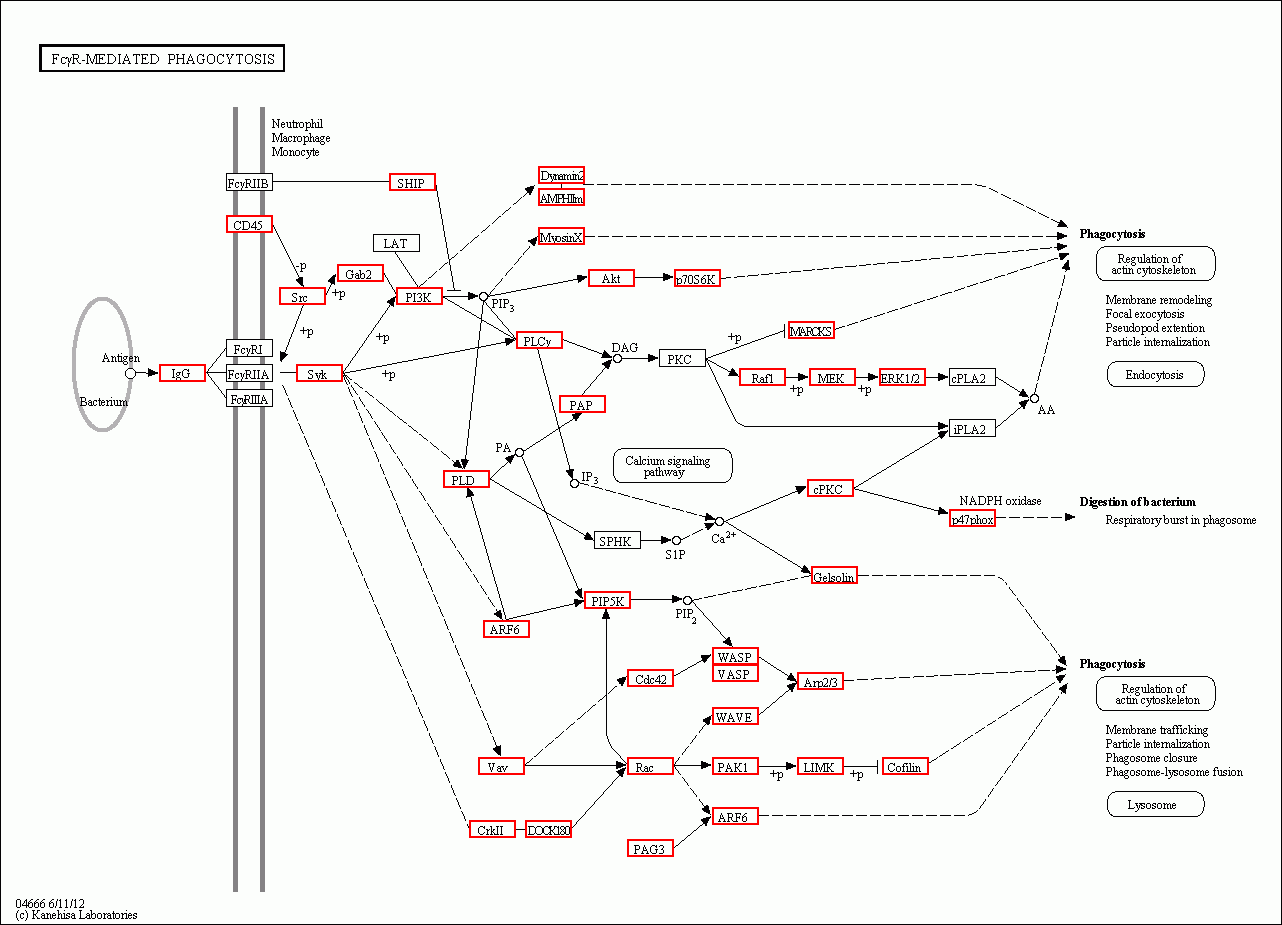

Supplement: Additional file 3: — Pathways found in the annotated portion of the transcriptomes. (ZIP 4950 kb) [file 12864_2015_1817_MOESM3_ESM.zip › map04666.png]

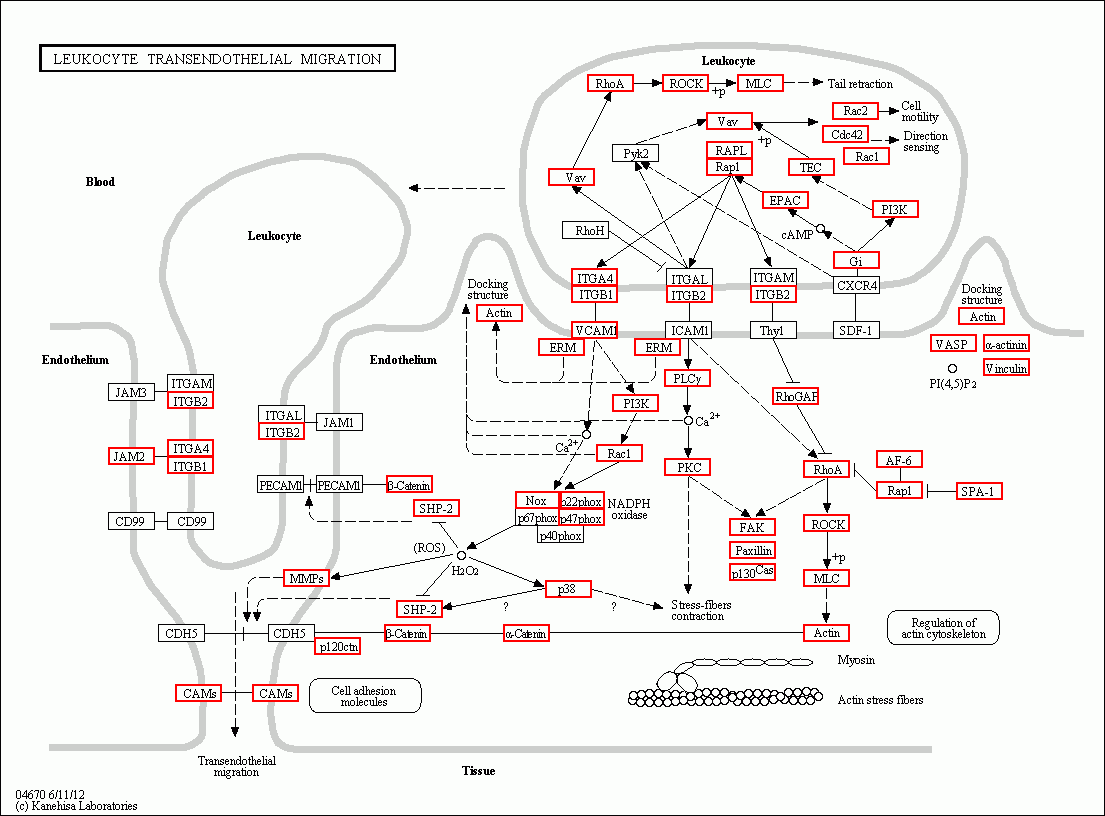

Supplement: Additional file 3: — Pathways found in the annotated portion of the transcriptomes. (ZIP 4950 kb) [file 12864_2015_1817_MOESM3_ESM.zip › map04670.png]

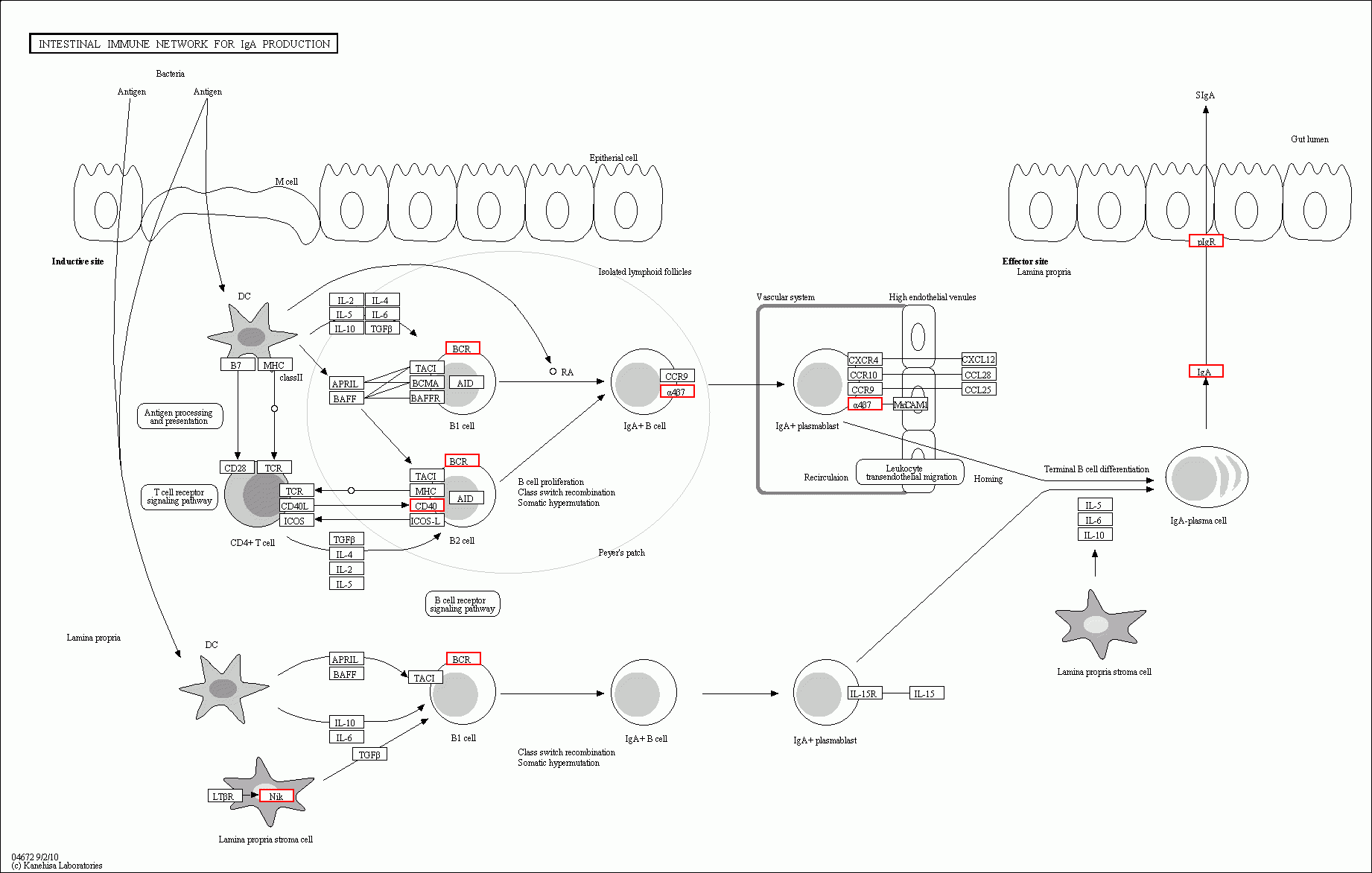

Supplement: Additional file 3: — Pathways found in the annotated portion of the transcriptomes. (ZIP 4950 kb) [file 12864_2015_1817_MOESM3_ESM.zip › map04672.png]

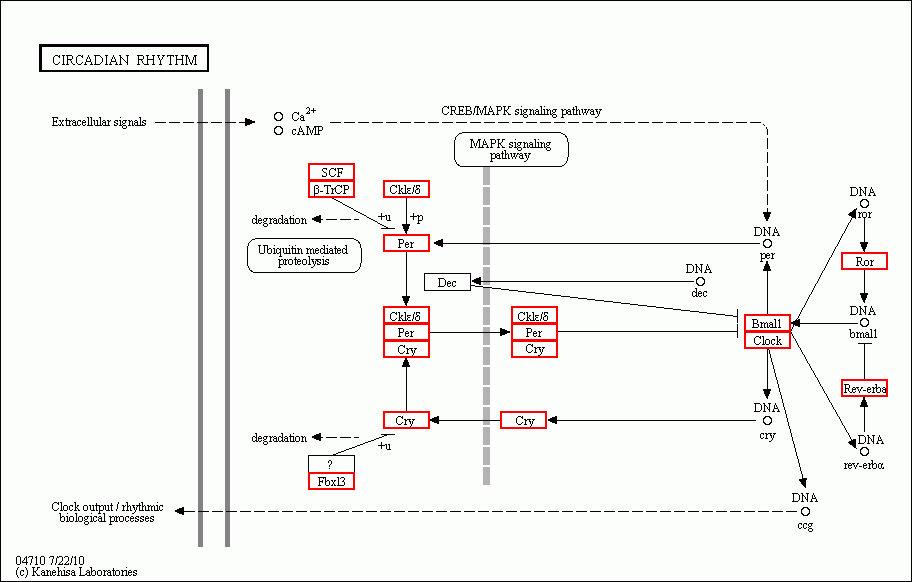

Supplement: Additional file 3: — Pathways found in the annotated portion of the transcriptomes. (ZIP 4950 kb) [file 12864_2015_1817_MOESM3_ESM.zip › map04710.png]

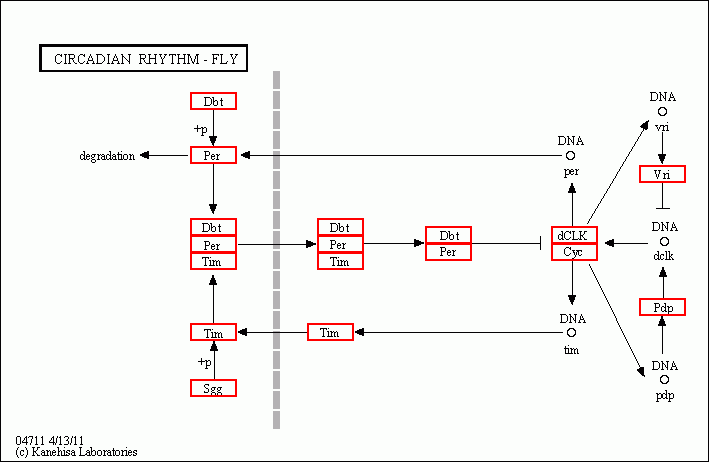

Supplement: Additional file 3: — Pathways found in the annotated portion of the transcriptomes. (ZIP 4950 kb) [file 12864_2015_1817_MOESM3_ESM.zip › map04711.png]

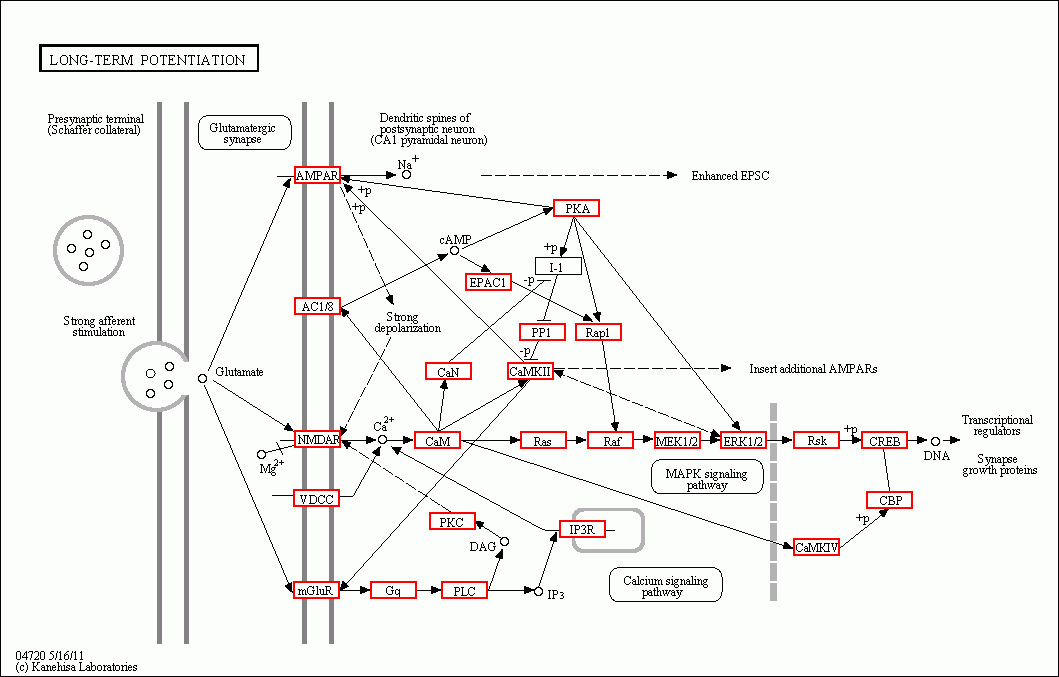

Supplement: Additional file 3: — Pathways found in the annotated portion of the transcriptomes. (ZIP 4950 kb) [file 12864_2015_1817_MOESM3_ESM.zip › map04720.png]

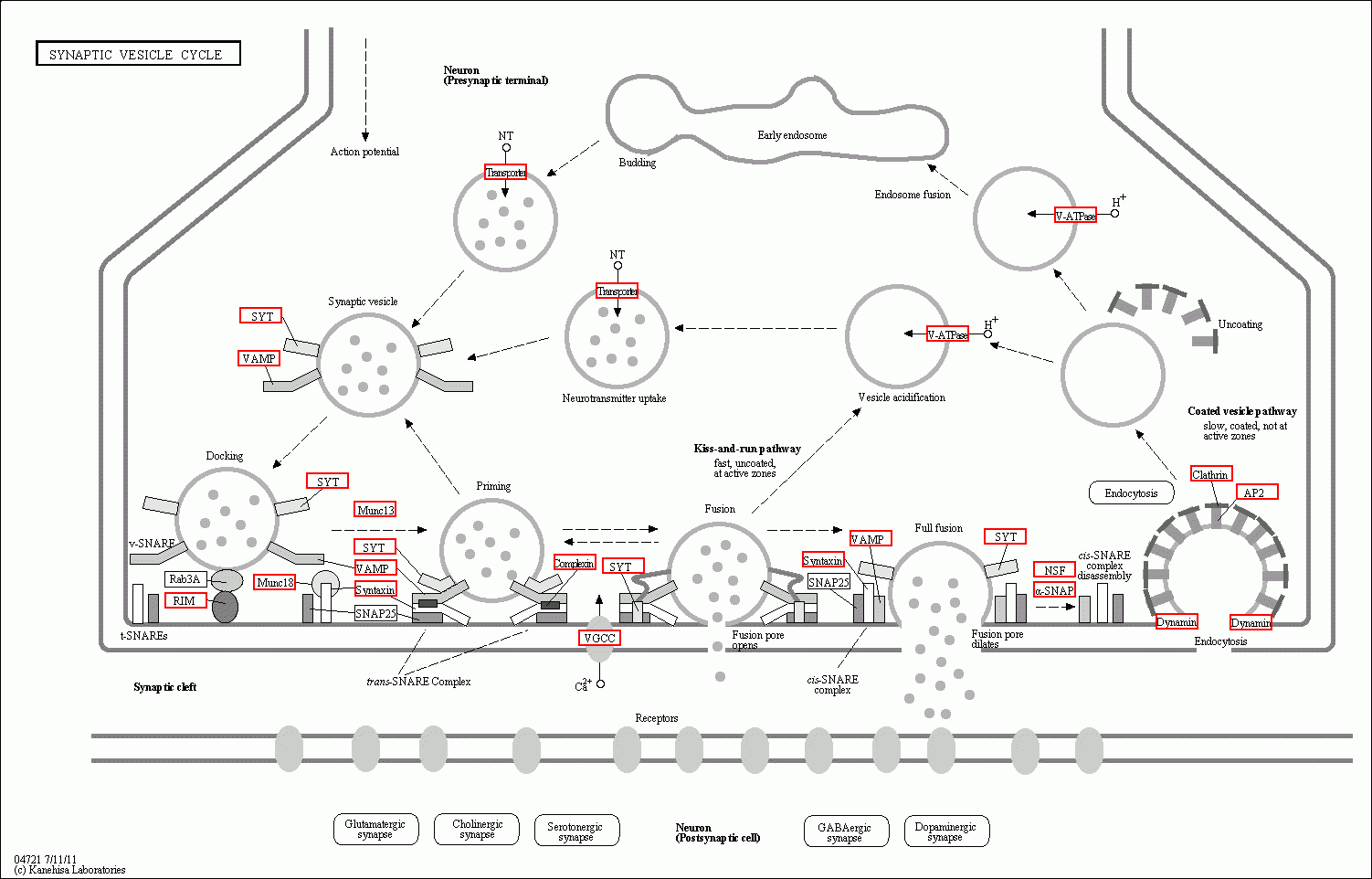

Supplement: Additional file 3: — Pathways found in the annotated portion of the transcriptomes. (ZIP 4950 kb) [file 12864_2015_1817_MOESM3_ESM.zip › map04721.png]

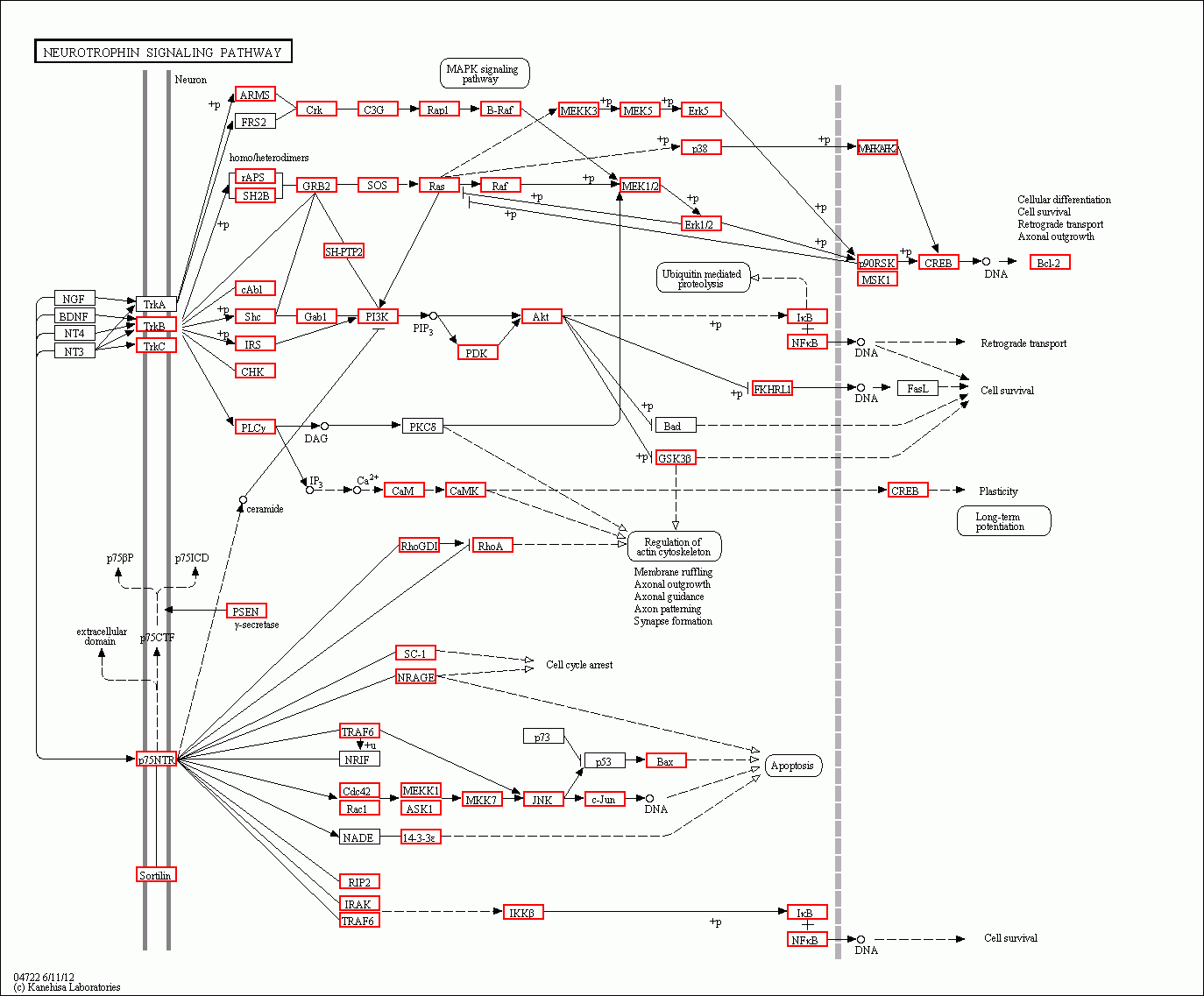

Supplement: Additional file 3: — Pathways found in the annotated portion of the transcriptomes. (ZIP 4950 kb) [file 12864_2015_1817_MOESM3_ESM.zip › map04722.png]

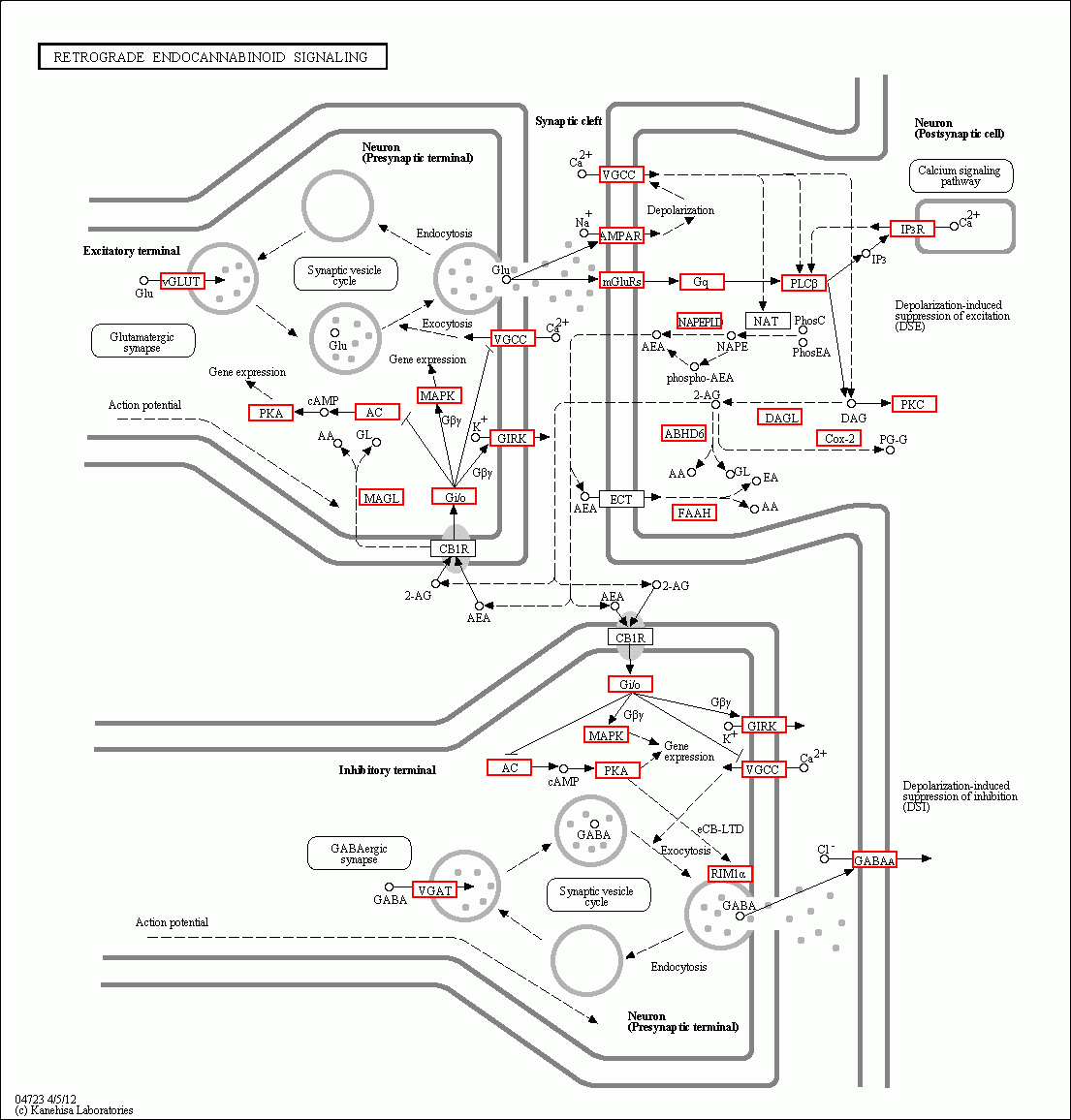

Supplement: Additional file 3: — Pathways found in the annotated portion of the transcriptomes. (ZIP 4950 kb) [file 12864_2015_1817_MOESM3_ESM.zip › map04723.png]

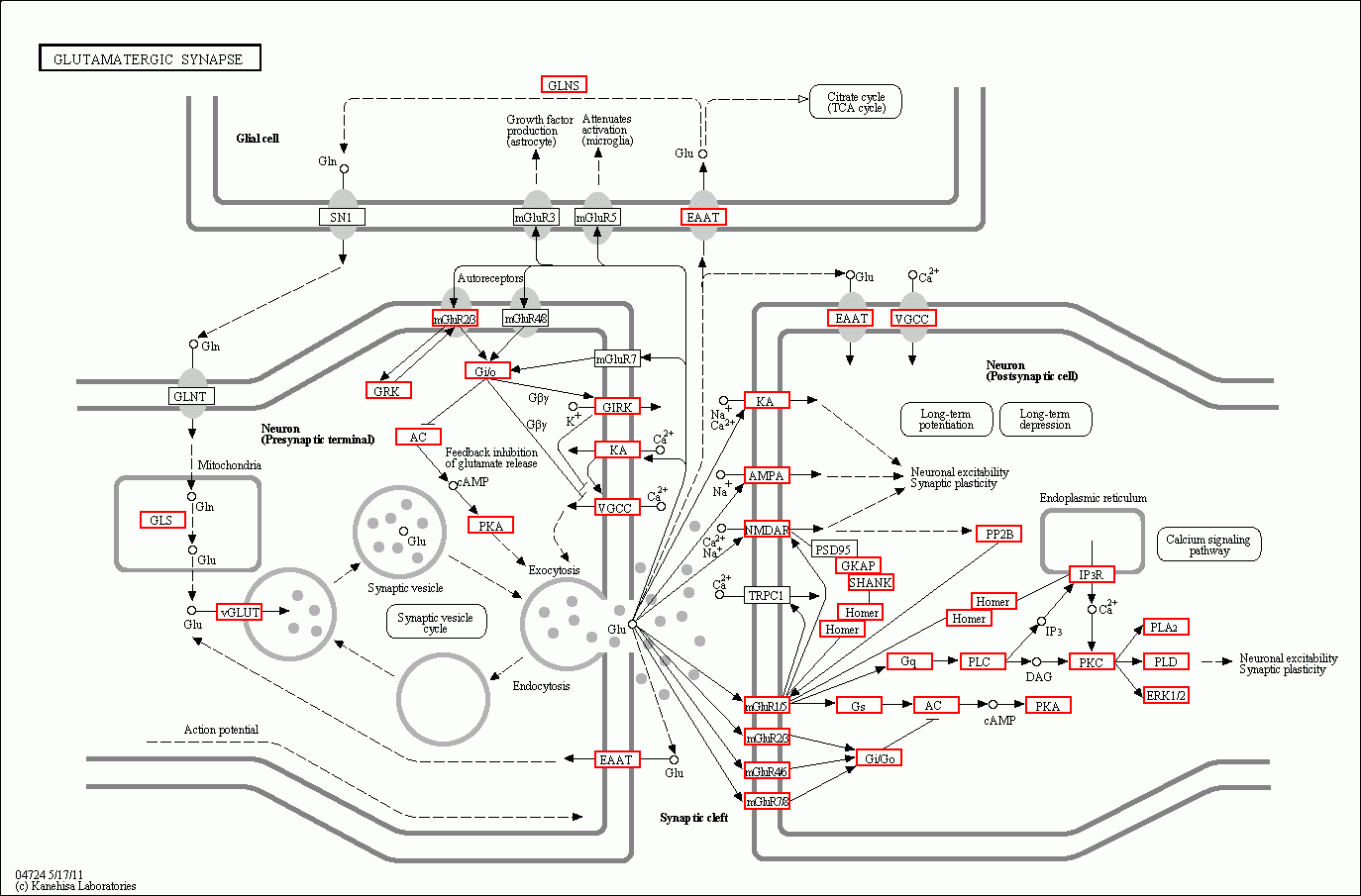

Supplement: Additional file 3: — Pathways found in the annotated portion of the transcriptomes. (ZIP 4950 kb) [file 12864_2015_1817_MOESM3_ESM.zip › map04724.png]

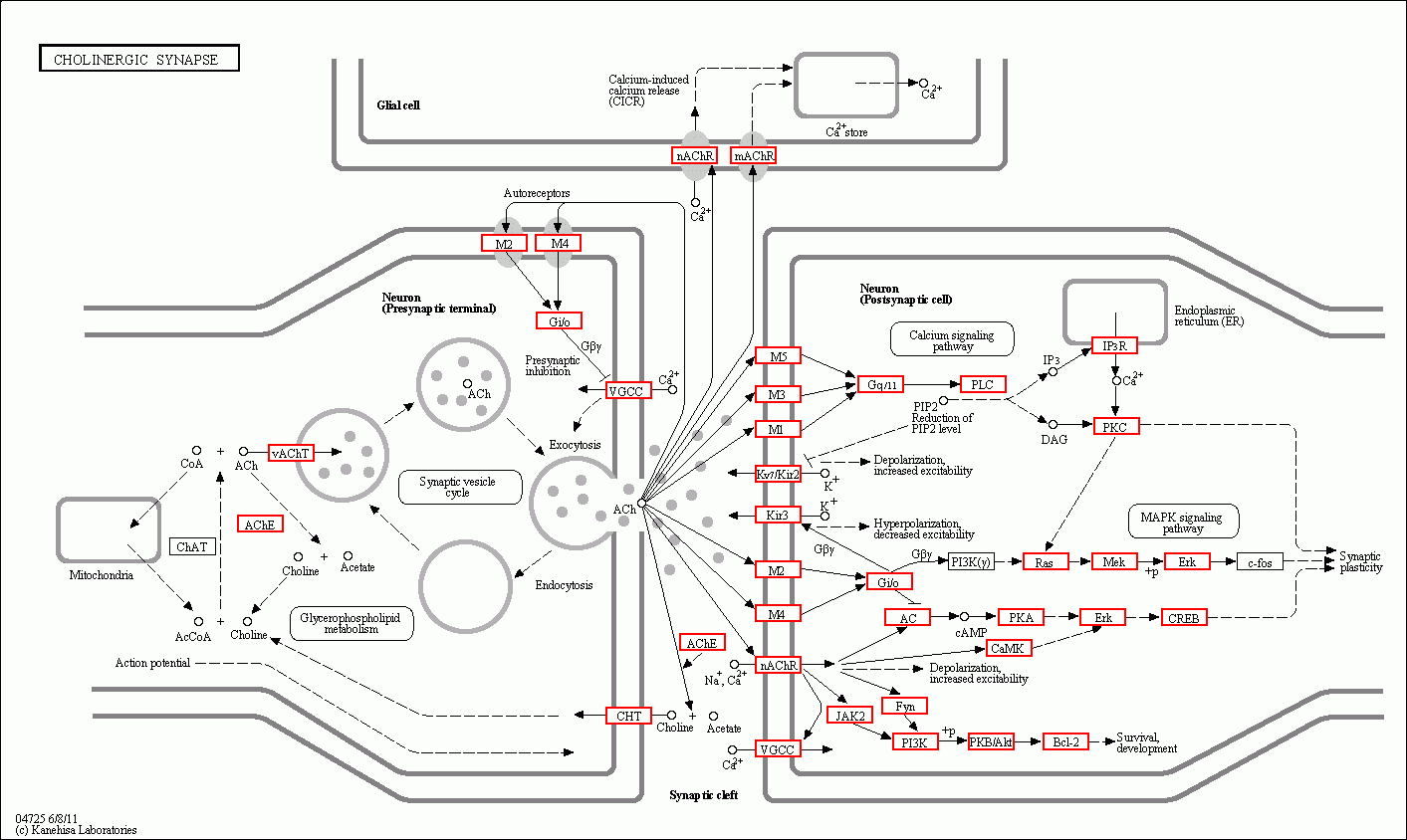

Supplement: Additional file 3: — Pathways found in the annotated portion of the transcriptomes. (ZIP 4950 kb) [file 12864_2015_1817_MOESM3_ESM.zip › map04725.png]

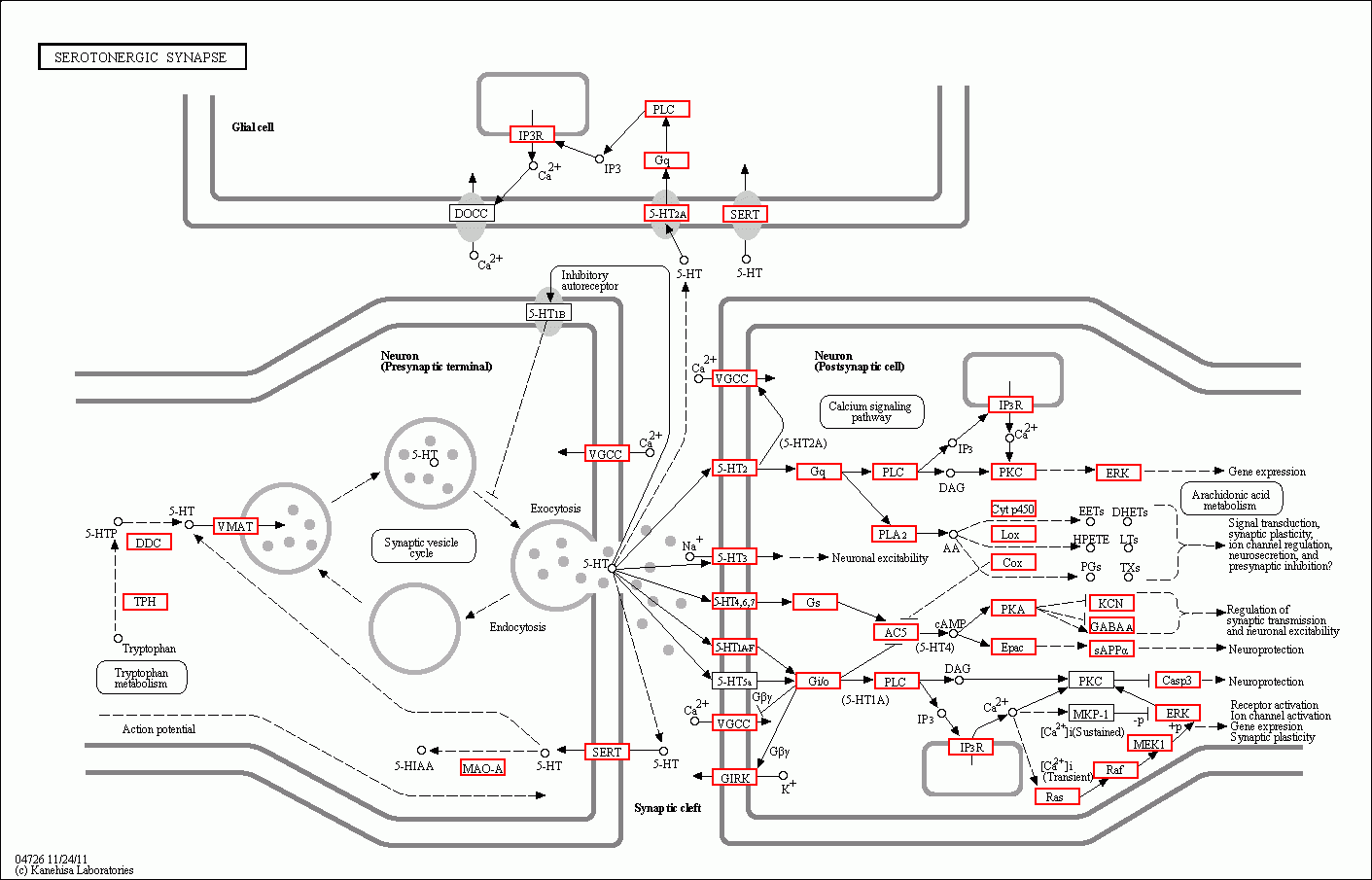

Supplement: Additional file 3: — Pathways found in the annotated portion of the transcriptomes. (ZIP 4950 kb) [file 12864_2015_1817_MOESM3_ESM.zip › map04726.png]

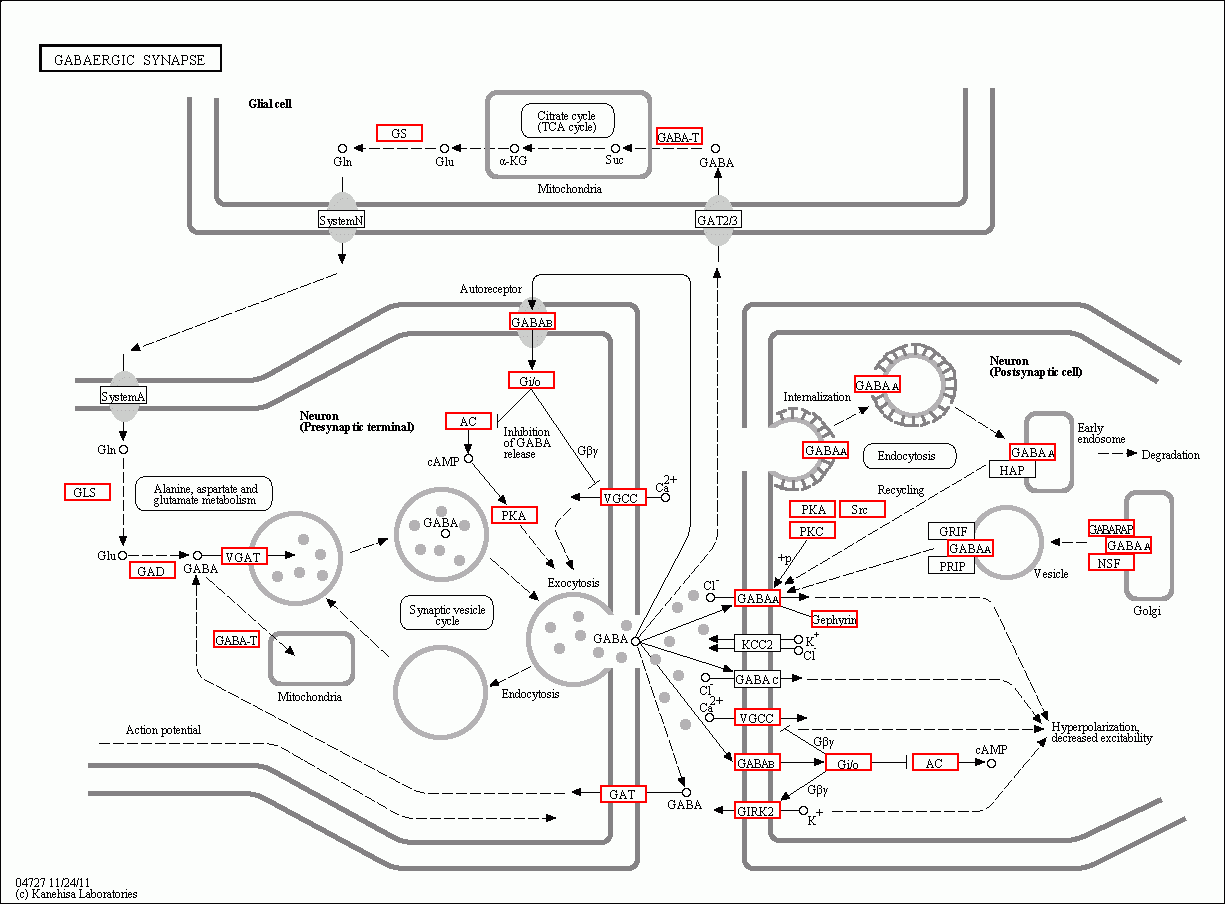

Supplement: Additional file 3: — Pathways found in the annotated portion of the transcriptomes. (ZIP 4950 kb) [file 12864_2015_1817_MOESM3_ESM.zip › map04727.png]

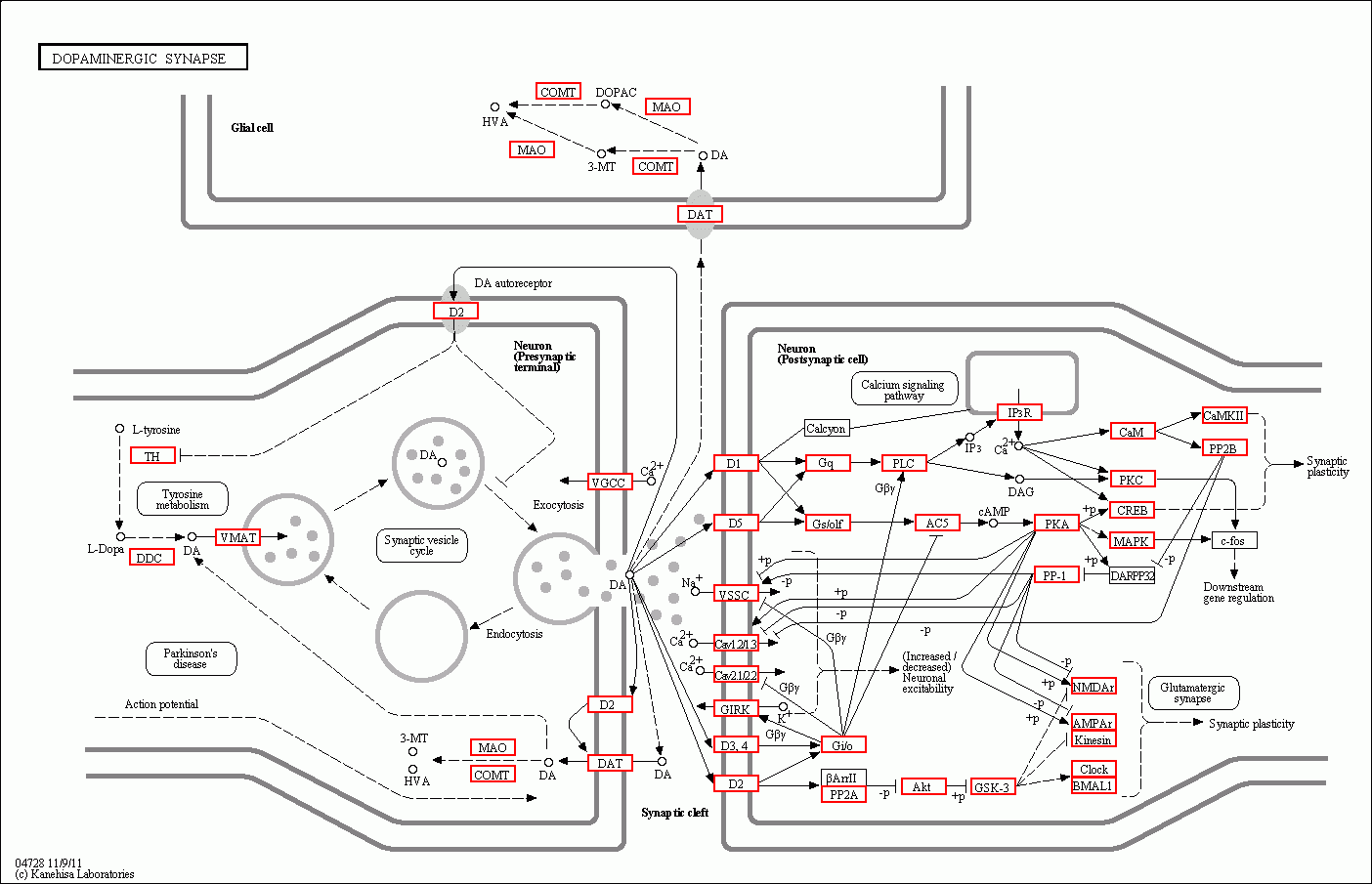

Supplement: Additional file 3: — Pathways found in the annotated portion of the transcriptomes. (ZIP 4950 kb) [file 12864_2015_1817_MOESM3_ESM.zip › map04728.png]

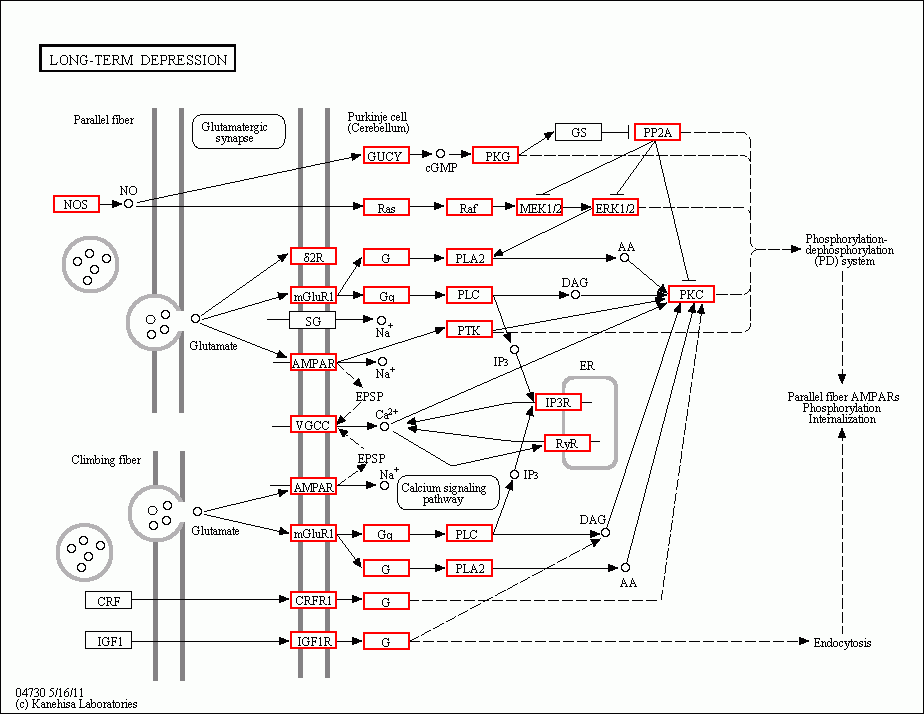

Supplement: Additional file 3: — Pathways found in the annotated portion of the transcriptomes. (ZIP 4950 kb) [file 12864_2015_1817_MOESM3_ESM.zip › map04730.png]

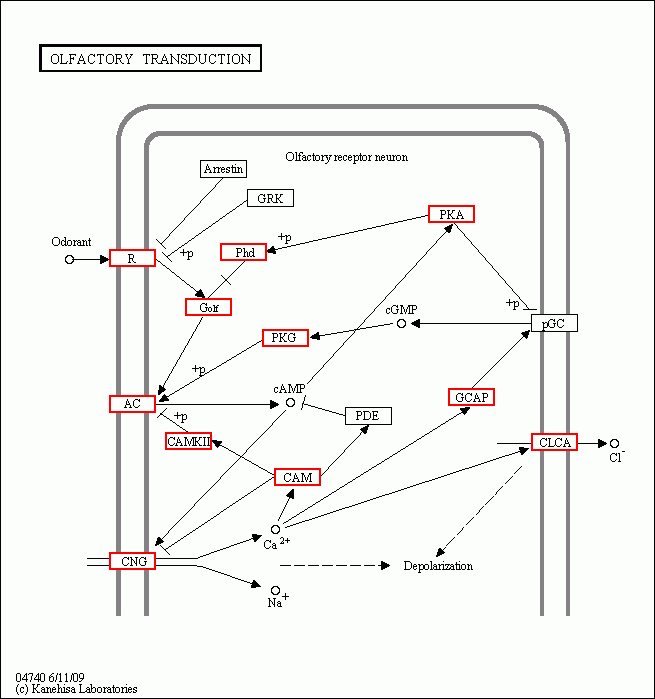

Supplement: Additional file 3: — Pathways found in the annotated portion of the transcriptomes. (ZIP 4950 kb) [file 12864_2015_1817_MOESM3_ESM.zip › map04740.png]

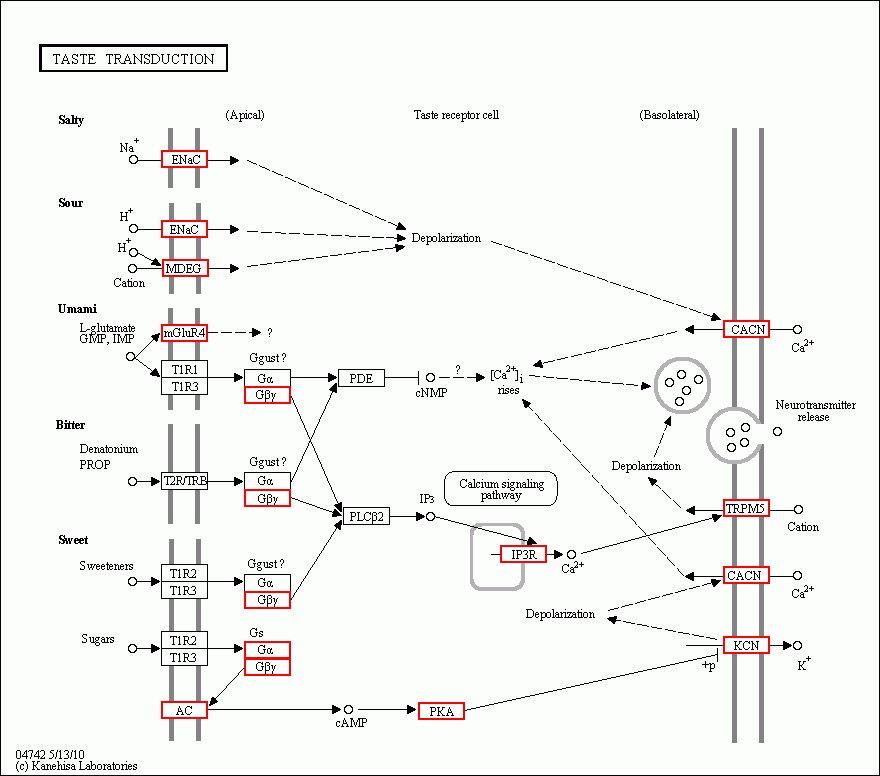

Supplement: Additional file 3: — Pathways found in the annotated portion of the transcriptomes. (ZIP 4950 kb) [file 12864_2015_1817_MOESM3_ESM.zip › map04742.png]

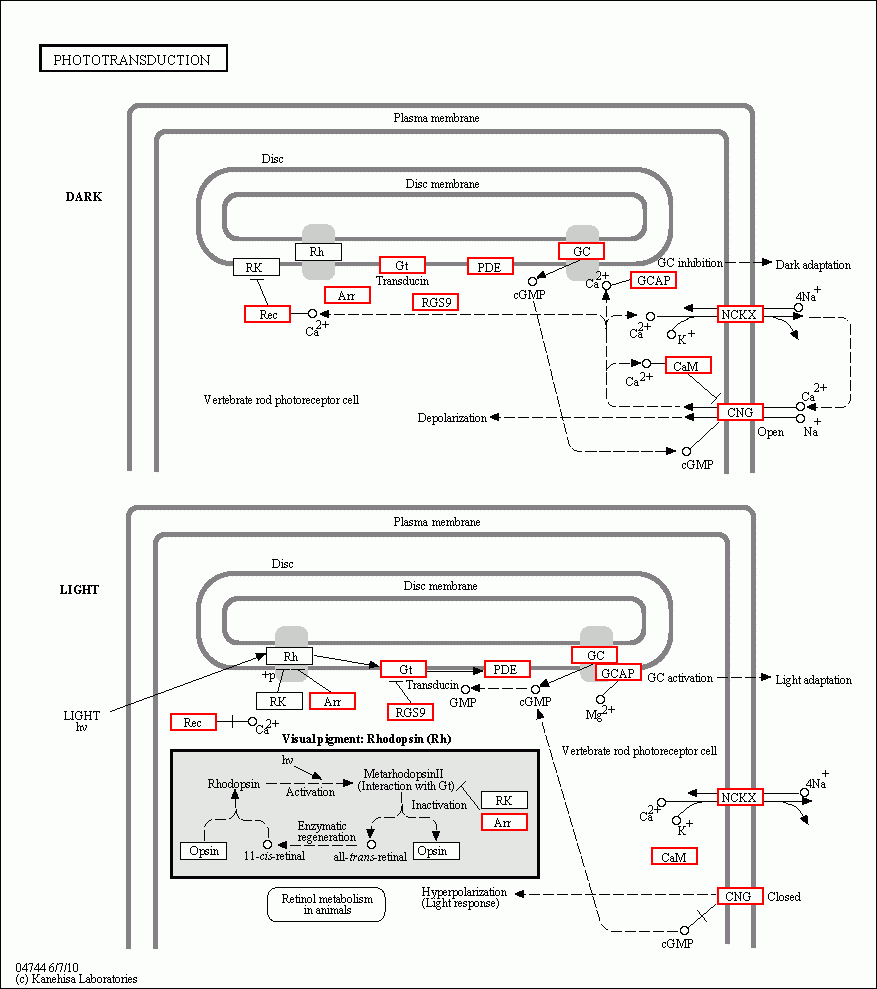

Supplement: Additional file 3: — Pathways found in the annotated portion of the transcriptomes. (ZIP 4950 kb) [file 12864_2015_1817_MOESM3_ESM.zip › map04744.png]

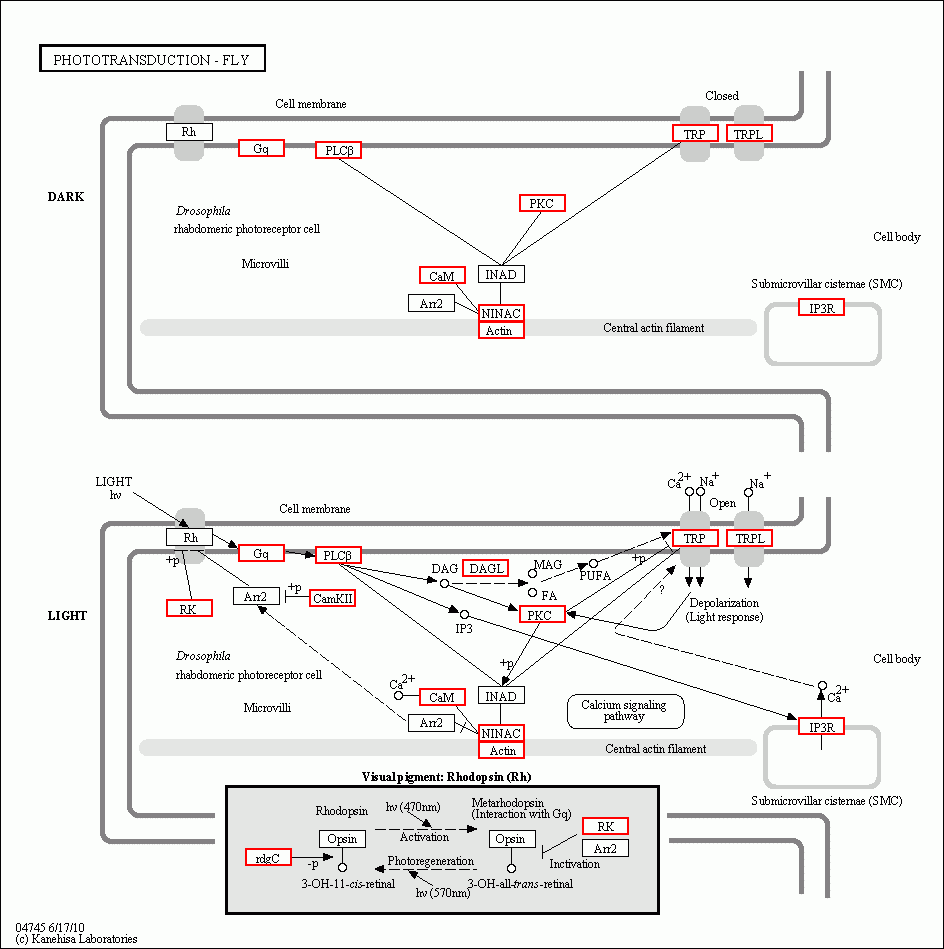

Supplement: Additional file 3: — Pathways found in the annotated portion of the transcriptomes. (ZIP 4950 kb) [file 12864_2015_1817_MOESM3_ESM.zip › map04745.png]

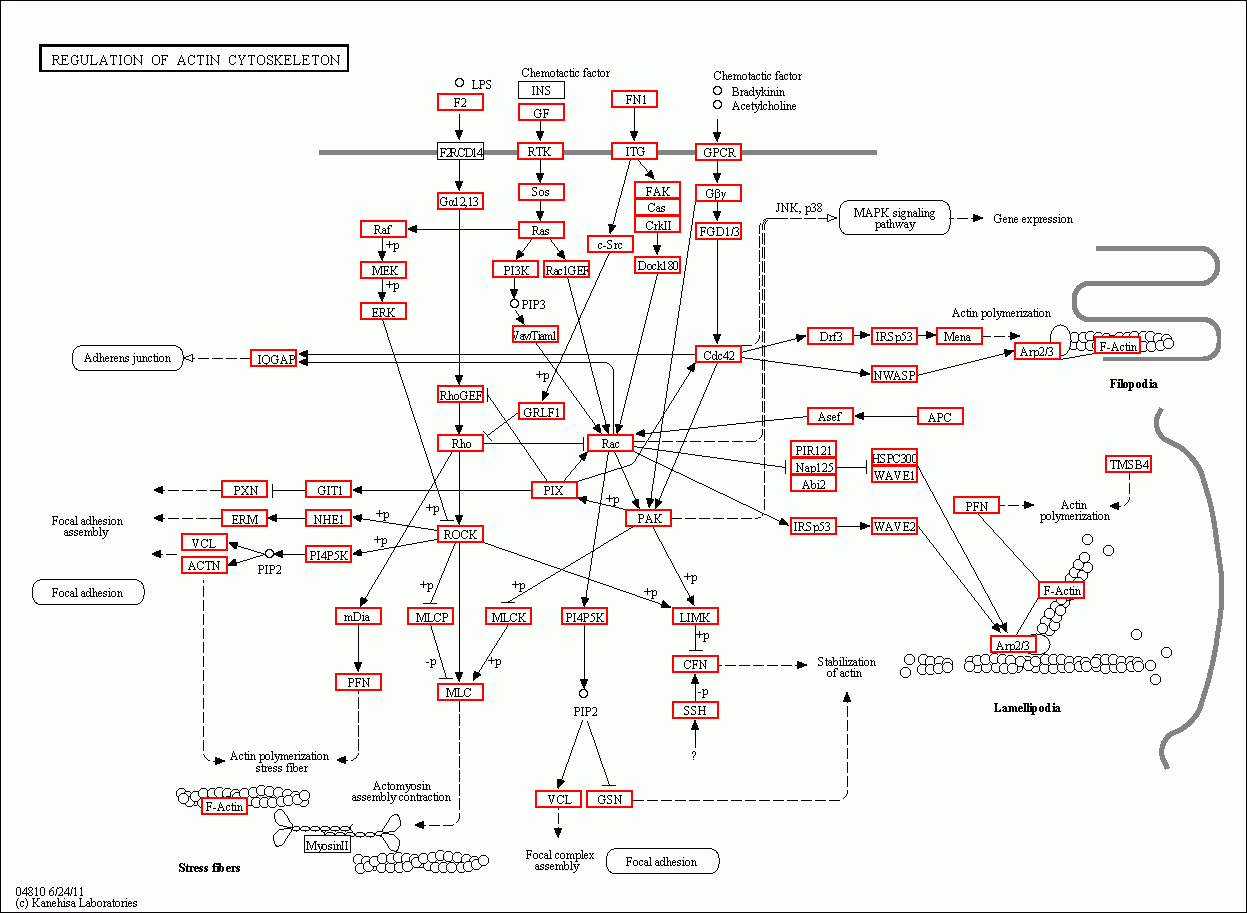

Supplement: Additional file 3: — Pathways found in the annotated portion of the transcriptomes. (ZIP 4950 kb) [file 12864_2015_1817_MOESM3_ESM.zip › map04810.png]

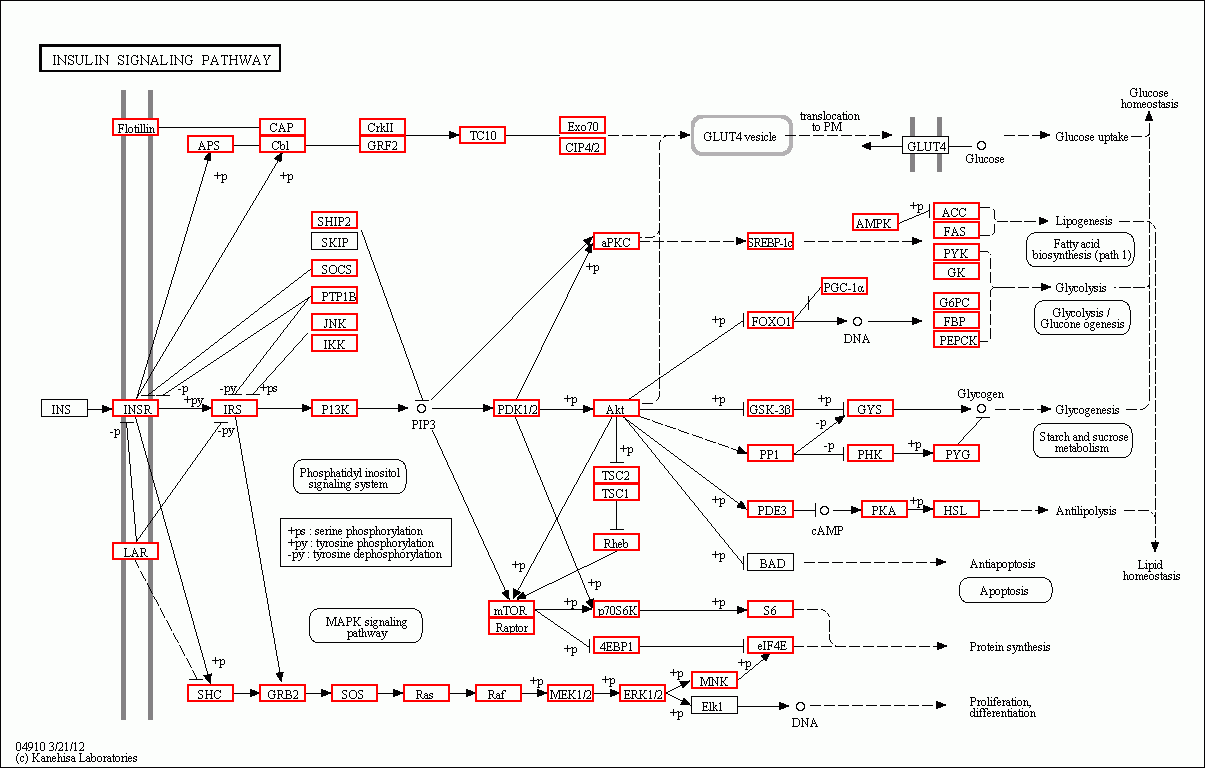

Supplement: Additional file 3: — Pathways found in the annotated portion of the transcriptomes. (ZIP 4950 kb) [file 12864_2015_1817_MOESM3_ESM.zip › map04910.png]

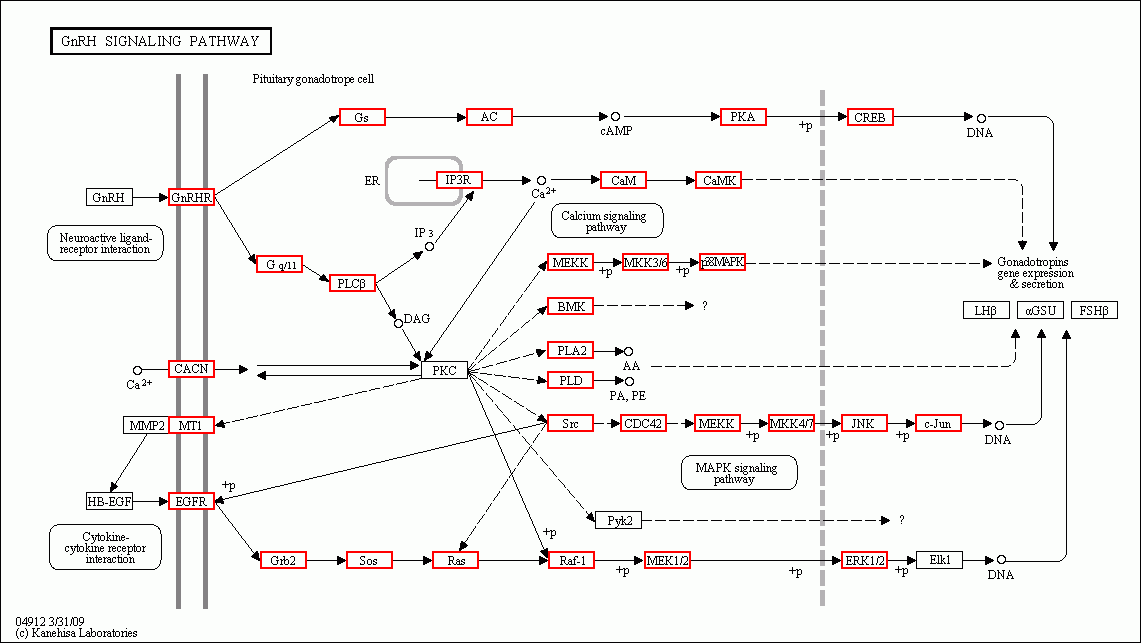

Supplement: Additional file 3: — Pathways found in the annotated portion of the transcriptomes. (ZIP 4950 kb) [file 12864_2015_1817_MOESM3_ESM.zip › map04912.png]

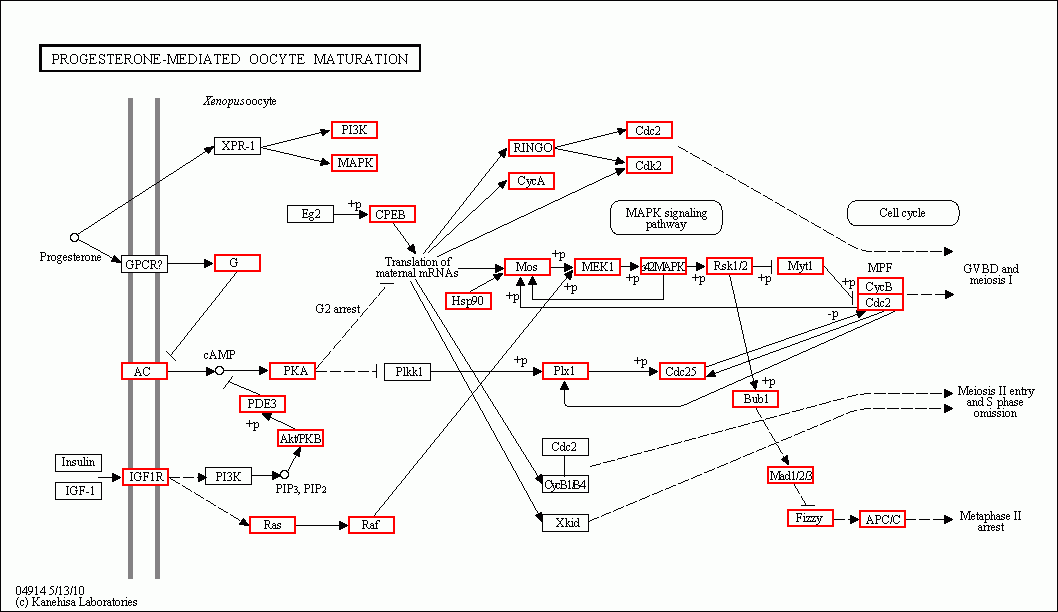

Supplement: Additional file 3: — Pathways found in the annotated portion of the transcriptomes. (ZIP 4950 kb) [file 12864_2015_1817_MOESM3_ESM.zip › map04914.png]

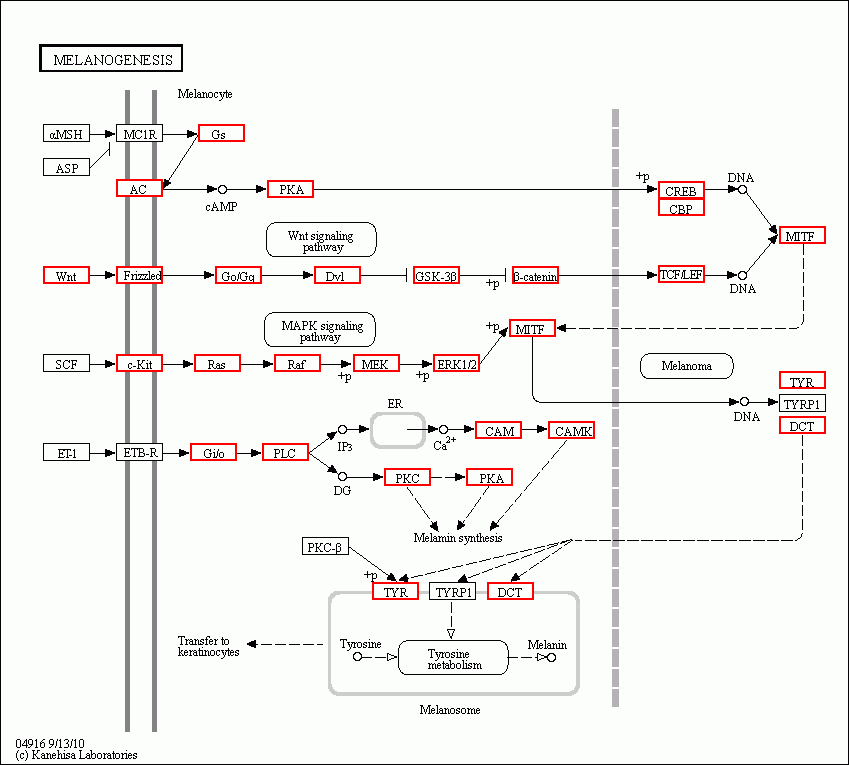

Supplement: Additional file 3: — Pathways found in the annotated portion of the transcriptomes. (ZIP 4950 kb) [file 12864_2015_1817_MOESM3_ESM.zip › map04916.png]

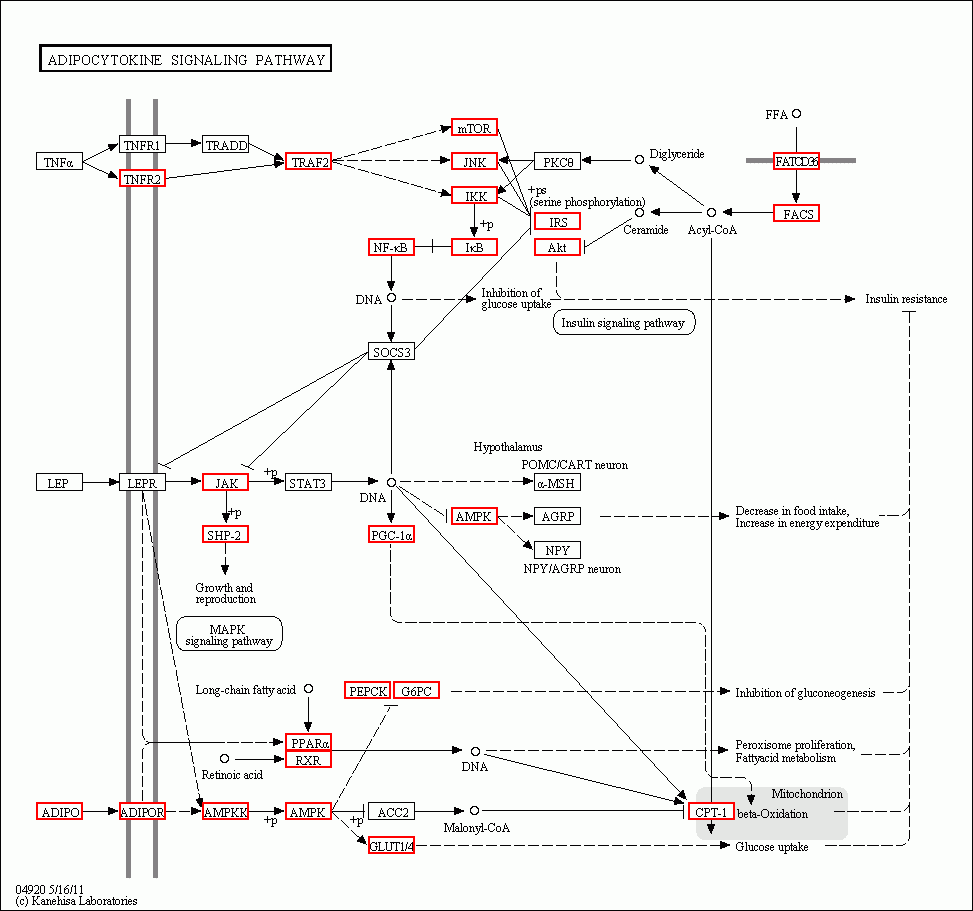

Supplement: Additional file 3: — Pathways found in the annotated portion of the transcriptomes. (ZIP 4950 kb) [file 12864_2015_1817_MOESM3_ESM.zip › map04920.png]

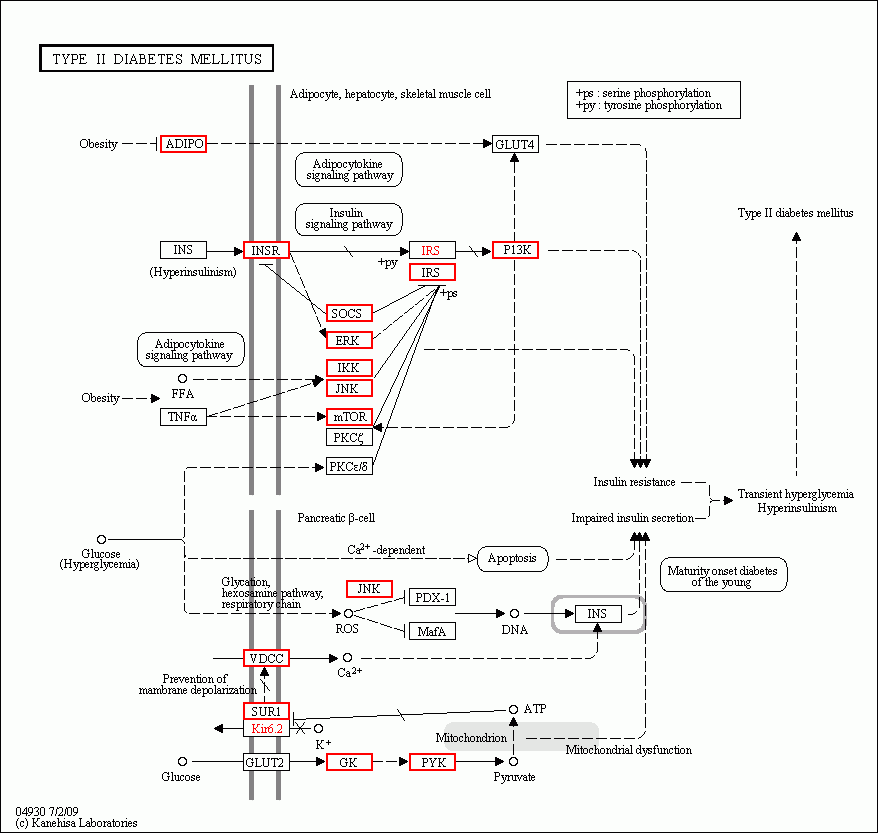

Supplement: Additional file 3: — Pathways found in the annotated portion of the transcriptomes. (ZIP 4950 kb) [file 12864_2015_1817_MOESM3_ESM.zip › map04930.png]

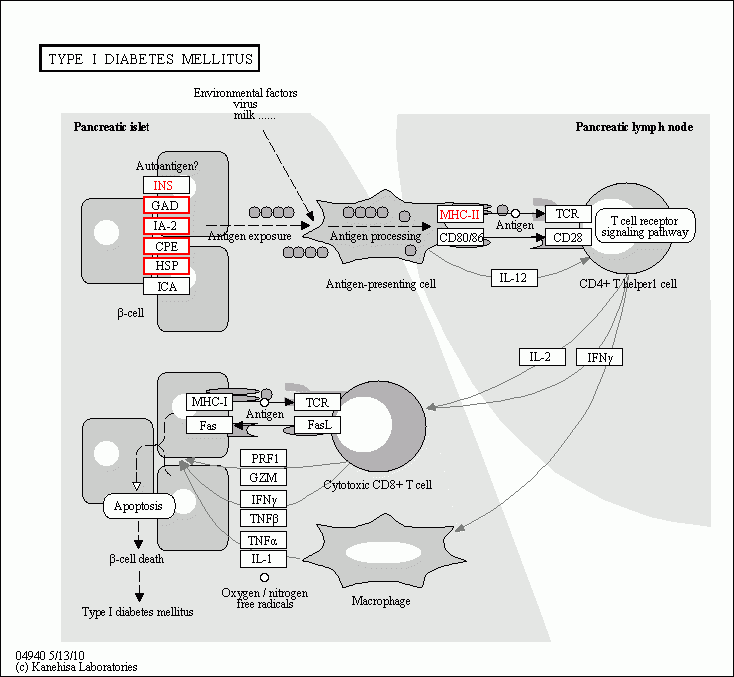

Supplement: Additional file 3: — Pathways found in the annotated portion of the transcriptomes. (ZIP 4950 kb) [file 12864_2015_1817_MOESM3_ESM.zip › map04940.png]

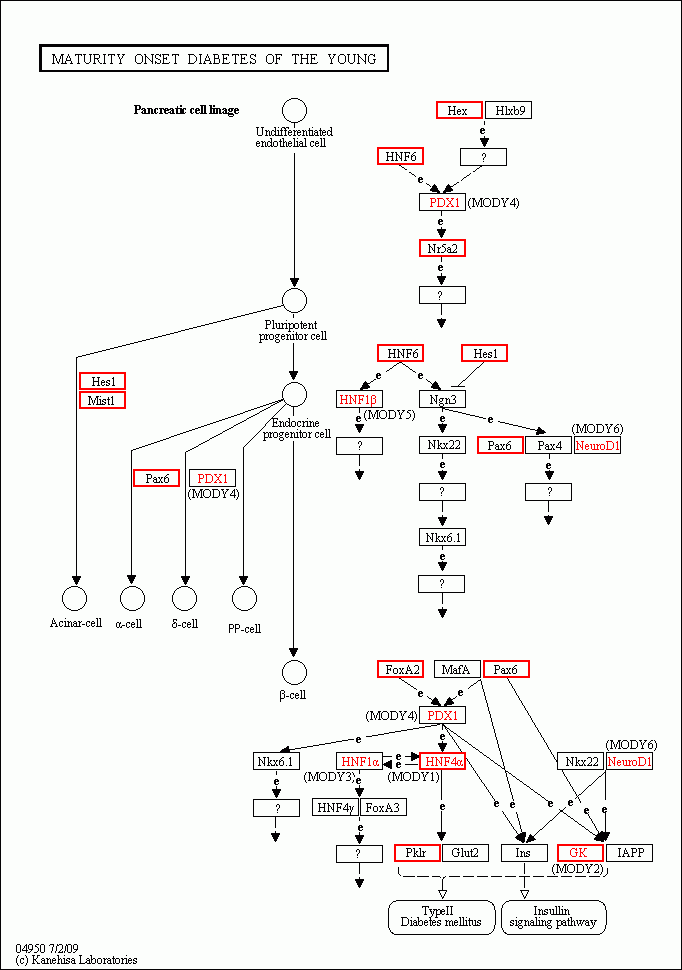

Supplement: Additional file 3: — Pathways found in the annotated portion of the transcriptomes. (ZIP 4950 kb) [file 12864_2015_1817_MOESM3_ESM.zip › map04950.png]

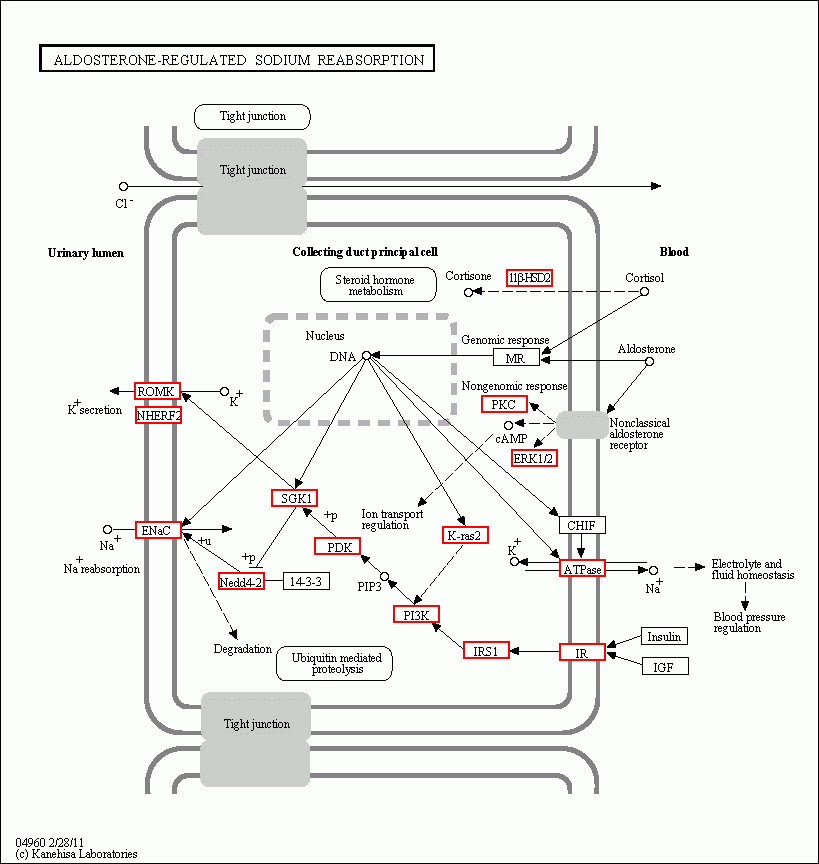

Supplement: Additional file 3: — Pathways found in the annotated portion of the transcriptomes. (ZIP 4950 kb) [file 12864_2015_1817_MOESM3_ESM.zip › map04960.png]

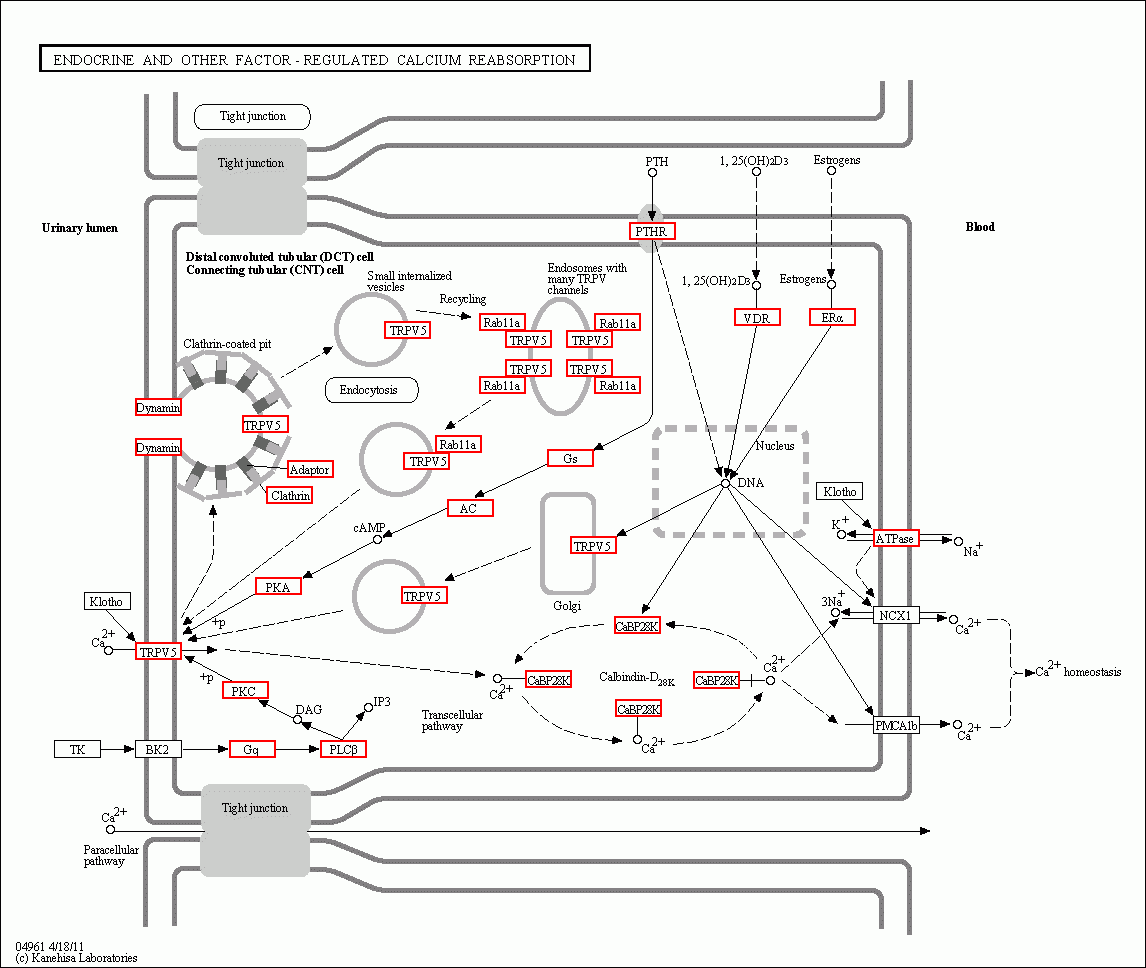

Supplement: Additional file 3: — Pathways found in the annotated portion of the transcriptomes. (ZIP 4950 kb) [file 12864_2015_1817_MOESM3_ESM.zip › map04961.png]

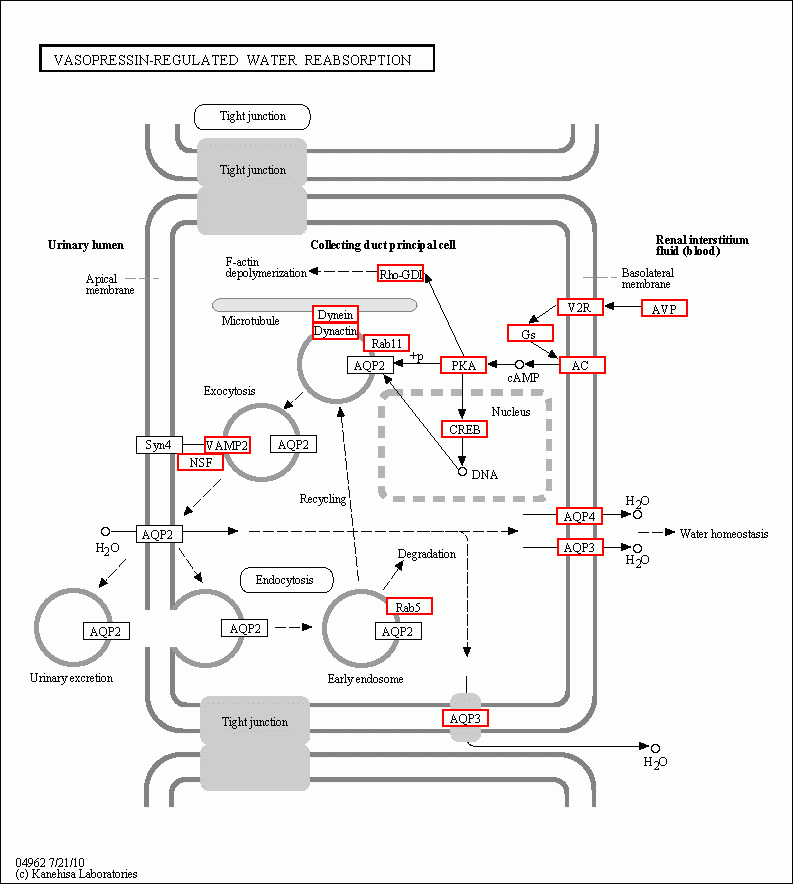

Supplement: Additional file 3: — Pathways found in the annotated portion of the transcriptomes. (ZIP 4950 kb) [file 12864_2015_1817_MOESM3_ESM.zip › map04962.png]

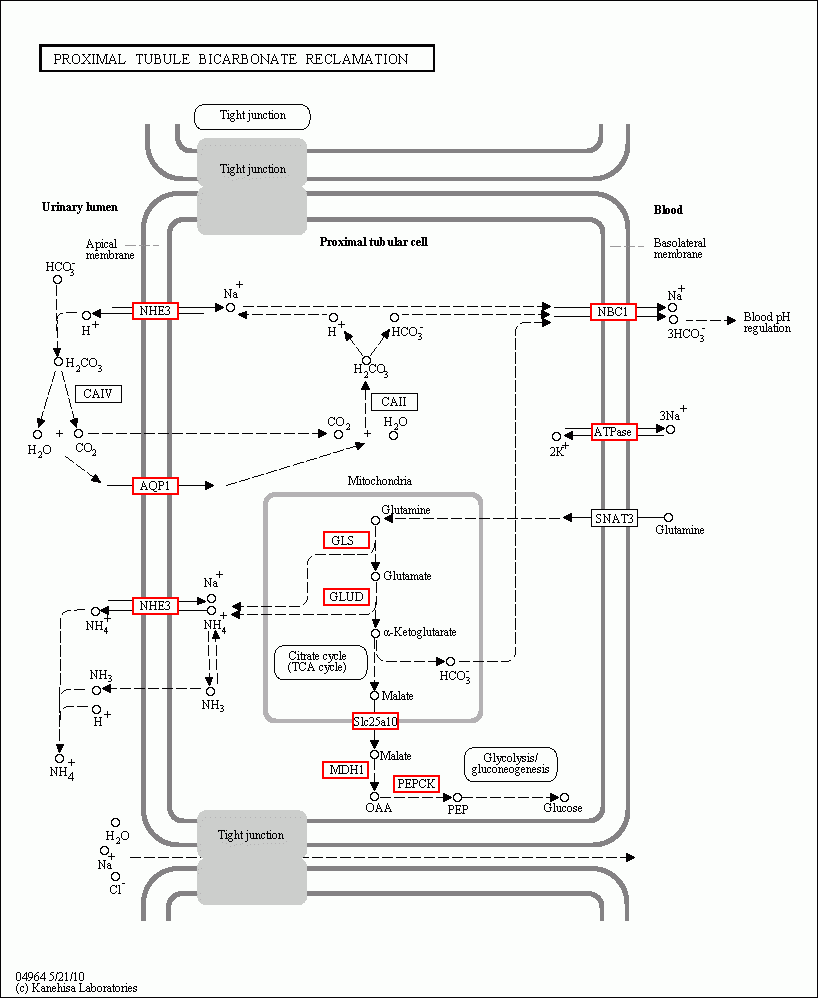

Supplement: Additional file 3: — Pathways found in the annotated portion of the transcriptomes. (ZIP 4950 kb) [file 12864_2015_1817_MOESM3_ESM.zip › map04964.png]

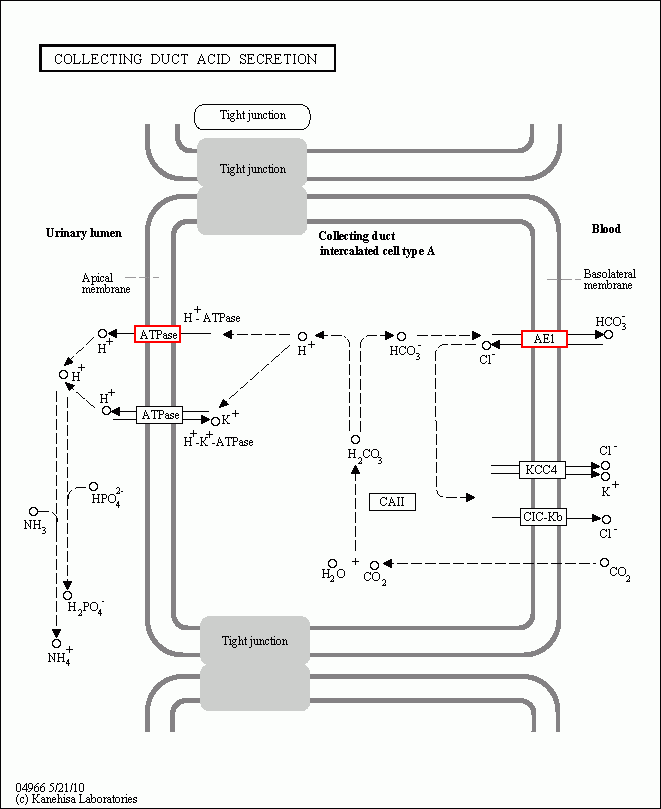

Supplement: Additional file 3: — Pathways found in the annotated portion of the transcriptomes. (ZIP 4950 kb) [file 12864_2015_1817_MOESM3_ESM.zip › map04966.png]

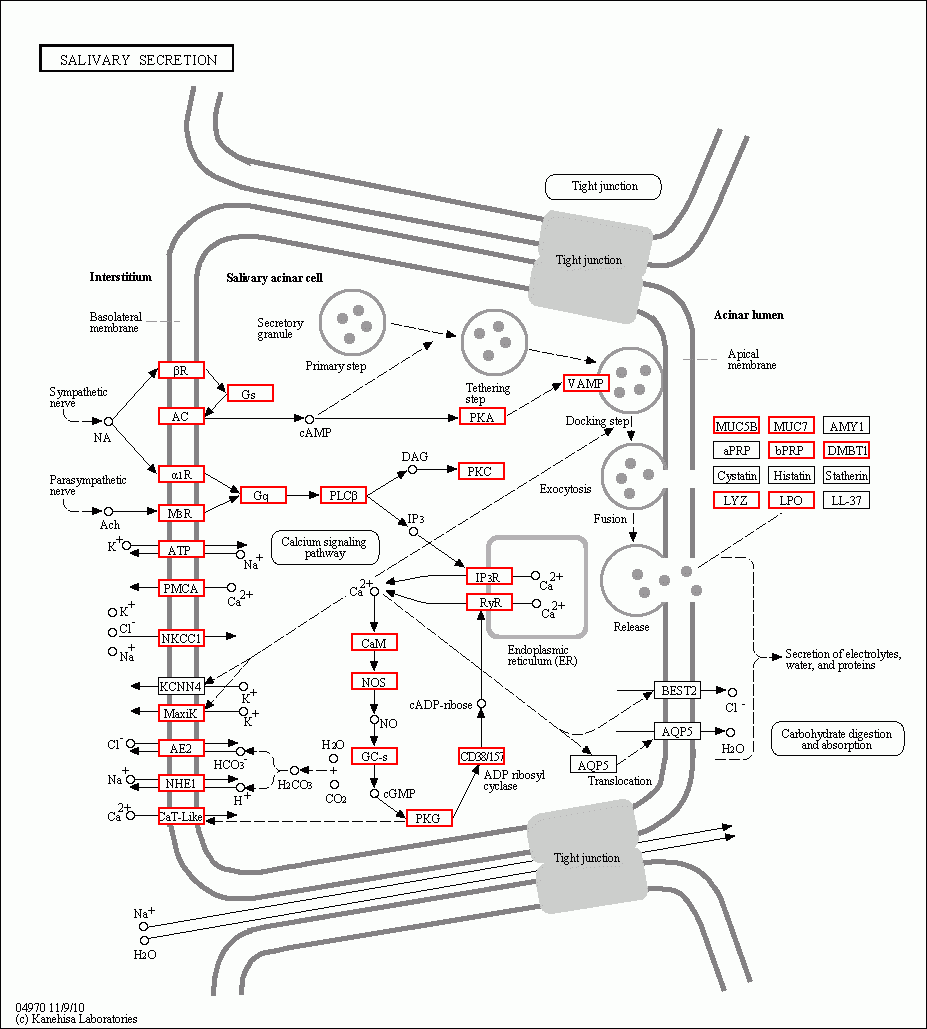

Supplement: Additional file 3: — Pathways found in the annotated portion of the transcriptomes. (ZIP 4950 kb) [file 12864_2015_1817_MOESM3_ESM.zip › map04970.png]

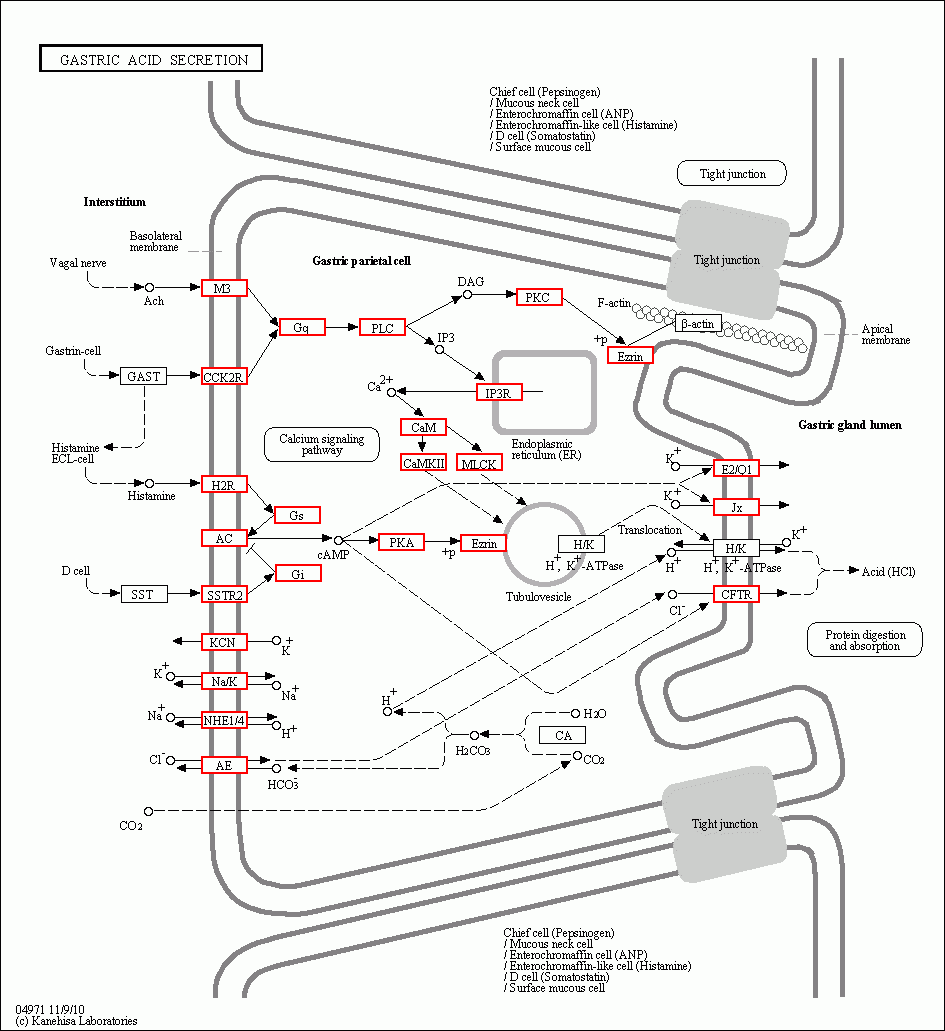

Supplement: Additional file 3: — Pathways found in the annotated portion of the transcriptomes. (ZIP 4950 kb) [file 12864_2015_1817_MOESM3_ESM.zip › map04971.png]

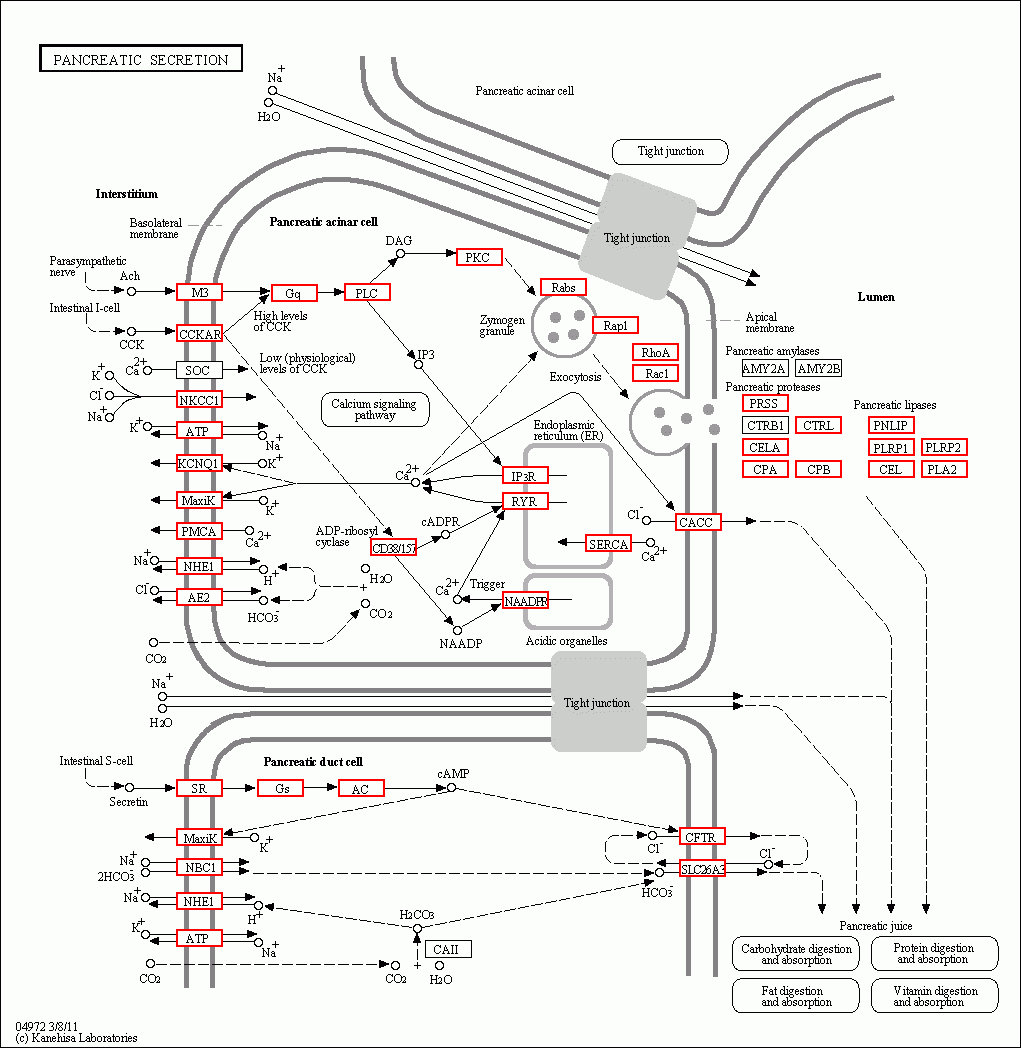

Supplement: Additional file 3: — Pathways found in the annotated portion of the transcriptomes. (ZIP 4950 kb) [file 12864_2015_1817_MOESM3_ESM.zip › map04972.png]

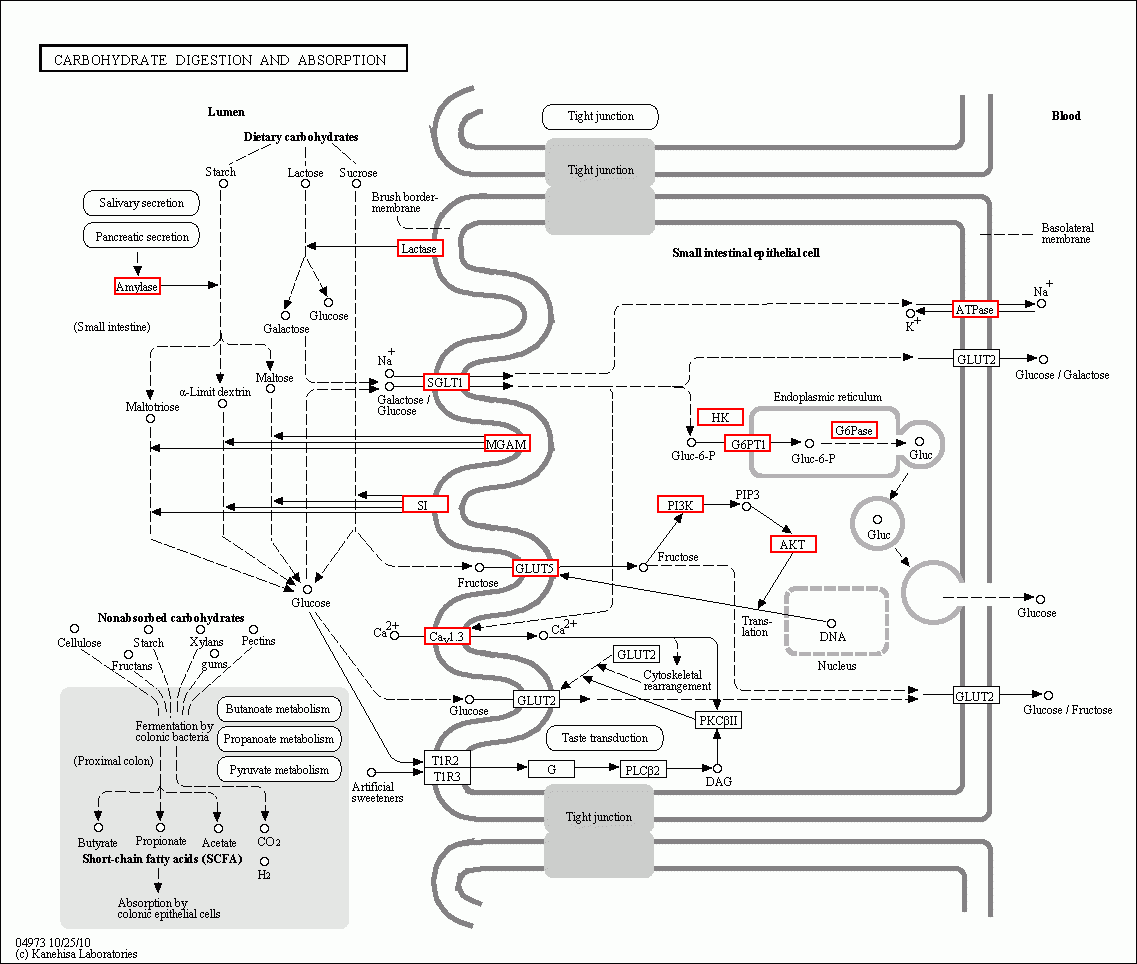

Supplement: Additional file 3: — Pathways found in the annotated portion of the transcriptomes. (ZIP 4950 kb) [file 12864_2015_1817_MOESM3_ESM.zip › map04973.png]

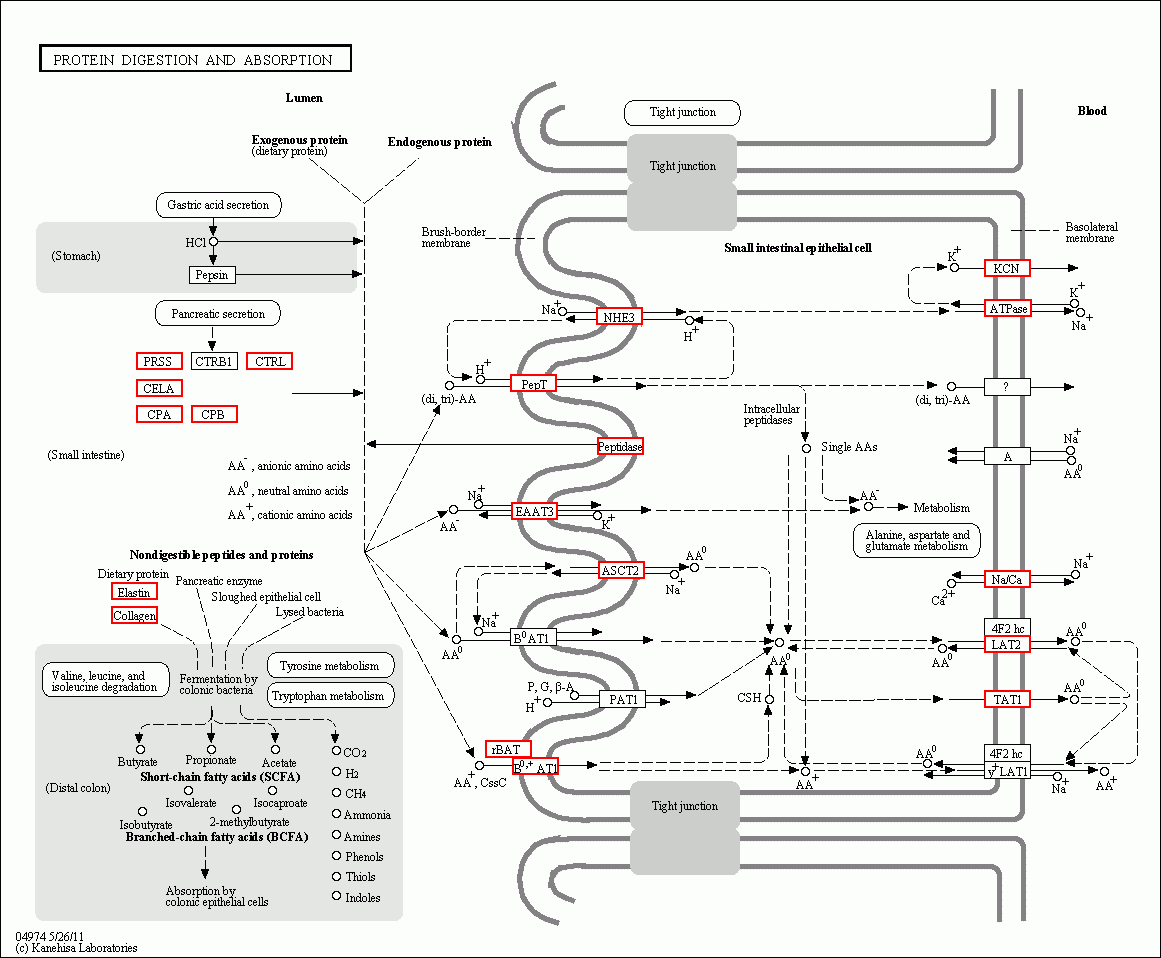

Supplement: Additional file 3: — Pathways found in the annotated portion of the transcriptomes. (ZIP 4950 kb) [file 12864_2015_1817_MOESM3_ESM.zip › map04974.png]

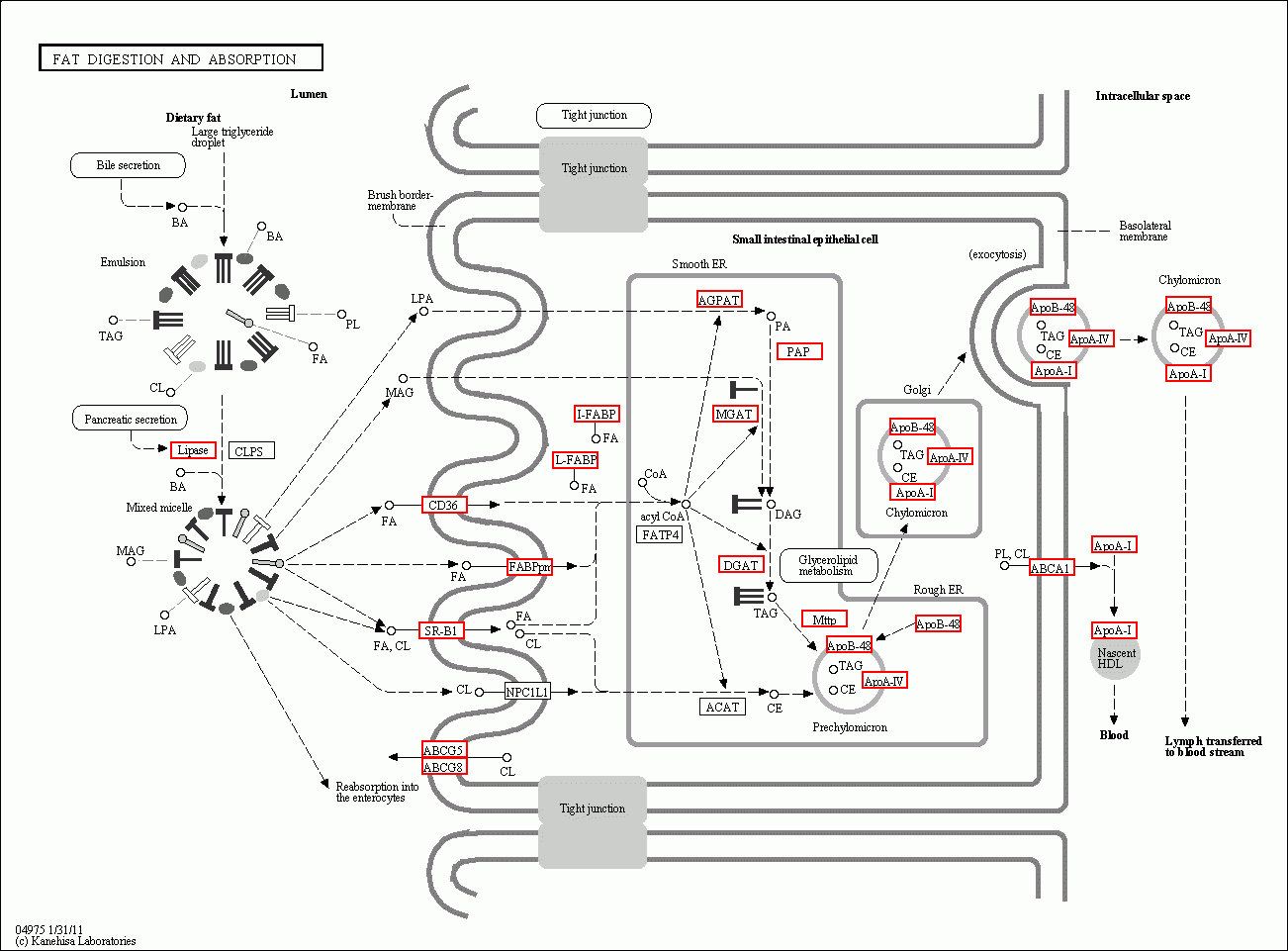

Supplement: Additional file 3: — Pathways found in the annotated portion of the transcriptomes. (ZIP 4950 kb) [file 12864_2015_1817_MOESM3_ESM.zip › map04975.png]

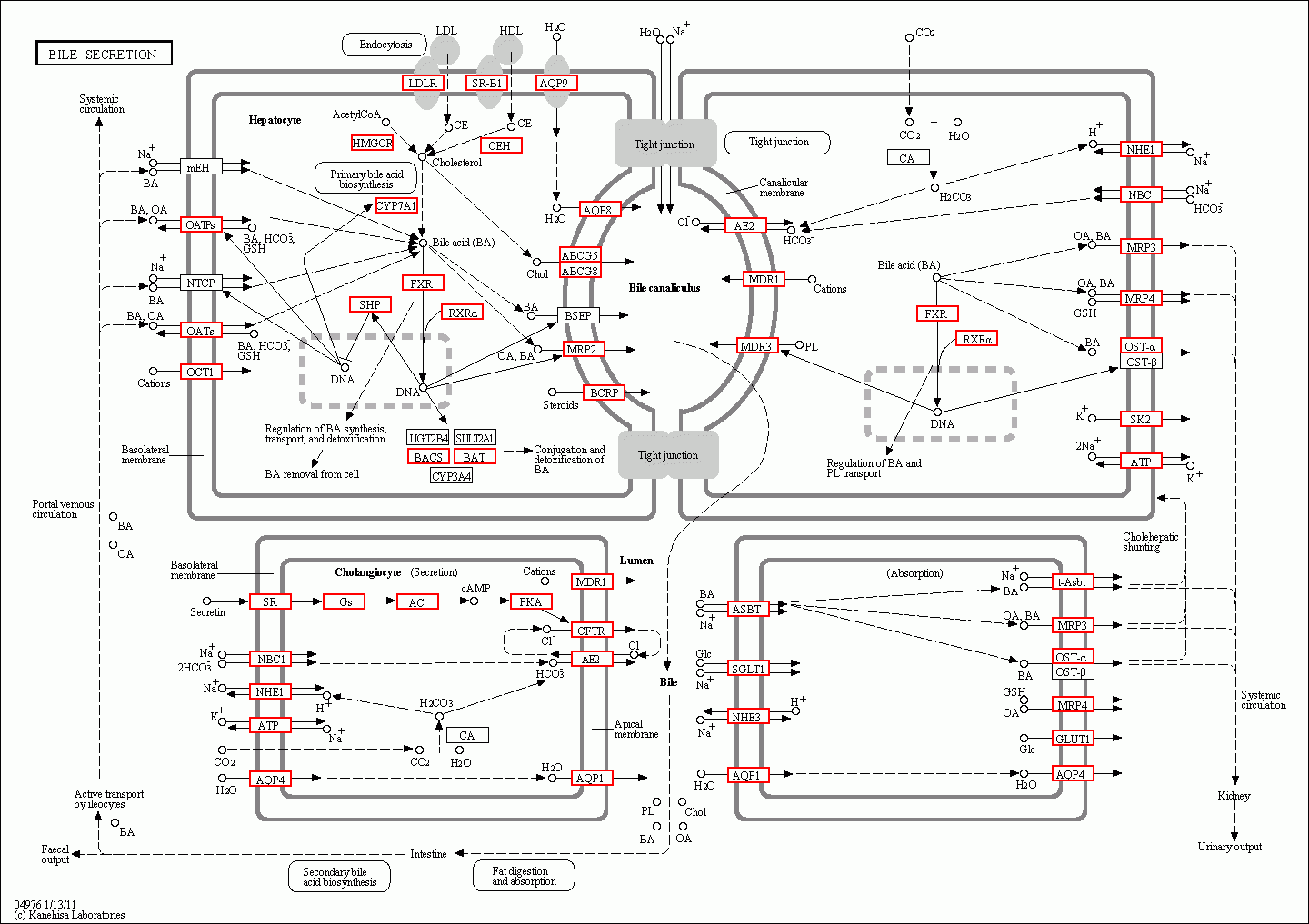

Supplement: Additional file 3: — Pathways found in the annotated portion of the transcriptomes. (ZIP 4950 kb) [file 12864_2015_1817_MOESM3_ESM.zip › map04976.png]

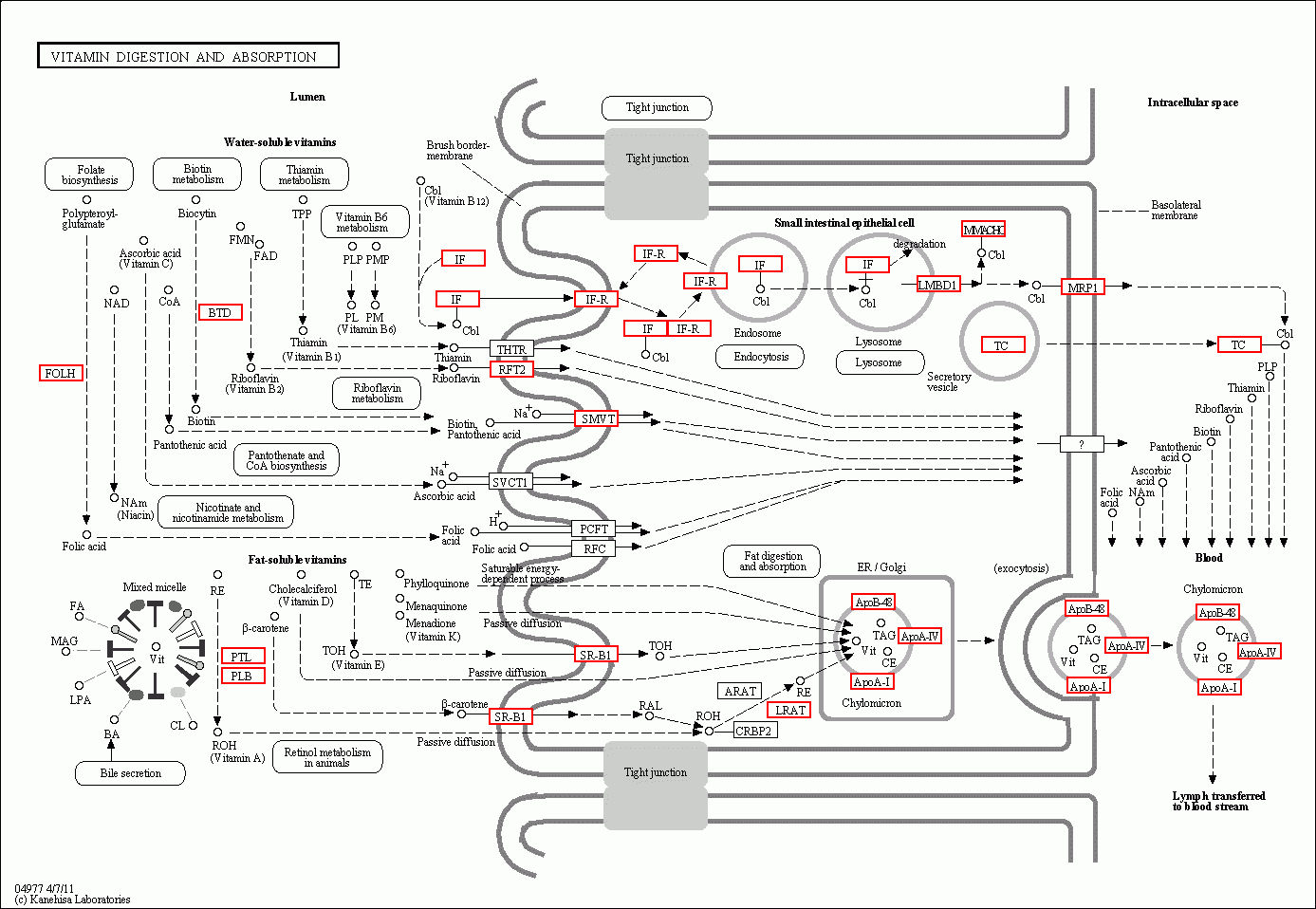

Supplement: Additional file 3: — Pathways found in the annotated portion of the transcriptomes. (ZIP 4950 kb) [file 12864_2015_1817_MOESM3_ESM.zip › map04977.png]

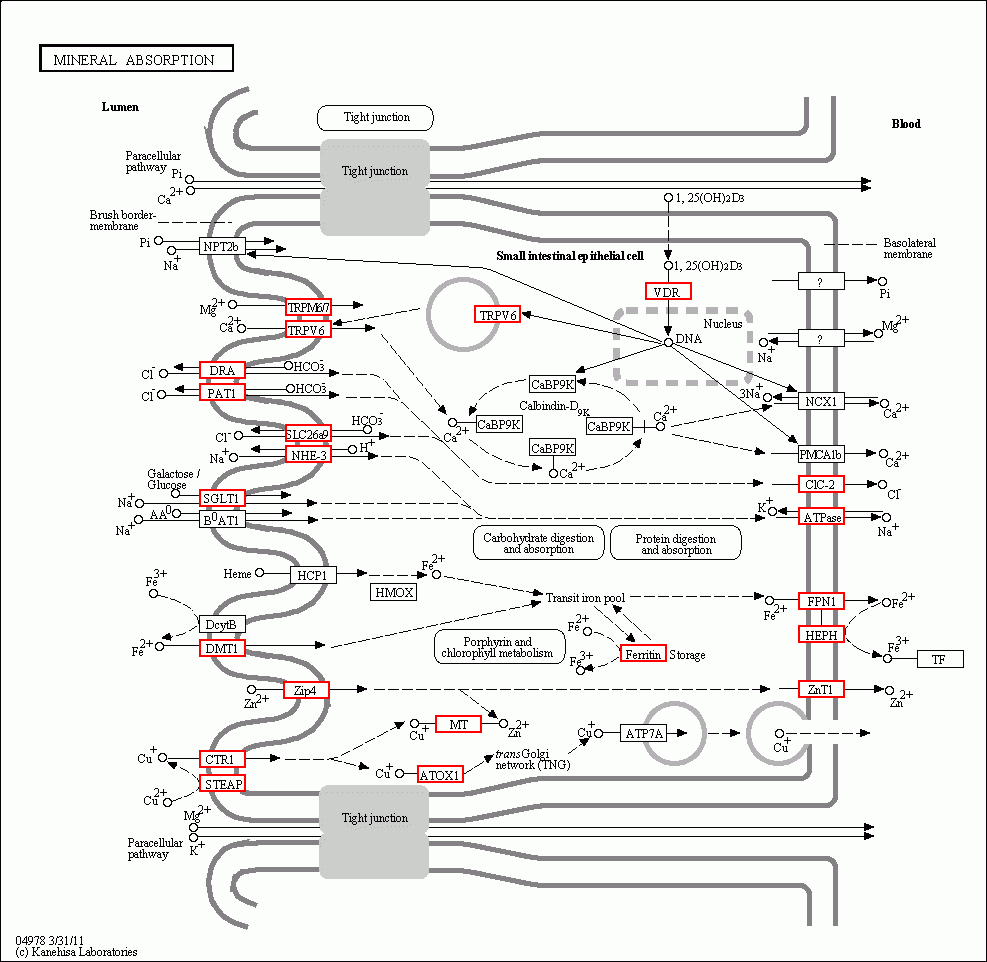

Supplement: Additional file 3: — Pathways found in the annotated portion of the transcriptomes. (ZIP 4950 kb) [file 12864_2015_1817_MOESM3_ESM.zip › map04978.png]

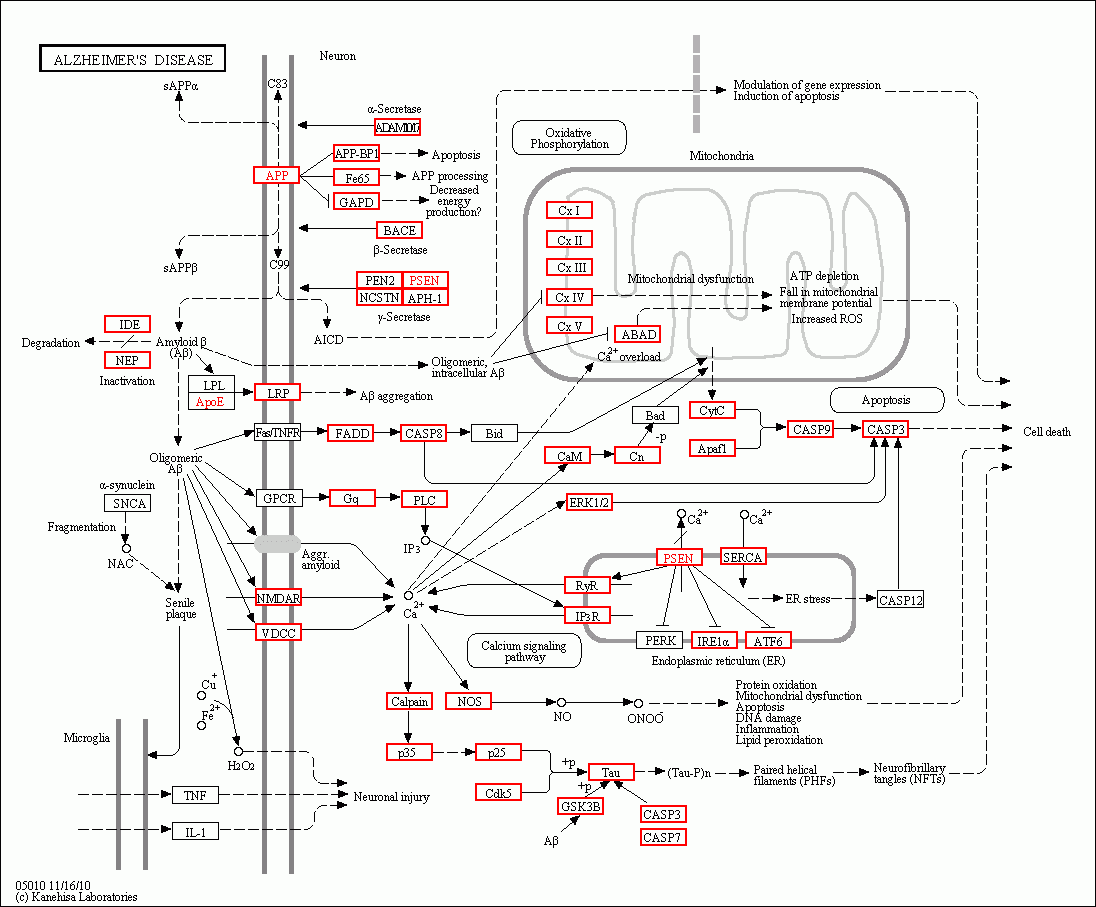

Supplement: Additional file 3: — Pathways found in the annotated portion of the transcriptomes. (ZIP 4950 kb) [file 12864_2015_1817_MOESM3_ESM.zip › map05010.png]

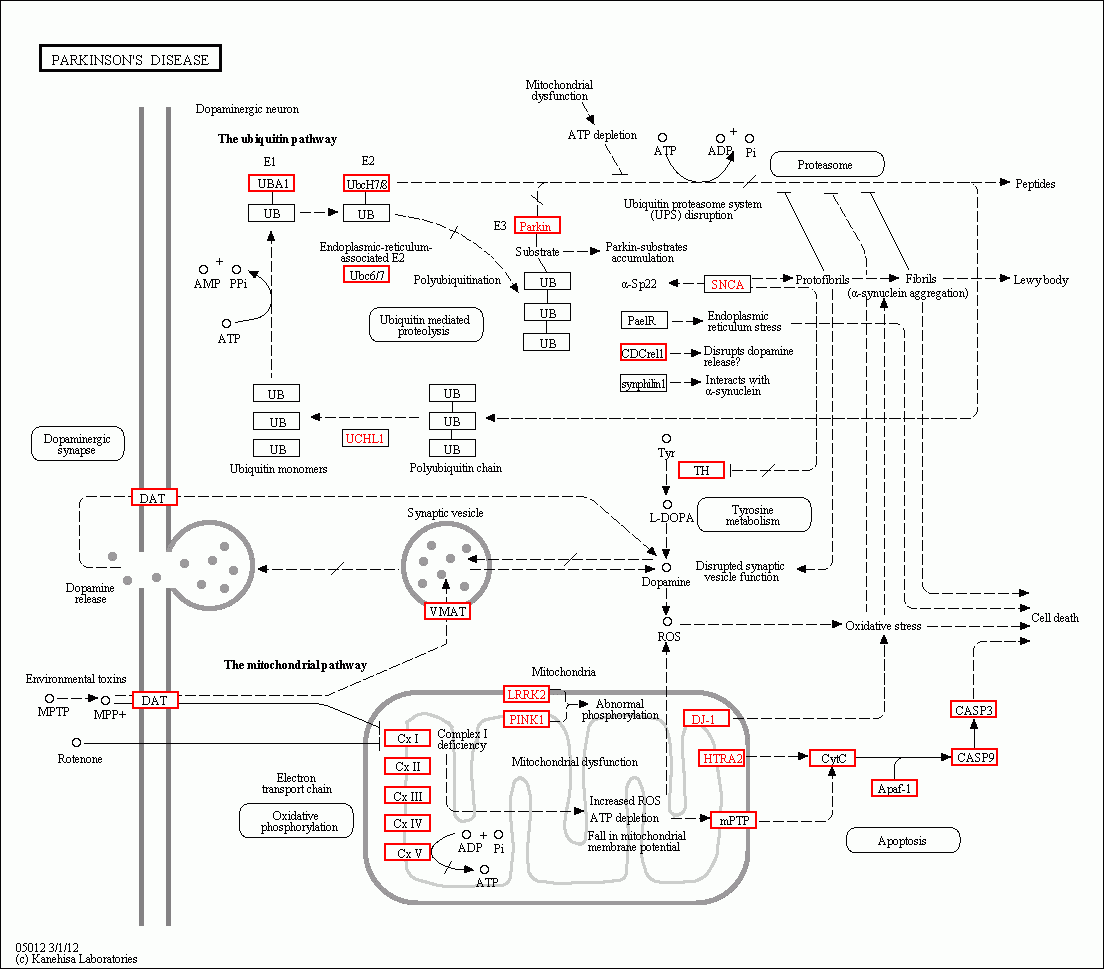

Supplement: Additional file 3: — Pathways found in the annotated portion of the transcriptomes. (ZIP 4950 kb) [file 12864_2015_1817_MOESM3_ESM.zip › map05012.png]

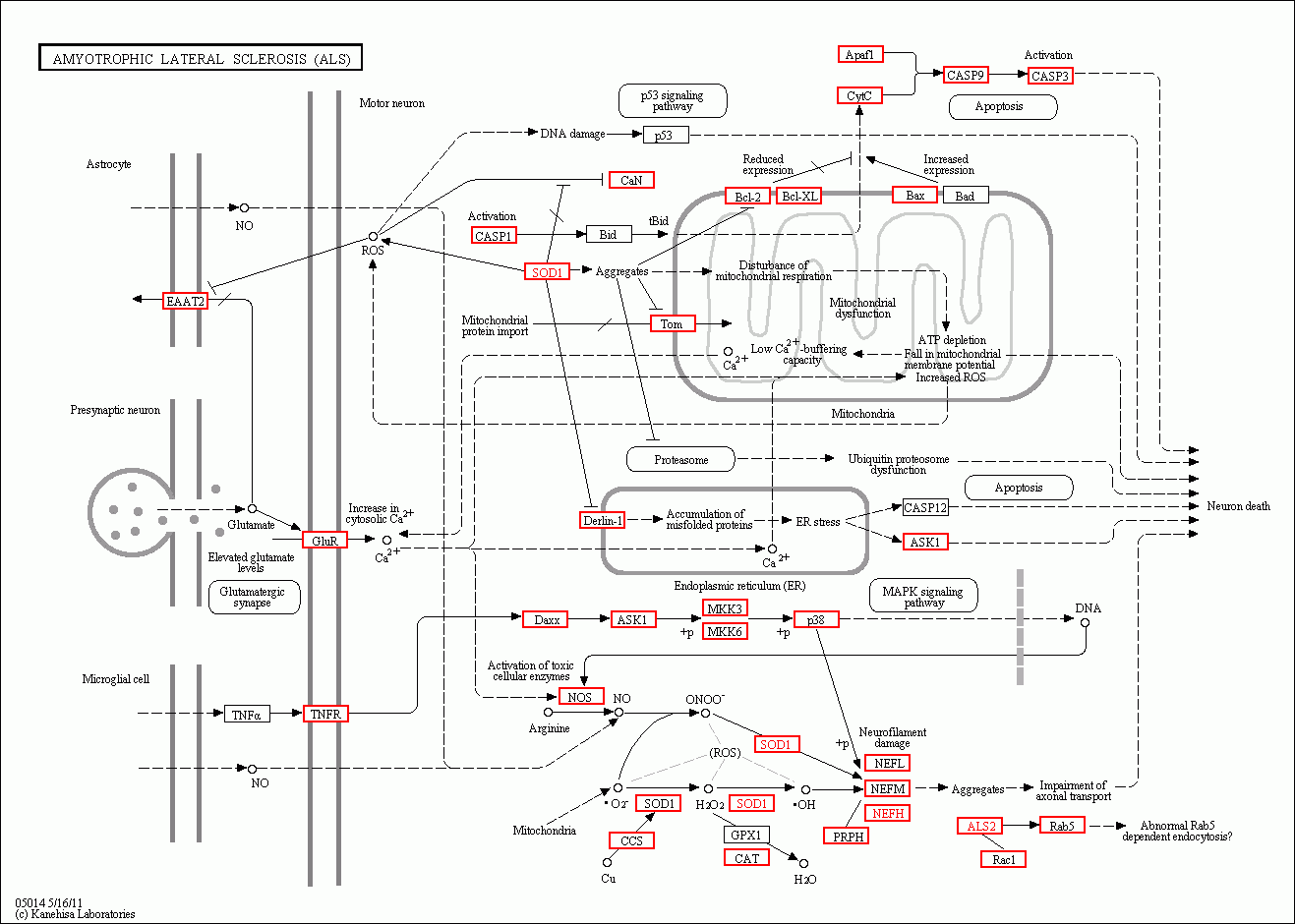

Supplement: Additional file 3: — Pathways found in the annotated portion of the transcriptomes. (ZIP 4950 kb) [file 12864_2015_1817_MOESM3_ESM.zip › map05014.png]

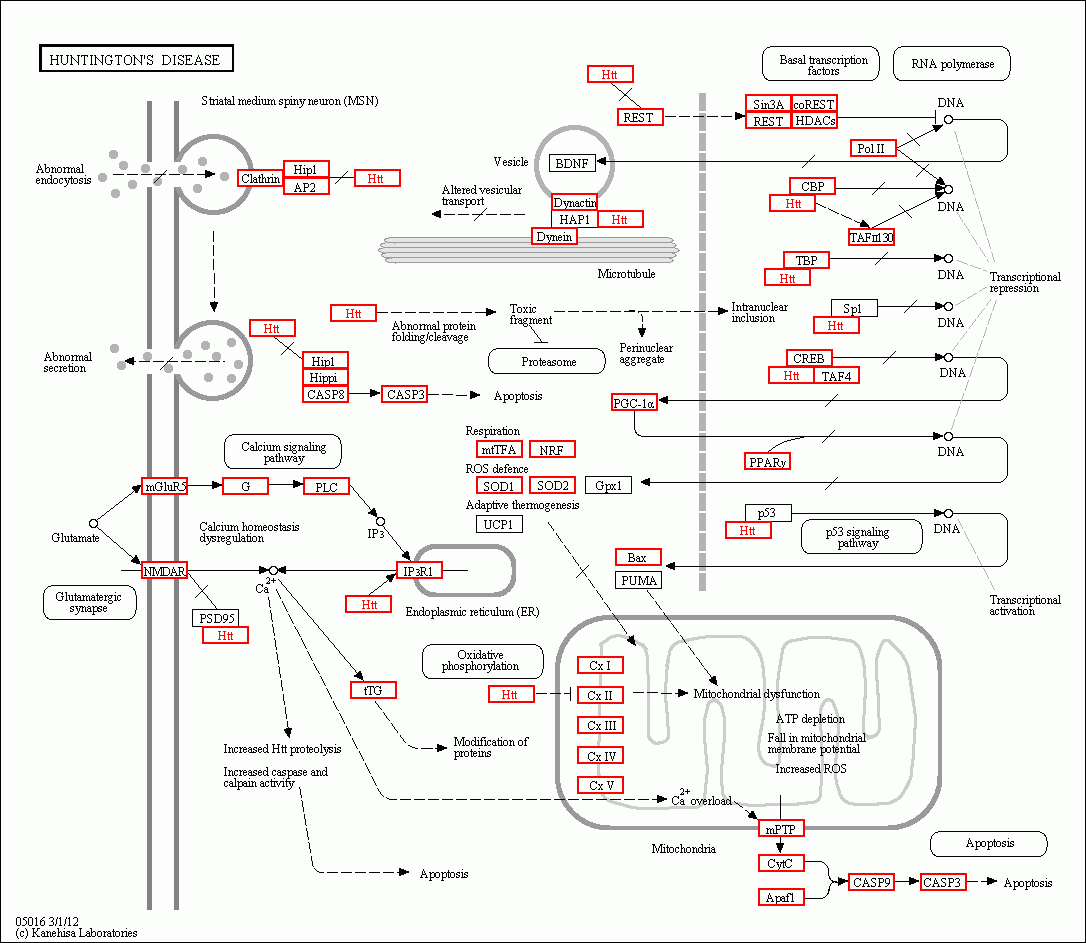

Supplement: Additional file 3: — Pathways found in the annotated portion of the transcriptomes. (ZIP 4950 kb) [file 12864_2015_1817_MOESM3_ESM.zip › map05016.png]

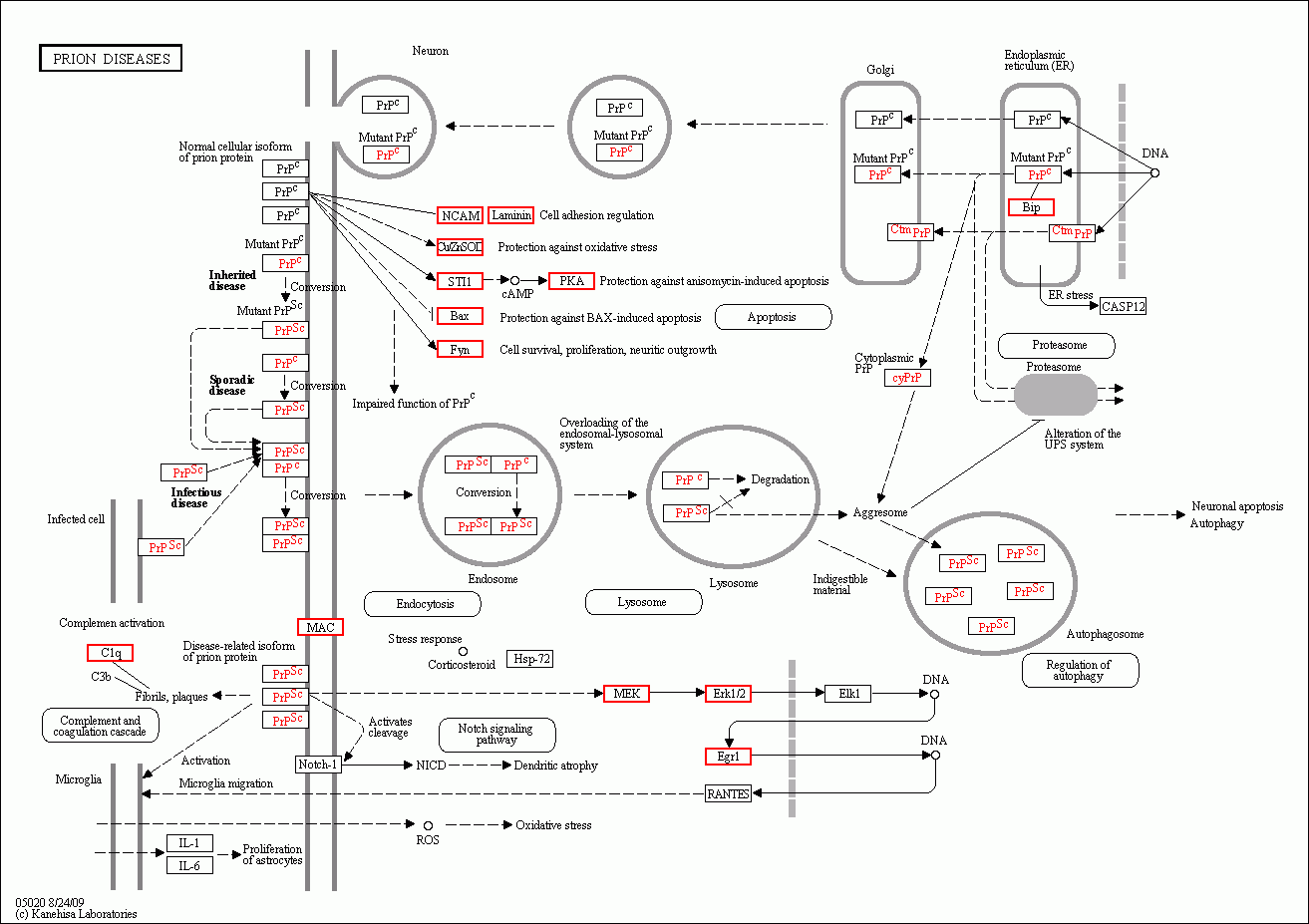

Supplement: Additional file 3: — Pathways found in the annotated portion of the transcriptomes. (ZIP 4950 kb) [file 12864_2015_1817_MOESM3_ESM.zip › map05020.png]

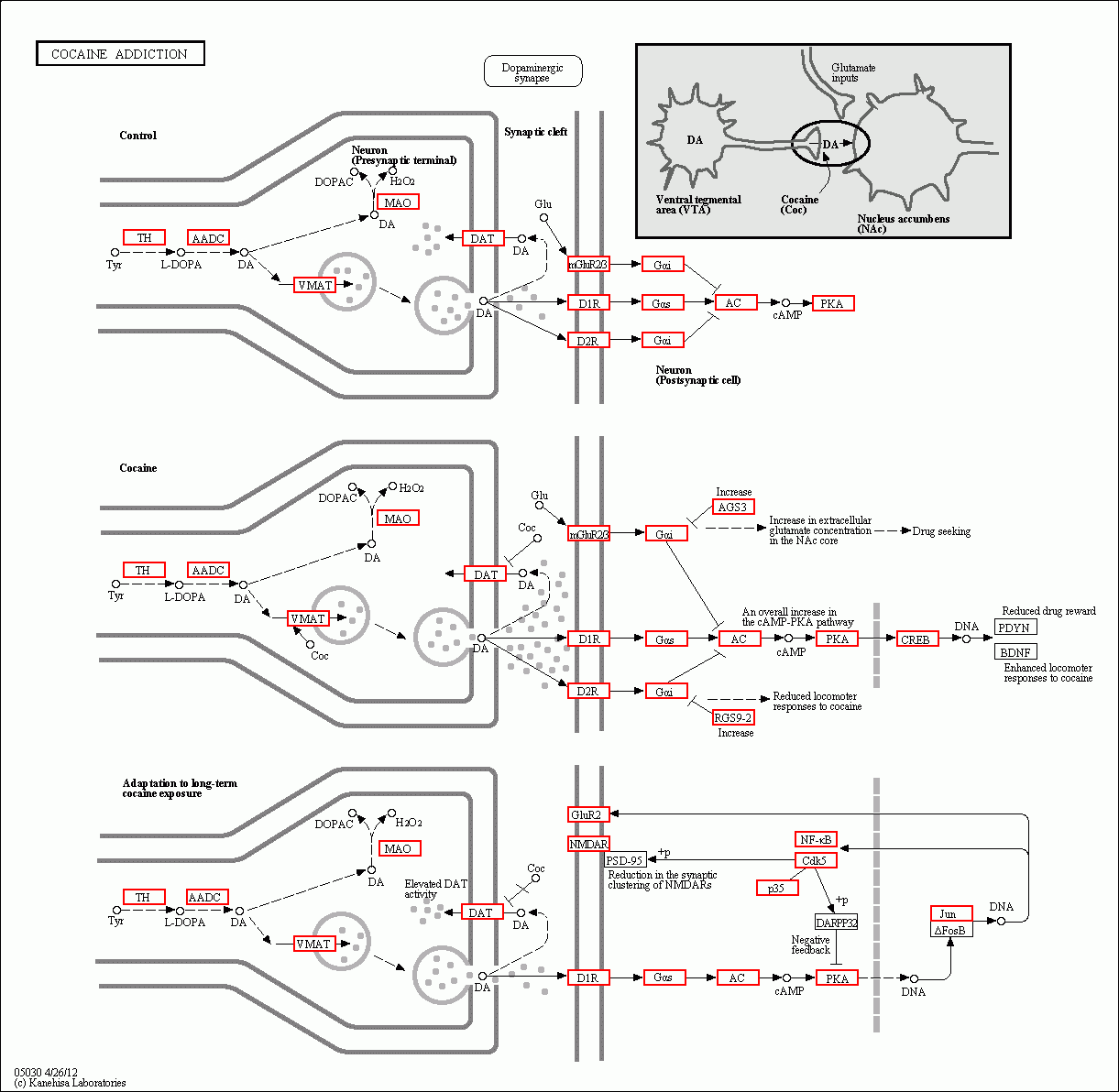

Supplement: Additional file 3: — Pathways found in the annotated portion of the transcriptomes. (ZIP 4950 kb) [file 12864_2015_1817_MOESM3_ESM.zip › map05030.png]

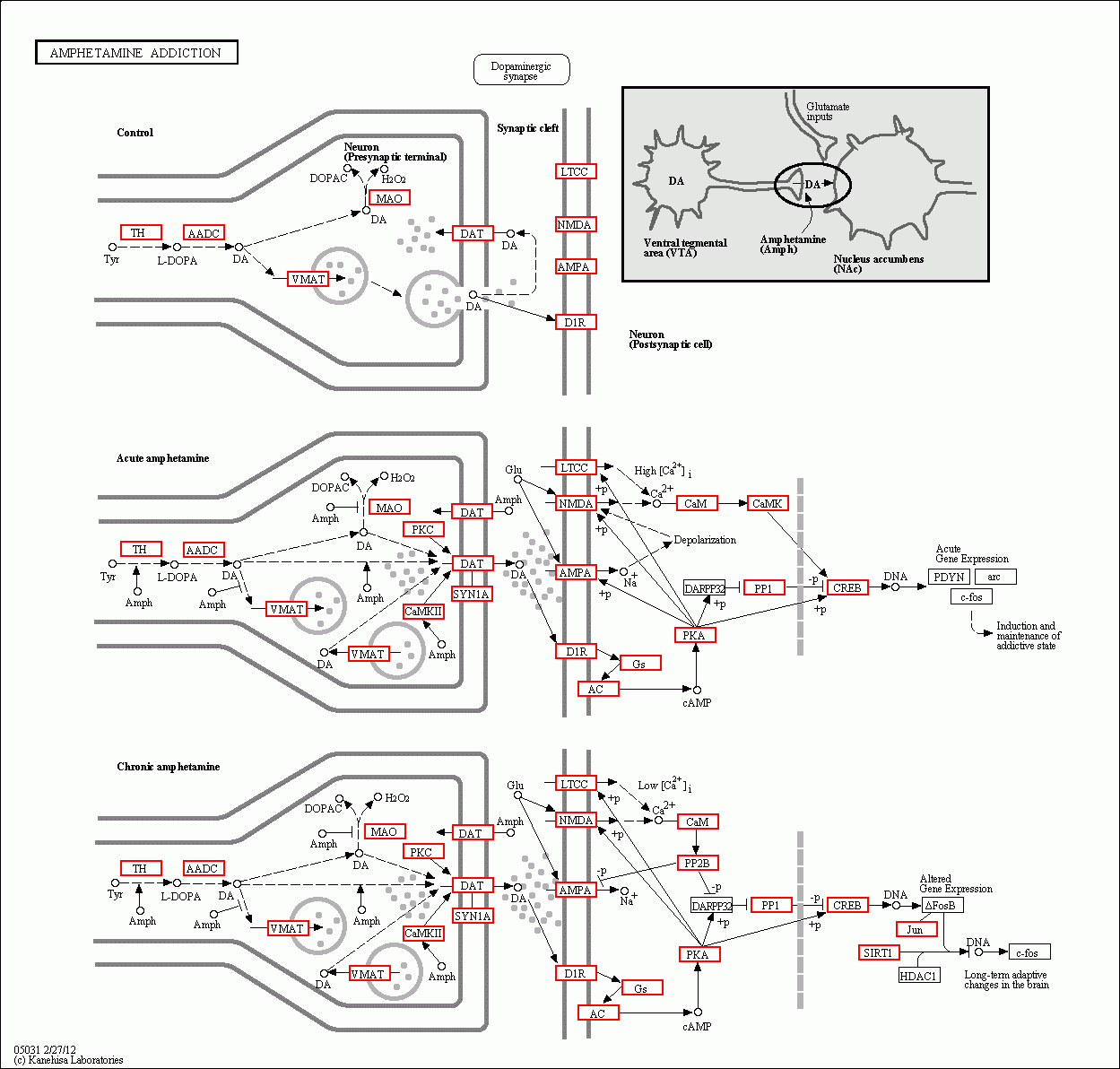

Supplement: Additional file 3: — Pathways found in the annotated portion of the transcriptomes. (ZIP 4950 kb) [file 12864_2015_1817_MOESM3_ESM.zip › map05031.png]

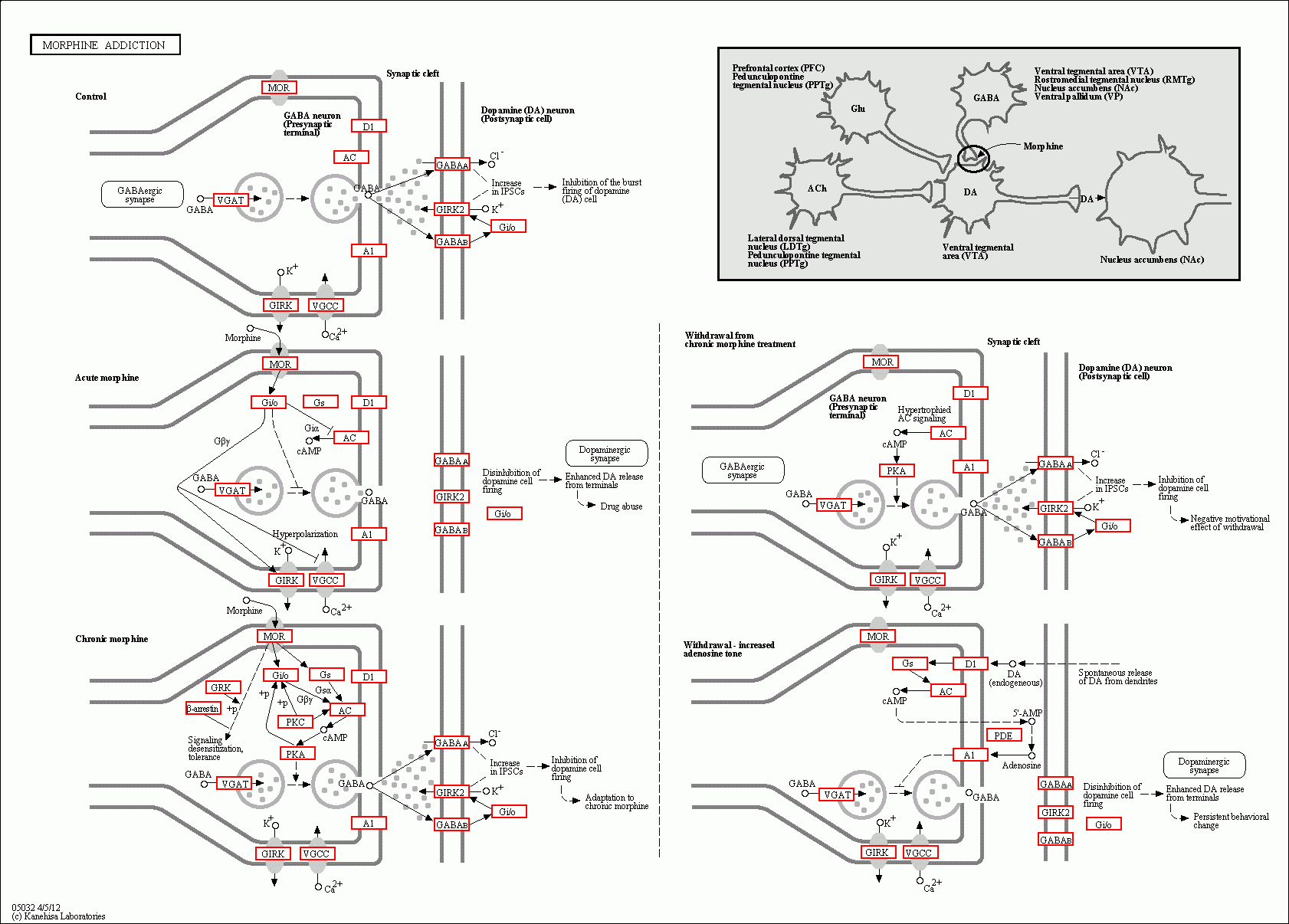

Supplement: Additional file 3: — Pathways found in the annotated portion of the transcriptomes. (ZIP 4950 kb) [file 12864_2015_1817_MOESM3_ESM.zip › map05032.png]

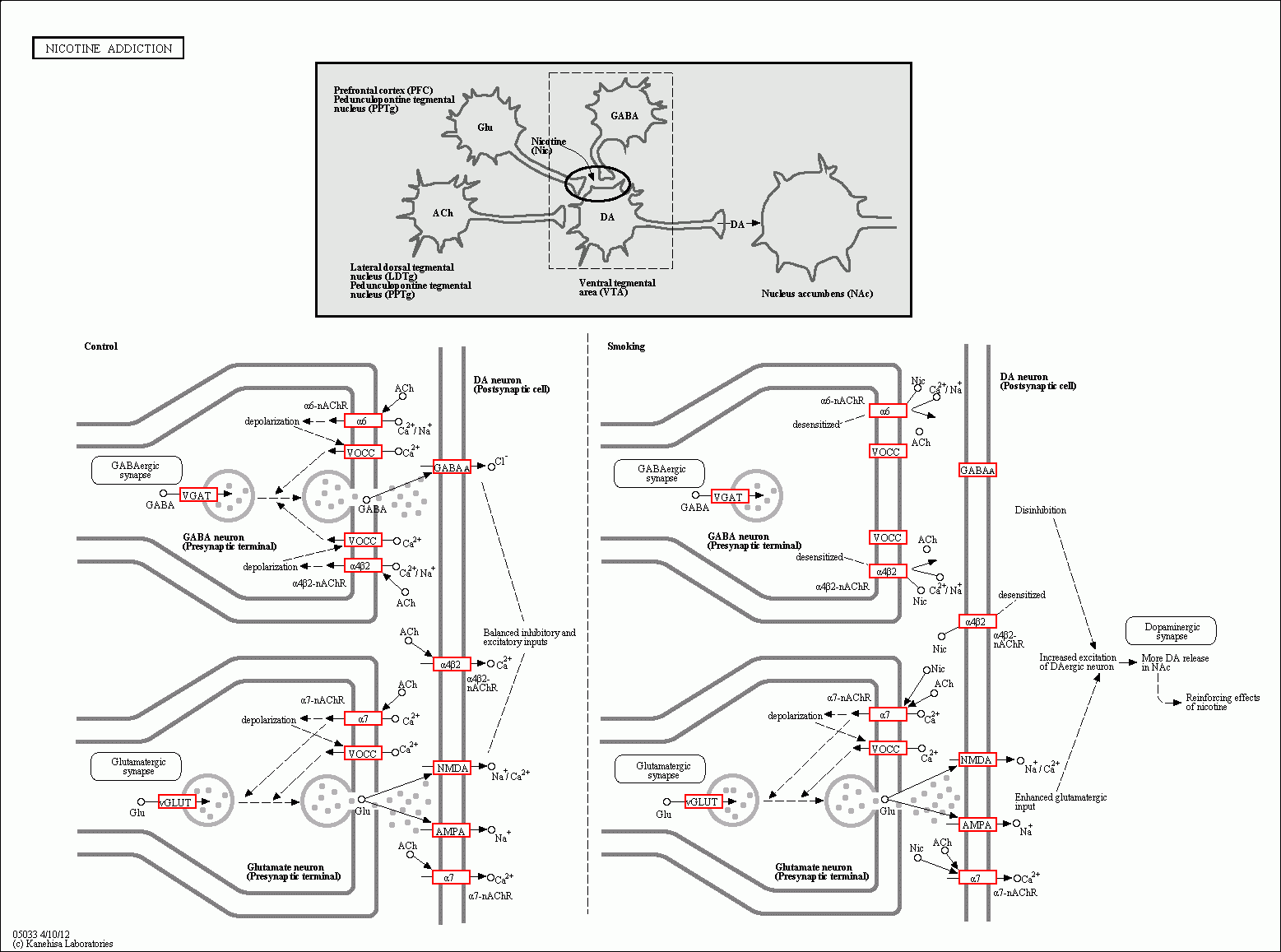

Supplement: Additional file 3: — Pathways found in the annotated portion of the transcriptomes. (ZIP 4950 kb) [file 12864_2015_1817_MOESM3_ESM.zip › map05033.png]

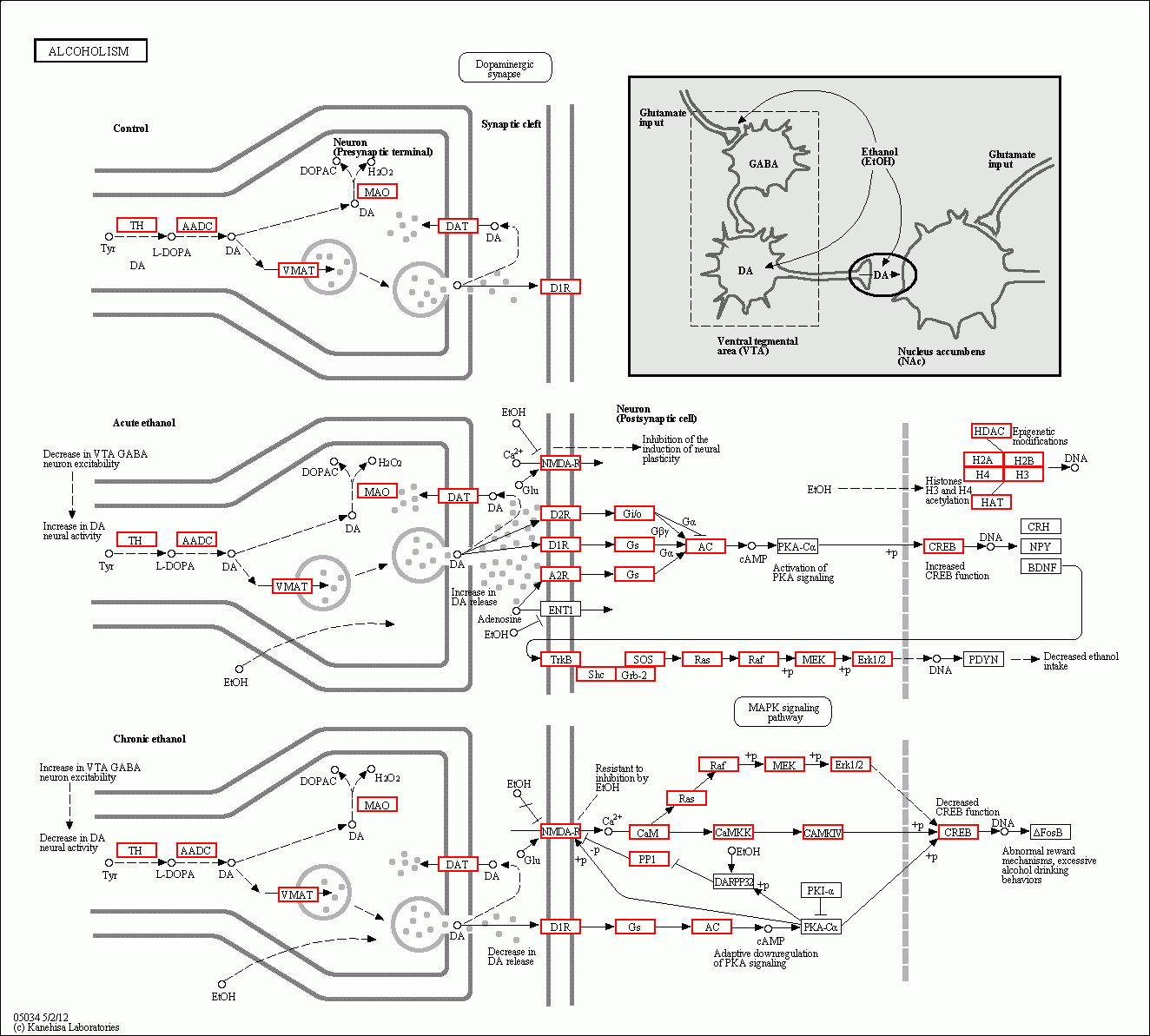

Supplement: Additional file 3: — Pathways found in the annotated portion of the transcriptomes. (ZIP 4950 kb) [file 12864_2015_1817_MOESM3_ESM.zip › map05034.png]

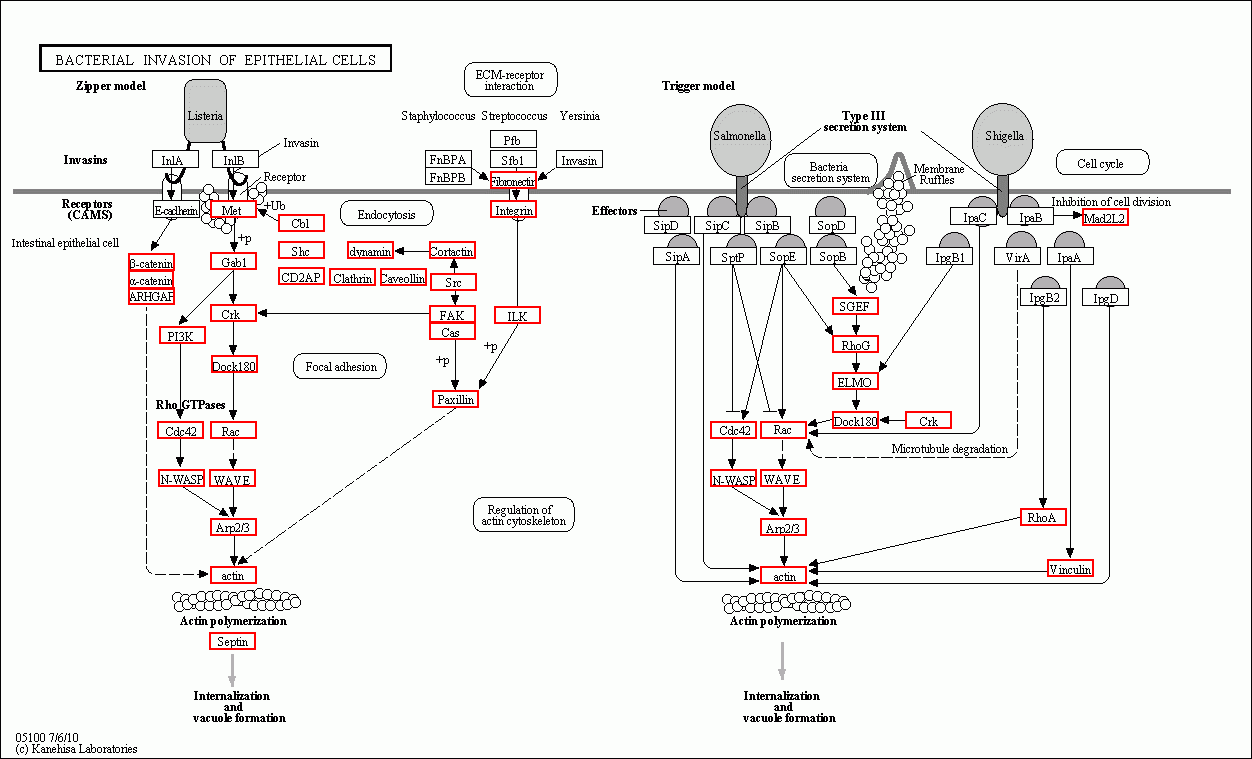

Supplement: Additional file 3: — Pathways found in the annotated portion of the transcriptomes. (ZIP 4950 kb) [file 12864_2015_1817_MOESM3_ESM.zip › map05100.png]

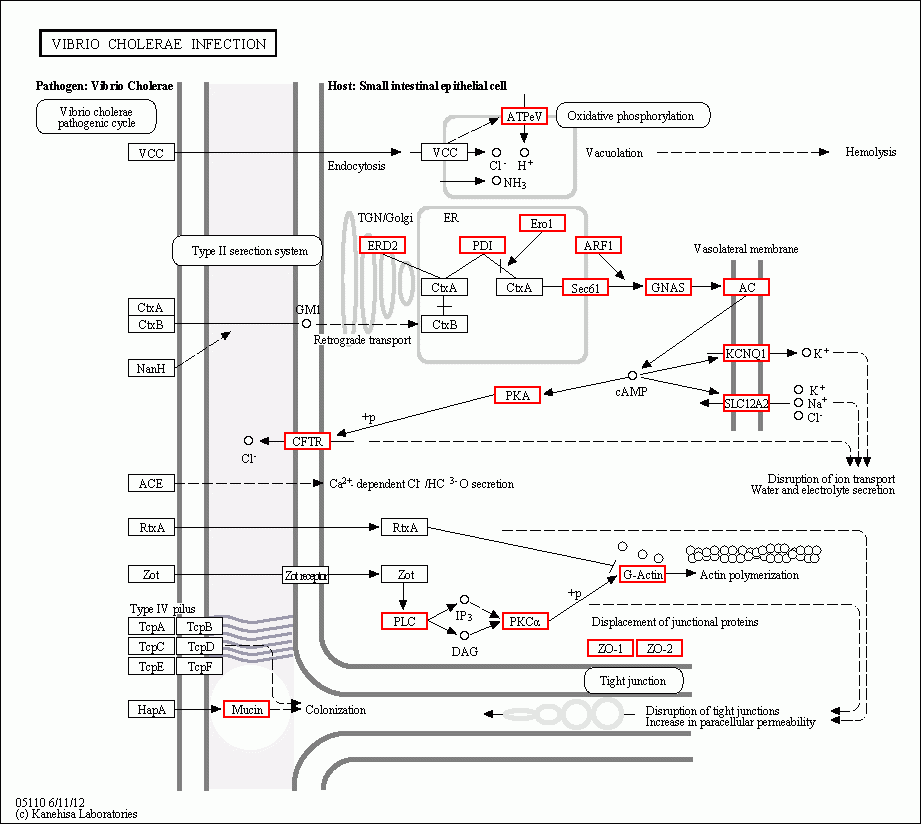

Supplement: Additional file 3: — Pathways found in the annotated portion of the transcriptomes. (ZIP 4950 kb) [file 12864_2015_1817_MOESM3_ESM.zip › map05110.png]

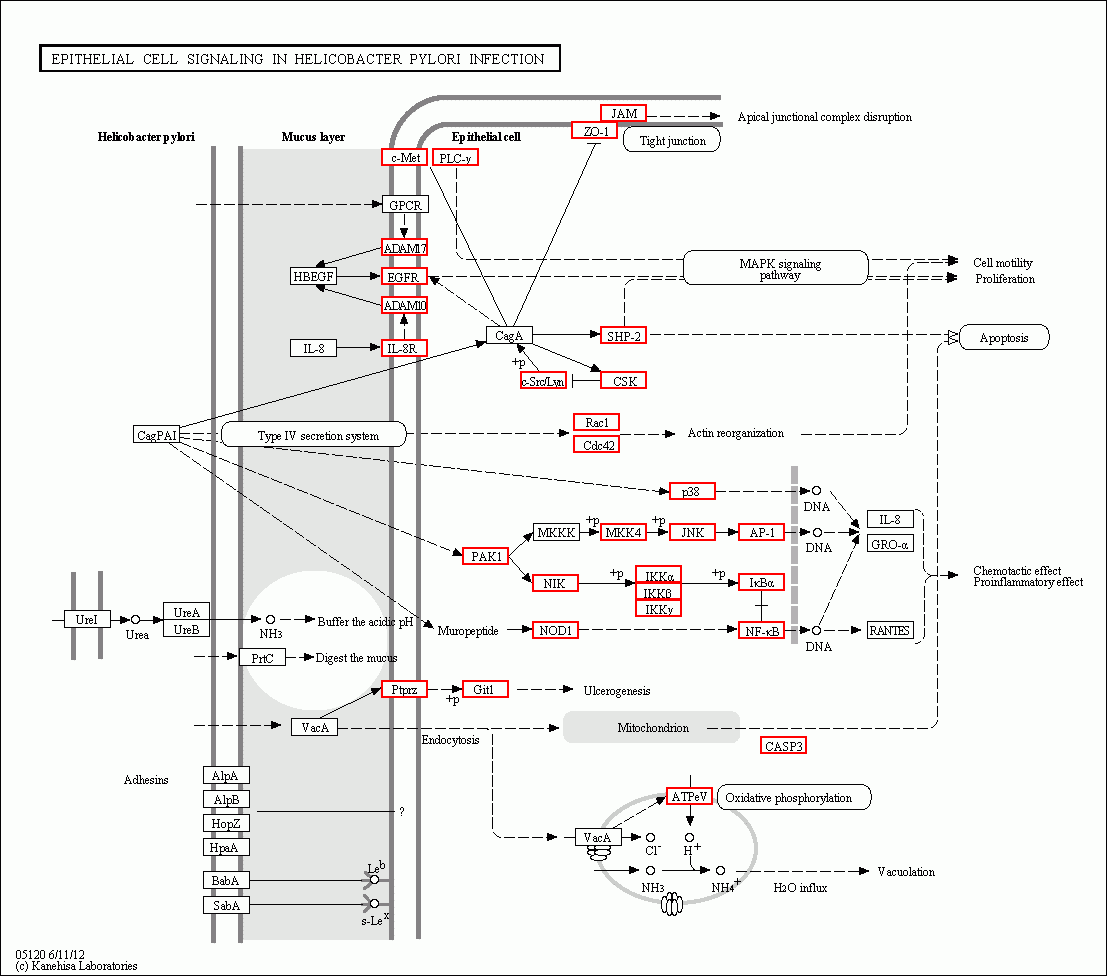

Supplement: Additional file 3: — Pathways found in the annotated portion of the transcriptomes. (ZIP 4950 kb) [file 12864_2015_1817_MOESM3_ESM.zip › map05120.png]

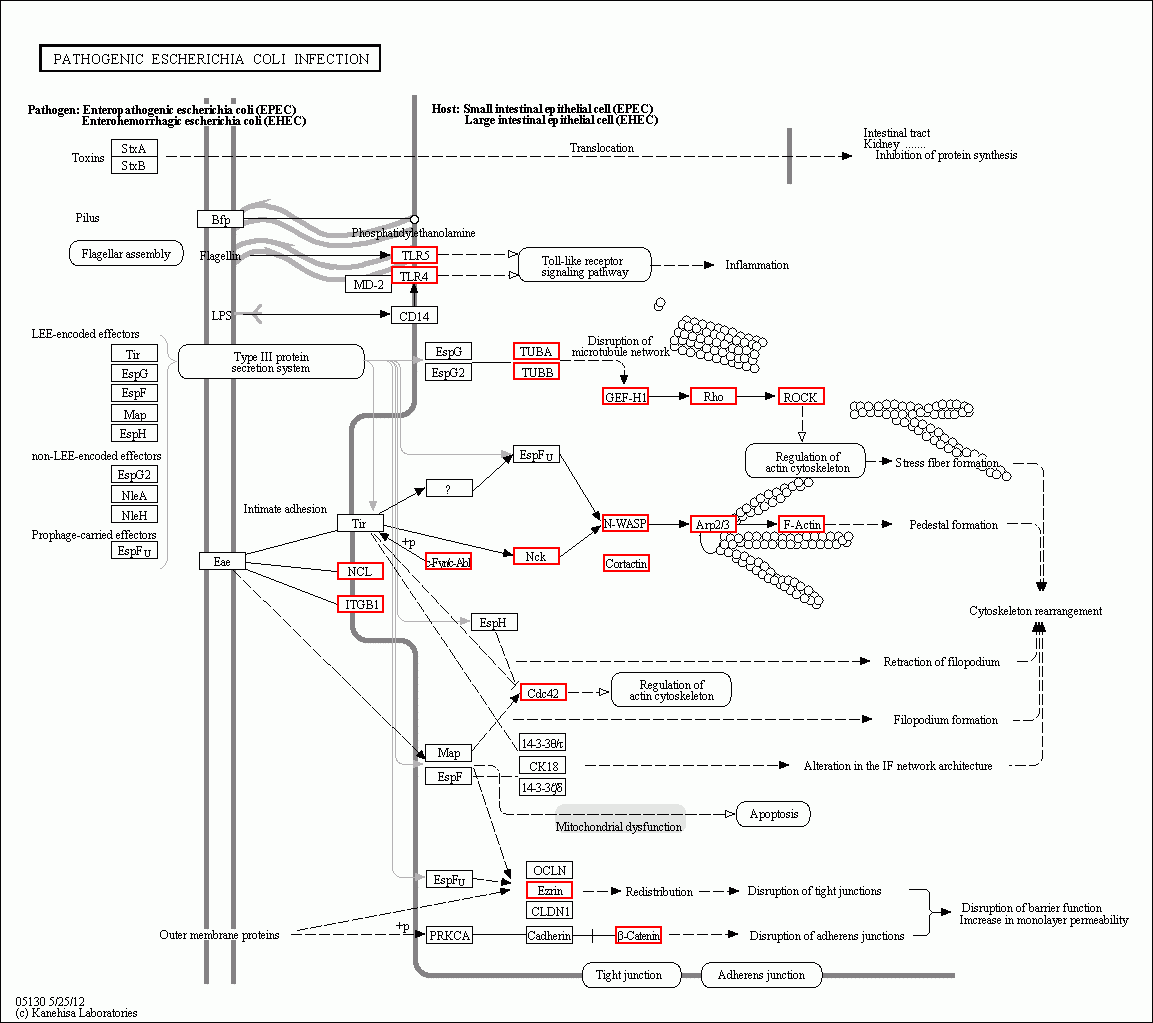

Supplement: Additional file 3: — Pathways found in the annotated portion of the transcriptomes. (ZIP 4950 kb) [file 12864_2015_1817_MOESM3_ESM.zip › map05130.png]

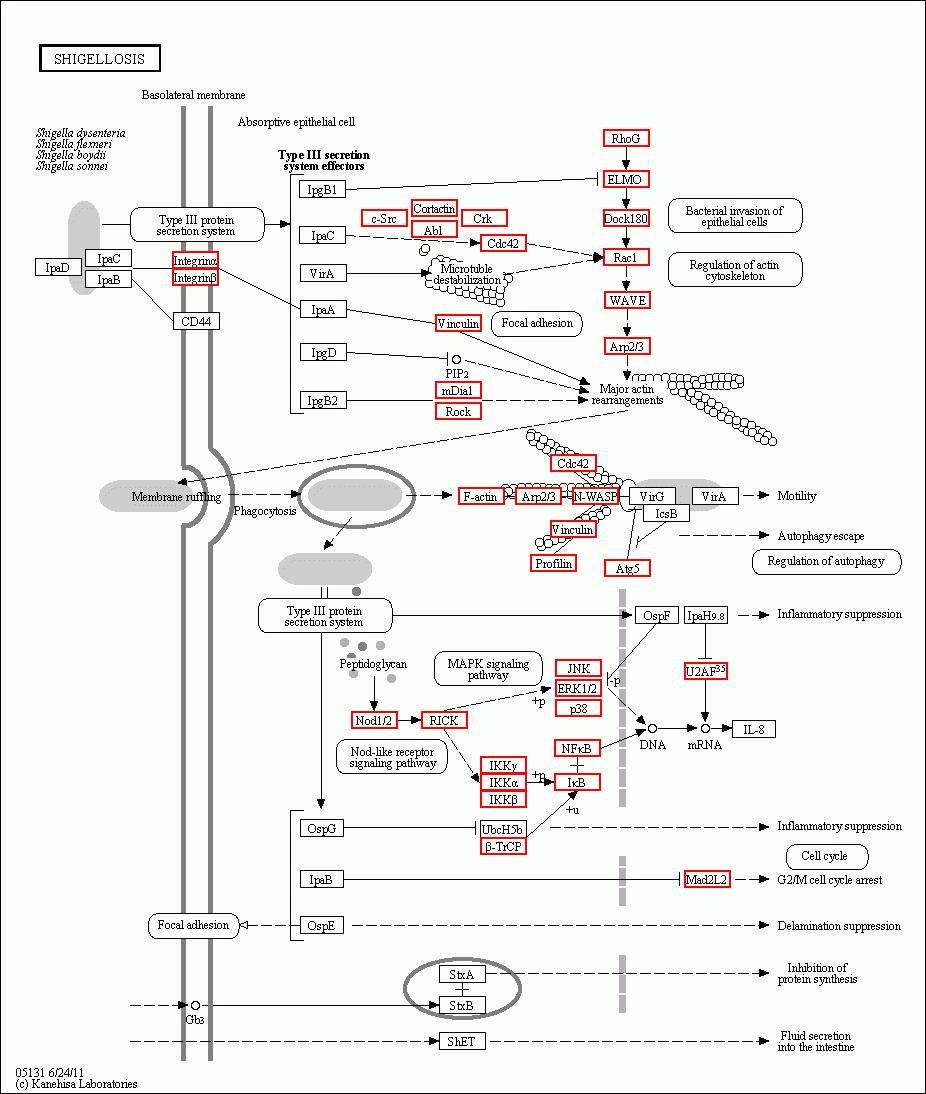

Supplement: Additional file 3: — Pathways found in the annotated portion of the transcriptomes. (ZIP 4950 kb) [file 12864_2015_1817_MOESM3_ESM.zip › map05131.png]

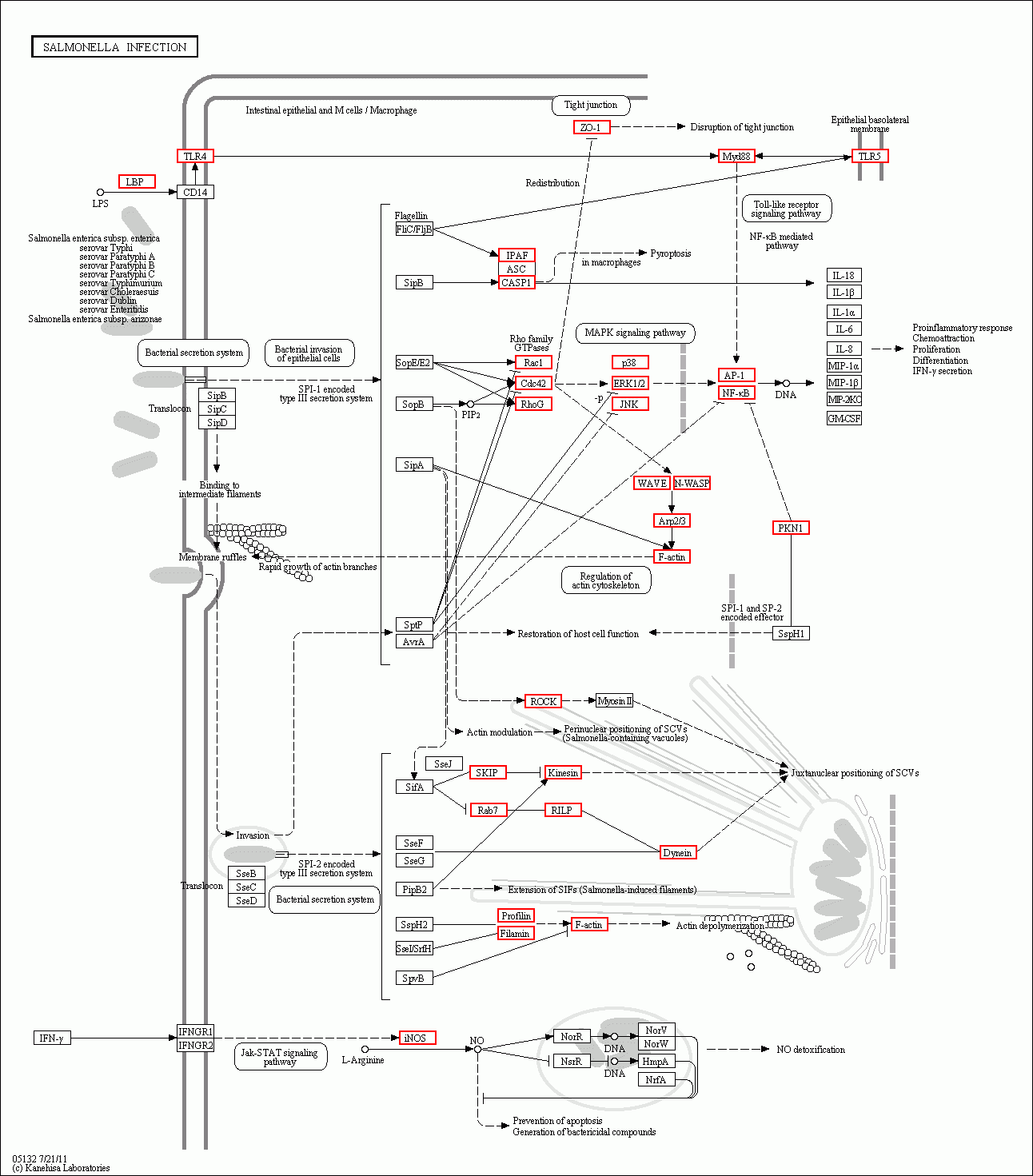

Supplement: Additional file 3: — Pathways found in the annotated portion of the transcriptomes. (ZIP 4950 kb) [file 12864_2015_1817_MOESM3_ESM.zip › map05132.png]

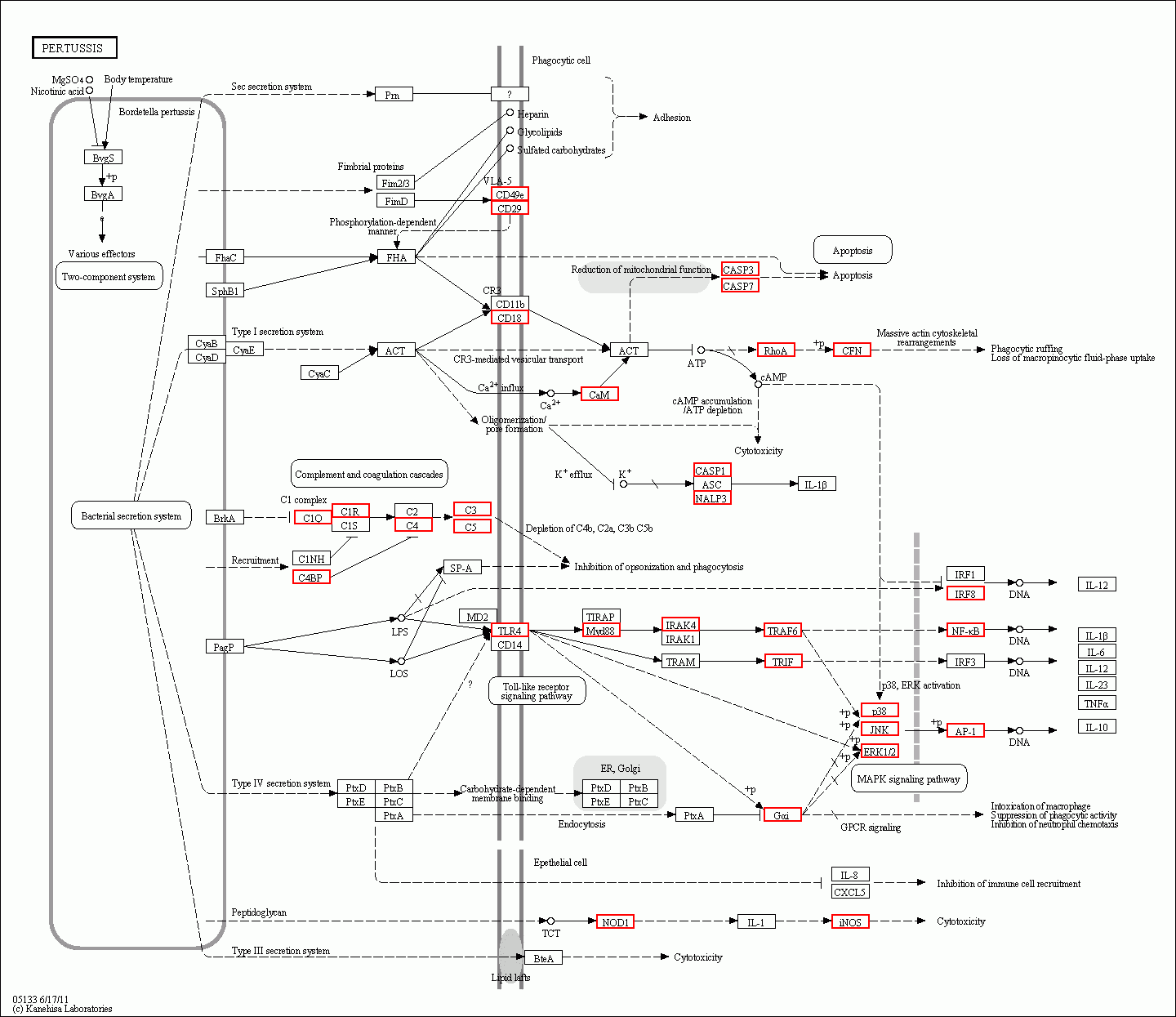

Supplement: Additional file 3: — Pathways found in the annotated portion of the transcriptomes. (ZIP 4950 kb) [file 12864_2015_1817_MOESM3_ESM.zip › map05133.png]

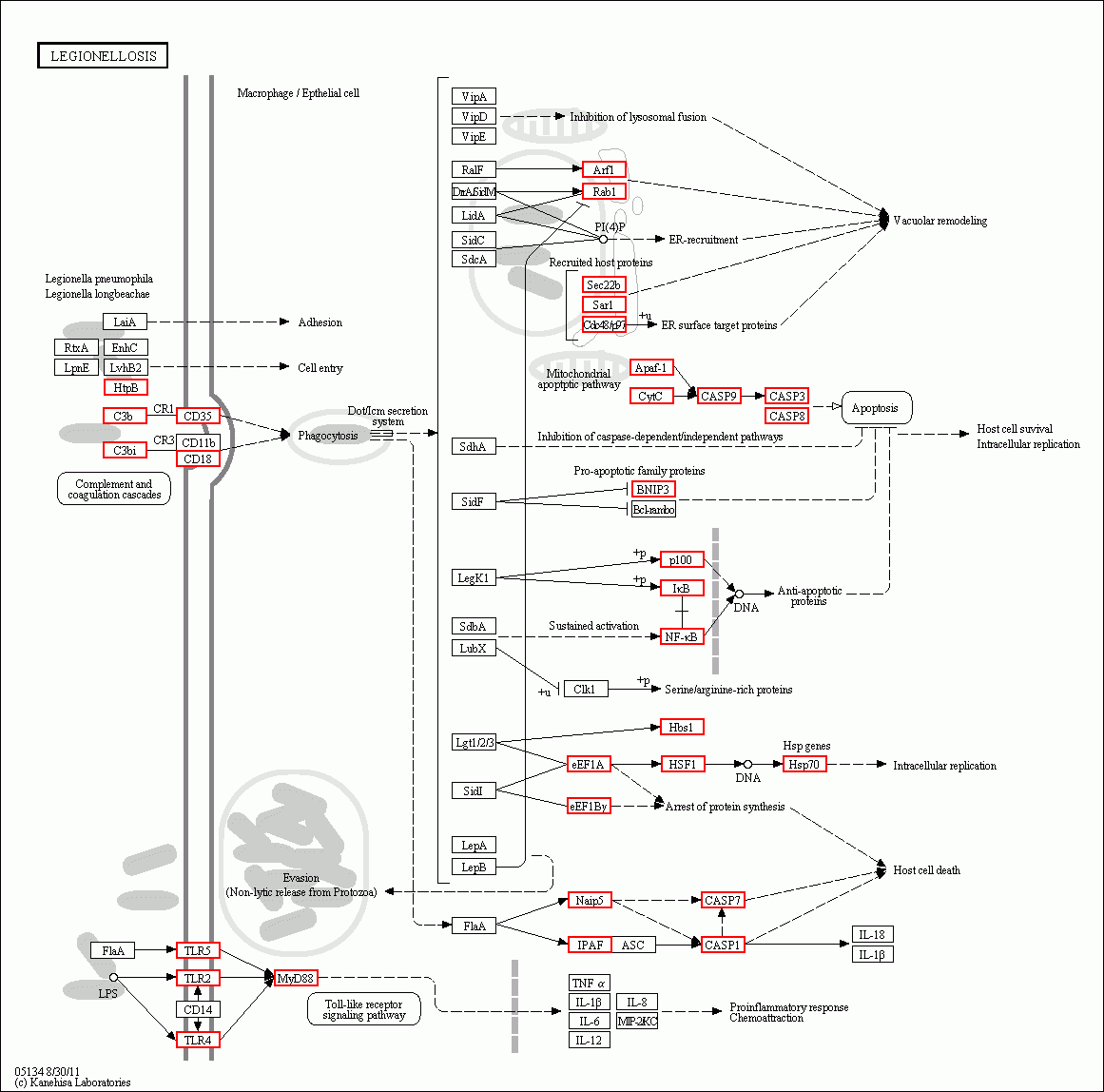

Supplement: Additional file 3: — Pathways found in the annotated portion of the transcriptomes. (ZIP 4950 kb) [file 12864_2015_1817_MOESM3_ESM.zip › map05134.png]

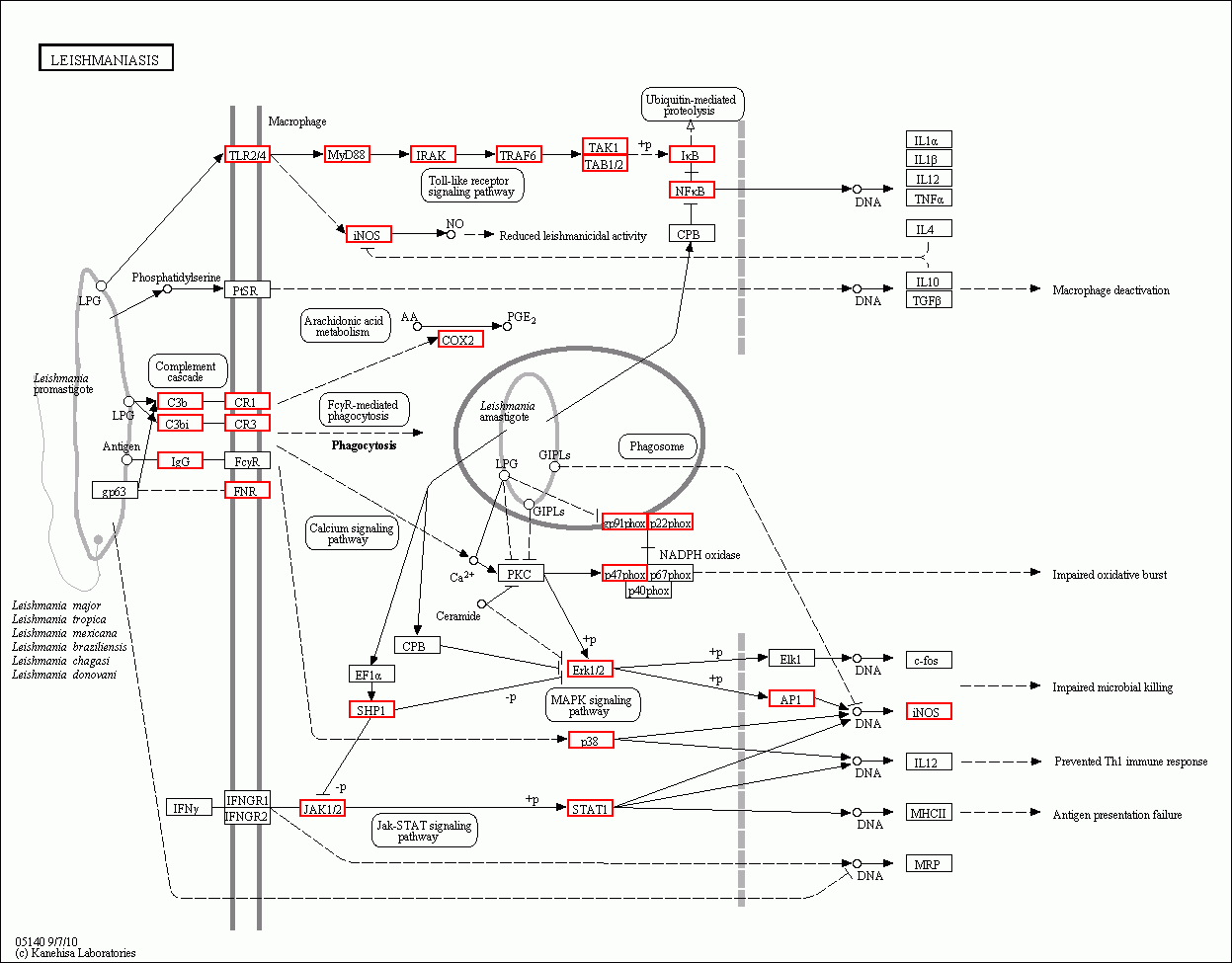

Supplement: Additional file 3: — Pathways found in the annotated portion of the transcriptomes. (ZIP 4950 kb) [file 12864_2015_1817_MOESM3_ESM.zip › map05140.png]

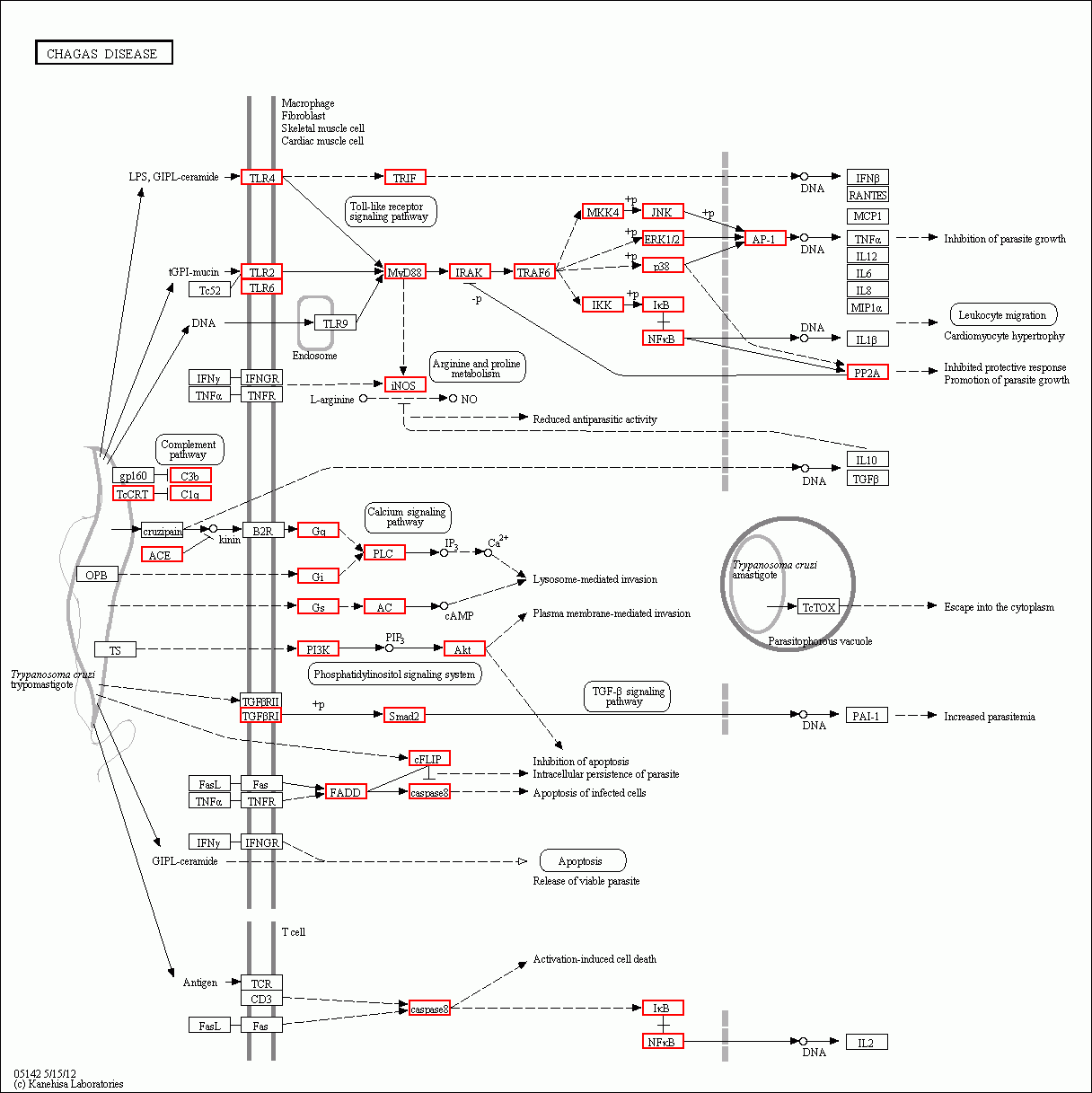

Supplement: Additional file 3: — Pathways found in the annotated portion of the transcriptomes. (ZIP 4950 kb) [file 12864_2015_1817_MOESM3_ESM.zip › map05142.png]

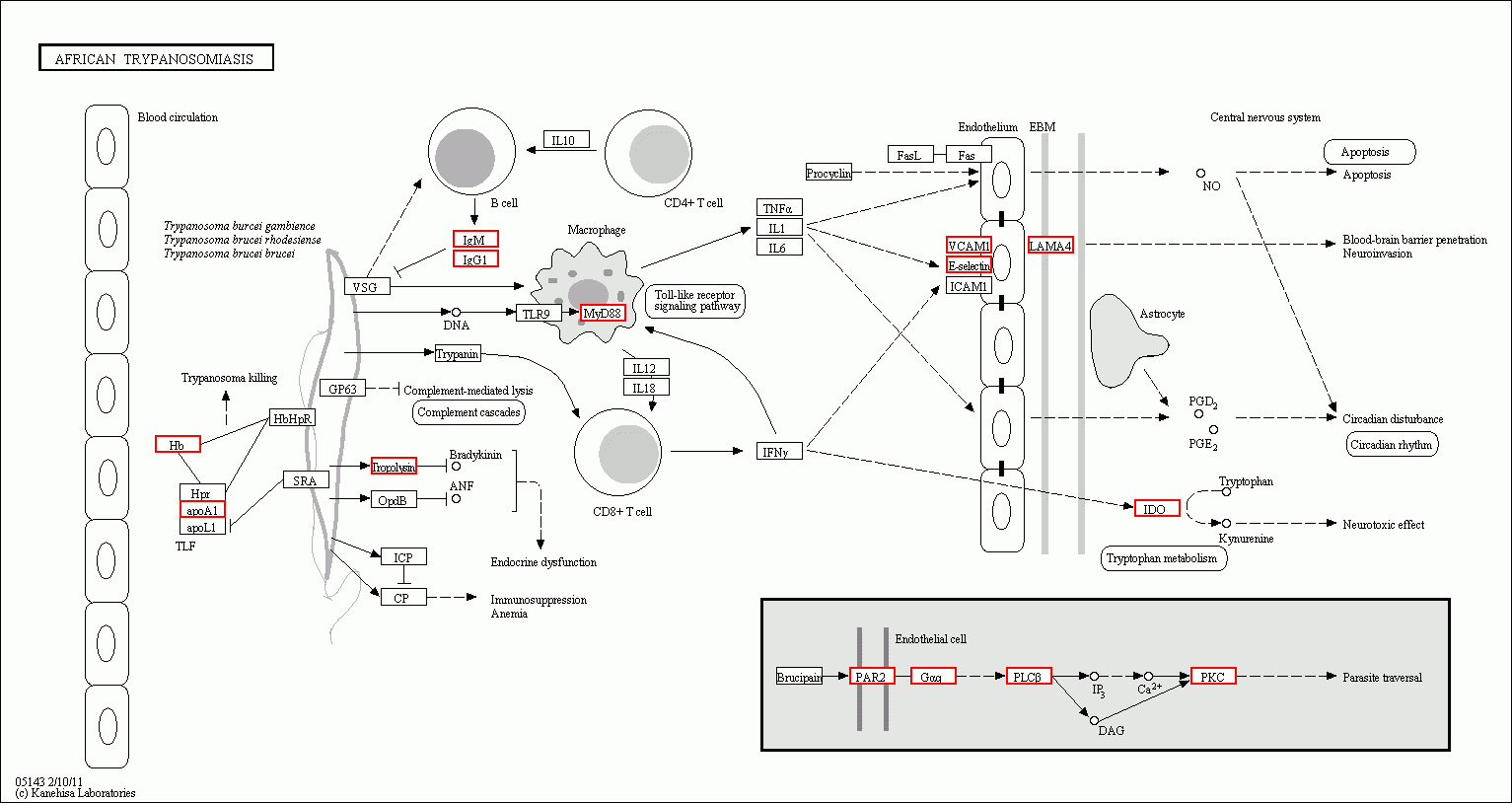

Supplement: Additional file 3: — Pathways found in the annotated portion of the transcriptomes. (ZIP 4950 kb) [file 12864_2015_1817_MOESM3_ESM.zip › map05143.png]

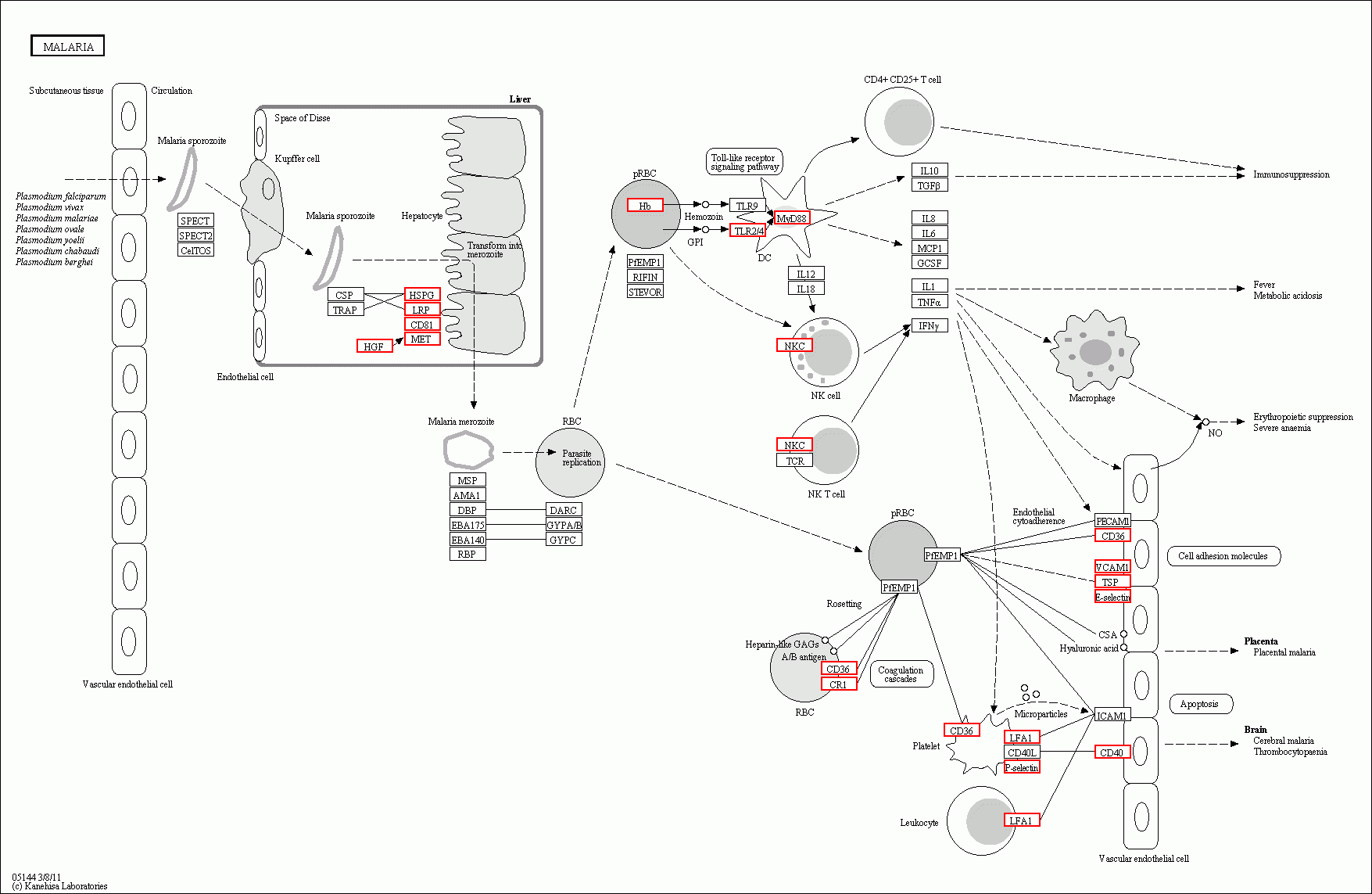

Supplement: Additional file 3: — Pathways found in the annotated portion of the transcriptomes. (ZIP 4950 kb) [file 12864_2015_1817_MOESM3_ESM.zip › map05144.png]

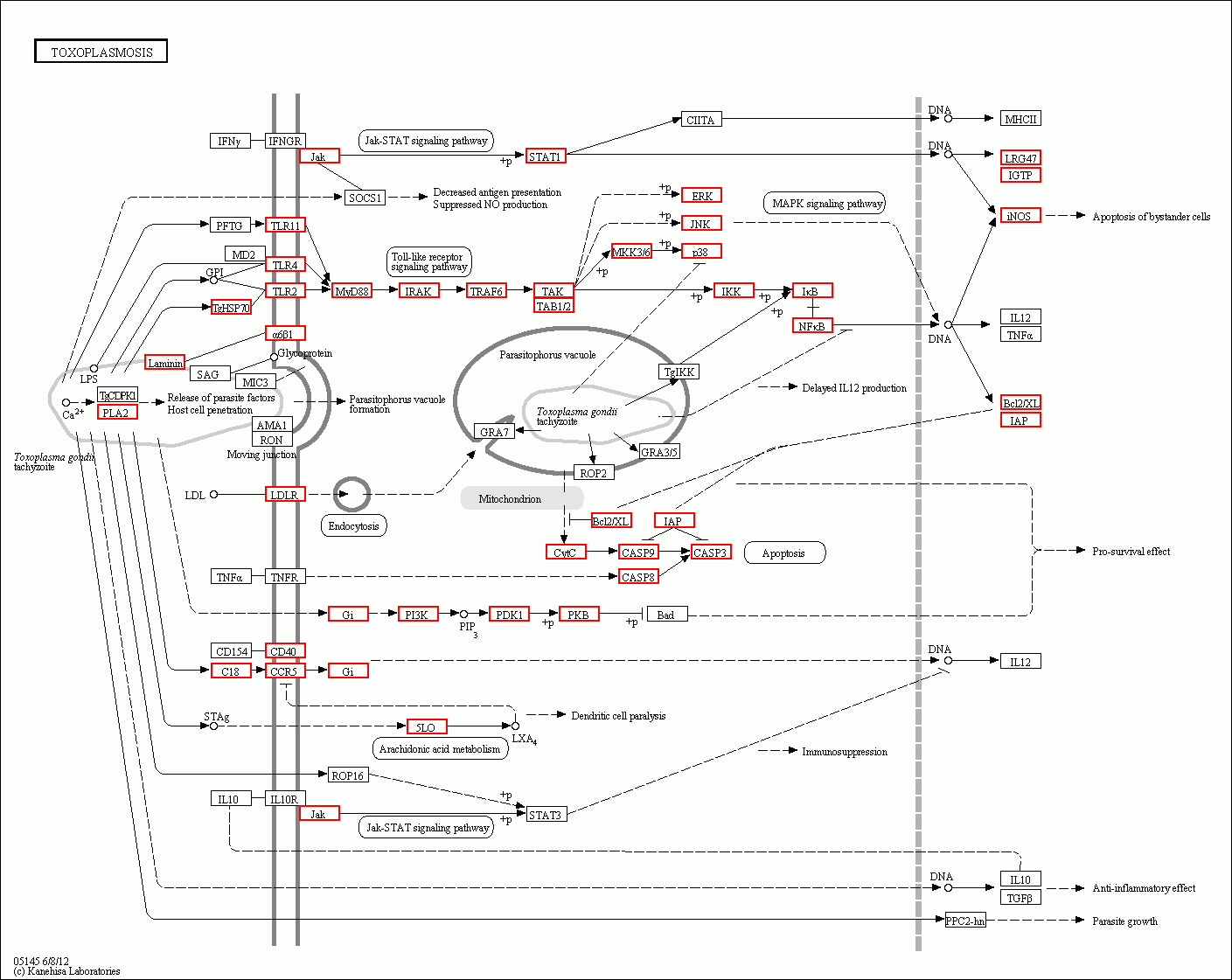

Supplement: Additional file 3: — Pathways found in the annotated portion of the transcriptomes. (ZIP 4950 kb) [file 12864_2015_1817_MOESM3_ESM.zip › map05145.png]

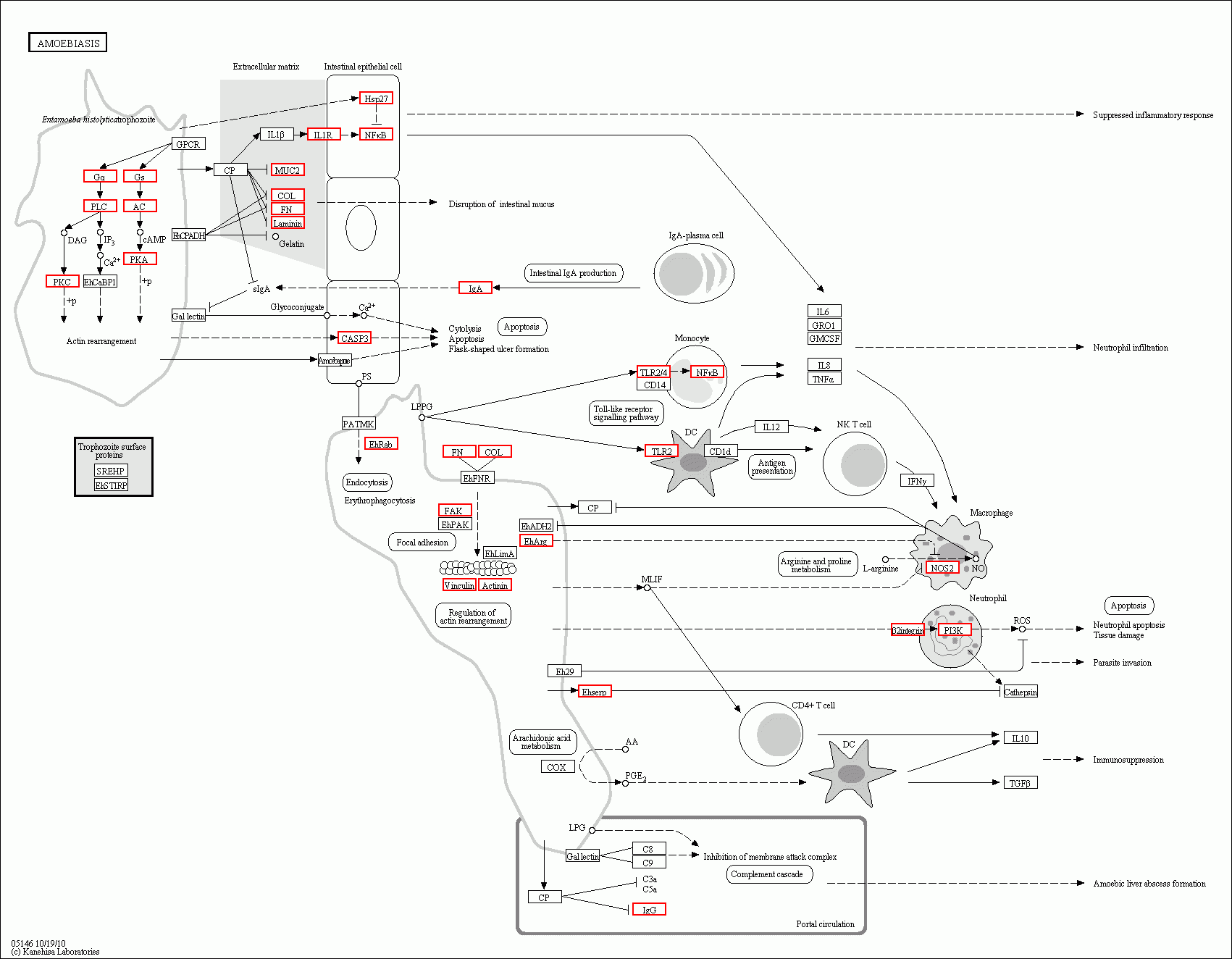

Supplement: Additional file 3: — Pathways found in the annotated portion of the transcriptomes. (ZIP 4950 kb) [file 12864_2015_1817_MOESM3_ESM.zip › map05146.png]

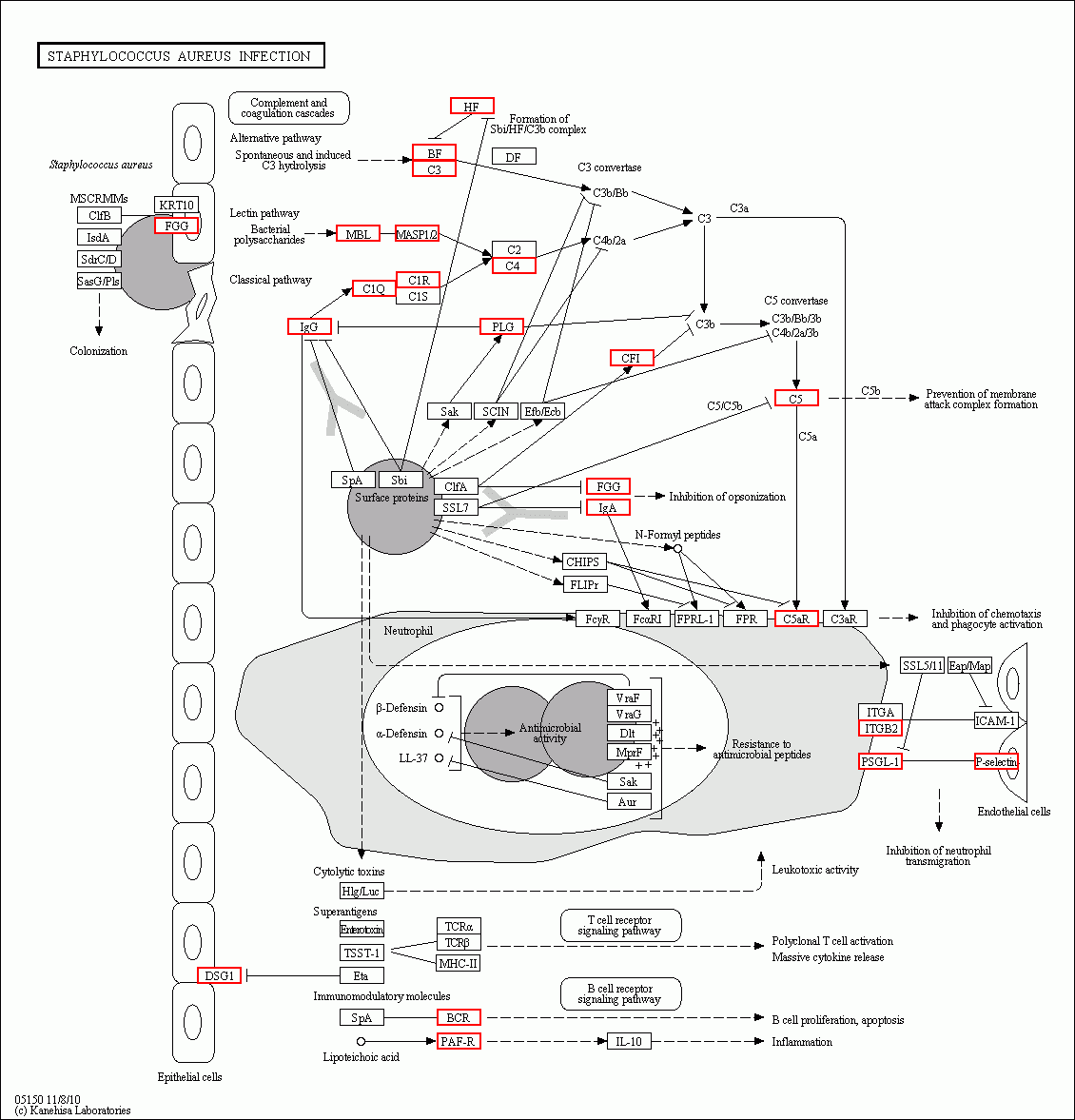

Supplement: Additional file 3: — Pathways found in the annotated portion of the transcriptomes. (ZIP 4950 kb) [file 12864_2015_1817_MOESM3_ESM.zip › map05150.png]

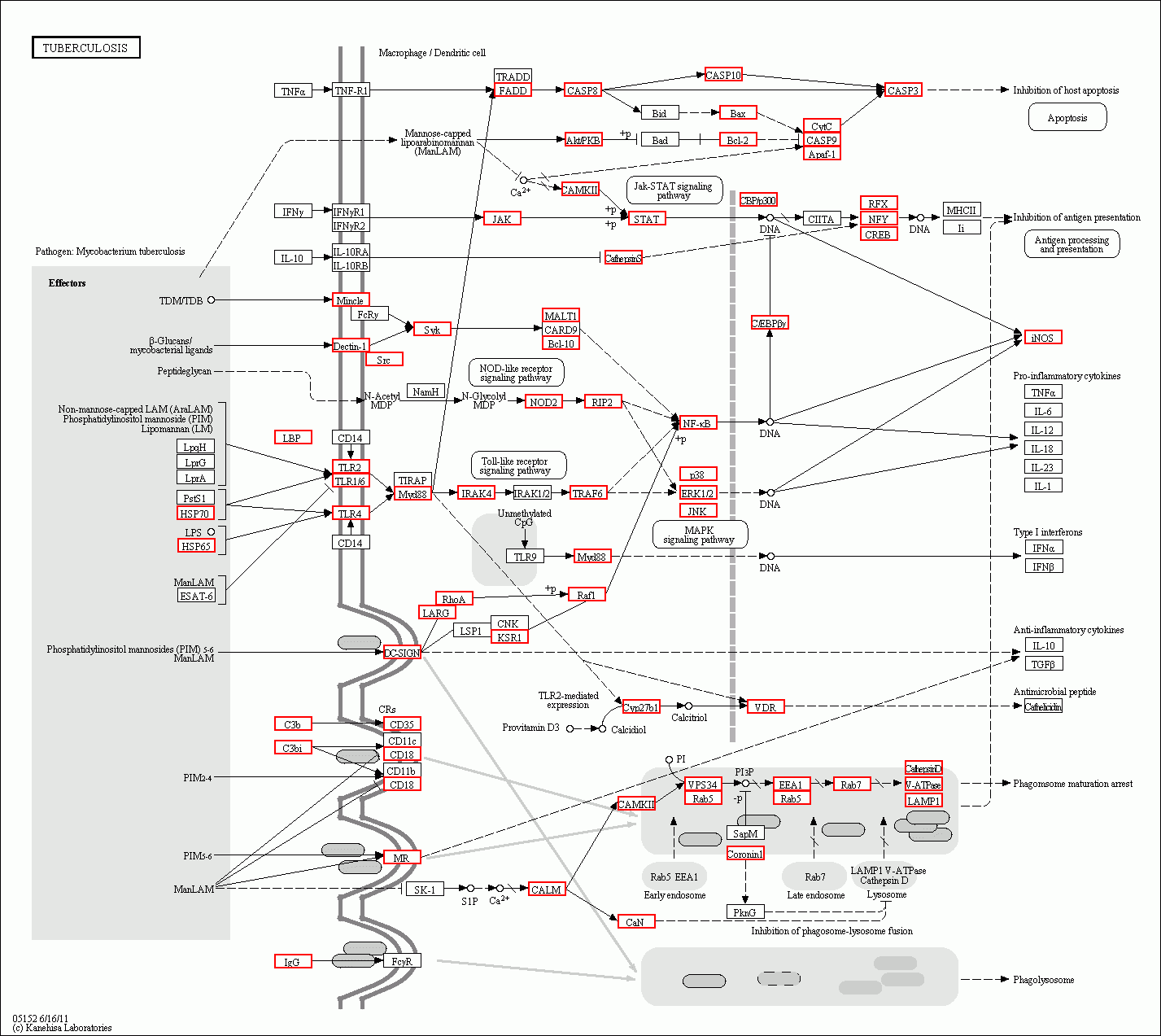

Supplement: Additional file 3: — Pathways found in the annotated portion of the transcriptomes. (ZIP 4950 kb) [file 12864_2015_1817_MOESM3_ESM.zip › map05152.png]

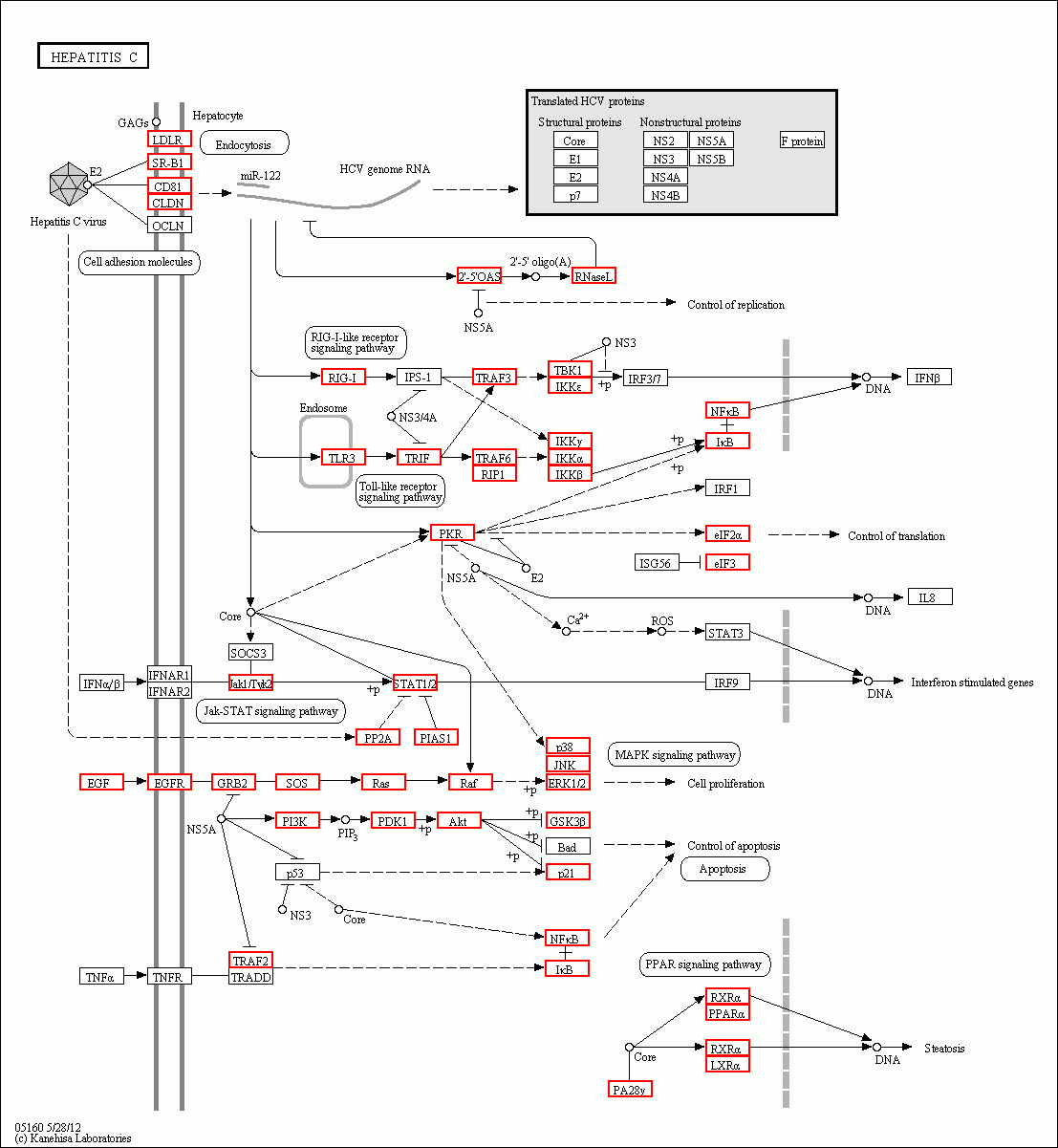

Supplement: Additional file 3: — Pathways found in the annotated portion of the transcriptomes. (ZIP 4950 kb) [file 12864_2015_1817_MOESM3_ESM.zip › map05160.png]

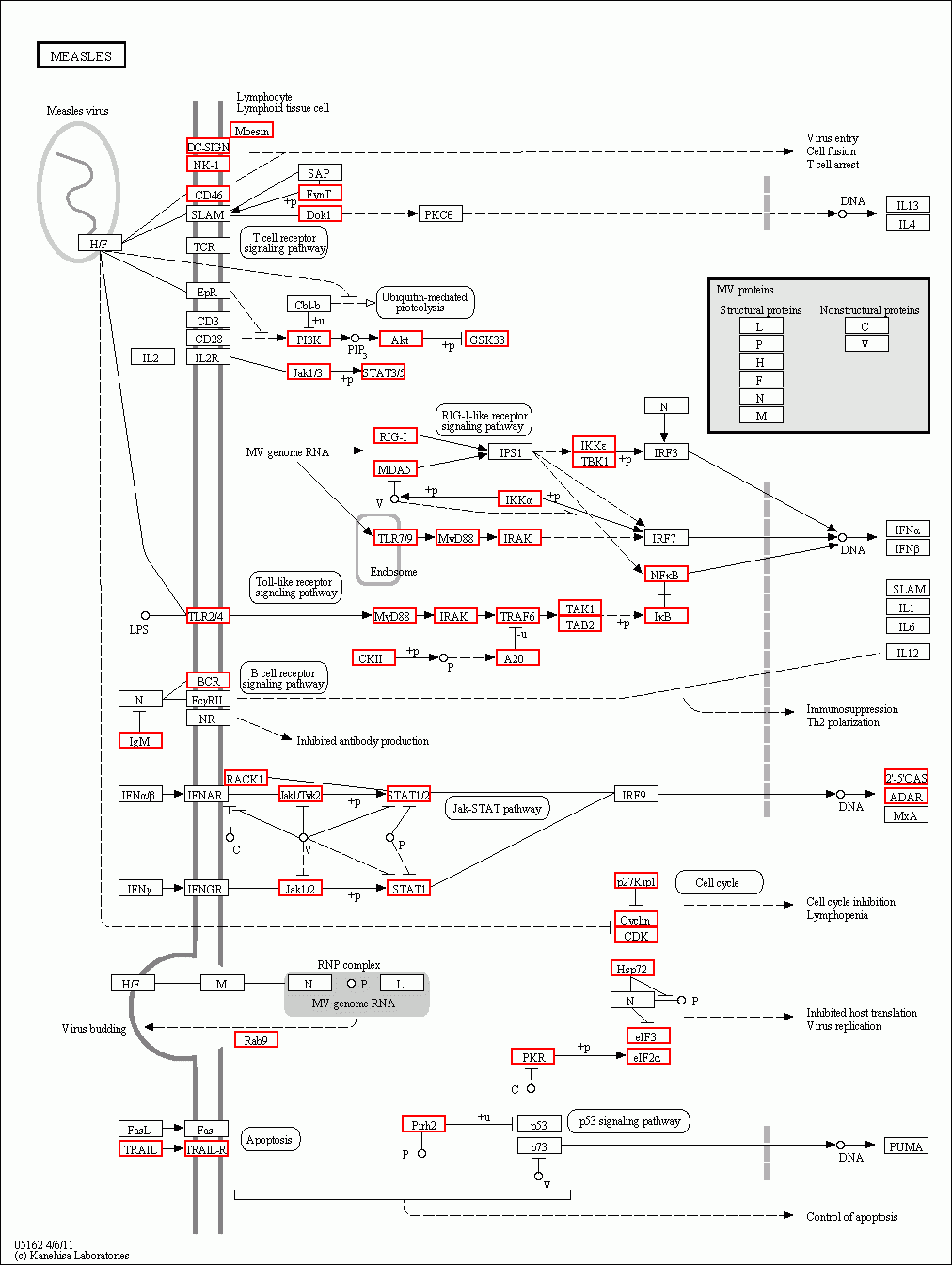

Supplement: Additional file 3: — Pathways found in the annotated portion of the transcriptomes. (ZIP 4950 kb) [file 12864_2015_1817_MOESM3_ESM.zip › map05162.png]

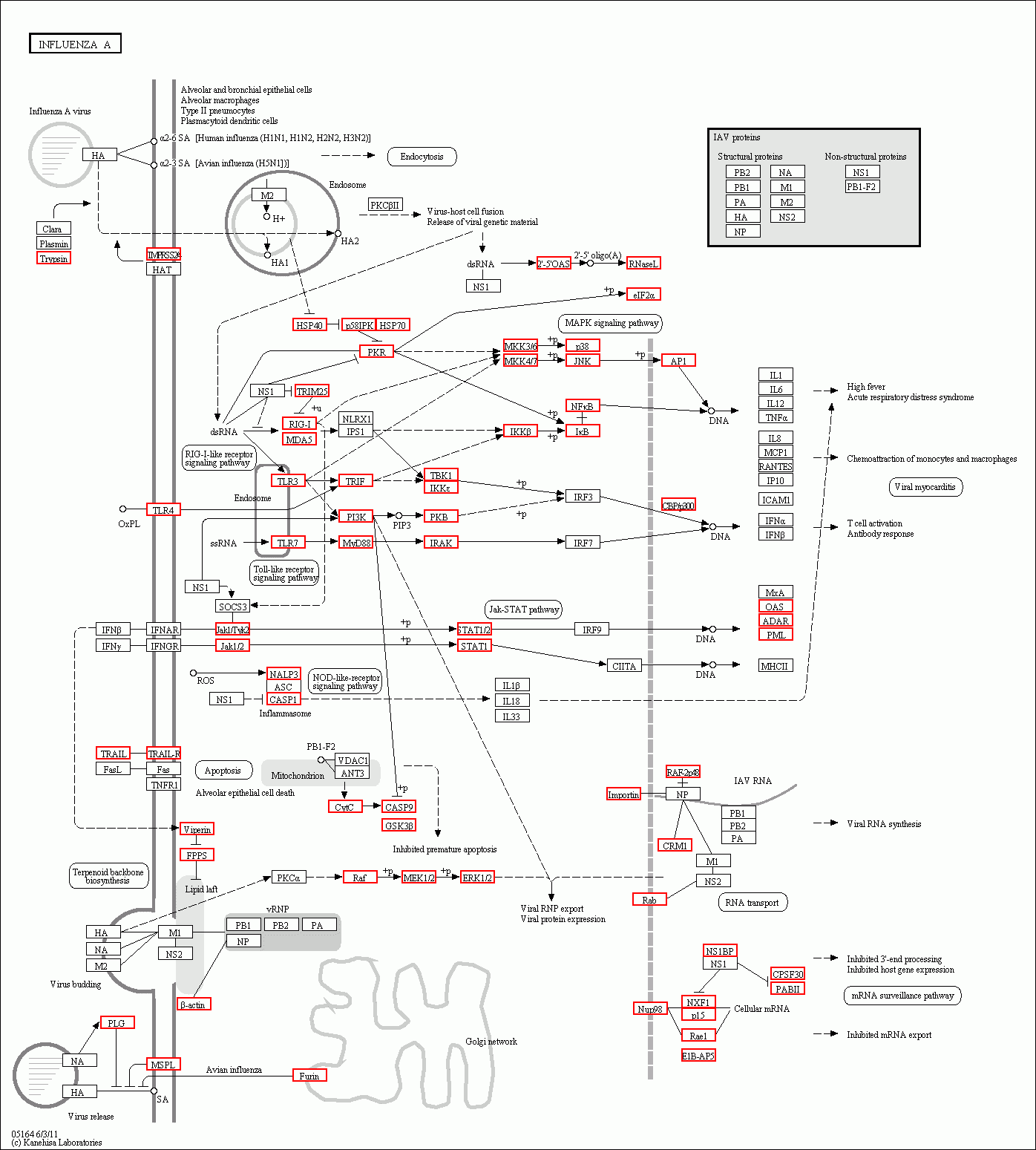

Supplement: Additional file 3: — Pathways found in the annotated portion of the transcriptomes. (ZIP 4950 kb) [file 12864_2015_1817_MOESM3_ESM.zip › map05164.png]

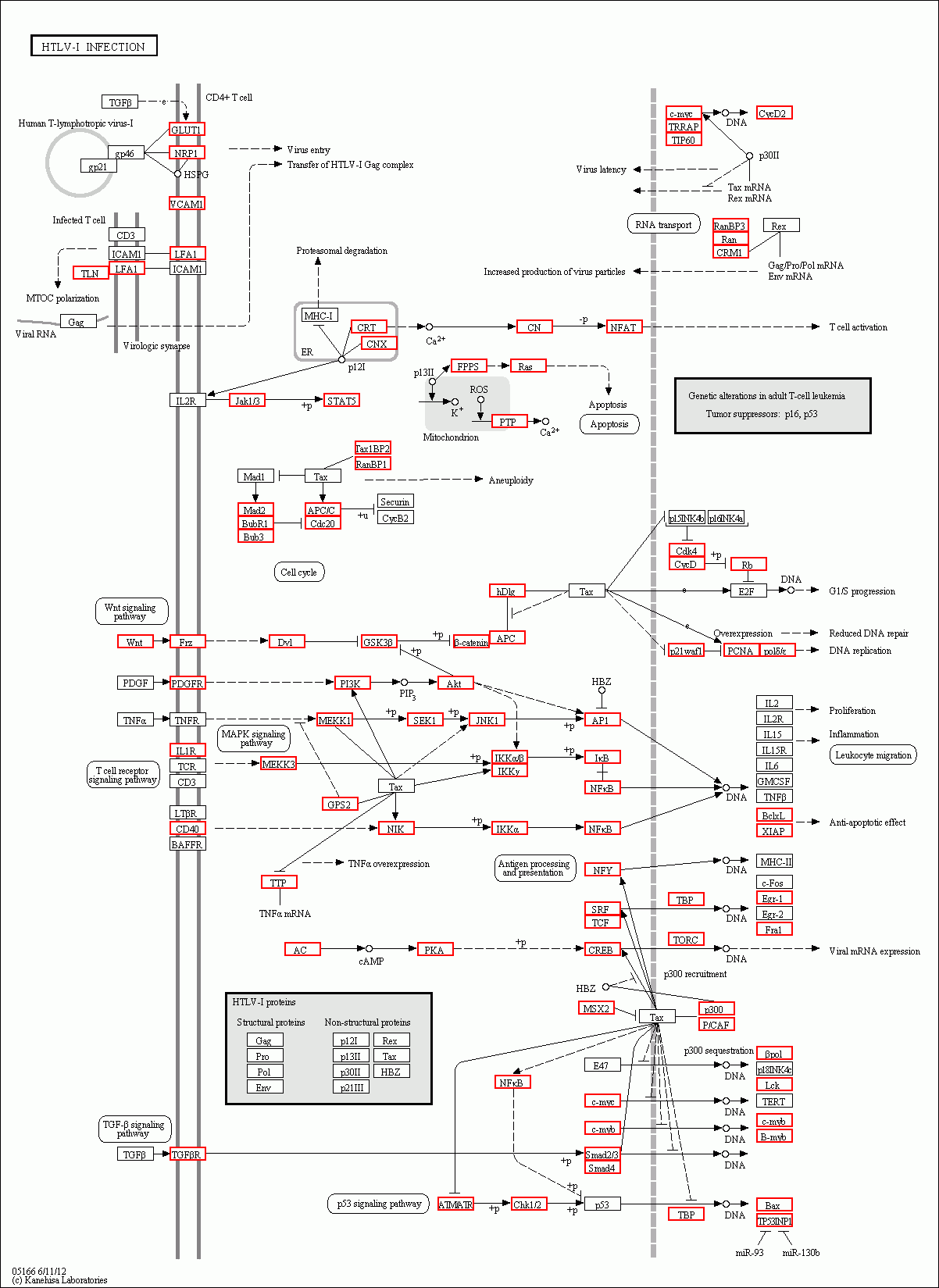

Supplement: Additional file 3: — Pathways found in the annotated portion of the transcriptomes. (ZIP 4950 kb) [file 12864_2015_1817_MOESM3_ESM.zip › map05166.png]

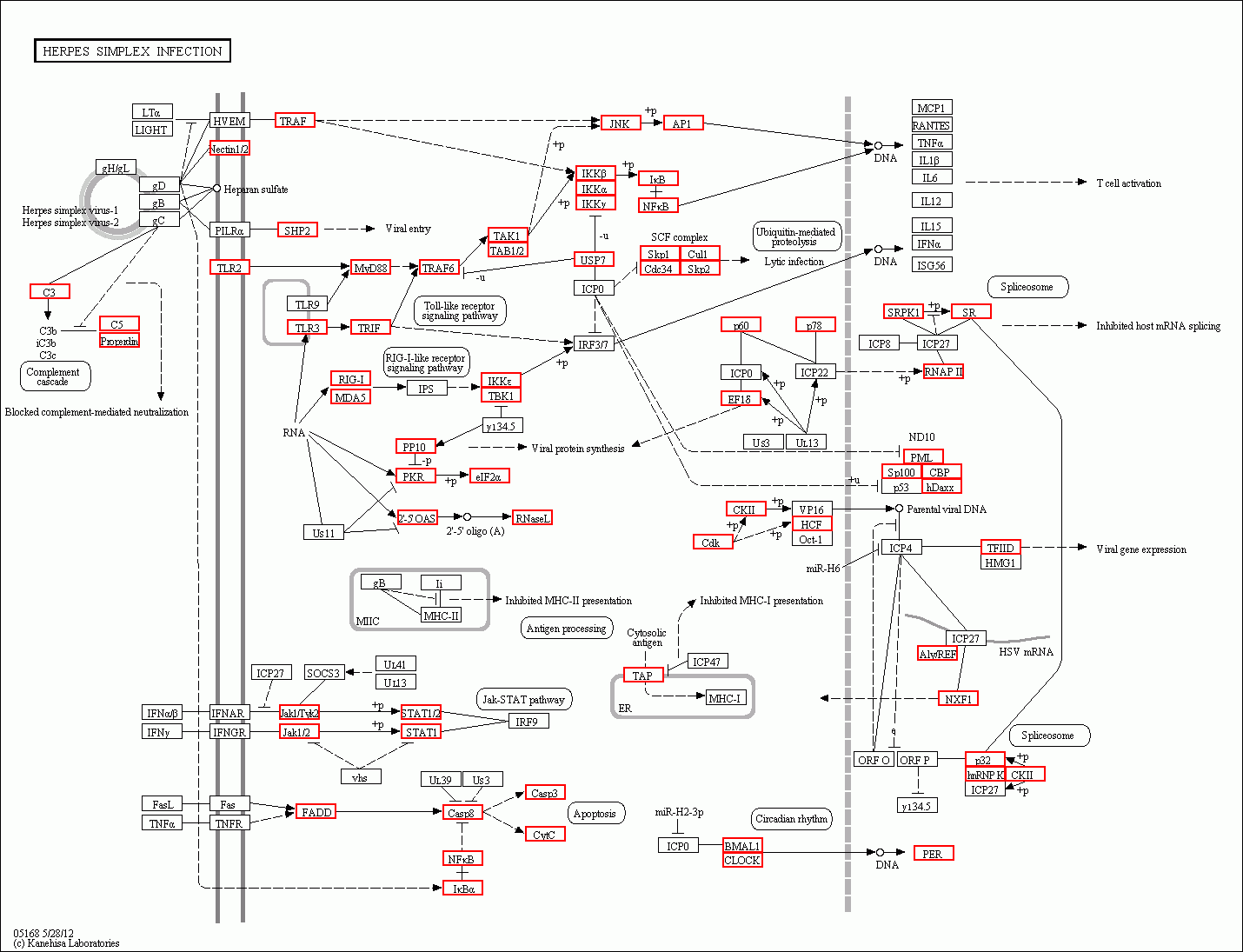

Supplement: Additional file 3: — Pathways found in the annotated portion of the transcriptomes. (ZIP 4950 kb) [file 12864_2015_1817_MOESM3_ESM.zip › map05168.png]

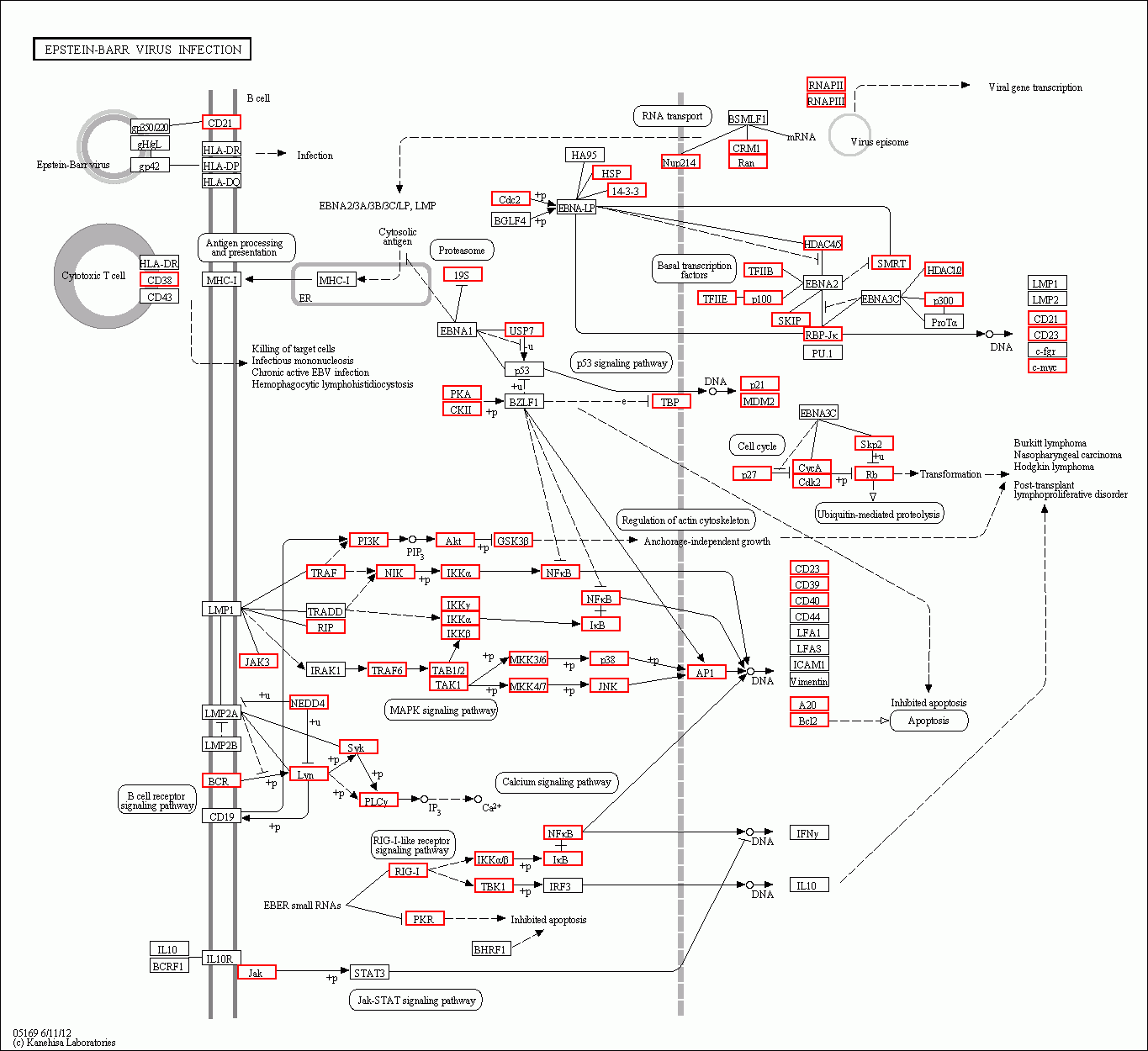

Supplement: Additional file 3: — Pathways found in the annotated portion of the transcriptomes. (ZIP 4950 kb) [file 12864_2015_1817_MOESM3_ESM.zip › map05169.png]

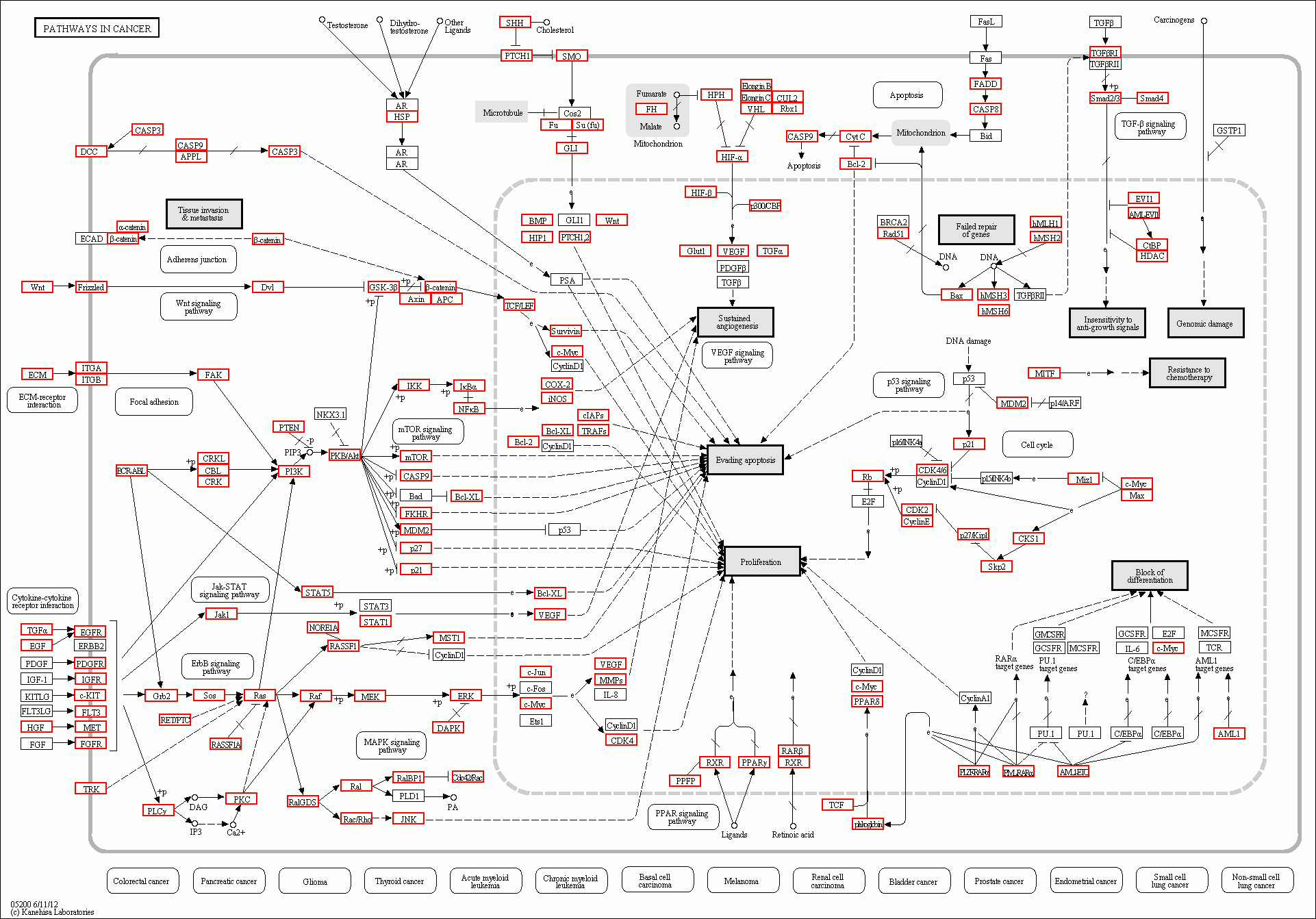

Supplement: Additional file 3: — Pathways found in the annotated portion of the transcriptomes. (ZIP 4950 kb) [file 12864_2015_1817_MOESM3_ESM.zip › map05200.png]

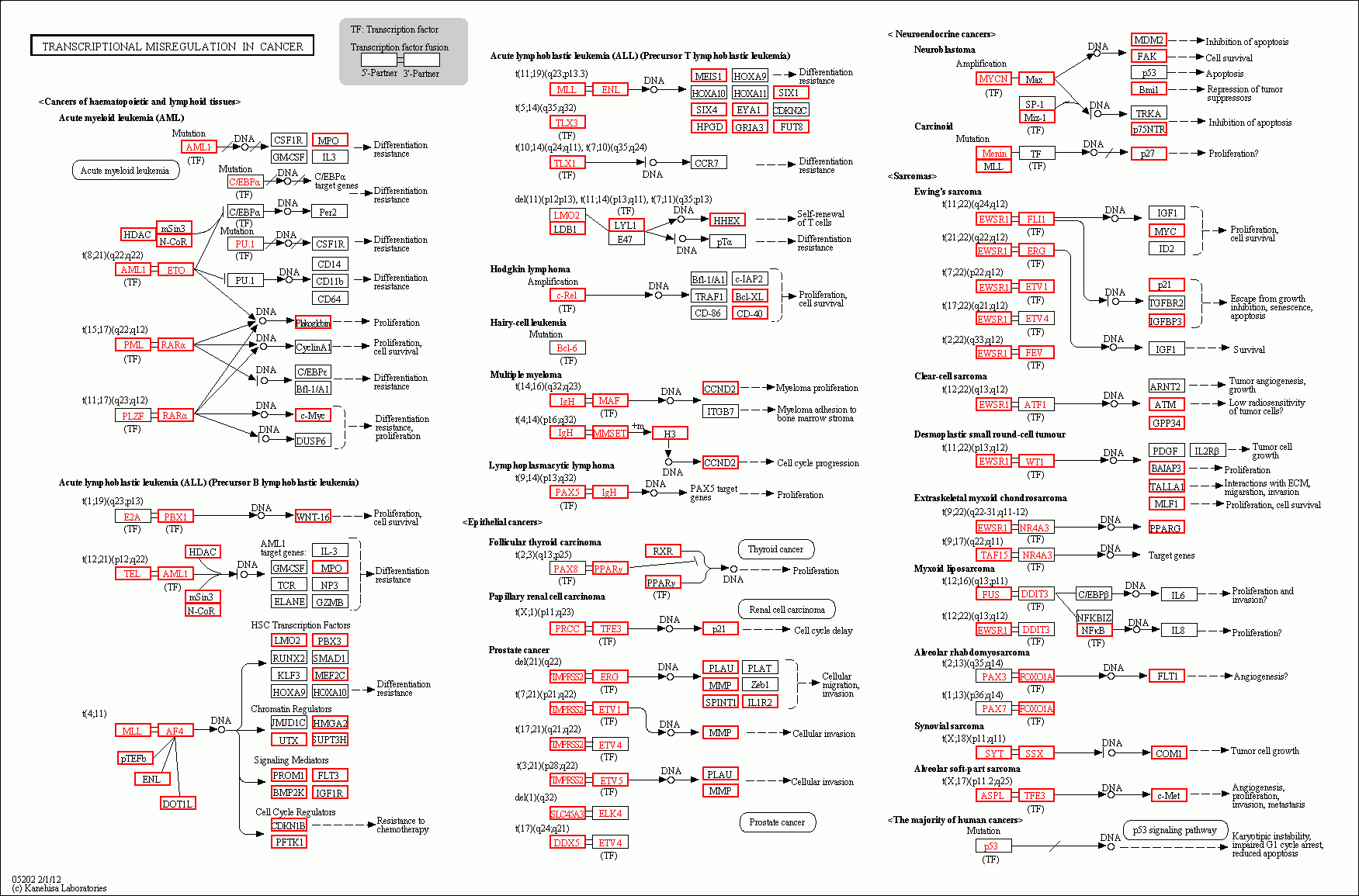

Supplement: Additional file 3: — Pathways found in the annotated portion of the transcriptomes. (ZIP 4950 kb) [file 12864_2015_1817_MOESM3_ESM.zip › map05202.png]

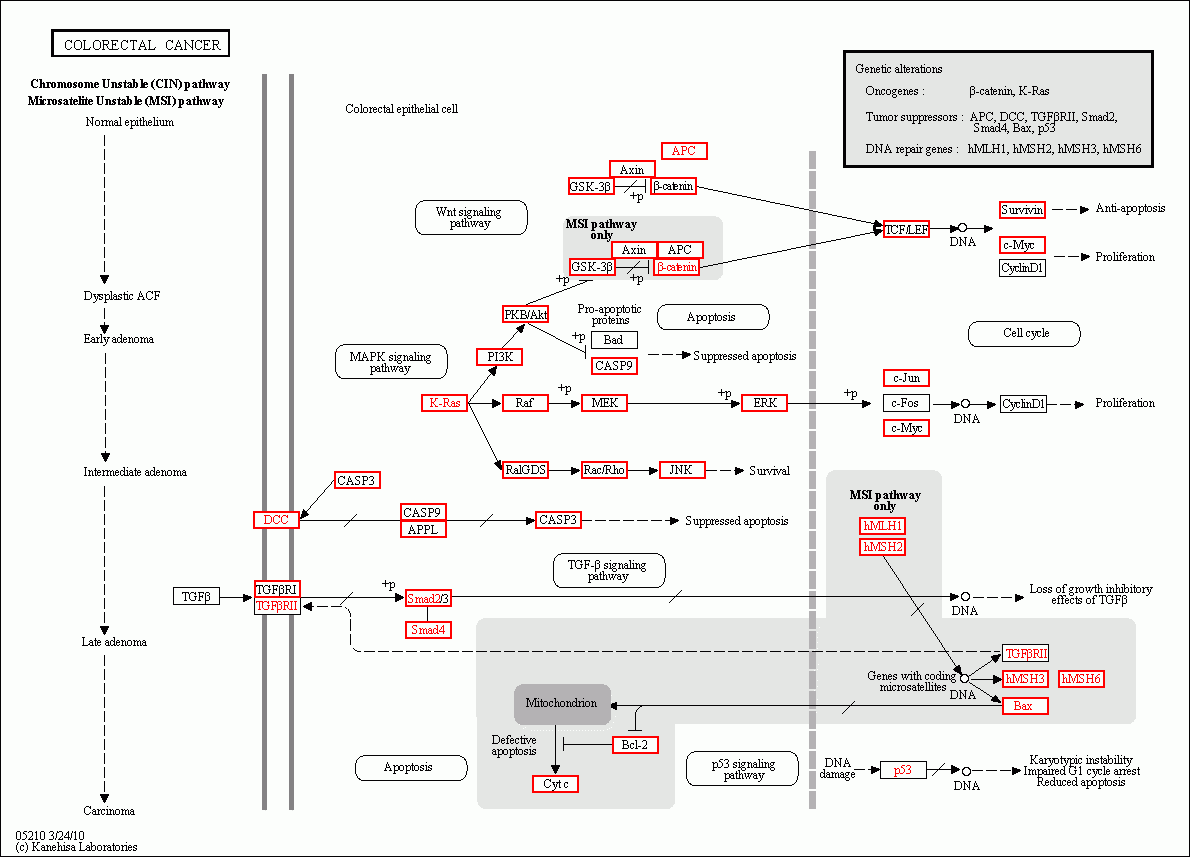

Supplement: Additional file 3: — Pathways found in the annotated portion of the transcriptomes. (ZIP 4950 kb) [file 12864_2015_1817_MOESM3_ESM.zip › map05210.png]

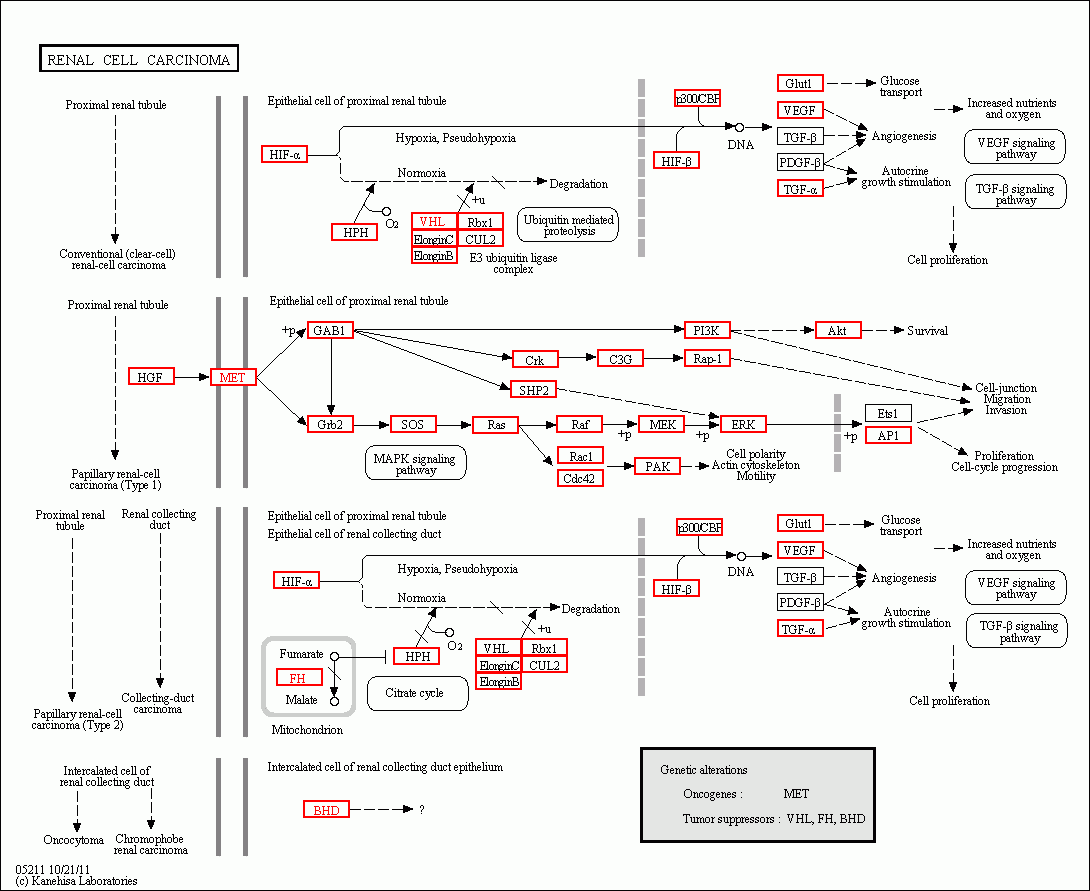

Supplement: Additional file 3: — Pathways found in the annotated portion of the transcriptomes. (ZIP 4950 kb) [file 12864_2015_1817_MOESM3_ESM.zip › map05211.png]

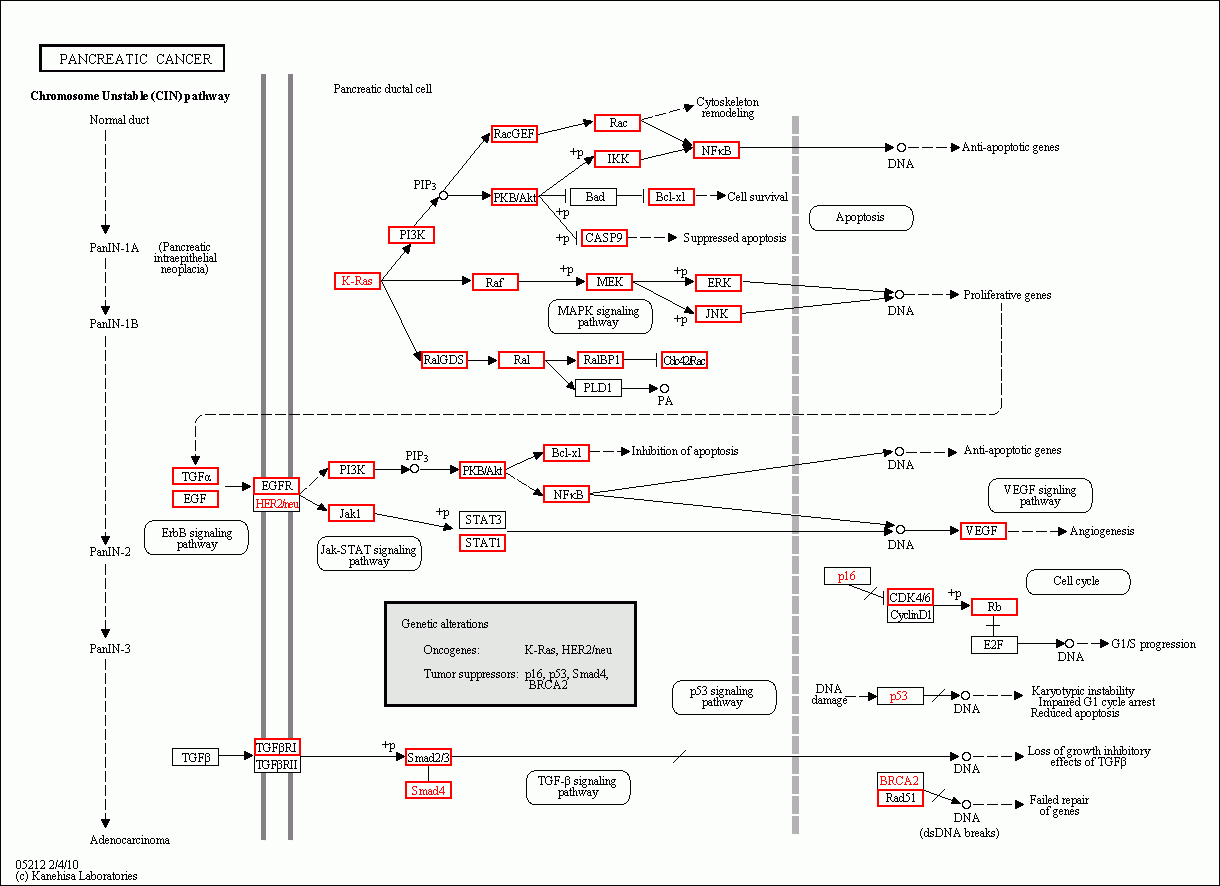

Supplement: Additional file 3: — Pathways found in the annotated portion of the transcriptomes. (ZIP 4950 kb) [file 12864_2015_1817_MOESM3_ESM.zip › map05212.png]

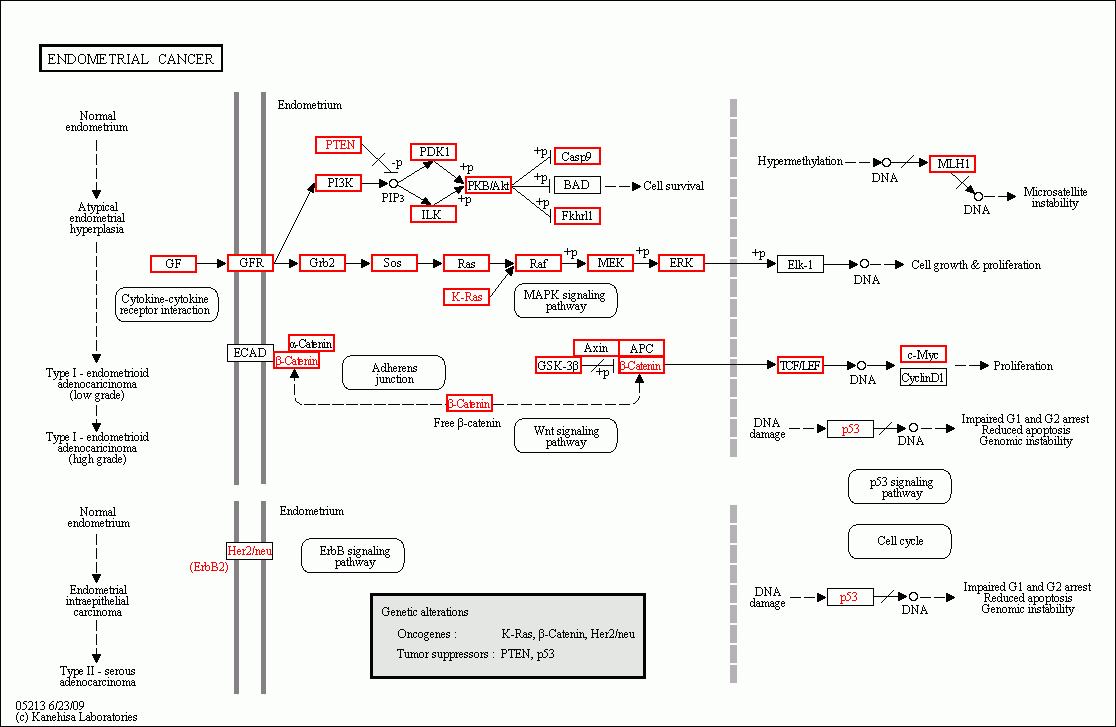

Supplement: Additional file 3: — Pathways found in the annotated portion of the transcriptomes. (ZIP 4950 kb) [file 12864_2015_1817_MOESM3_ESM.zip › map05213.png]

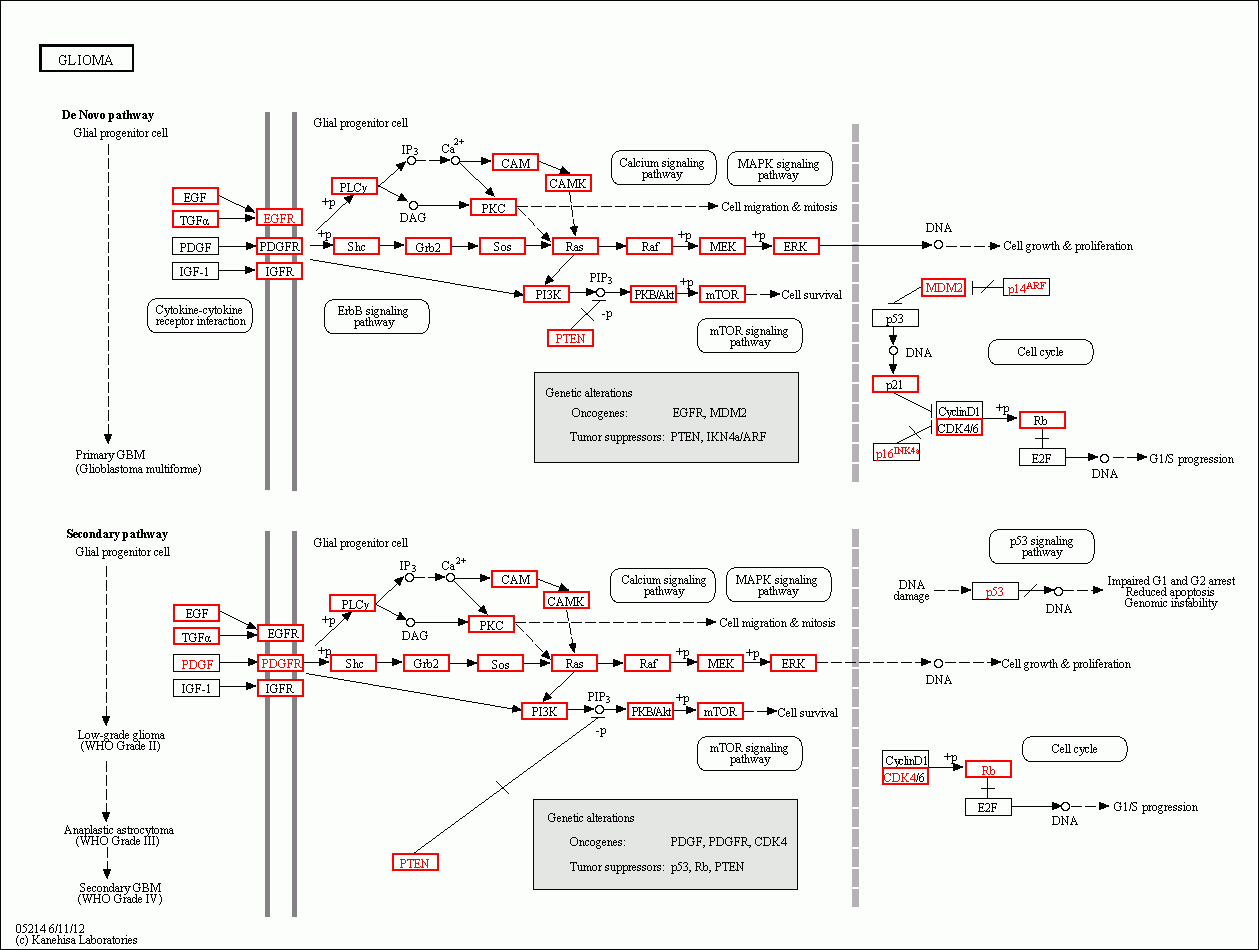

Supplement: Additional file 3: — Pathways found in the annotated portion of the transcriptomes. (ZIP 4950 kb) [file 12864_2015_1817_MOESM3_ESM.zip › map05214.png]

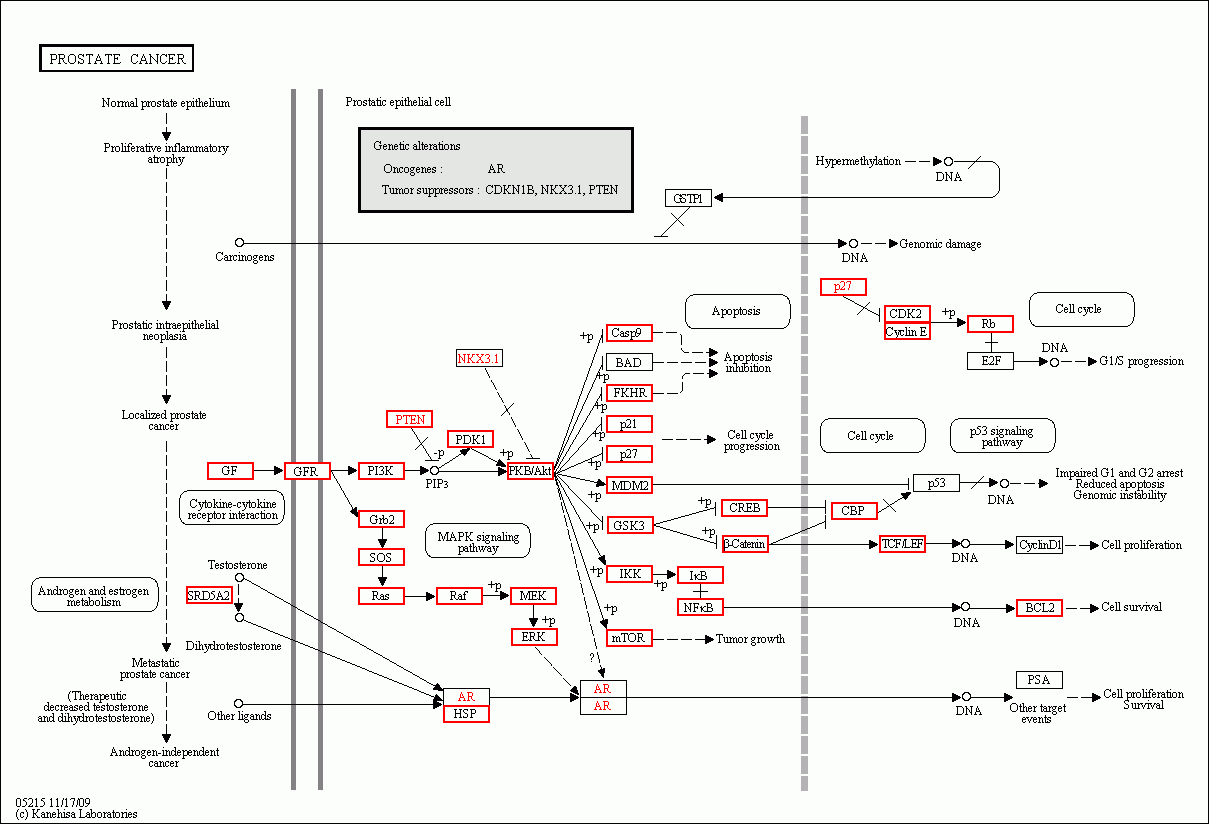

Supplement: Additional file 3: — Pathways found in the annotated portion of the transcriptomes. (ZIP 4950 kb) [file 12864_2015_1817_MOESM3_ESM.zip › map05215.png]

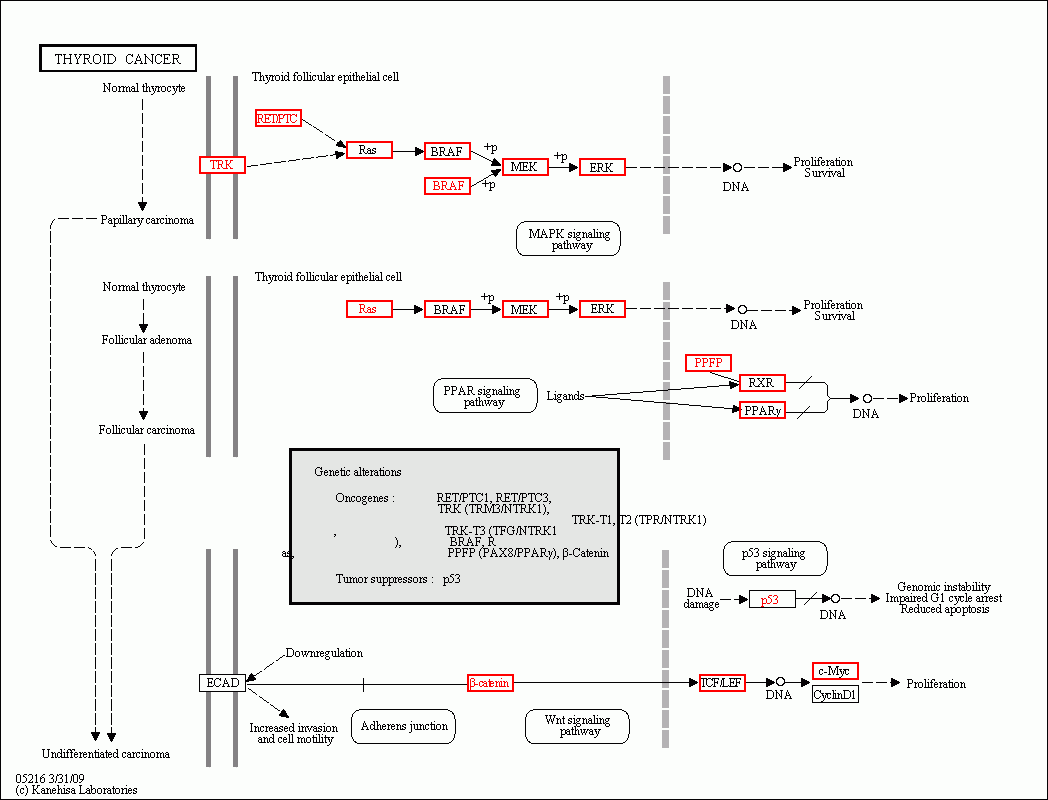

Supplement: Additional file 3: — Pathways found in the annotated portion of the transcriptomes. (ZIP 4950 kb) [file 12864_2015_1817_MOESM3_ESM.zip › map05216.png]

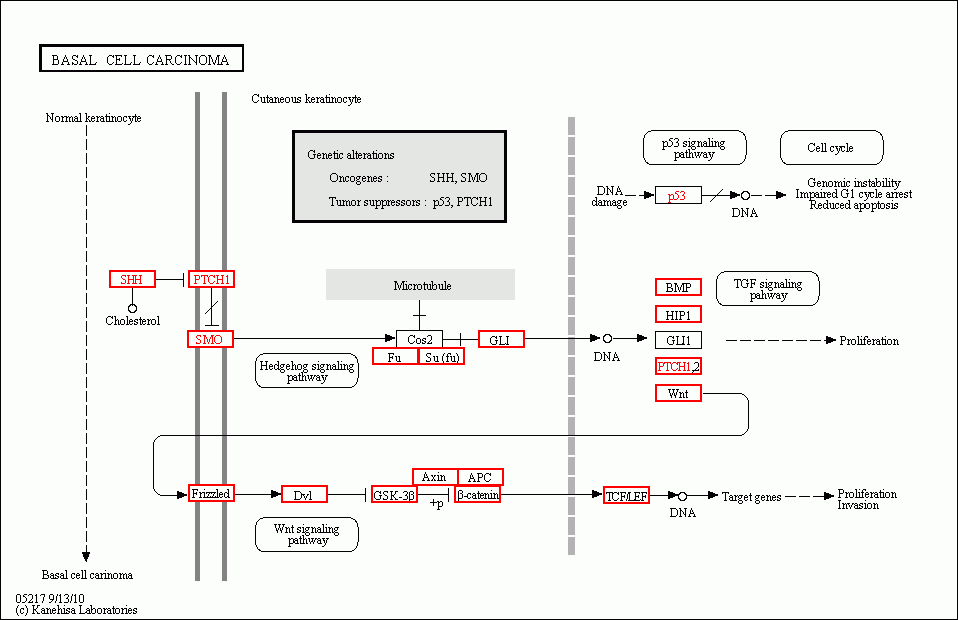

Supplement: Additional file 3: — Pathways found in the annotated portion of the transcriptomes. (ZIP 4950 kb) [file 12864_2015_1817_MOESM3_ESM.zip › map05217.png]

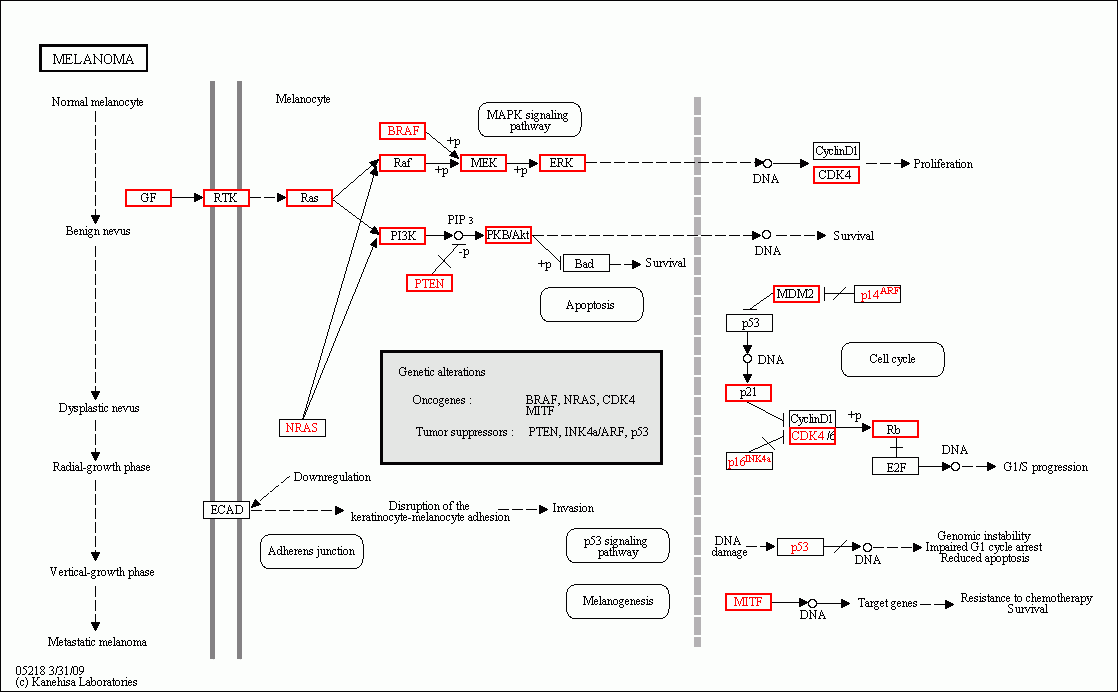

Supplement: Additional file 3: — Pathways found in the annotated portion of the transcriptomes. (ZIP 4950 kb) [file 12864_2015_1817_MOESM3_ESM.zip › map05218.png]

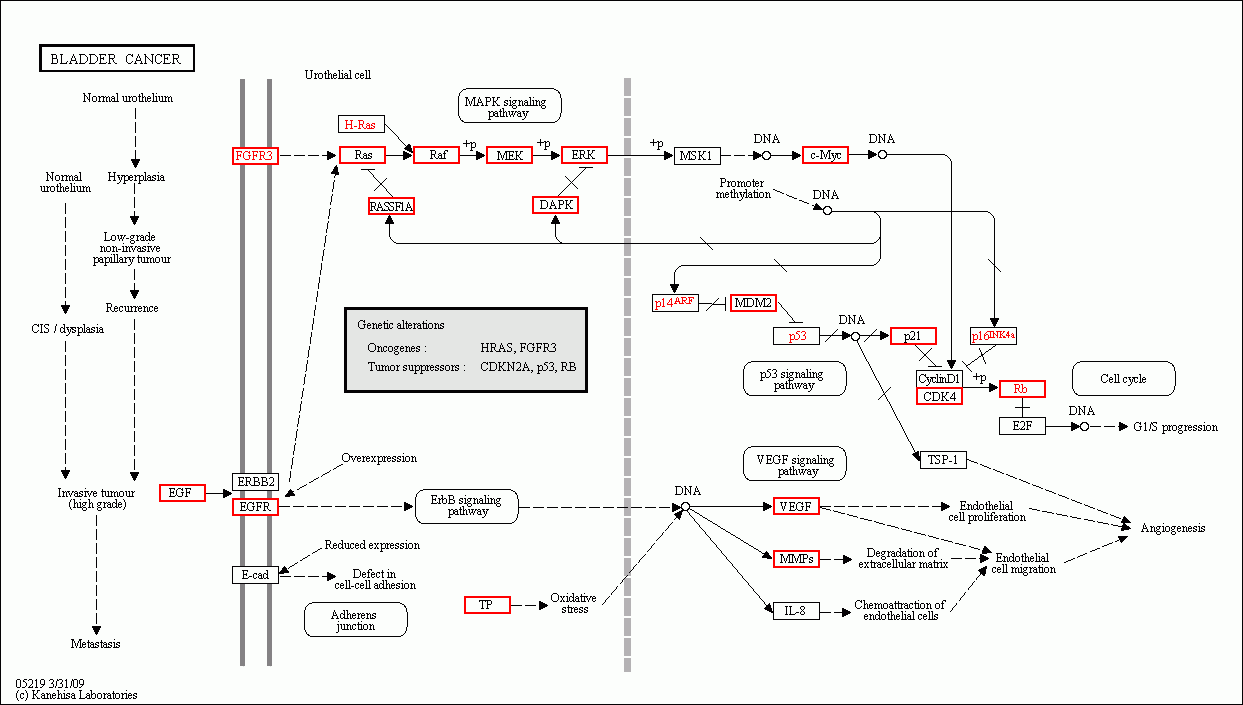

Supplement: Additional file 3: — Pathways found in the annotated portion of the transcriptomes. (ZIP 4950 kb) [file 12864_2015_1817_MOESM3_ESM.zip › map05219.png]

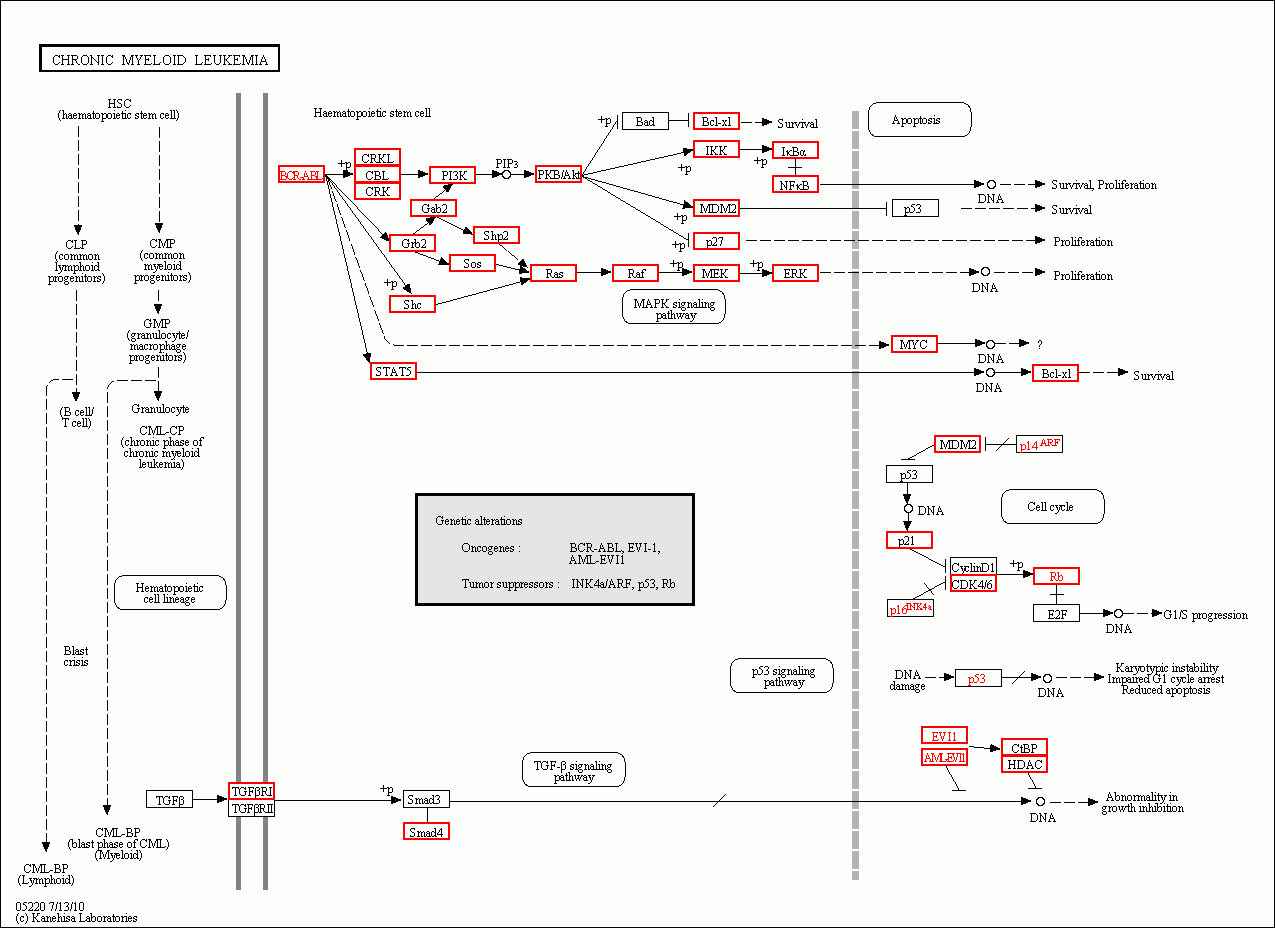

Supplement: Additional file 3: — Pathways found in the annotated portion of the transcriptomes. (ZIP 4950 kb) [file 12864_2015_1817_MOESM3_ESM.zip › map05220.png]

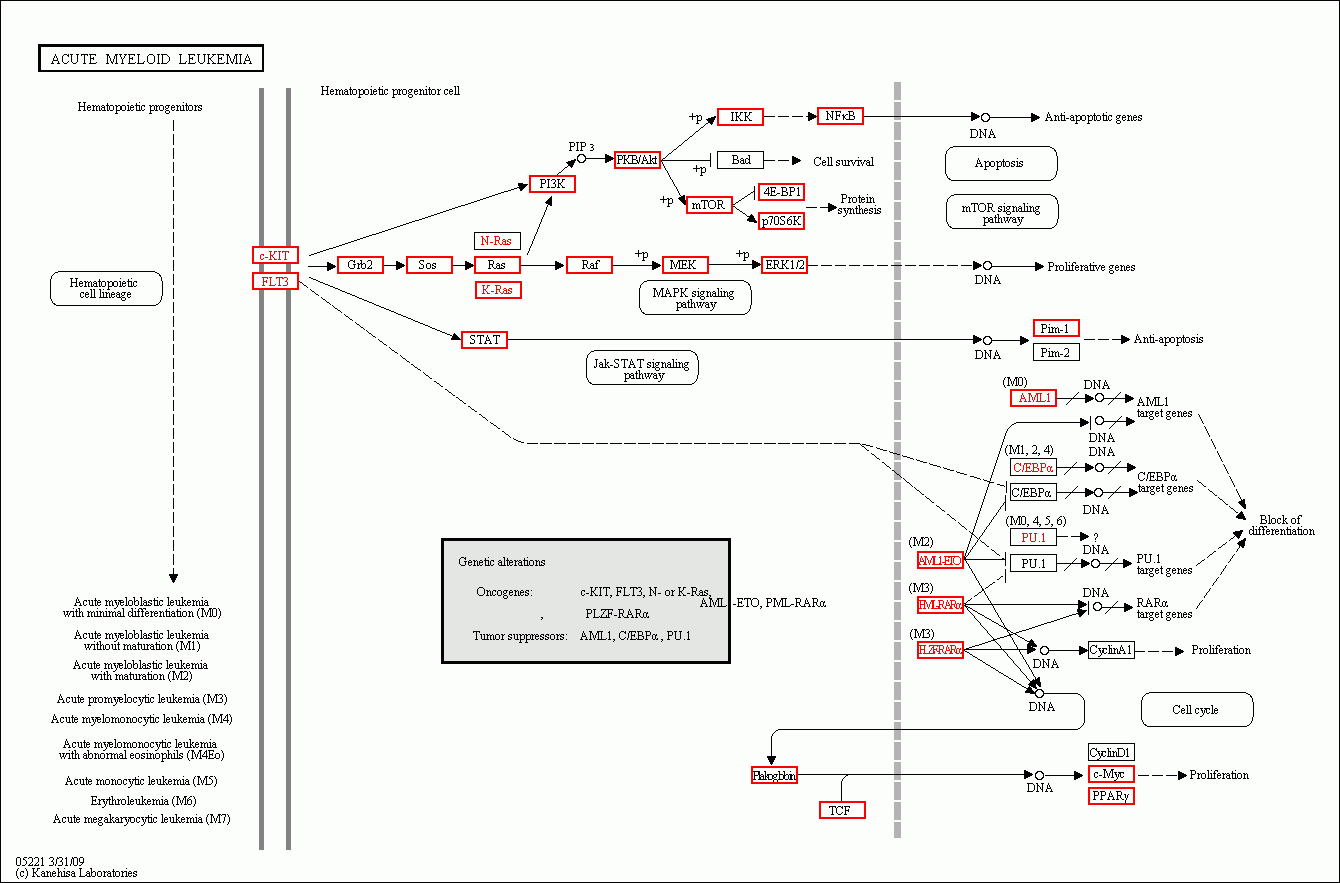

Supplement: Additional file 3: — Pathways found in the annotated portion of the transcriptomes. (ZIP 4950 kb) [file 12864_2015_1817_MOESM3_ESM.zip › map05221.png]

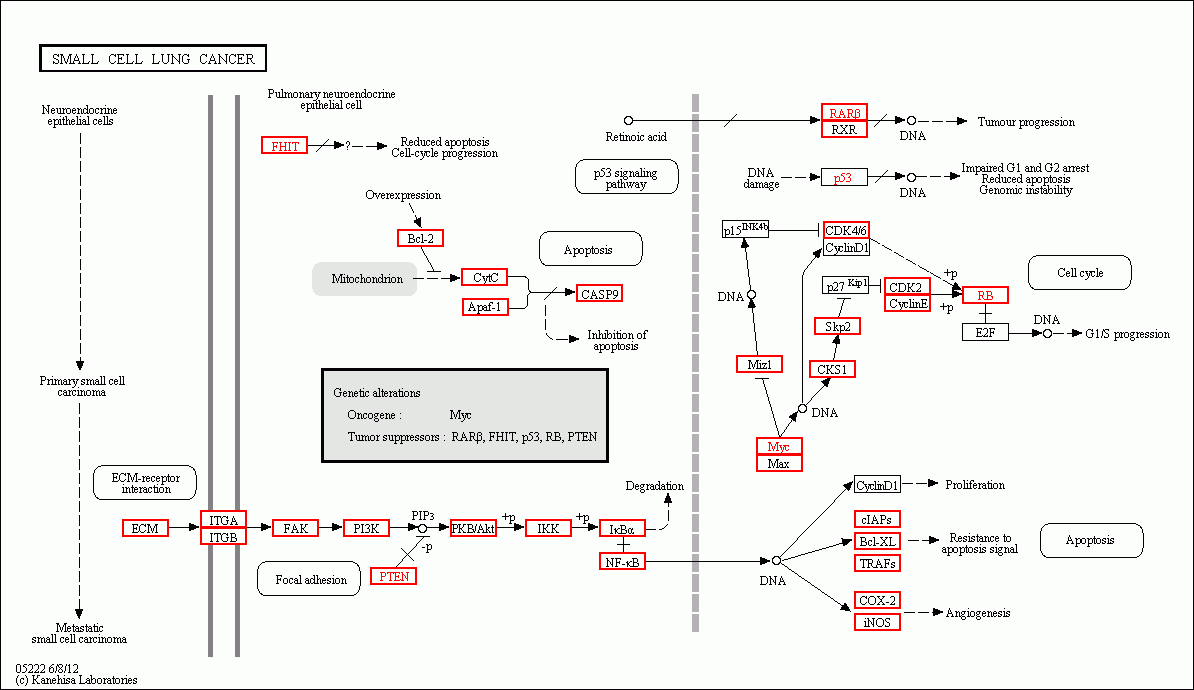

Supplement: Additional file 3: — Pathways found in the annotated portion of the transcriptomes. (ZIP 4950 kb) [file 12864_2015_1817_MOESM3_ESM.zip › map05222.png]

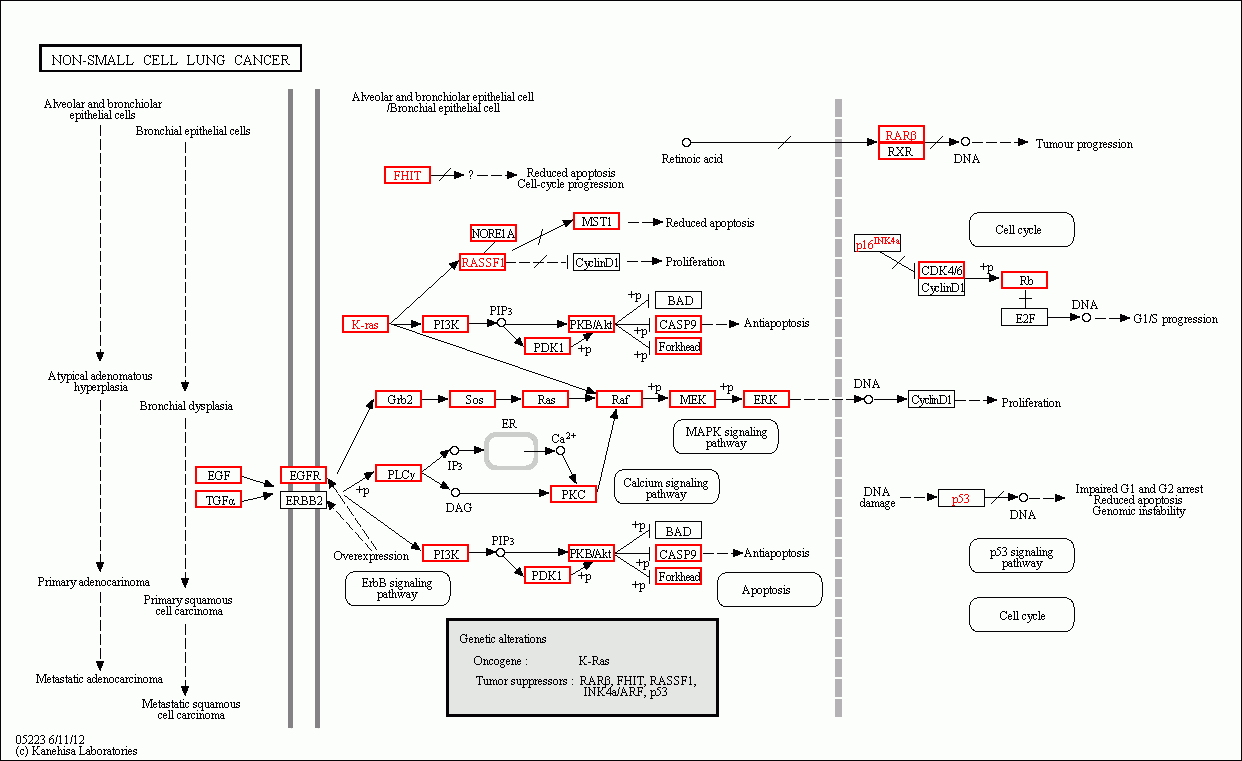

Supplement: Additional file 3: — Pathways found in the annotated portion of the transcriptomes. (ZIP 4950 kb) [file 12864_2015_1817_MOESM3_ESM.zip › map05223.png]

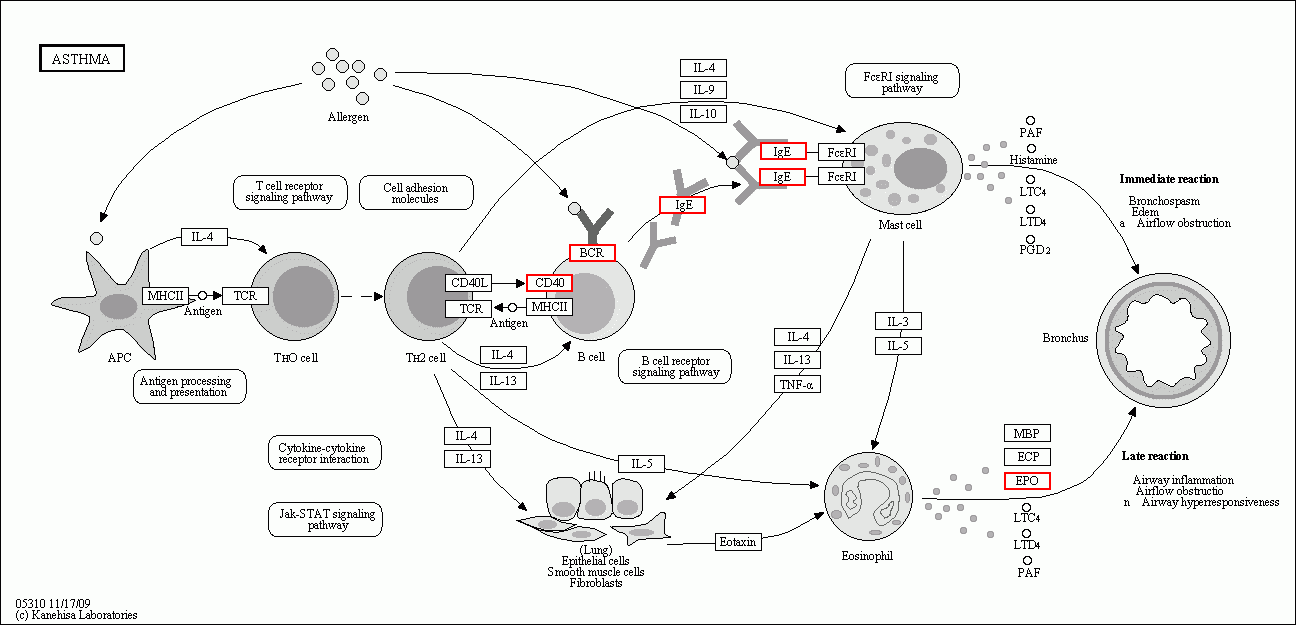

Supplement: Additional file 3: — Pathways found in the annotated portion of the transcriptomes. (ZIP 4950 kb) [file 12864_2015_1817_MOESM3_ESM.zip › map05310.png]

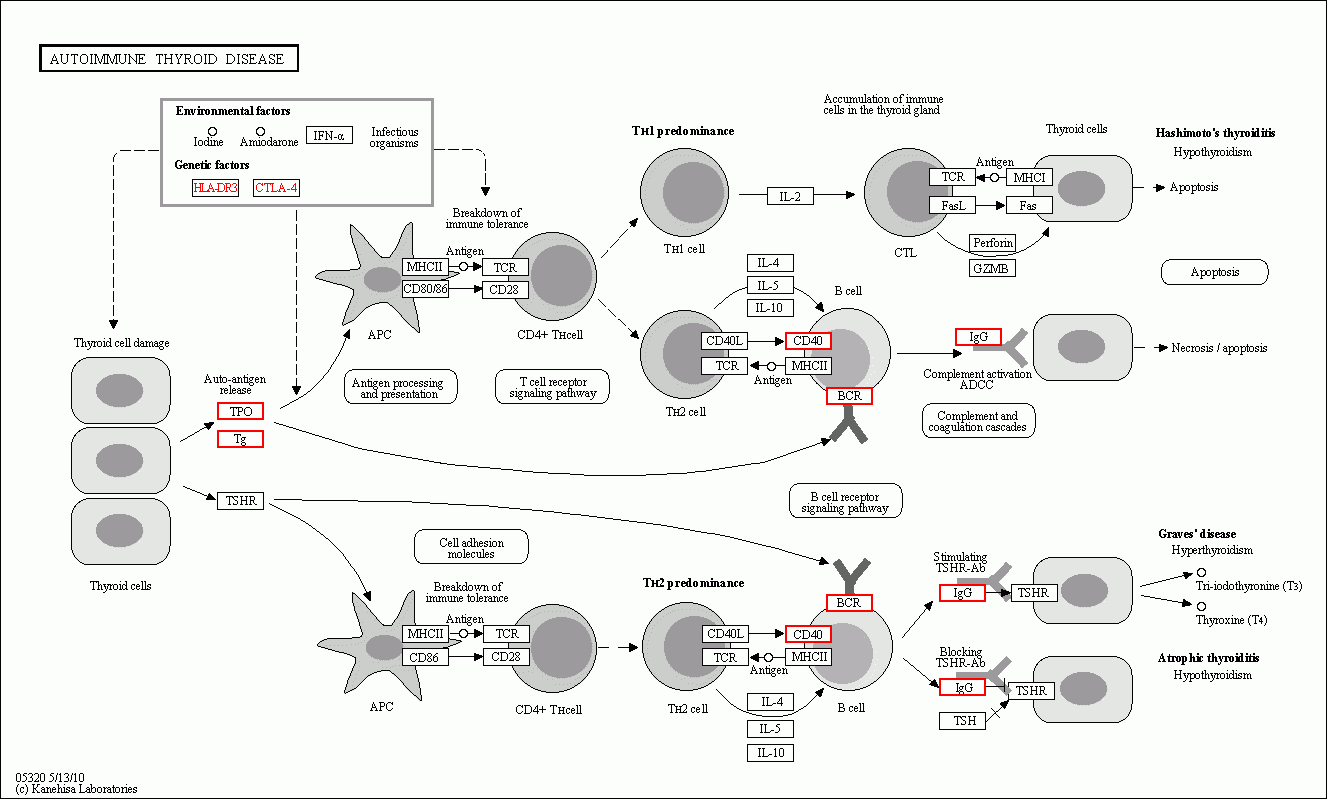

Supplement: Additional file 3: — Pathways found in the annotated portion of the transcriptomes. (ZIP 4950 kb) [file 12864_2015_1817_MOESM3_ESM.zip › map05320.png]

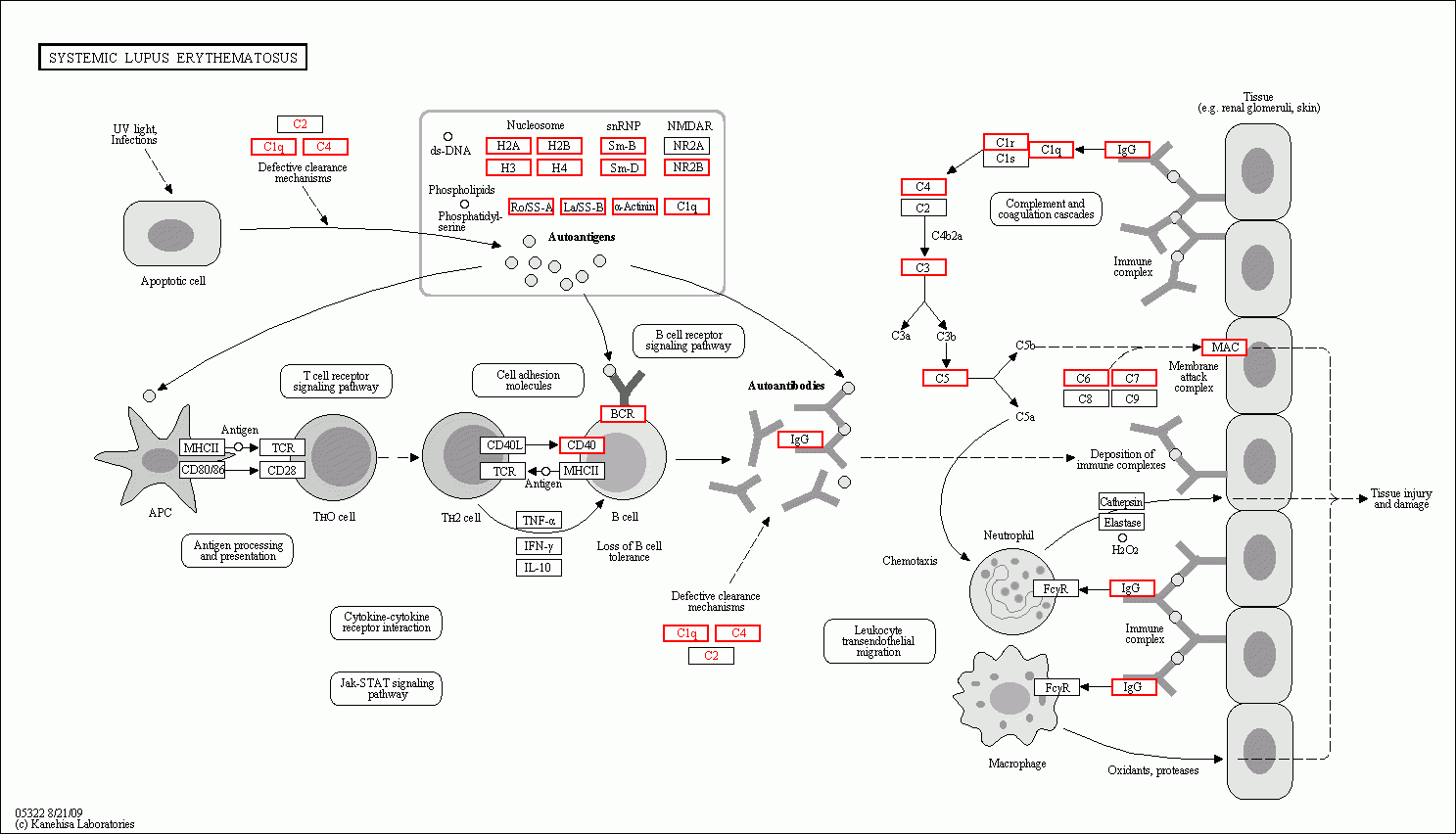

Supplement: Additional file 3: — Pathways found in the annotated portion of the transcriptomes. (ZIP 4950 kb) [file 12864_2015_1817_MOESM3_ESM.zip › map05322.png]

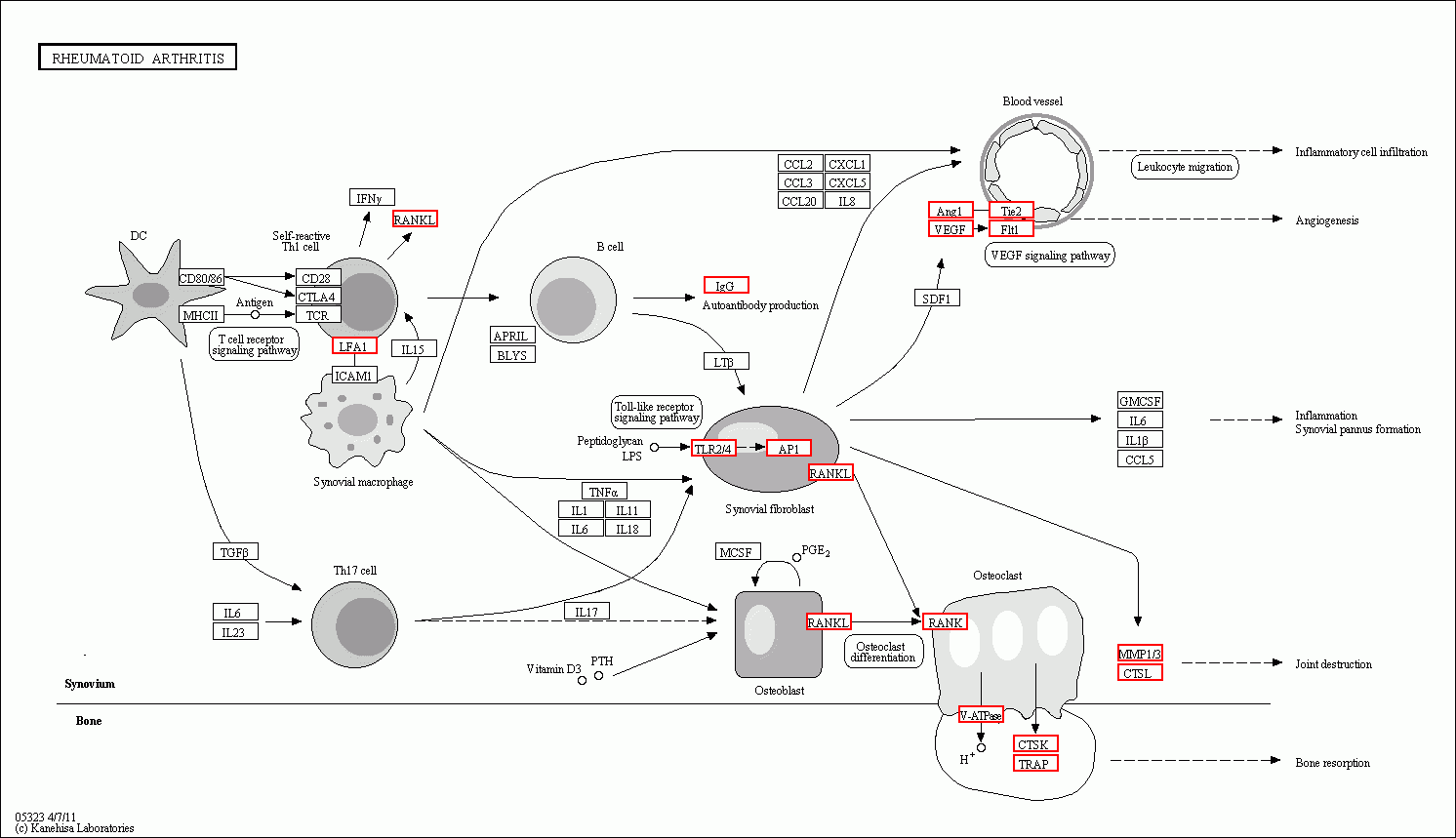

Supplement: Additional file 3: — Pathways found in the annotated portion of the transcriptomes. (ZIP 4950 kb) [file 12864_2015_1817_MOESM3_ESM.zip › map05323.png]

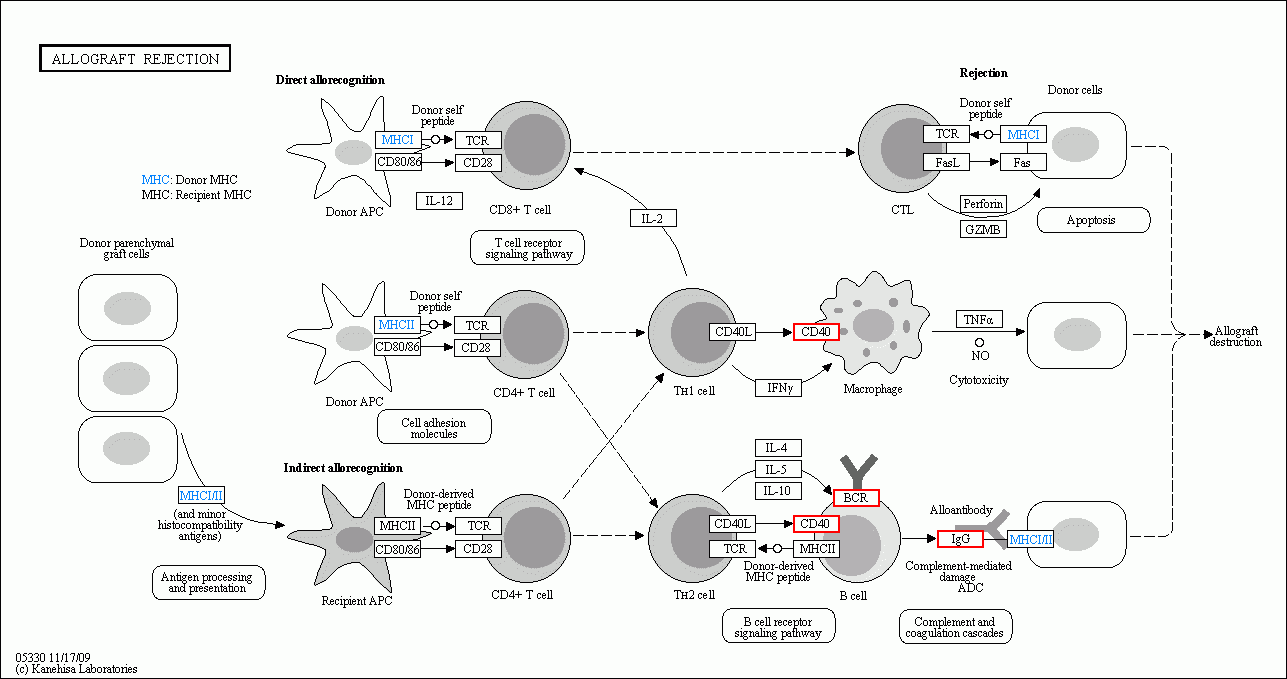

Supplement: Additional file 3: — Pathways found in the annotated portion of the transcriptomes. (ZIP 4950 kb) [file 12864_2015_1817_MOESM3_ESM.zip › map05330.png]

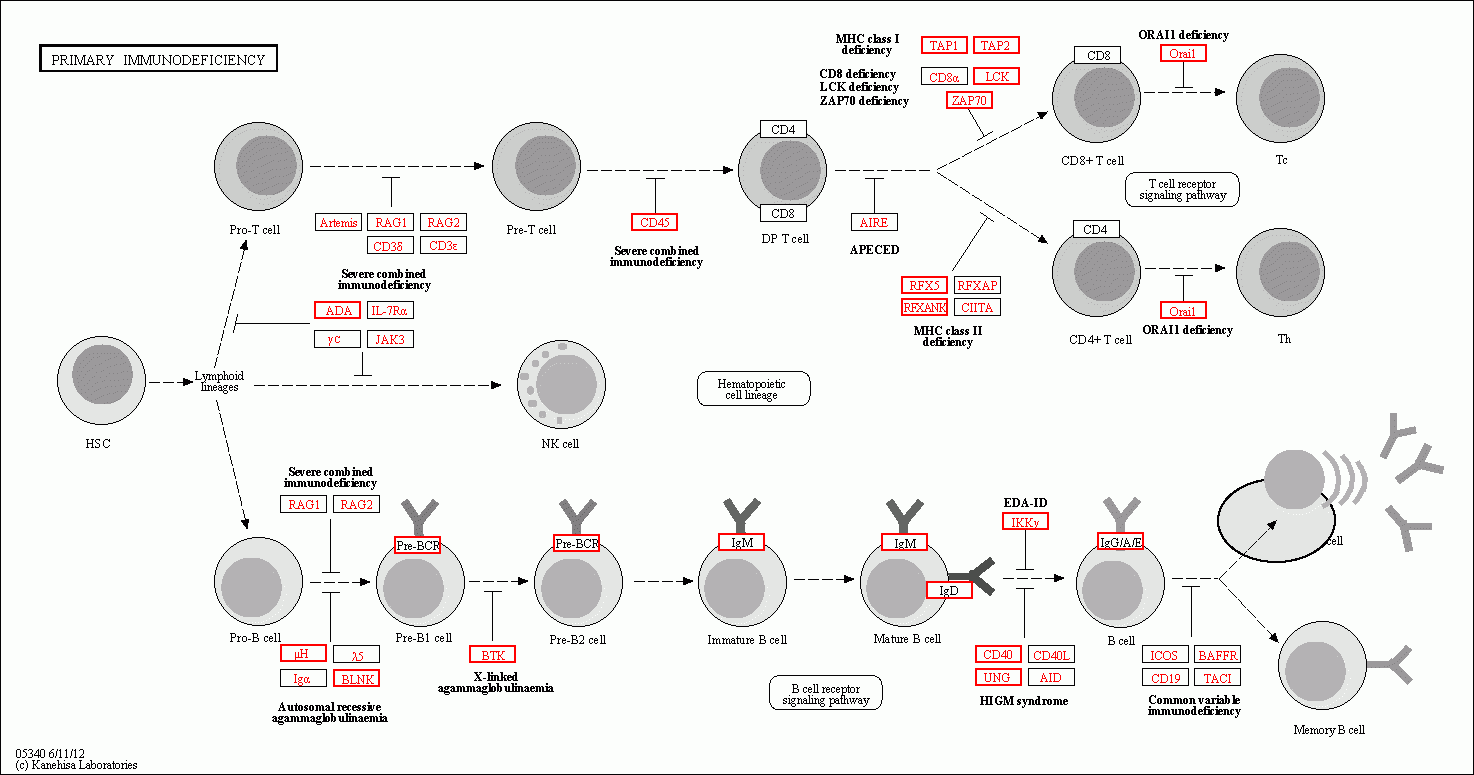

Supplement: Additional file 3: — Pathways found in the annotated portion of the transcriptomes. (ZIP 4950 kb) [file 12864_2015_1817_MOESM3_ESM.zip › map05340.png]

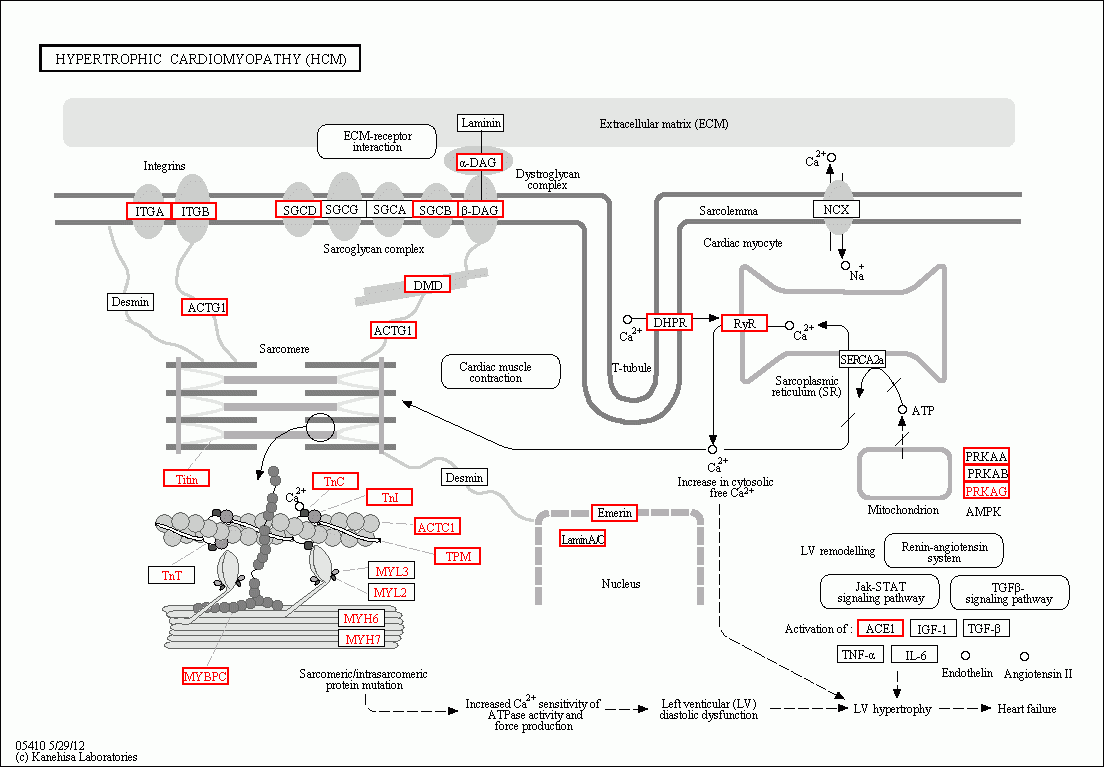

Supplement: Additional file 3: — Pathways found in the annotated portion of the transcriptomes. (ZIP 4950 kb) [file 12864_2015_1817_MOESM3_ESM.zip › map05410.png]
