# Supplementary figures and images for: RNA-Seq in Mytilus galloprovincialis: comparative transcriptomics and expression profiles among different tissues (part 1 of 2)
Source: BMC Genomics. 2015 Sep 24;16(1):728. doi: 10.1186/s12864-015-1817-5 (PMC4581086; doi:10.1186/s12864-015-1817-5)

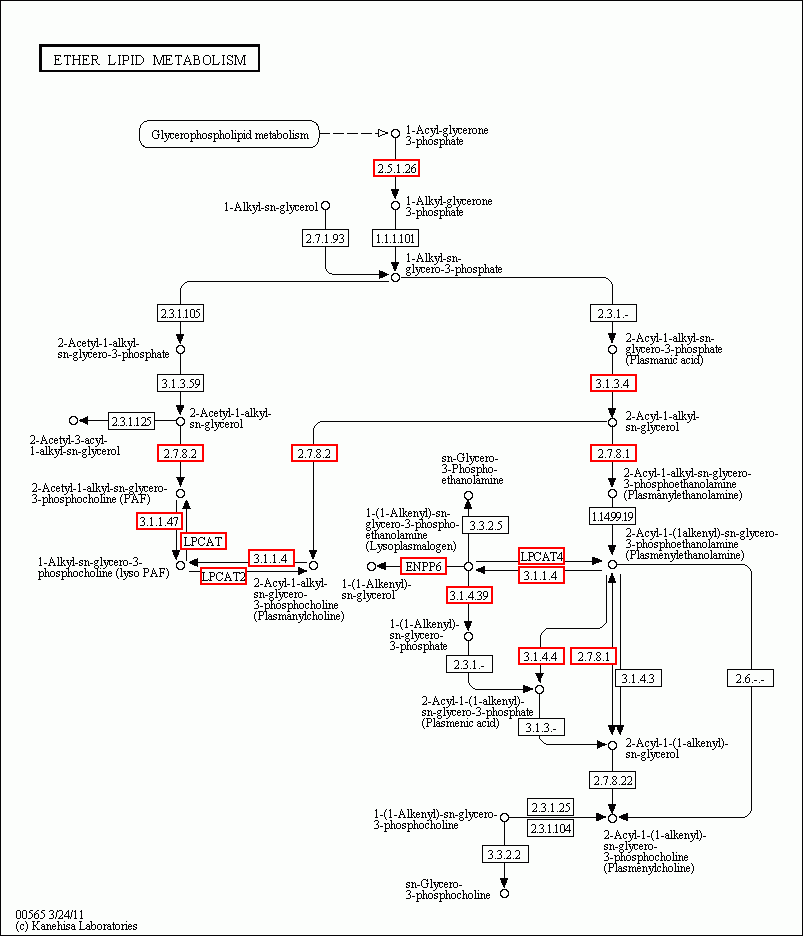

Supplement: Additional file 3: — Pathways found in the annotated portion of the transcriptomes. (ZIP 4950 kb) [file 12864_2015_1817_MOESM3_ESM.zip › map00565.png]

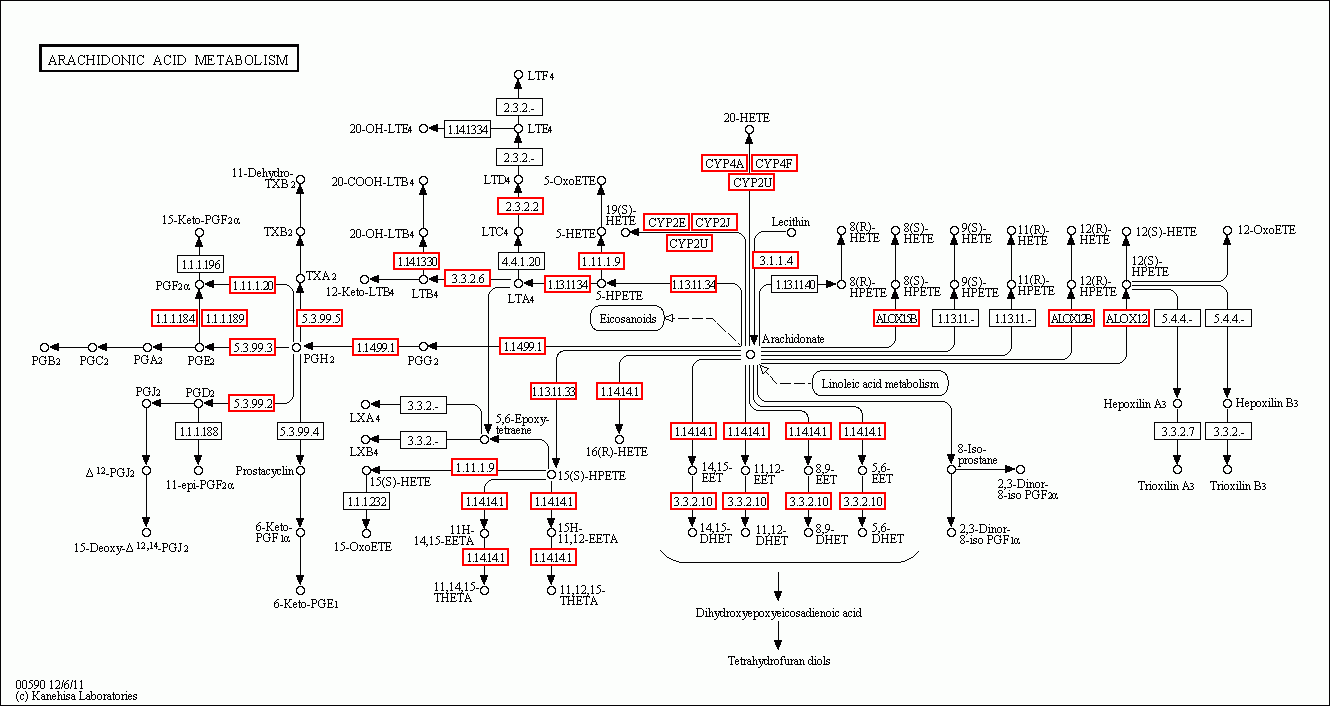

Supplement: Additional file 3: — Pathways found in the annotated portion of the transcriptomes. (ZIP 4950 kb) [file 12864_2015_1817_MOESM3_ESM.zip › map00590.png]

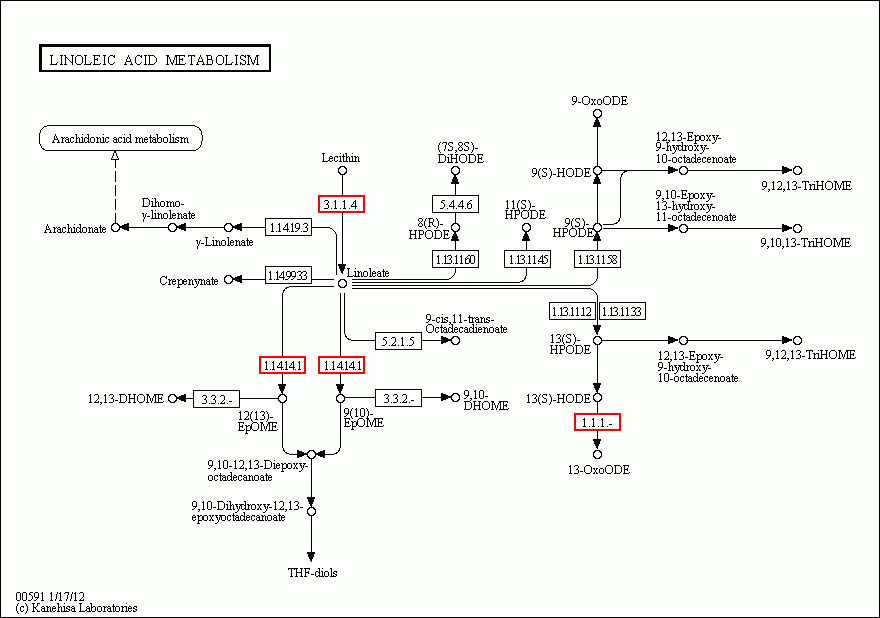

Supplement: Additional file 3: — Pathways found in the annotated portion of the transcriptomes. (ZIP 4950 kb) [file 12864_2015_1817_MOESM3_ESM.zip › map00591.png]

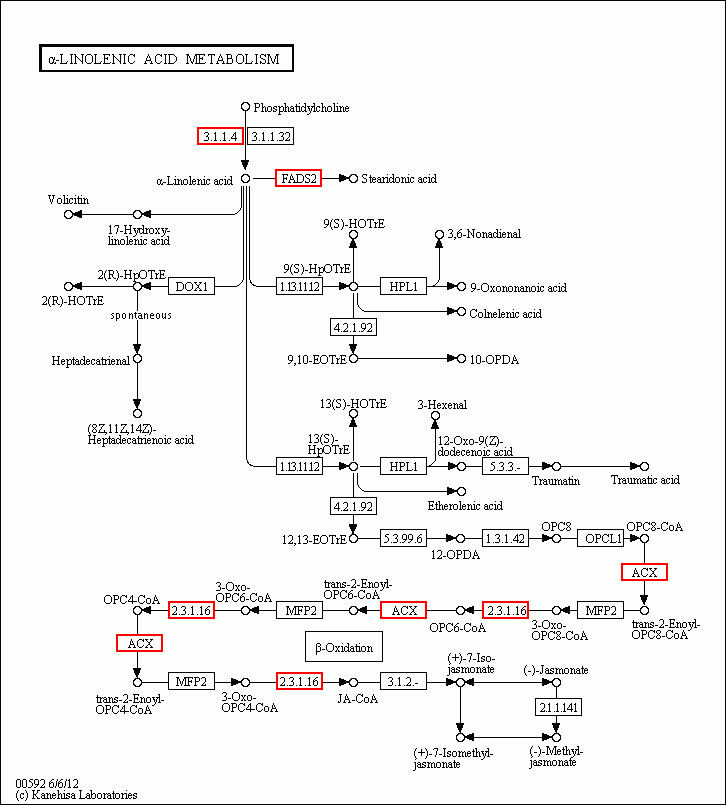

Supplement: Additional file 3: — Pathways found in the annotated portion of the transcriptomes. (ZIP 4950 kb) [file 12864_2015_1817_MOESM3_ESM.zip › map00592.png]

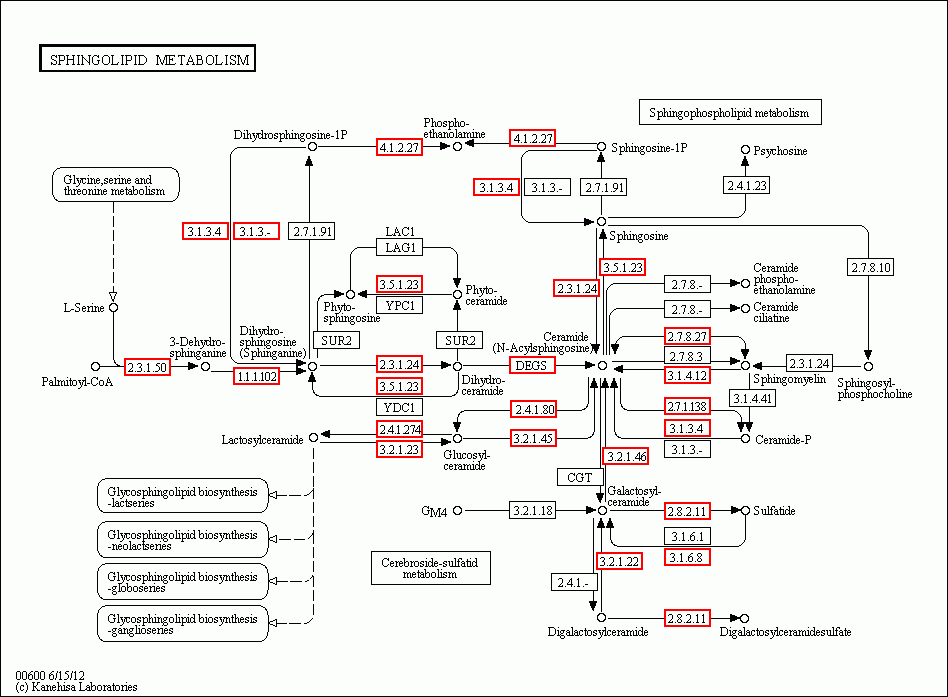

Supplement: Additional file 3: — Pathways found in the annotated portion of the transcriptomes. (ZIP 4950 kb) [file 12864_2015_1817_MOESM3_ESM.zip › map00600.png]

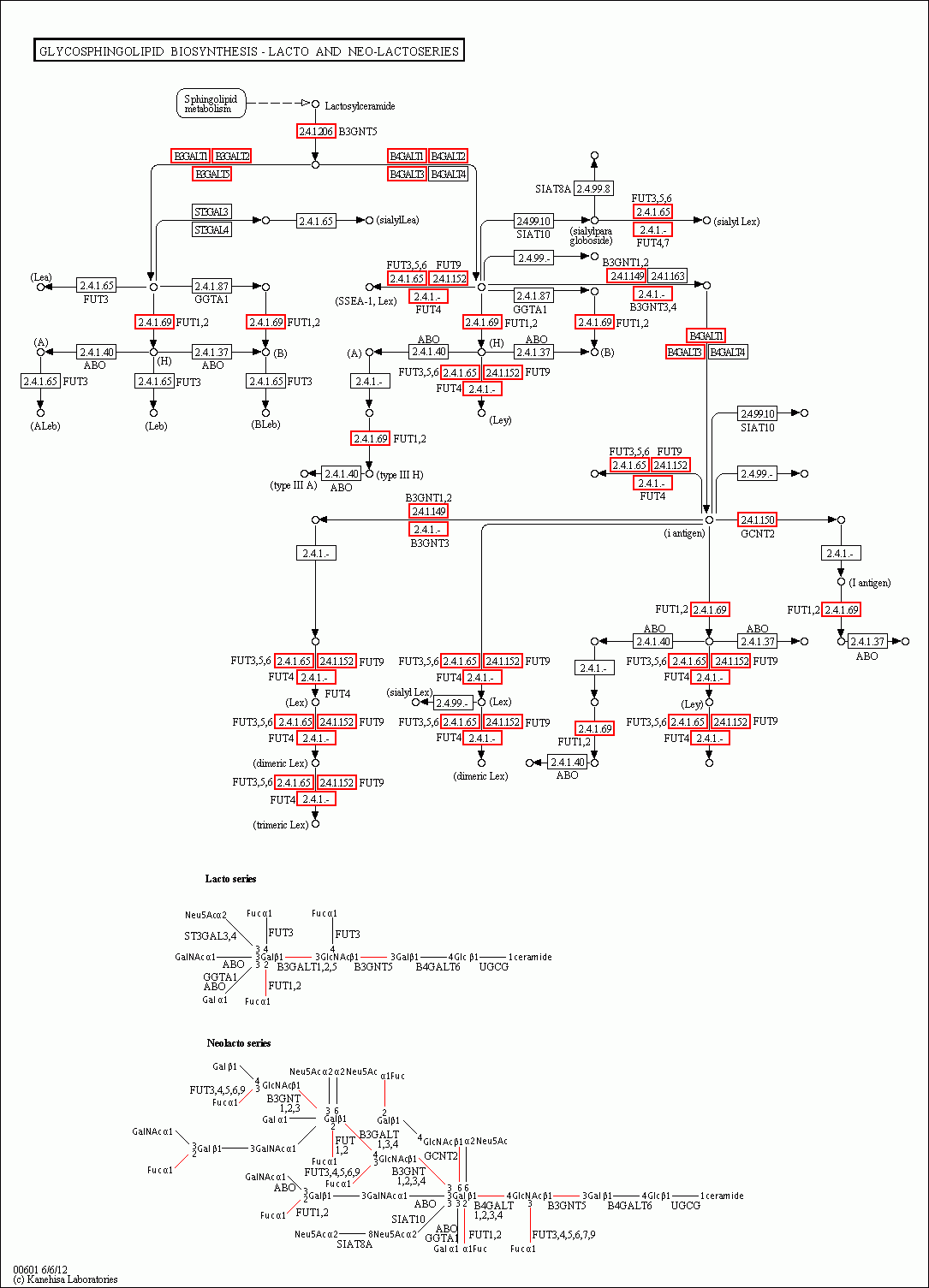

Supplement: Additional file 3: — Pathways found in the annotated portion of the transcriptomes. (ZIP 4950 kb) [file 12864_2015_1817_MOESM3_ESM.zip › map00601.png]

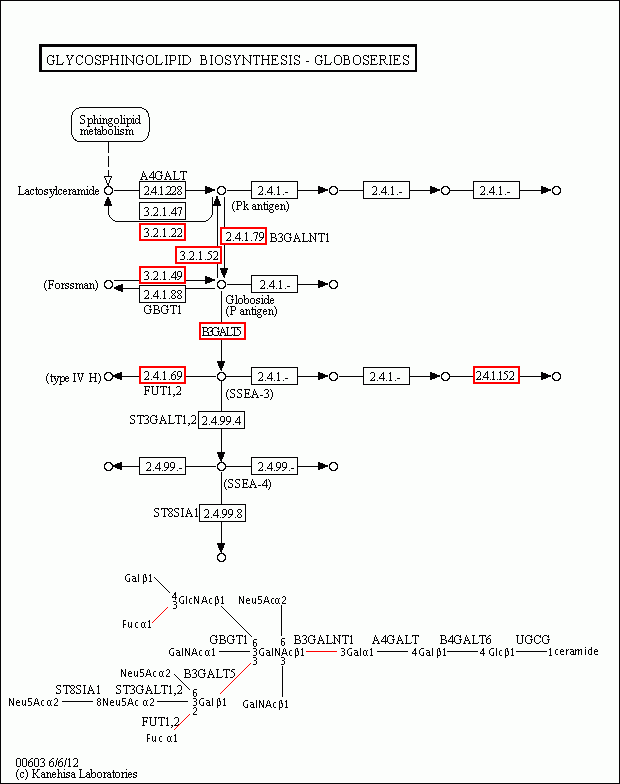

Supplement: Additional file 3: — Pathways found in the annotated portion of the transcriptomes. (ZIP 4950 kb) [file 12864_2015_1817_MOESM3_ESM.zip › map00603.png]

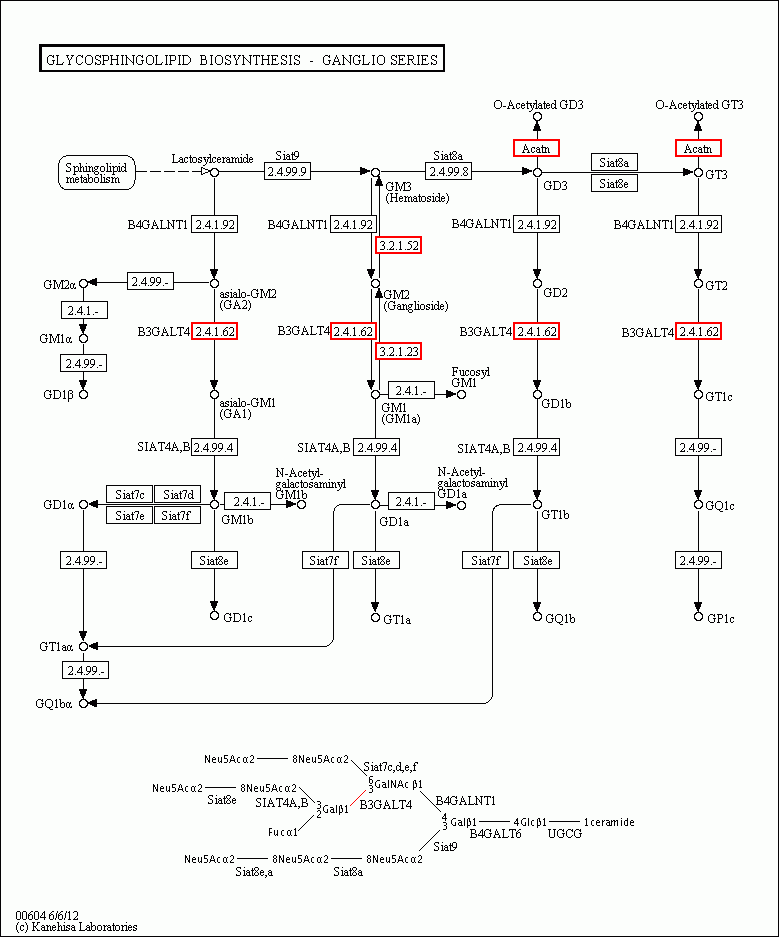

Supplement: Additional file 3: — Pathways found in the annotated portion of the transcriptomes. (ZIP 4950 kb) [file 12864_2015_1817_MOESM3_ESM.zip › map00604.png]

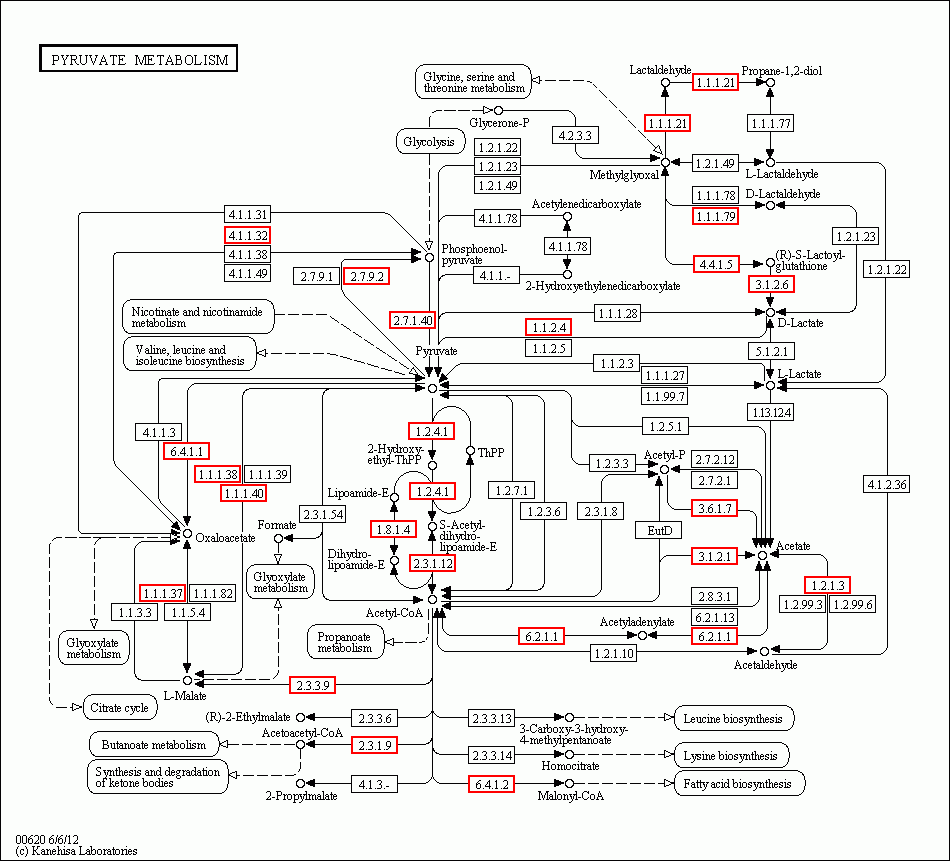

Supplement: Additional file 3: — Pathways found in the annotated portion of the transcriptomes. (ZIP 4950 kb) [file 12864_2015_1817_MOESM3_ESM.zip › map00620.png]

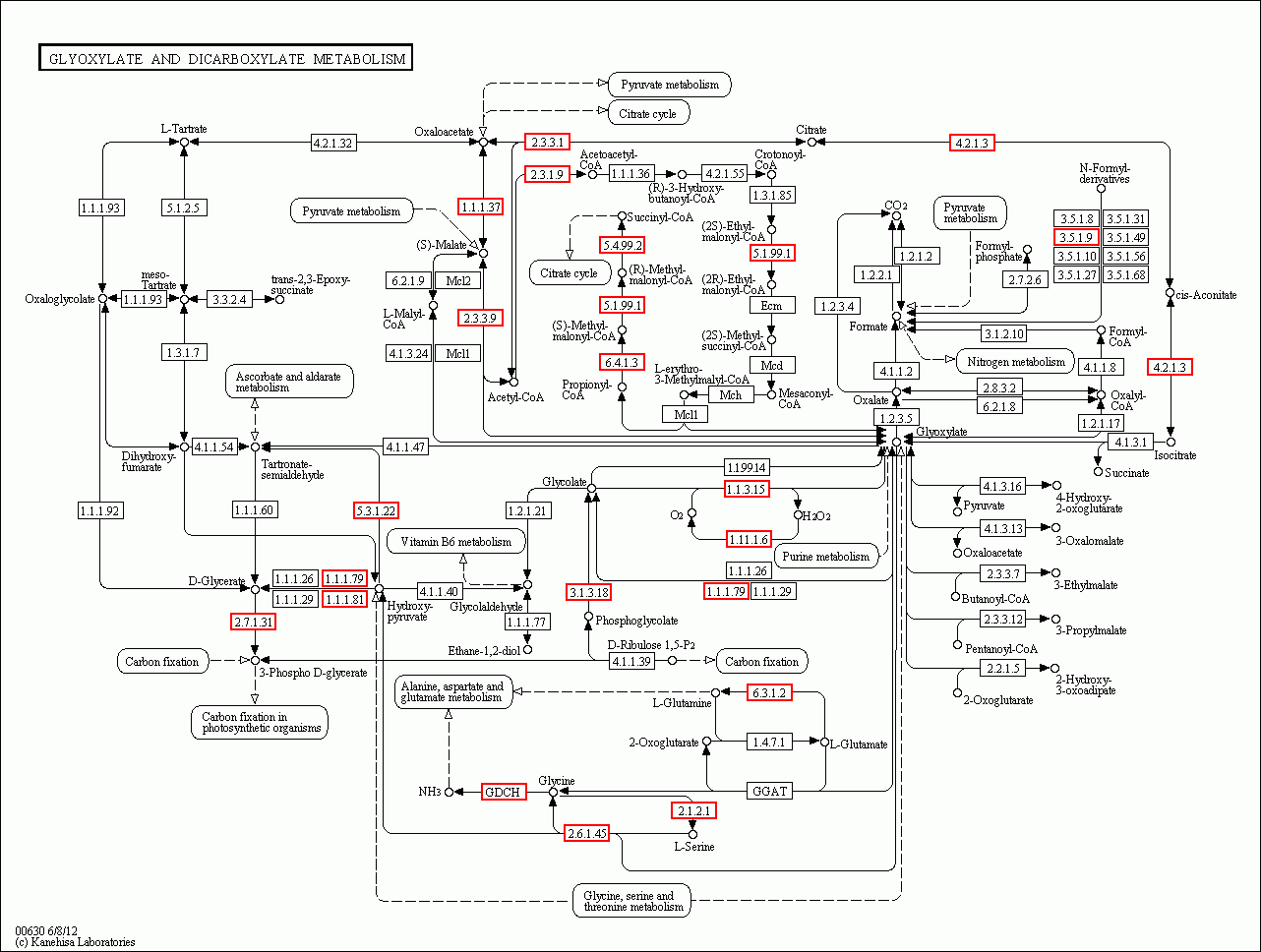

Supplement: Additional file 3: — Pathways found in the annotated portion of the transcriptomes. (ZIP 4950 kb) [file 12864_2015_1817_MOESM3_ESM.zip › map00630.png]

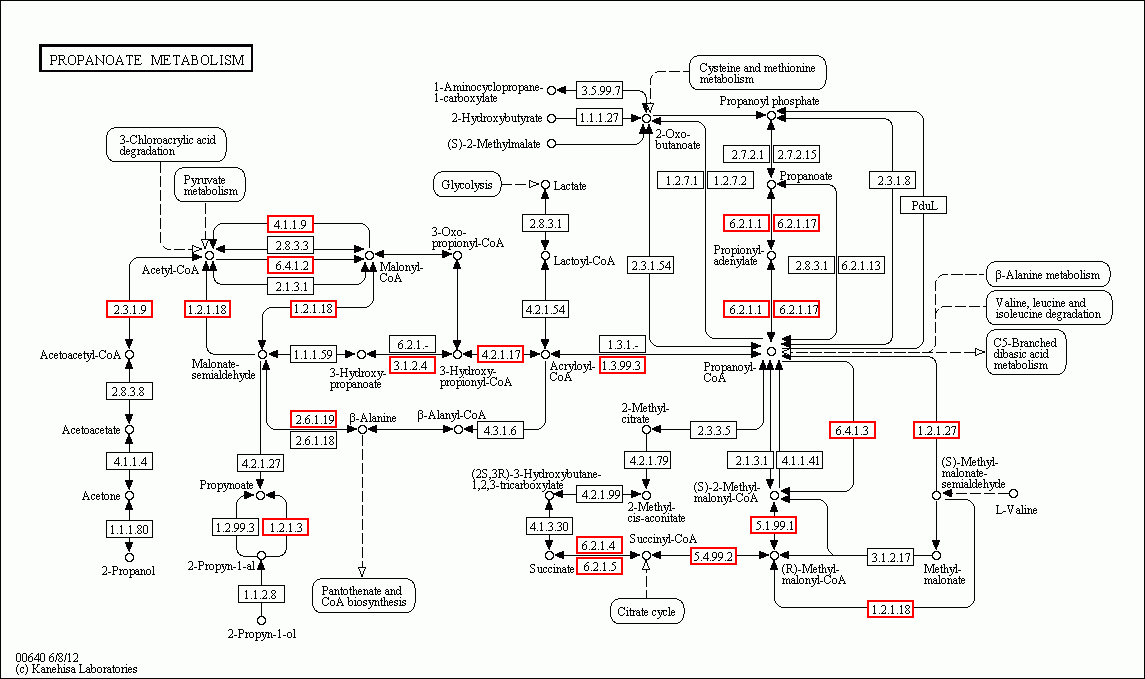

Supplement: Additional file 3: — Pathways found in the annotated portion of the transcriptomes. (ZIP 4950 kb) [file 12864_2015_1817_MOESM3_ESM.zip › map00640.png]

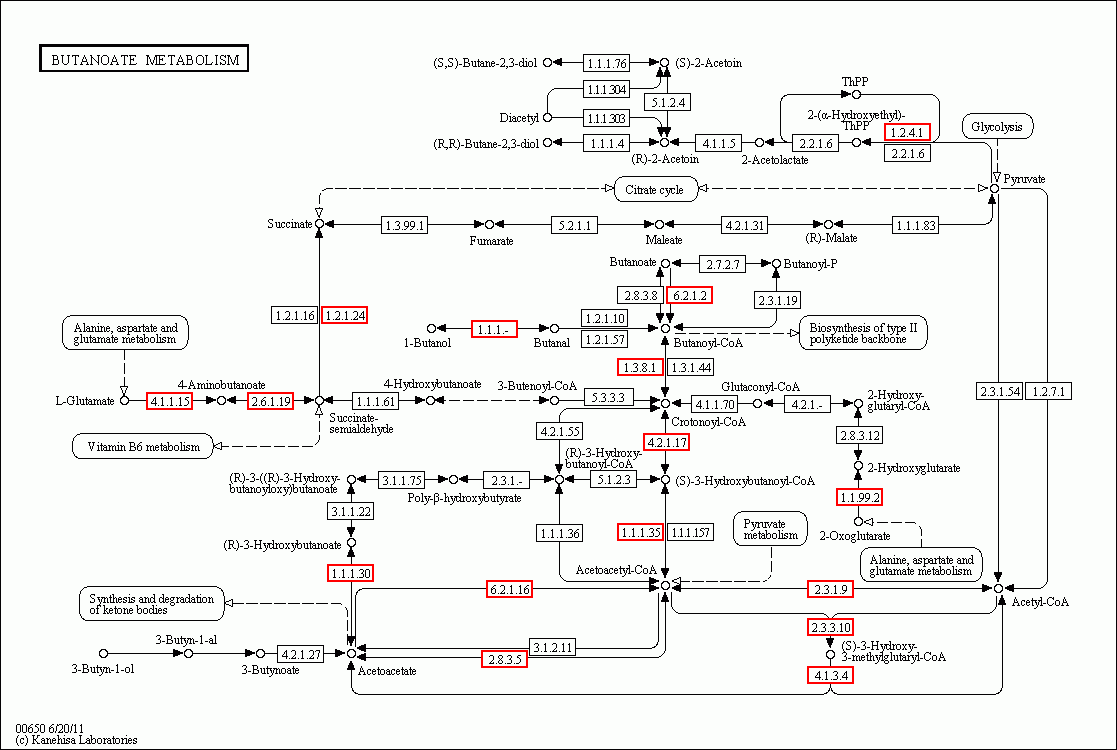

Supplement: Additional file 3: — Pathways found in the annotated portion of the transcriptomes. (ZIP 4950 kb) [file 12864_2015_1817_MOESM3_ESM.zip › map00650.png]

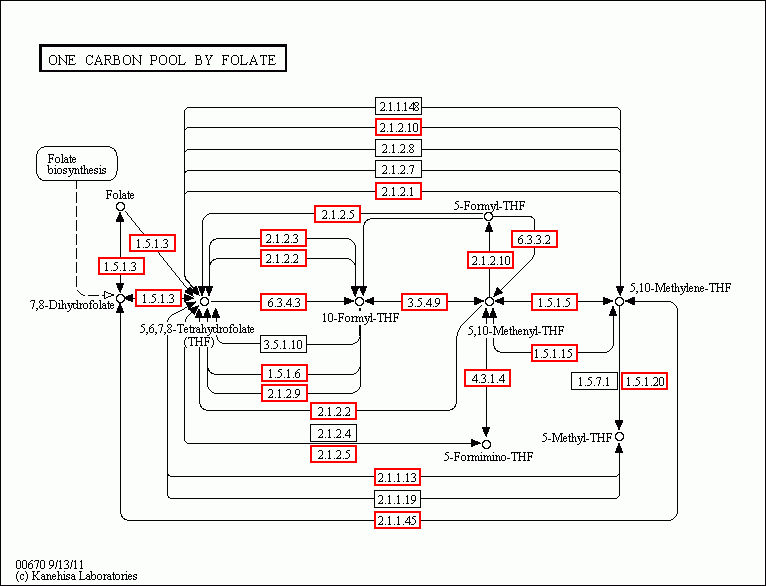

Supplement: Additional file 3: — Pathways found in the annotated portion of the transcriptomes. (ZIP 4950 kb) [file 12864_2015_1817_MOESM3_ESM.zip › map00670.png]

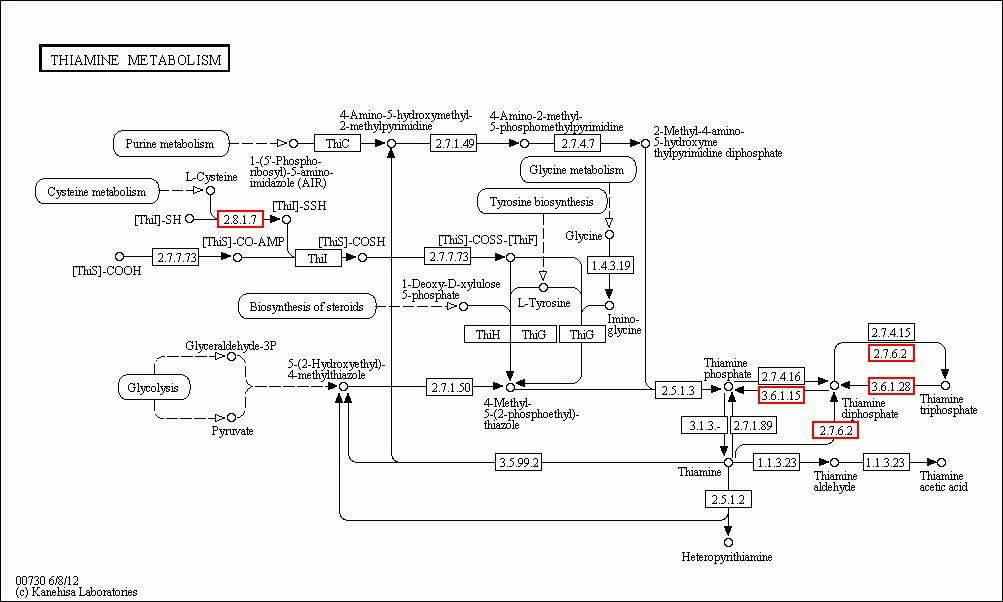

Supplement: Additional file 3: — Pathways found in the annotated portion of the transcriptomes. (ZIP 4950 kb) [file 12864_2015_1817_MOESM3_ESM.zip › map00730.png]

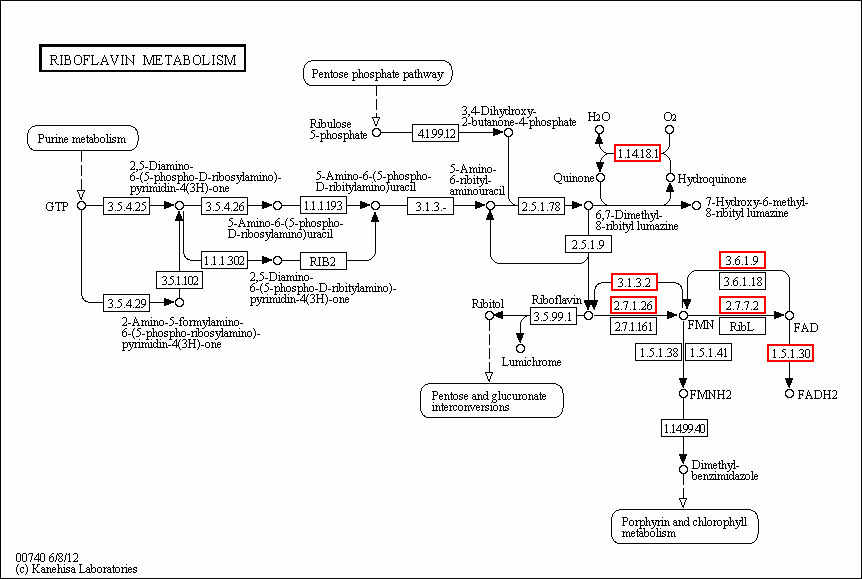

Supplement: Additional file 3: — Pathways found in the annotated portion of the transcriptomes. (ZIP 4950 kb) [file 12864_2015_1817_MOESM3_ESM.zip › map00740.png]

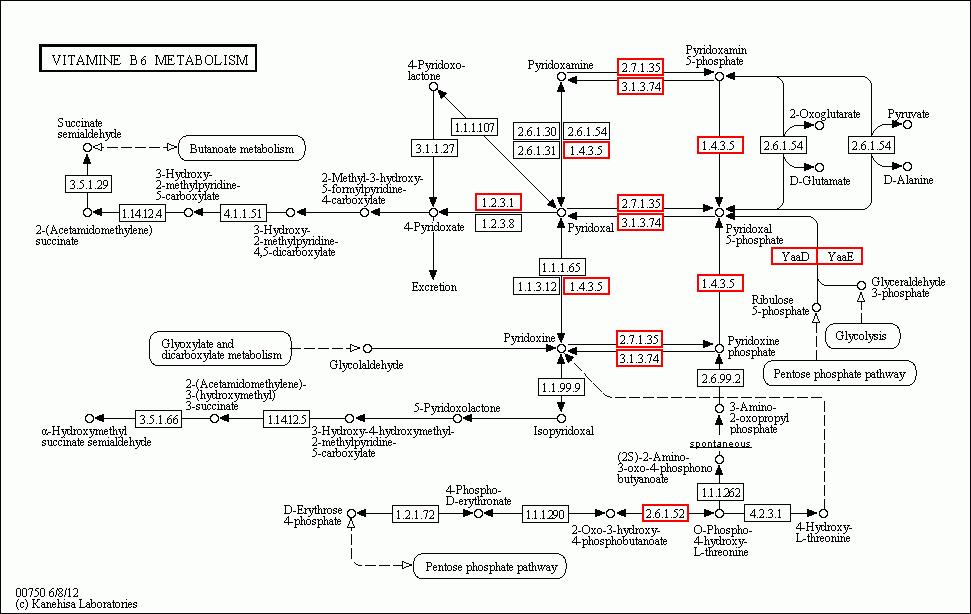

Supplement: Additional file 3: — Pathways found in the annotated portion of the transcriptomes. (ZIP 4950 kb) [file 12864_2015_1817_MOESM3_ESM.zip › map00750.png]

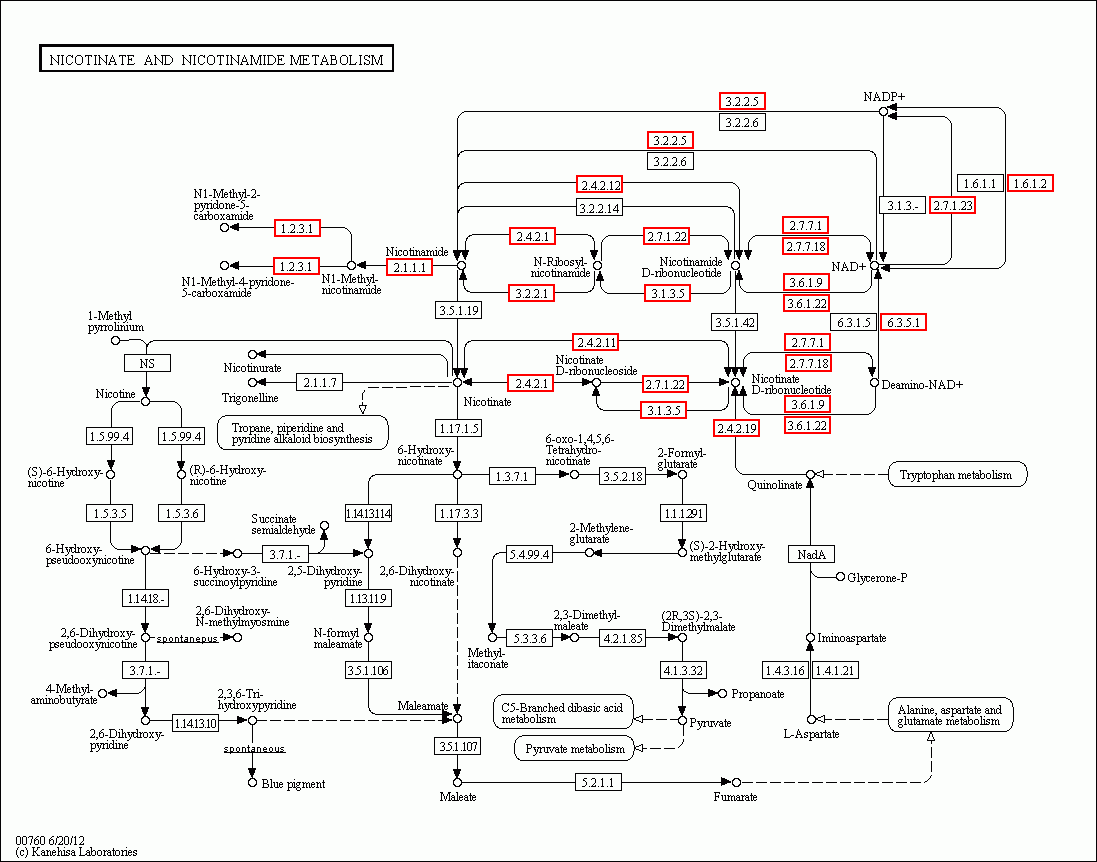

Supplement: Additional file 3: — Pathways found in the annotated portion of the transcriptomes. (ZIP 4950 kb) [file 12864_2015_1817_MOESM3_ESM.zip › map00760.png]

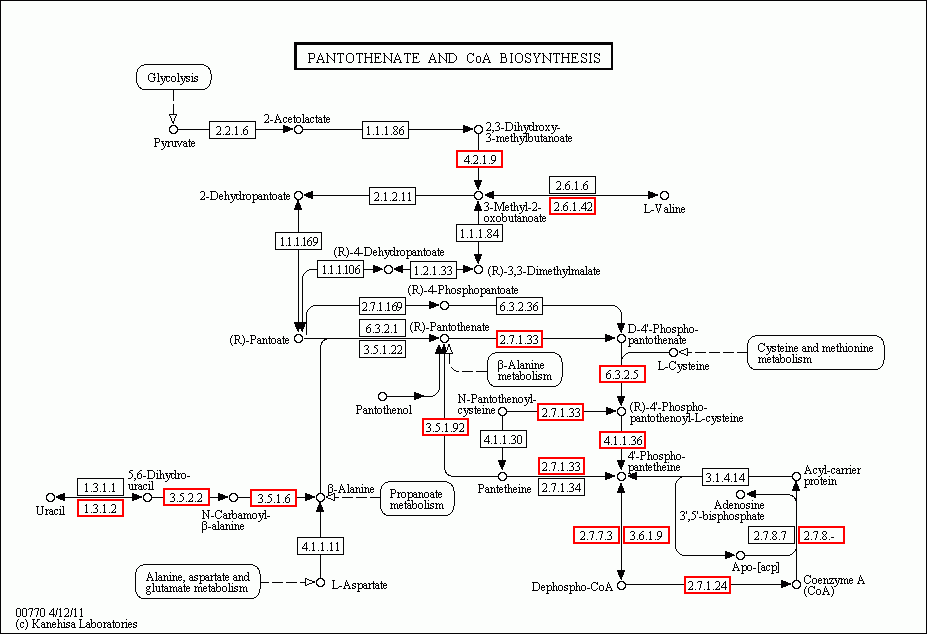

Supplement: Additional file 3: — Pathways found in the annotated portion of the transcriptomes. (ZIP 4950 kb) [file 12864_2015_1817_MOESM3_ESM.zip › map00770.png]

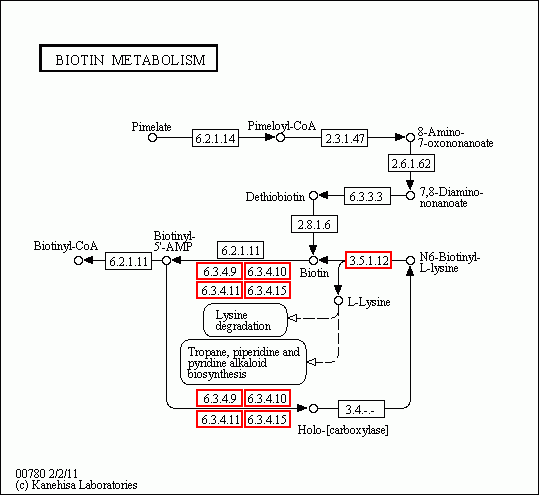

Supplement: Additional file 3: — Pathways found in the annotated portion of the transcriptomes. (ZIP 4950 kb) [file 12864_2015_1817_MOESM3_ESM.zip › map00780.png]

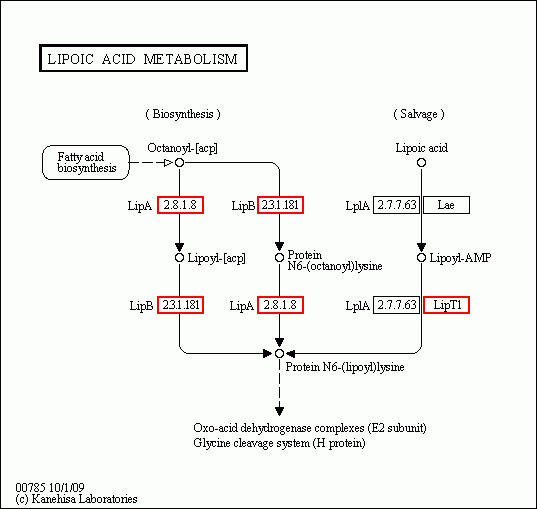

Supplement: Additional file 3: — Pathways found in the annotated portion of the transcriptomes. (ZIP 4950 kb) [file 12864_2015_1817_MOESM3_ESM.zip › map00785.png]

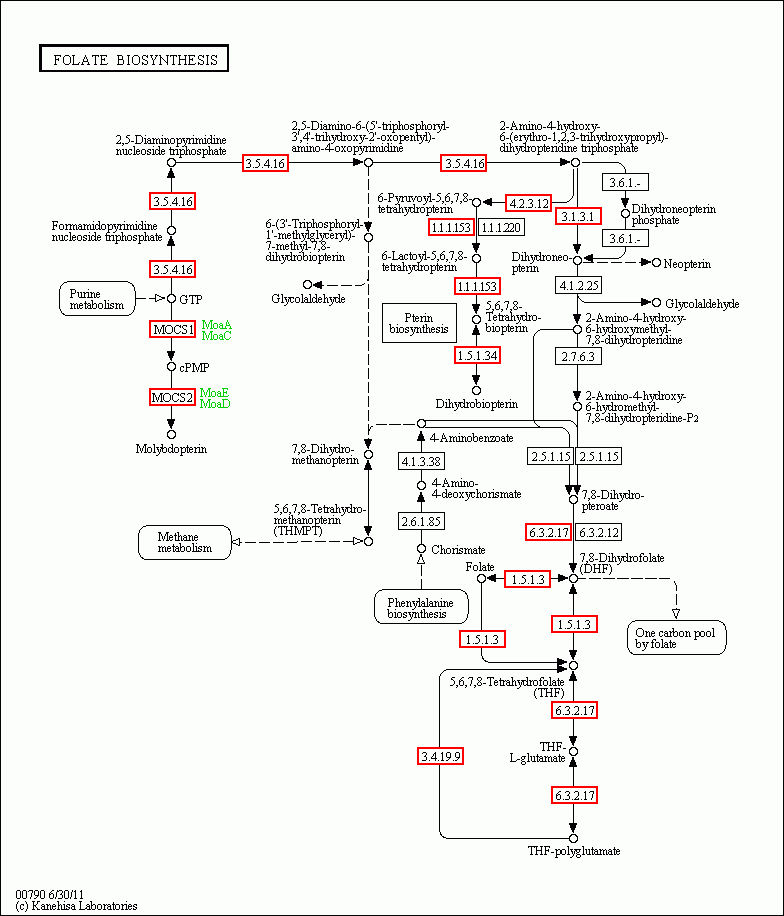

Supplement: Additional file 3: — Pathways found in the annotated portion of the transcriptomes. (ZIP 4950 kb) [file 12864_2015_1817_MOESM3_ESM.zip › map00790.png]

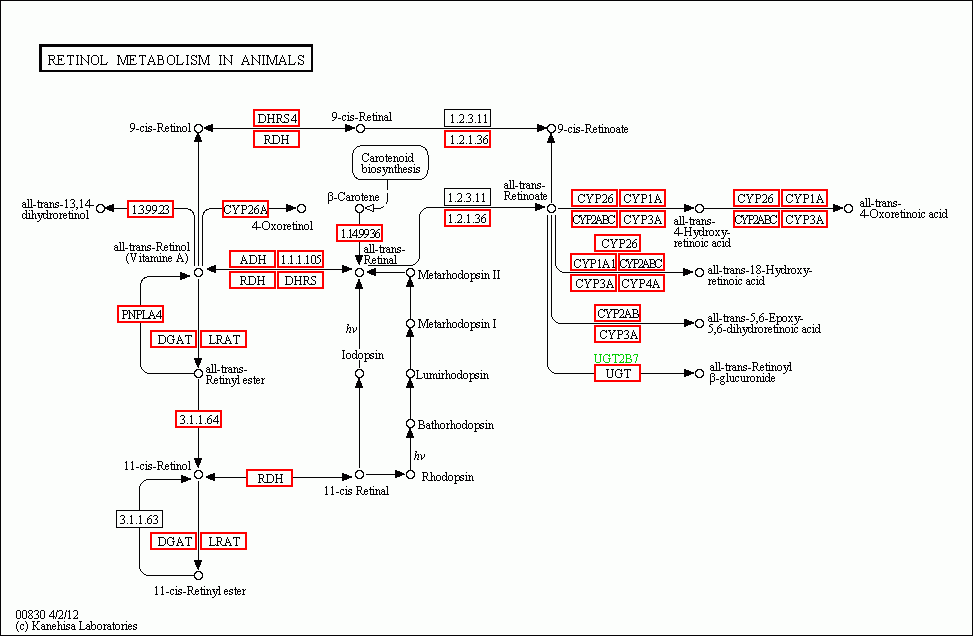

Supplement: Additional file 3: — Pathways found in the annotated portion of the transcriptomes. (ZIP 4950 kb) [file 12864_2015_1817_MOESM3_ESM.zip › map00830.png]

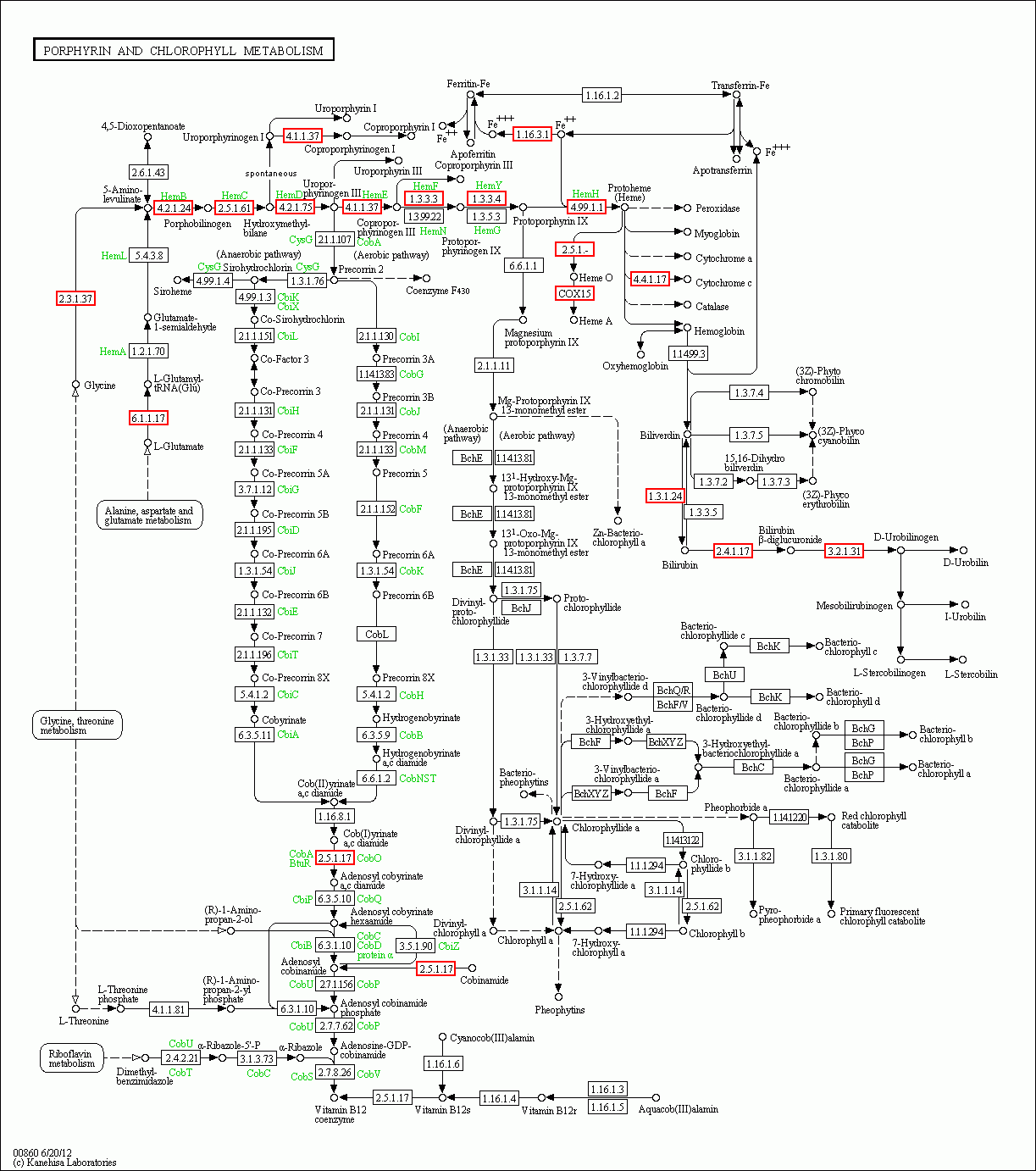

Supplement: Additional file 3: — Pathways found in the annotated portion of the transcriptomes. (ZIP 4950 kb) [file 12864_2015_1817_MOESM3_ESM.zip › map00860.png]

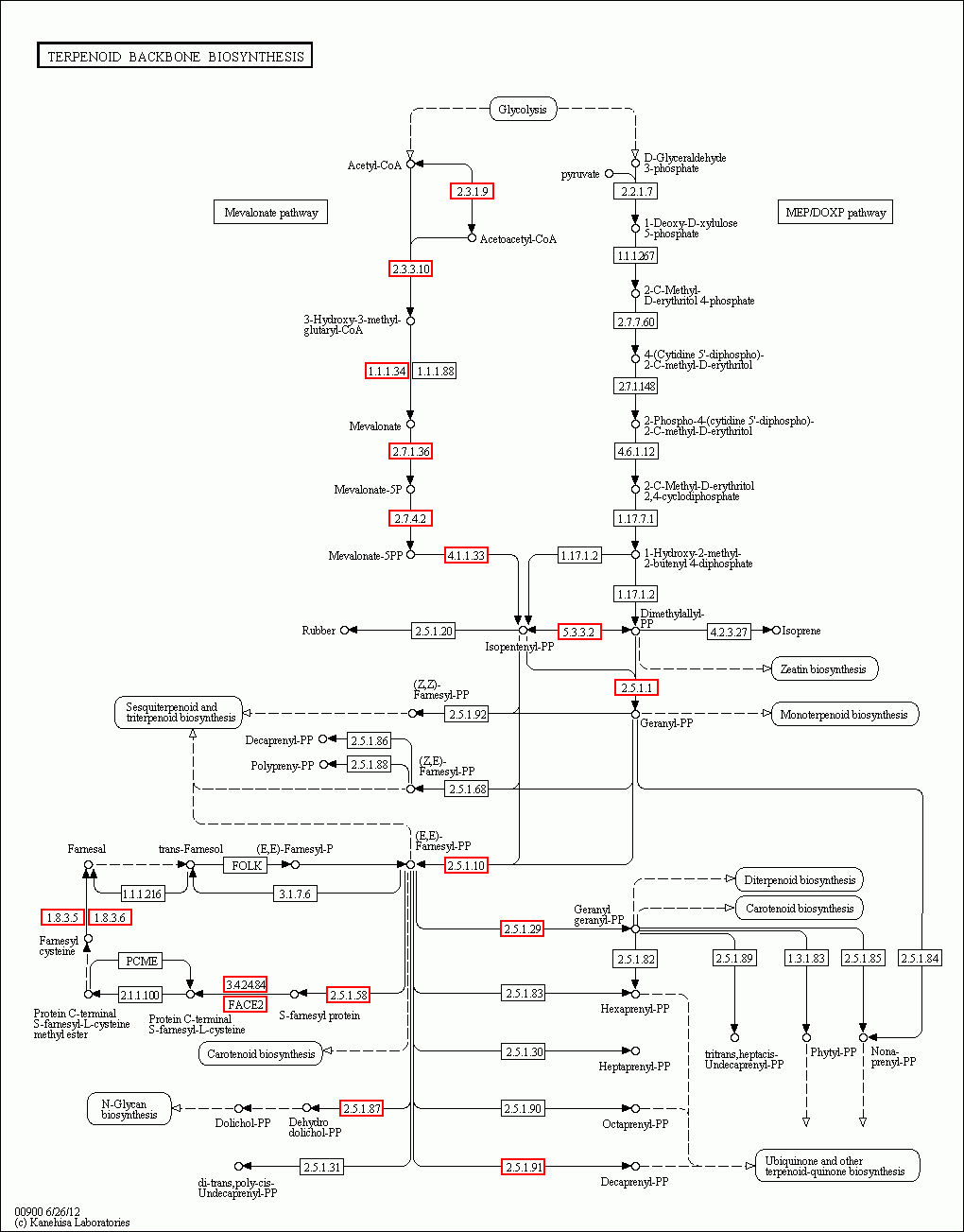

Supplement: Additional file 3: — Pathways found in the annotated portion of the transcriptomes. (ZIP 4950 kb) [file 12864_2015_1817_MOESM3_ESM.zip › map00900.png]

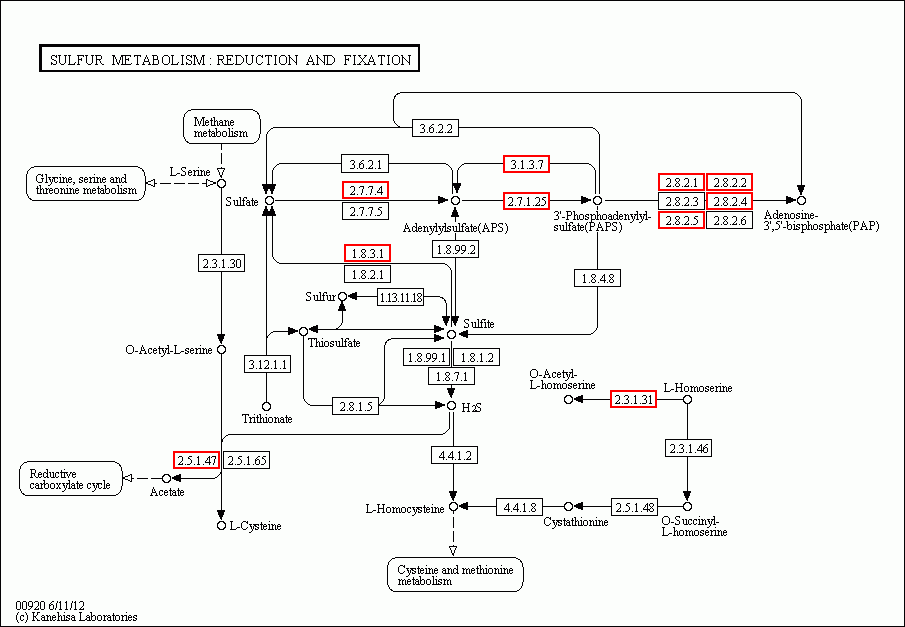

Supplement: Additional file 3: — Pathways found in the annotated portion of the transcriptomes. (ZIP 4950 kb) [file 12864_2015_1817_MOESM3_ESM.zip › map00920.png]

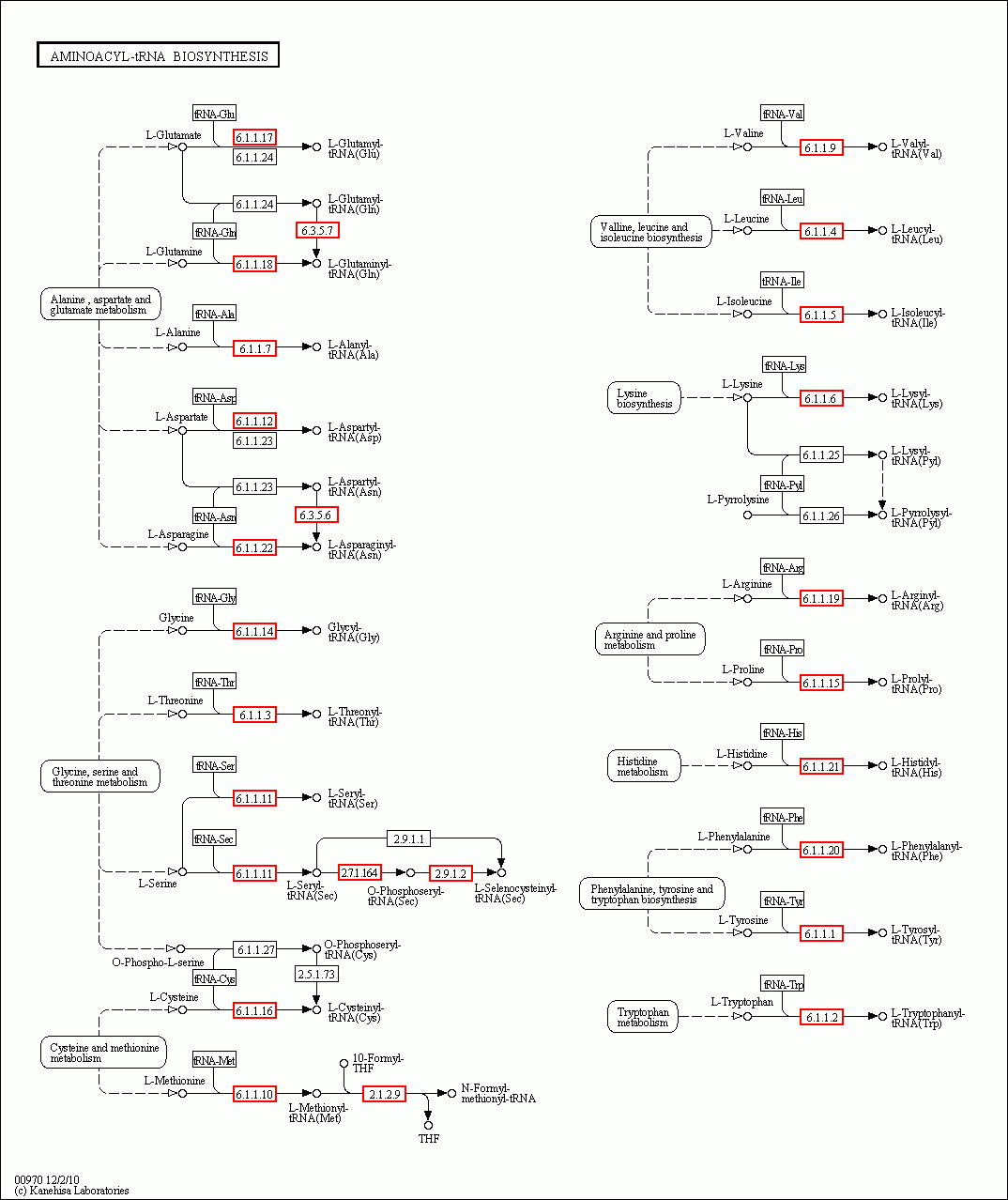

Supplement: Additional file 3: — Pathways found in the annotated portion of the transcriptomes. (ZIP 4950 kb) [file 12864_2015_1817_MOESM3_ESM.zip › map00970.png]

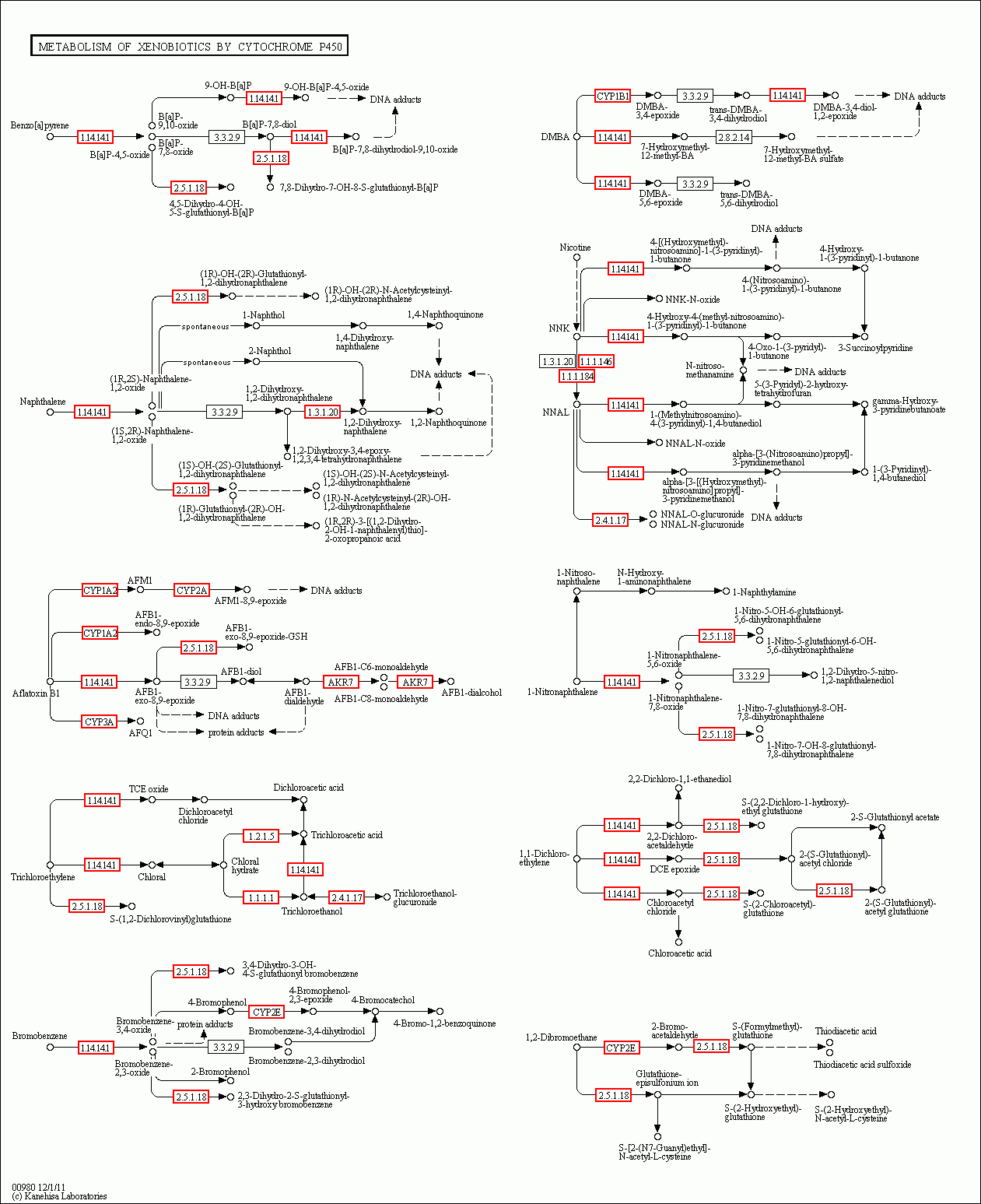

Supplement: Additional file 3: — Pathways found in the annotated portion of the transcriptomes. (ZIP 4950 kb) [file 12864_2015_1817_MOESM3_ESM.zip › map00980.png]

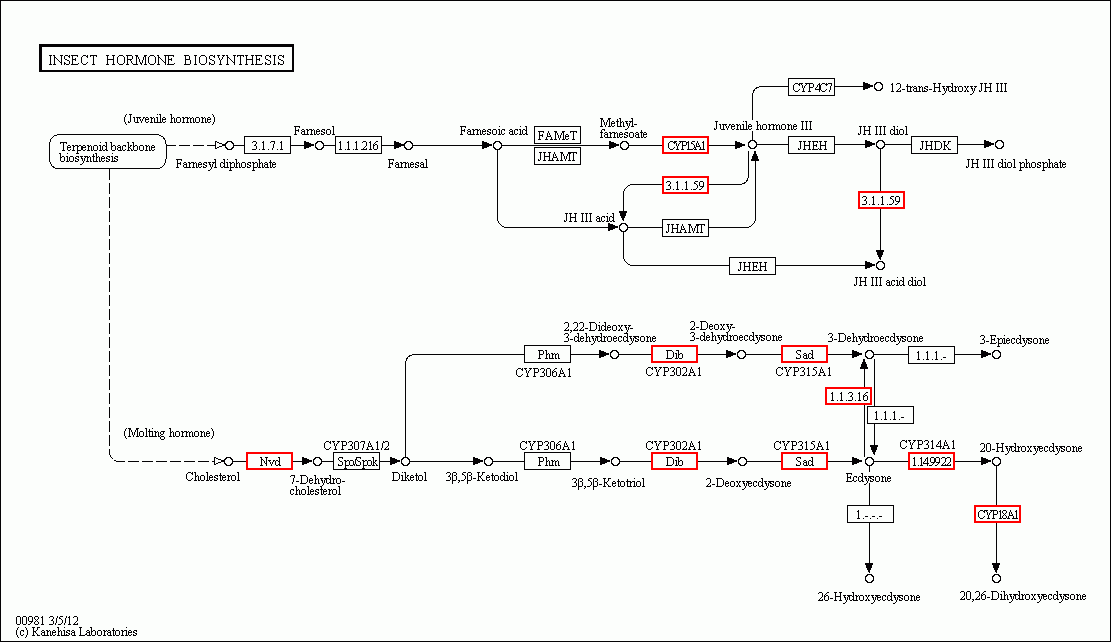

Supplement: Additional file 3: — Pathways found in the annotated portion of the transcriptomes. (ZIP 4950 kb) [file 12864_2015_1817_MOESM3_ESM.zip › map00981.png]

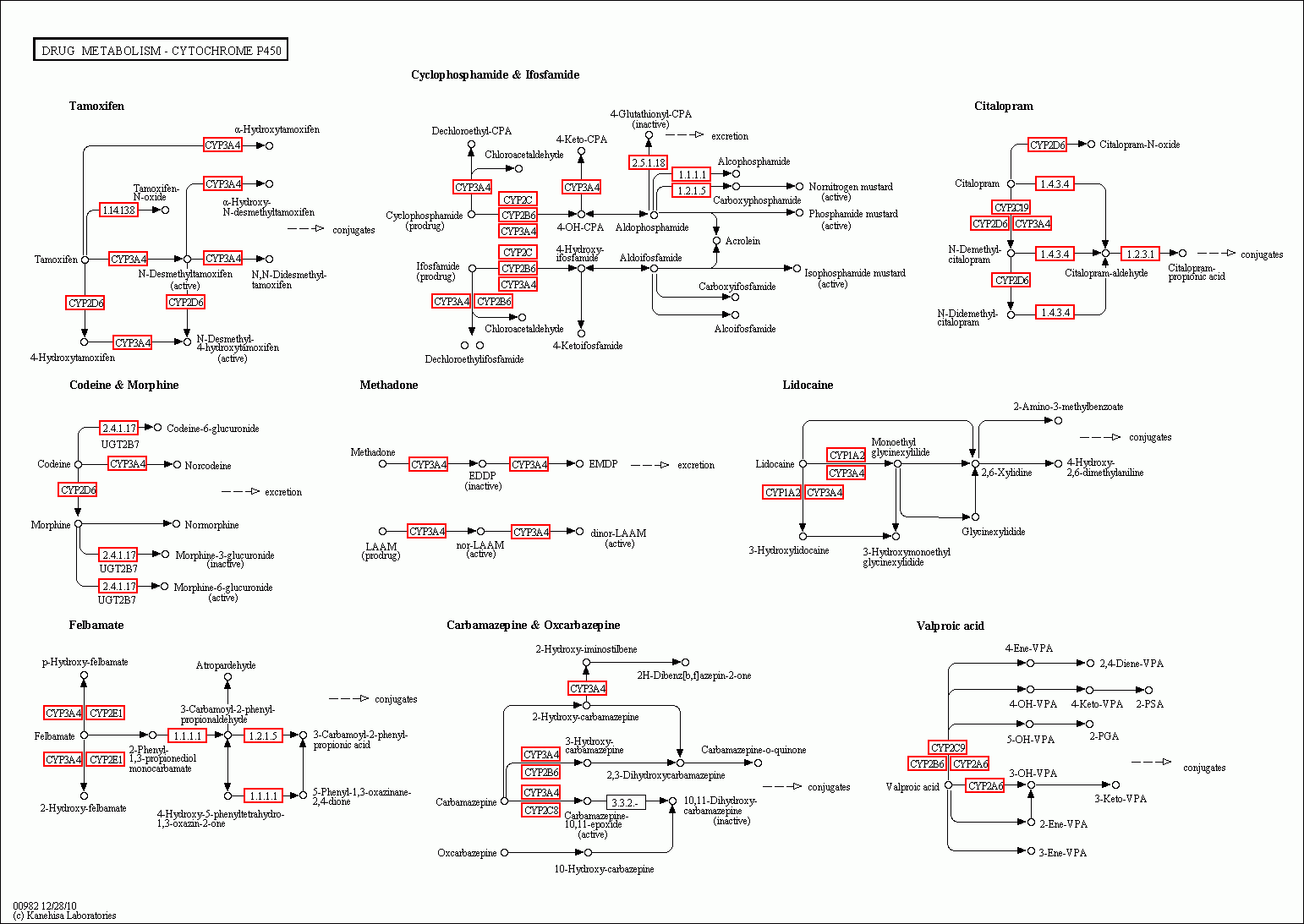

Supplement: Additional file 3: — Pathways found in the annotated portion of the transcriptomes. (ZIP 4950 kb) [file 12864_2015_1817_MOESM3_ESM.zip › map00982.png]

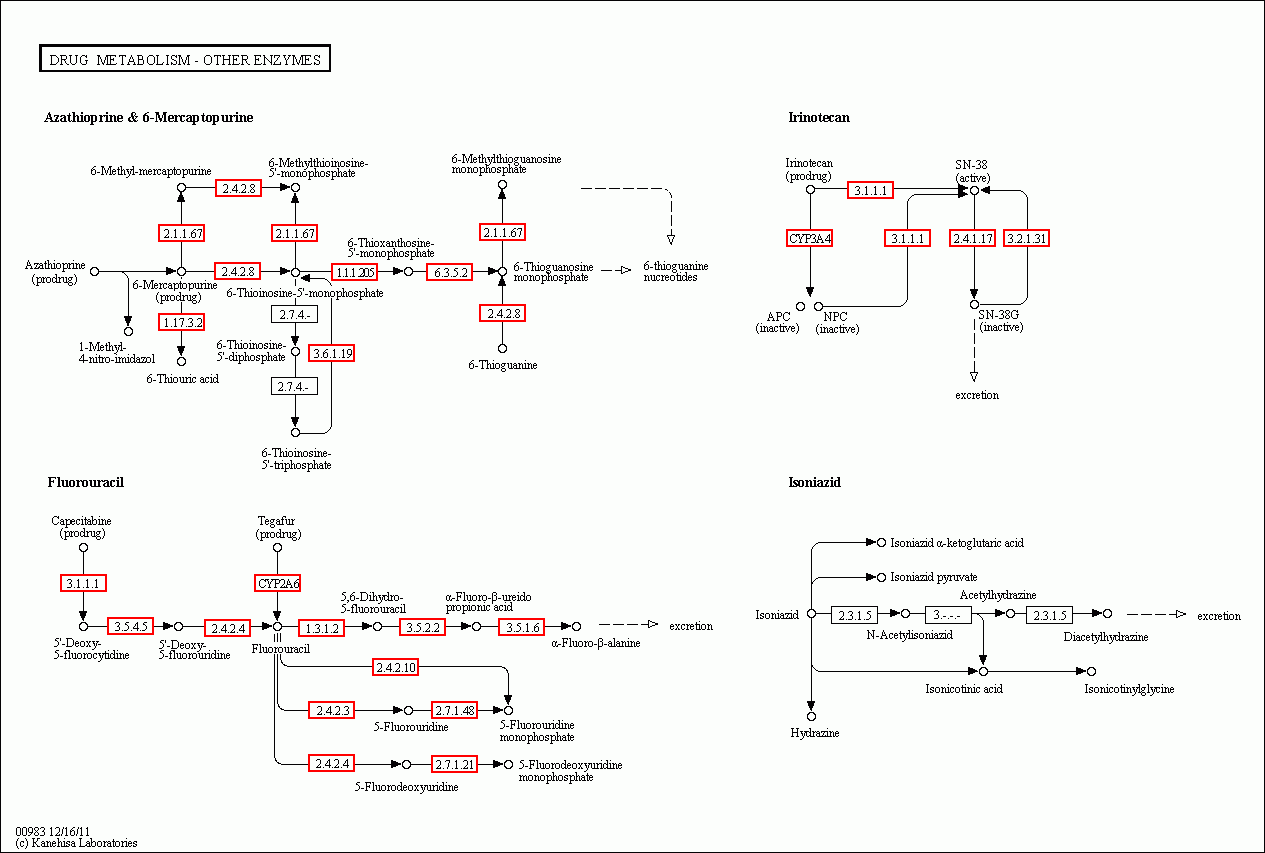

Supplement: Additional file 3: — Pathways found in the annotated portion of the transcriptomes. (ZIP 4950 kb) [file 12864_2015_1817_MOESM3_ESM.zip › map00983.png]

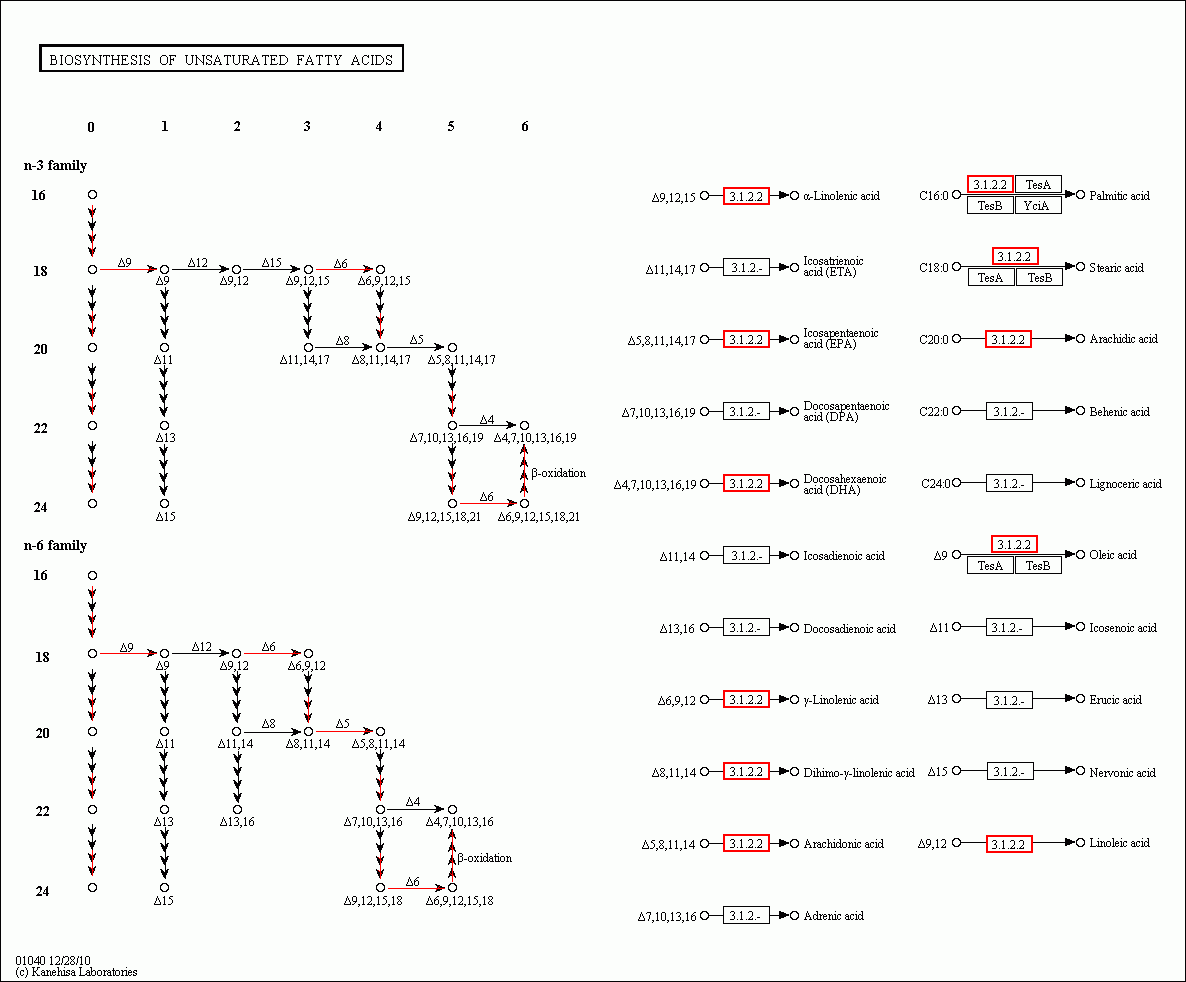

Supplement: Additional file 3: — Pathways found in the annotated portion of the transcriptomes. (ZIP 4950 kb) [file 12864_2015_1817_MOESM3_ESM.zip › map01040.png]

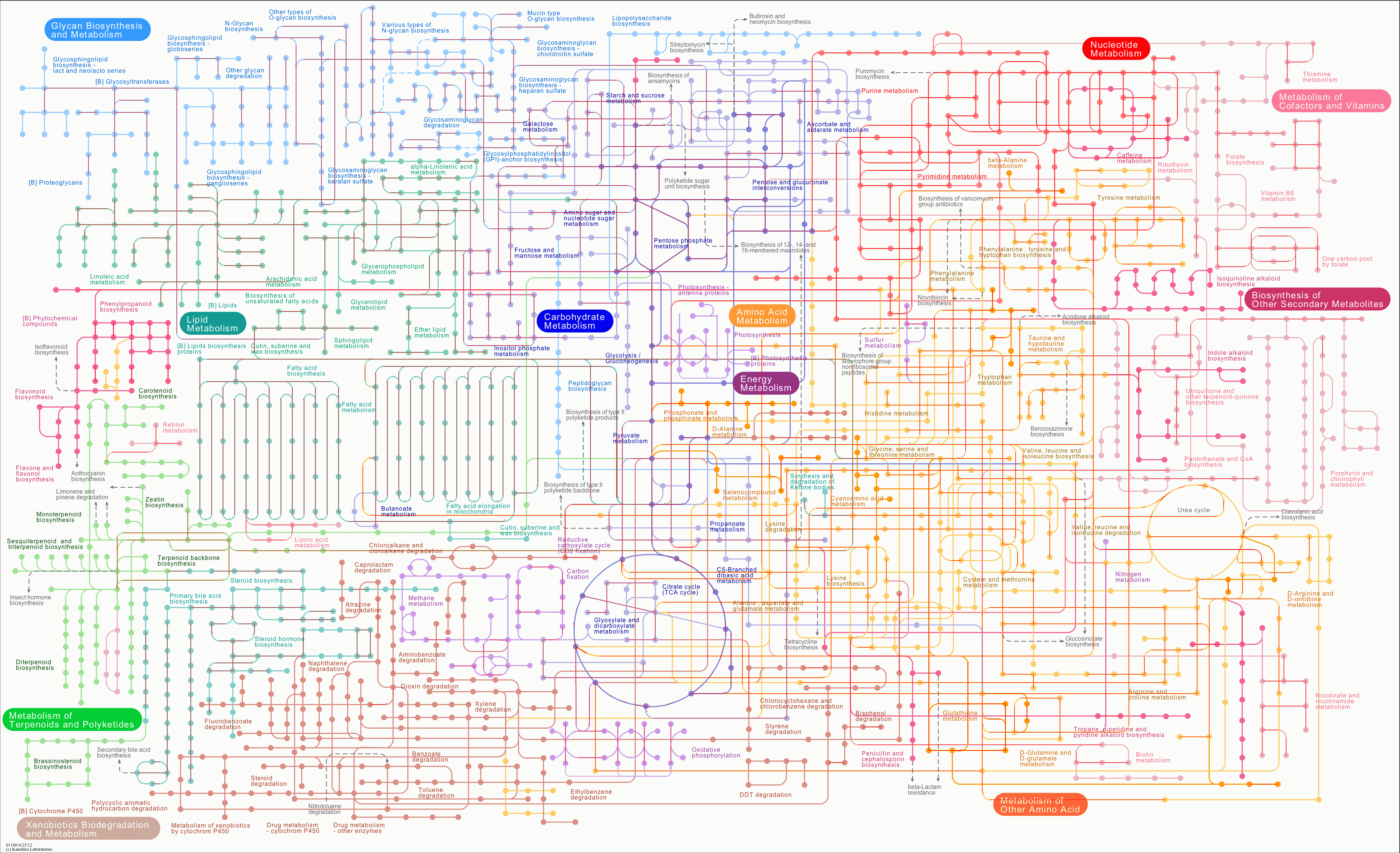

Supplement: Additional file 3: — Pathways found in the annotated portion of the transcriptomes. (ZIP 4950 kb) [file 12864_2015_1817_MOESM3_ESM.zip › map01100.png]

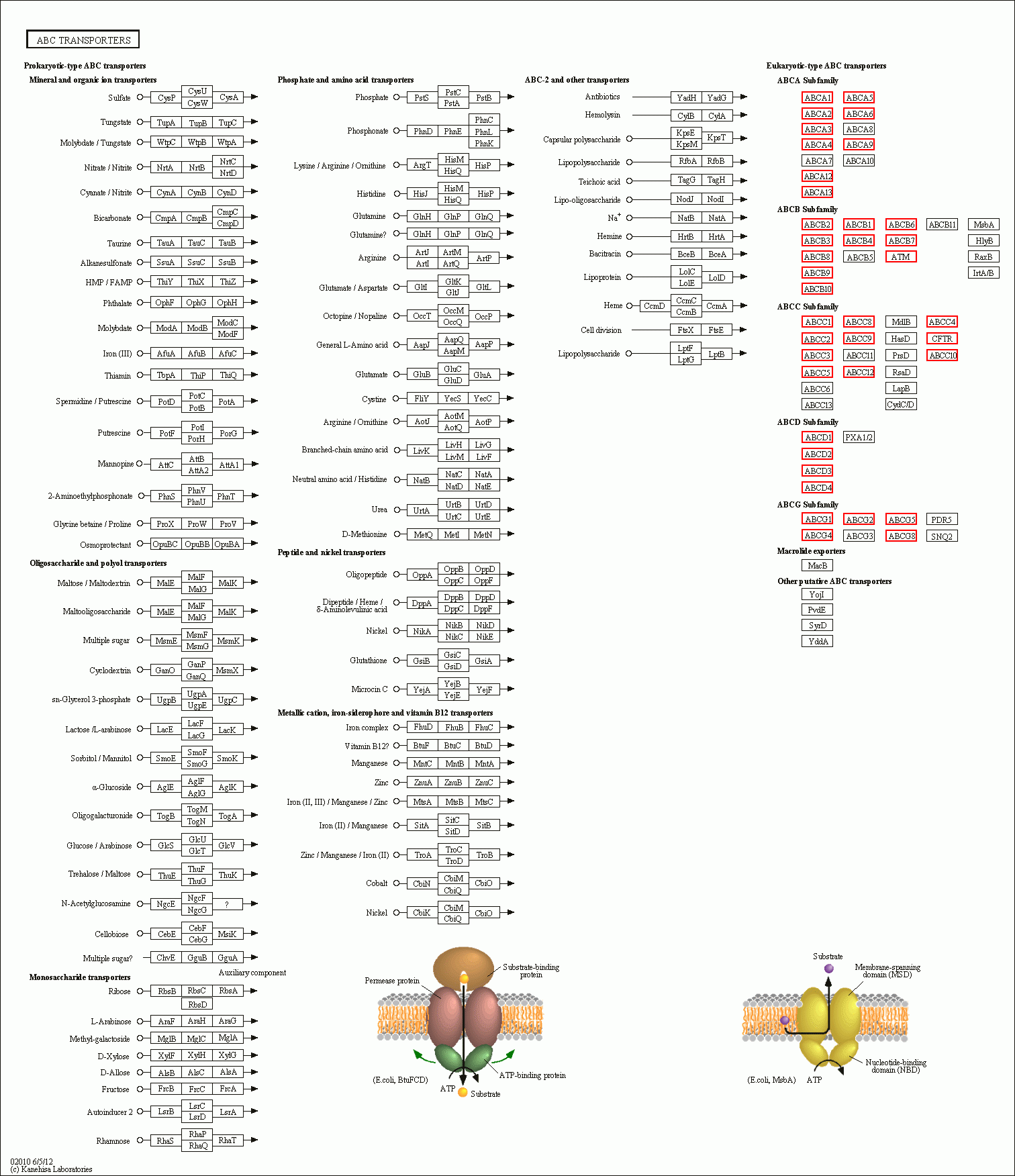

Supplement: Additional file 3: — Pathways found in the annotated portion of the transcriptomes. (ZIP 4950 kb) [file 12864_2015_1817_MOESM3_ESM.zip › map02010.png]

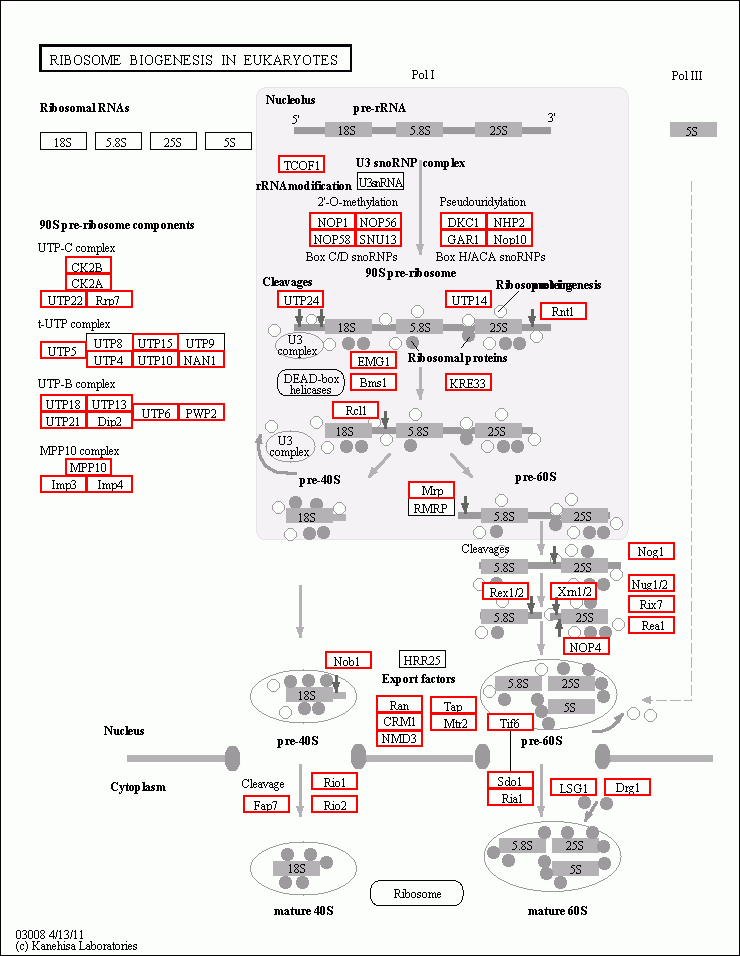

Supplement: Additional file 3: — Pathways found in the annotated portion of the transcriptomes. (ZIP 4950 kb) [file 12864_2015_1817_MOESM3_ESM.zip › map03008.png]

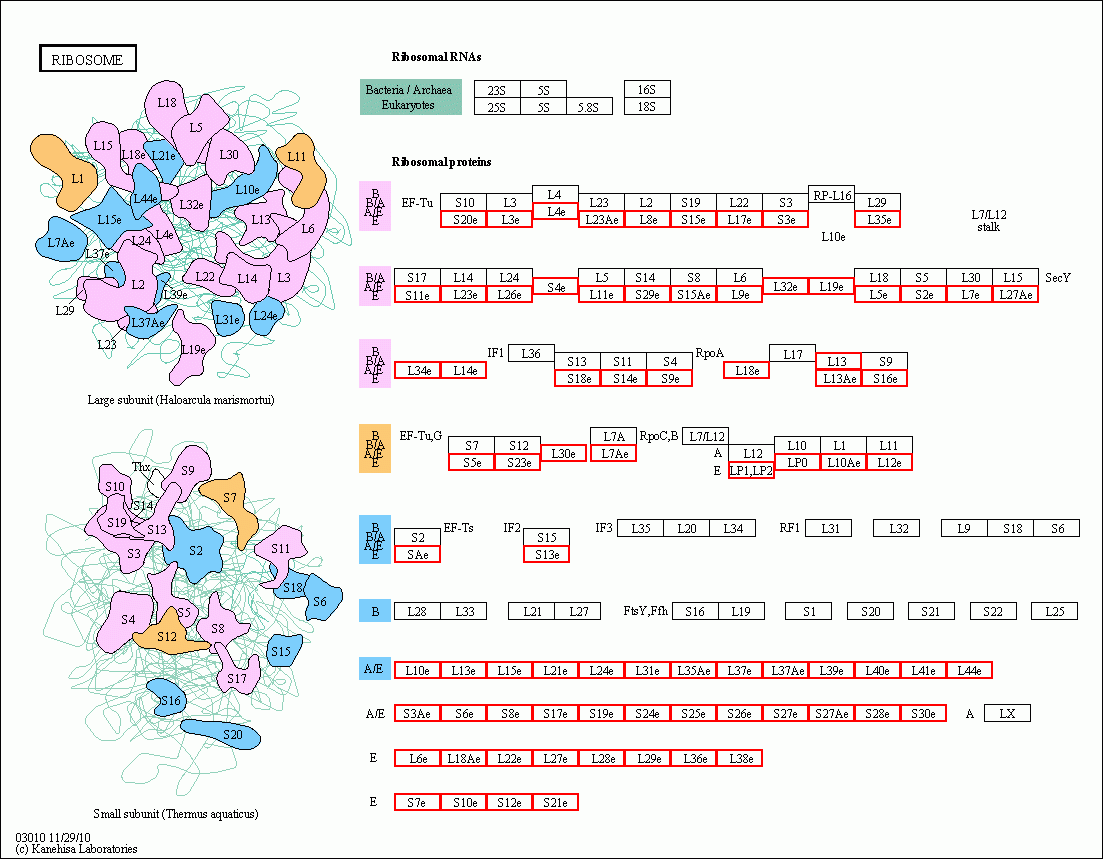

Supplement: Additional file 3: — Pathways found in the annotated portion of the transcriptomes. (ZIP 4950 kb) [file 12864_2015_1817_MOESM3_ESM.zip › map03010.png]

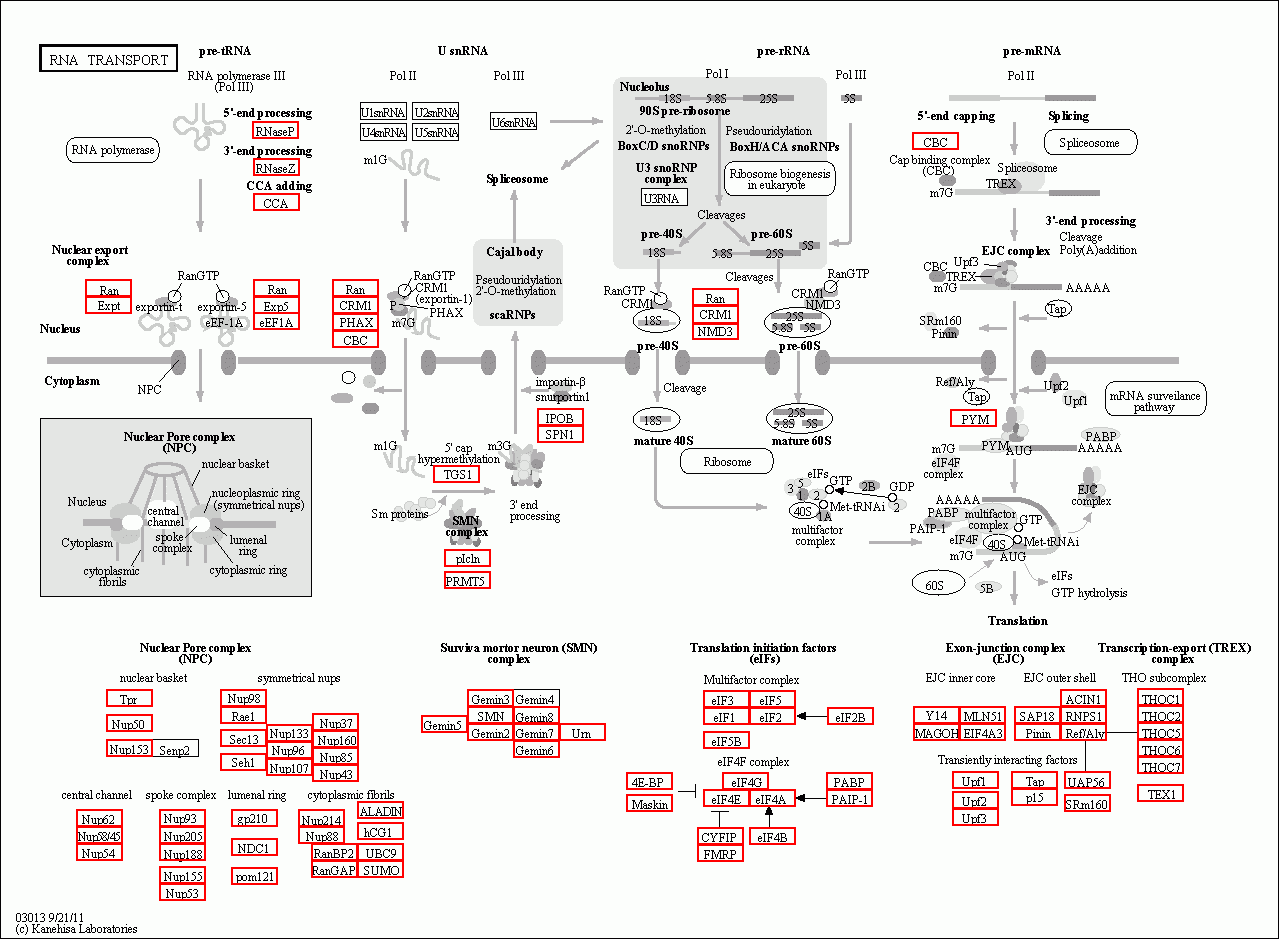

Supplement: Additional file 3: — Pathways found in the annotated portion of the transcriptomes. (ZIP 4950 kb) [file 12864_2015_1817_MOESM3_ESM.zip › map03013.png]

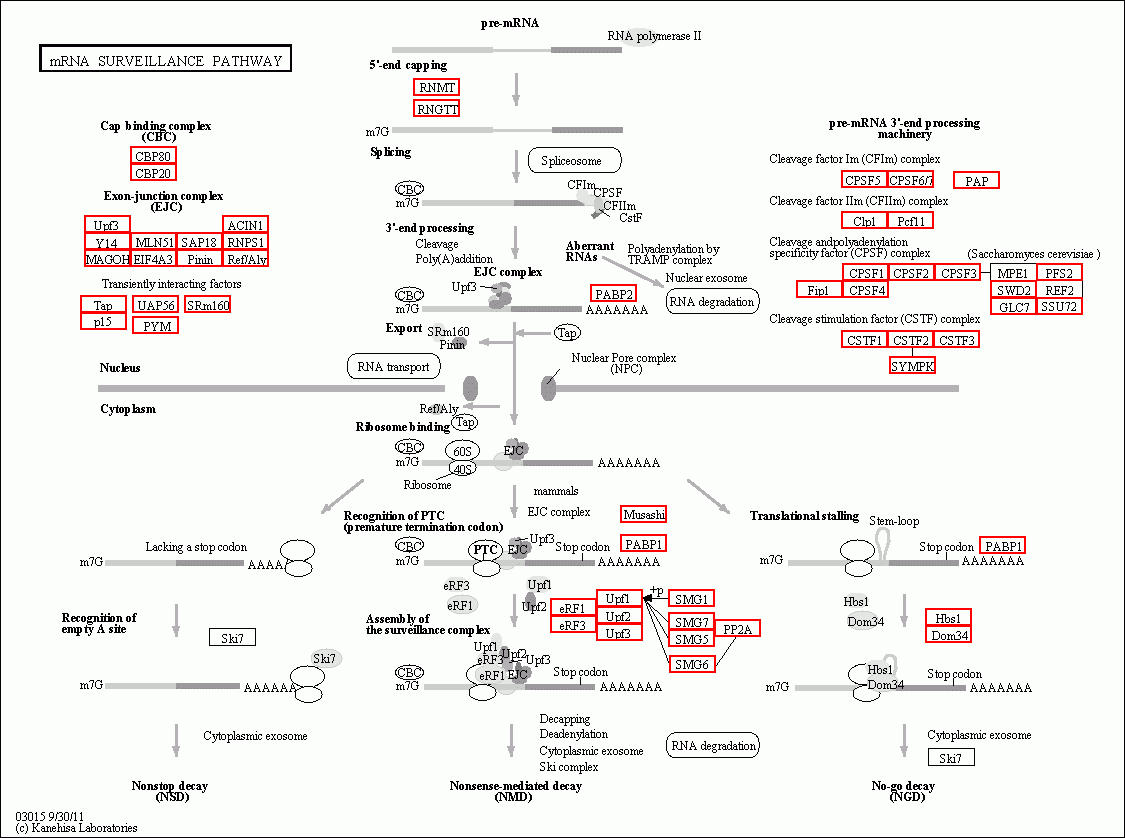

Supplement: Additional file 3: — Pathways found in the annotated portion of the transcriptomes. (ZIP 4950 kb) [file 12864_2015_1817_MOESM3_ESM.zip › map03015.png]

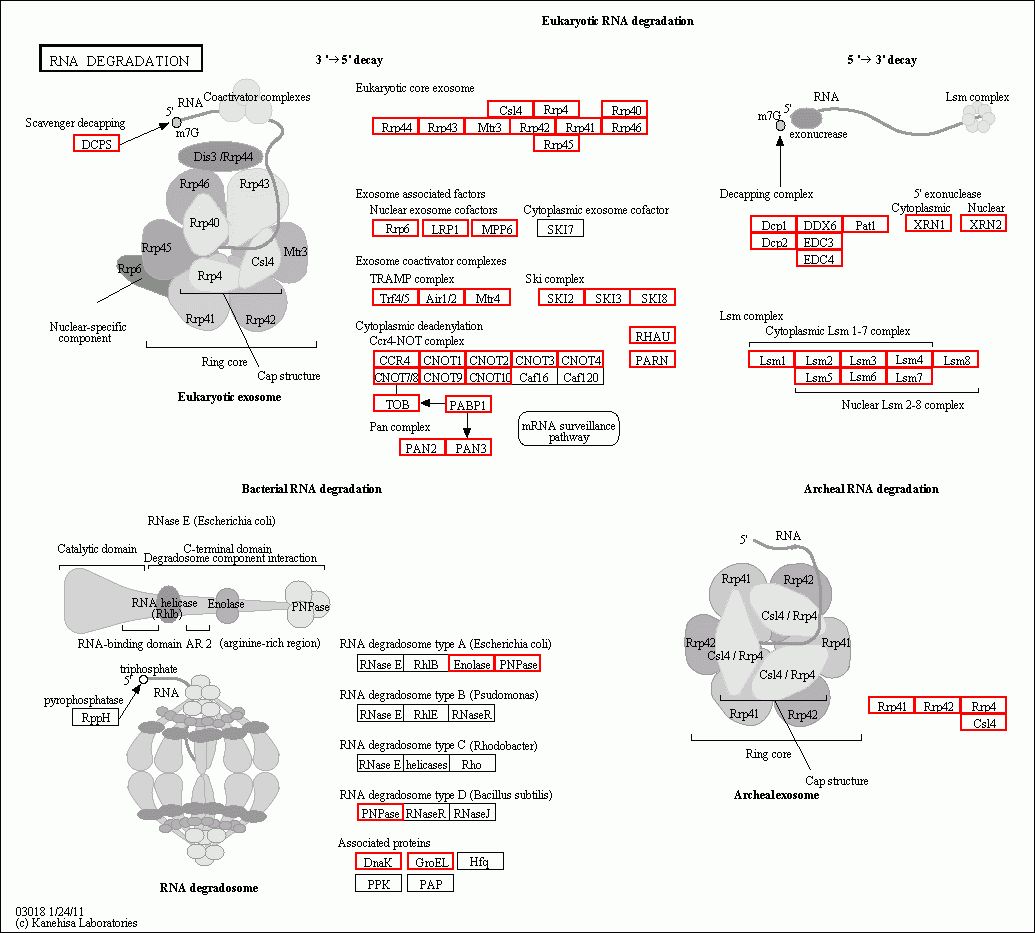

Supplement: Additional file 3: — Pathways found in the annotated portion of the transcriptomes. (ZIP 4950 kb) [file 12864_2015_1817_MOESM3_ESM.zip › map03018.png]

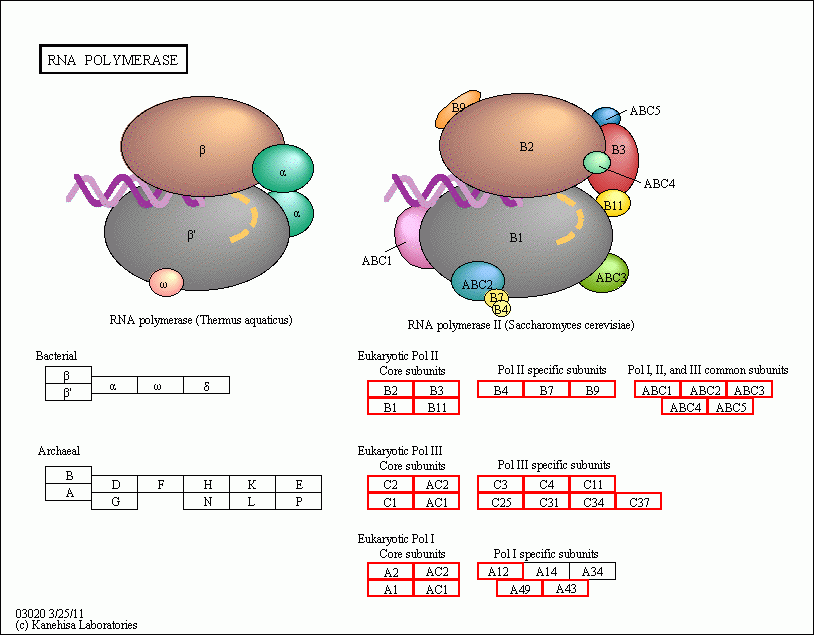

Supplement: Additional file 3: — Pathways found in the annotated portion of the transcriptomes. (ZIP 4950 kb) [file 12864_2015_1817_MOESM3_ESM.zip › map03020.png]

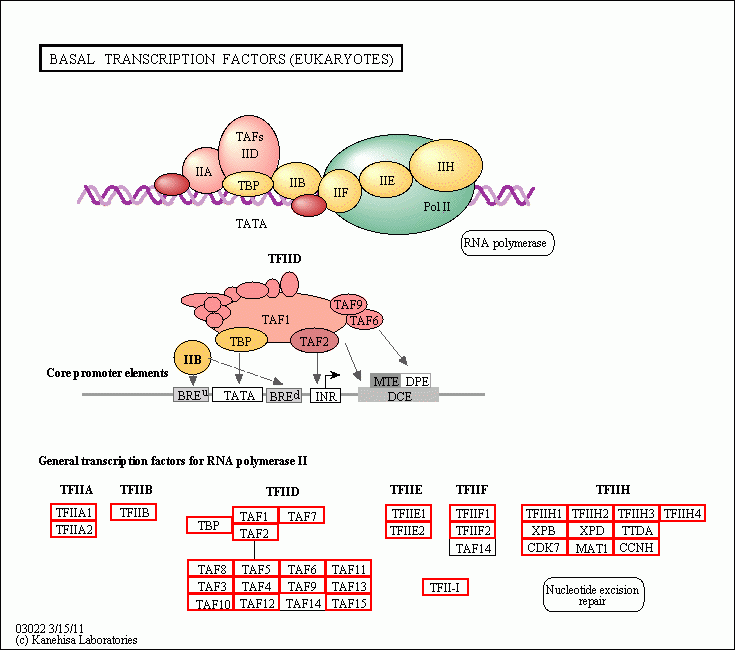

Supplement: Additional file 3: — Pathways found in the annotated portion of the transcriptomes. (ZIP 4950 kb) [file 12864_2015_1817_MOESM3_ESM.zip › map03022.png]

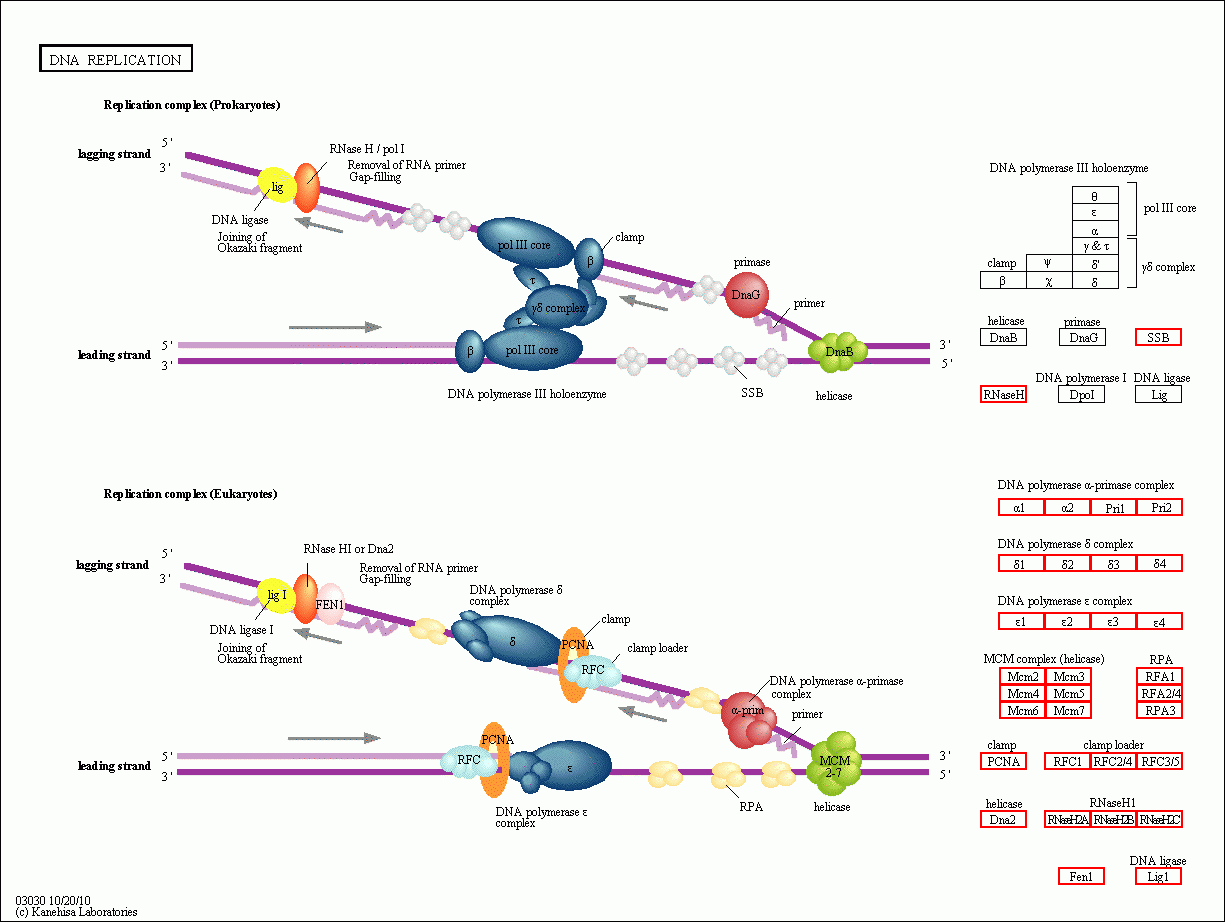

Supplement: Additional file 3: — Pathways found in the annotated portion of the transcriptomes. (ZIP 4950 kb) [file 12864_2015_1817_MOESM3_ESM.zip › map03030.png]

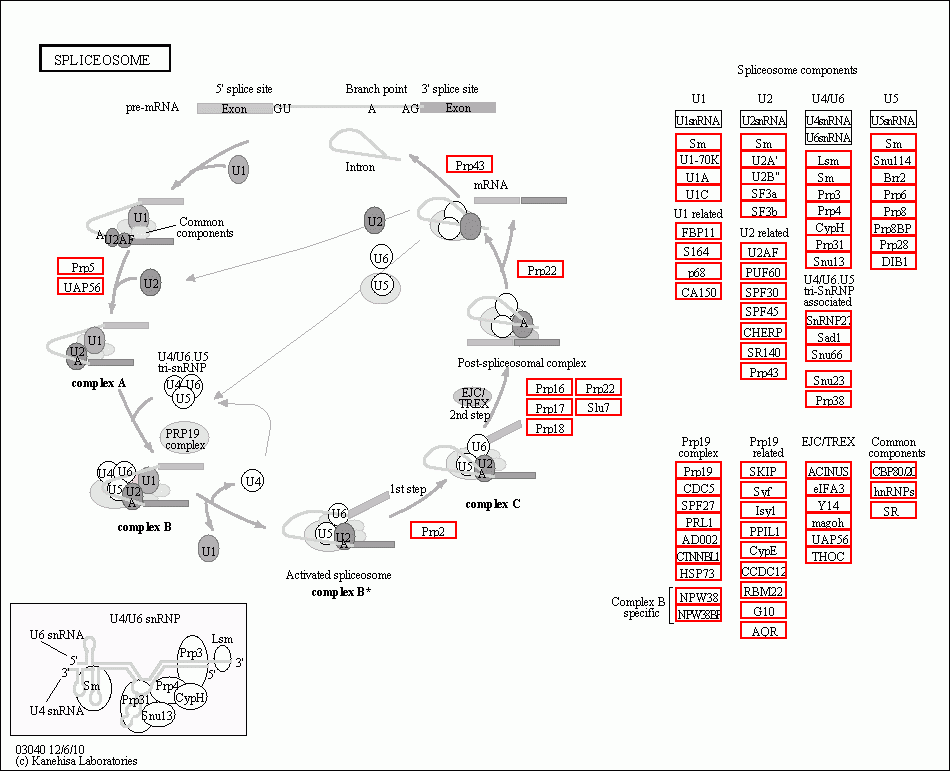

Supplement: Additional file 3: — Pathways found in the annotated portion of the transcriptomes. (ZIP 4950 kb) [file 12864_2015_1817_MOESM3_ESM.zip › map03040.png]

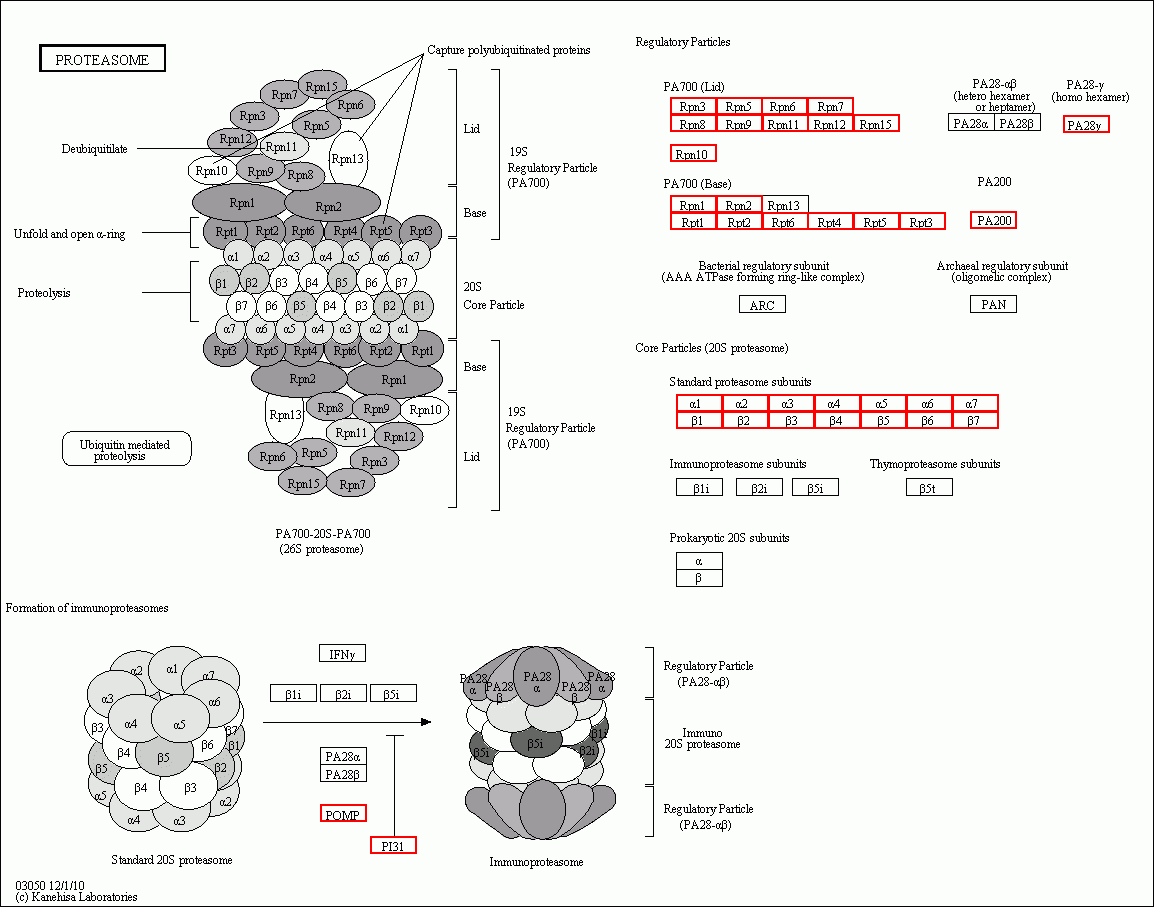

Supplement: Additional file 3: — Pathways found in the annotated portion of the transcriptomes. (ZIP 4950 kb) [file 12864_2015_1817_MOESM3_ESM.zip › map03050.png]

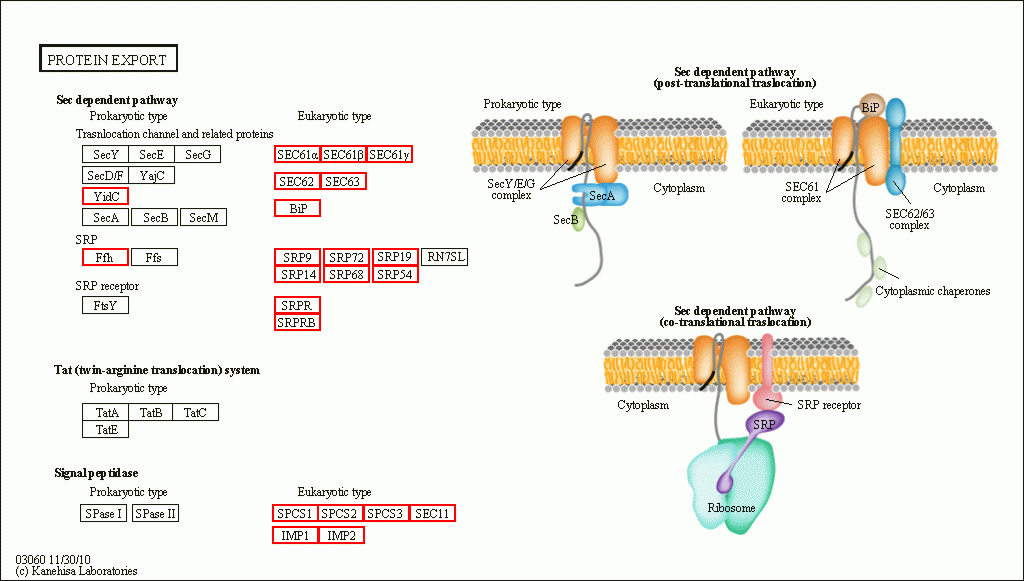

Supplement: Additional file 3: — Pathways found in the annotated portion of the transcriptomes. (ZIP 4950 kb) [file 12864_2015_1817_MOESM3_ESM.zip › map03060.png]

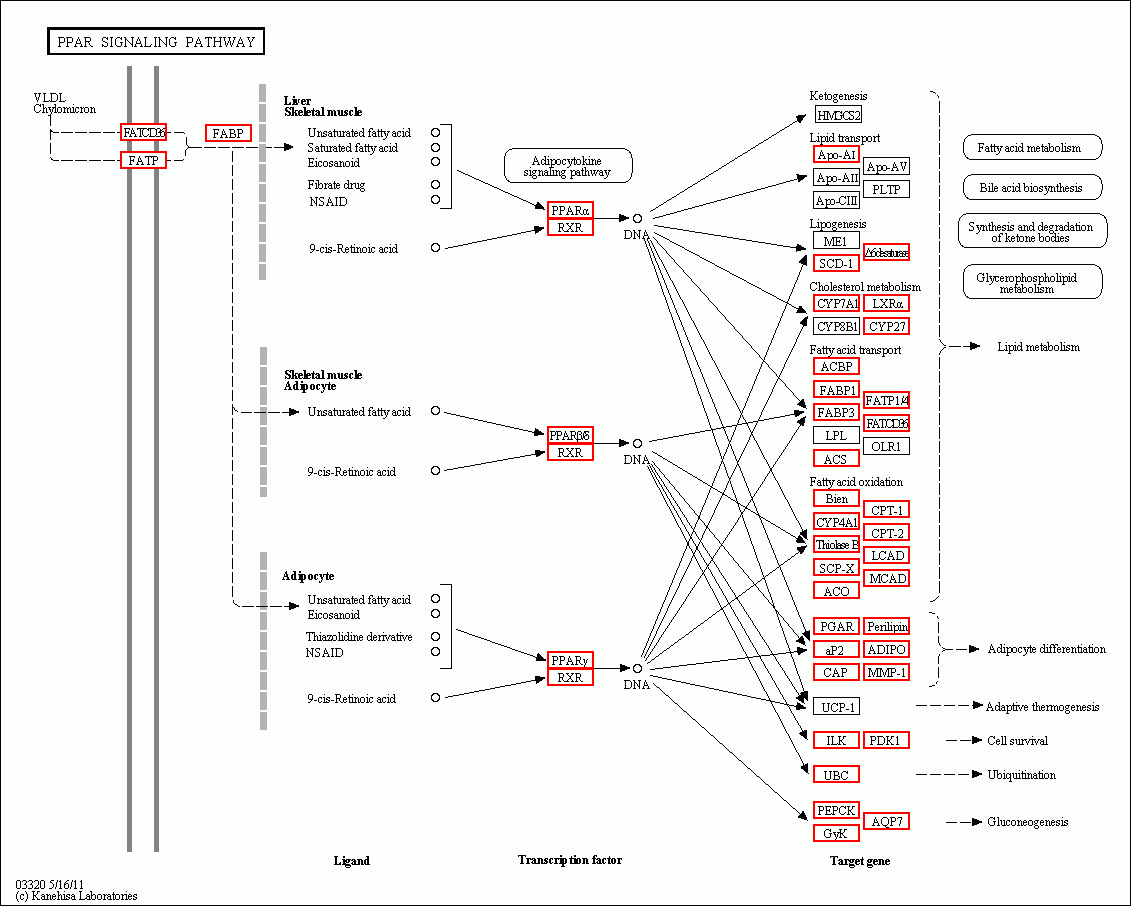

Supplement: Additional file 3: — Pathways found in the annotated portion of the transcriptomes. (ZIP 4950 kb) [file 12864_2015_1817_MOESM3_ESM.zip › map03320.png]

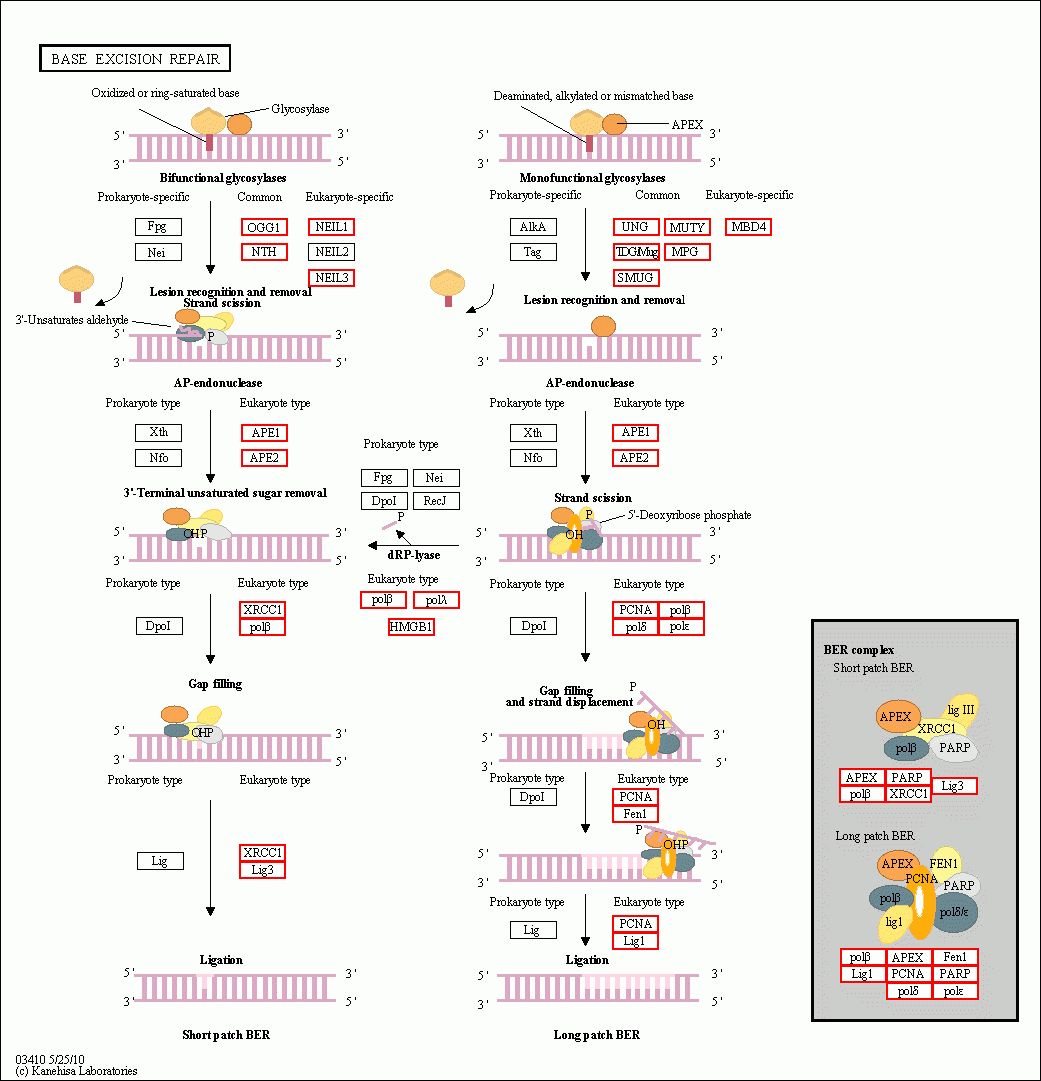

Supplement: Additional file 3: — Pathways found in the annotated portion of the transcriptomes. (ZIP 4950 kb) [file 12864_2015_1817_MOESM3_ESM.zip › map03410.png]

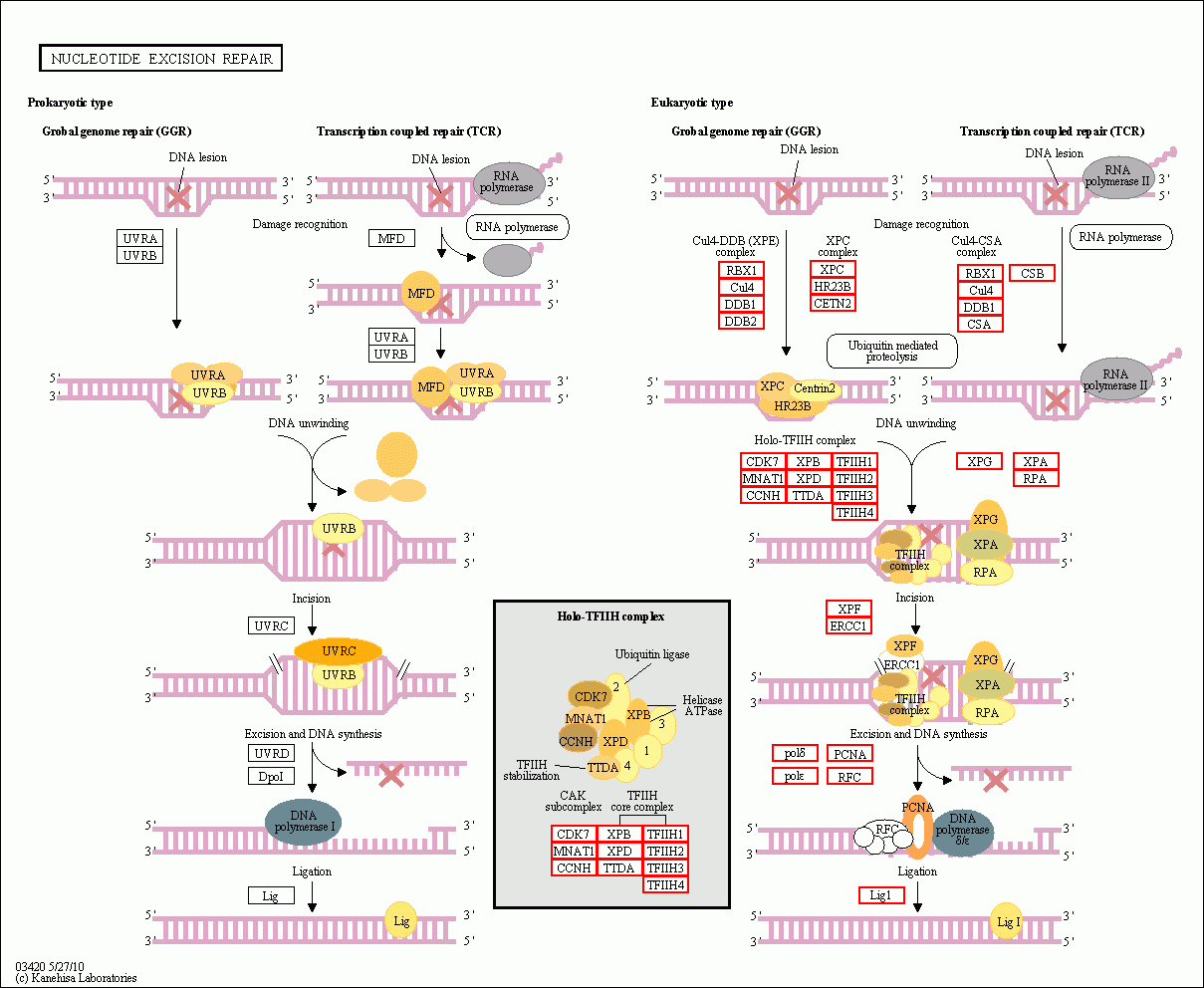

Supplement: Additional file 3: — Pathways found in the annotated portion of the transcriptomes. (ZIP 4950 kb) [file 12864_2015_1817_MOESM3_ESM.zip › map03420.png]

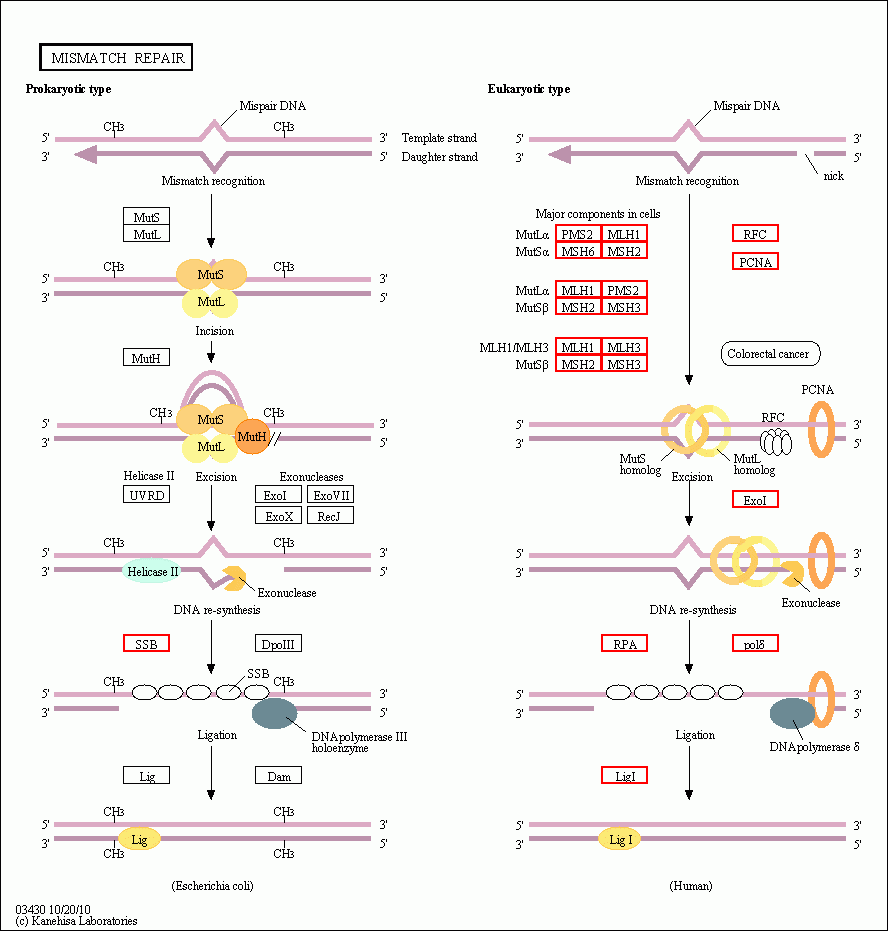

Supplement: Additional file 3: — Pathways found in the annotated portion of the transcriptomes. (ZIP 4950 kb) [file 12864_2015_1817_MOESM3_ESM.zip › map03430.png]

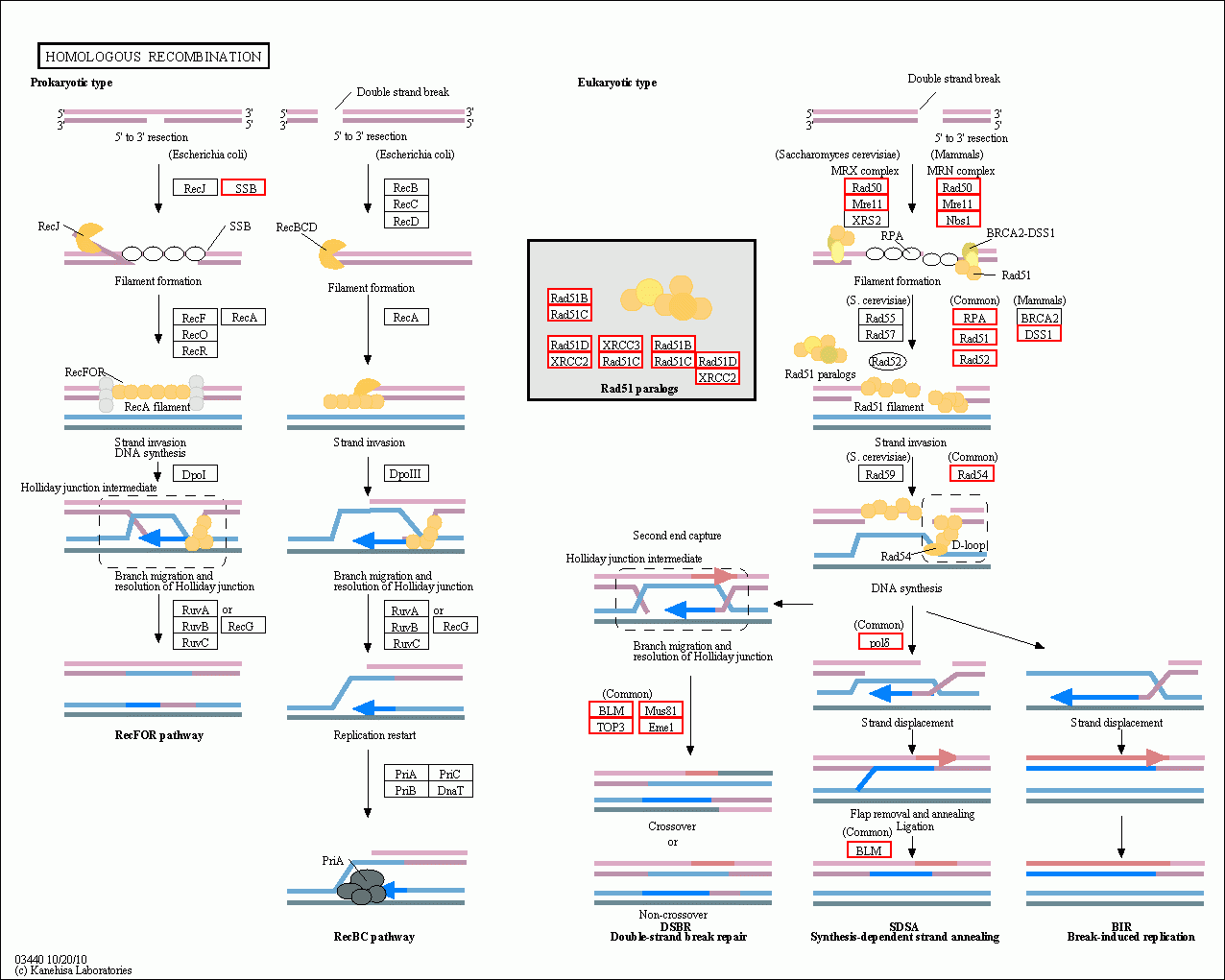

Supplement: Additional file 3: — Pathways found in the annotated portion of the transcriptomes. (ZIP 4950 kb) [file 12864_2015_1817_MOESM3_ESM.zip › map03440.png]

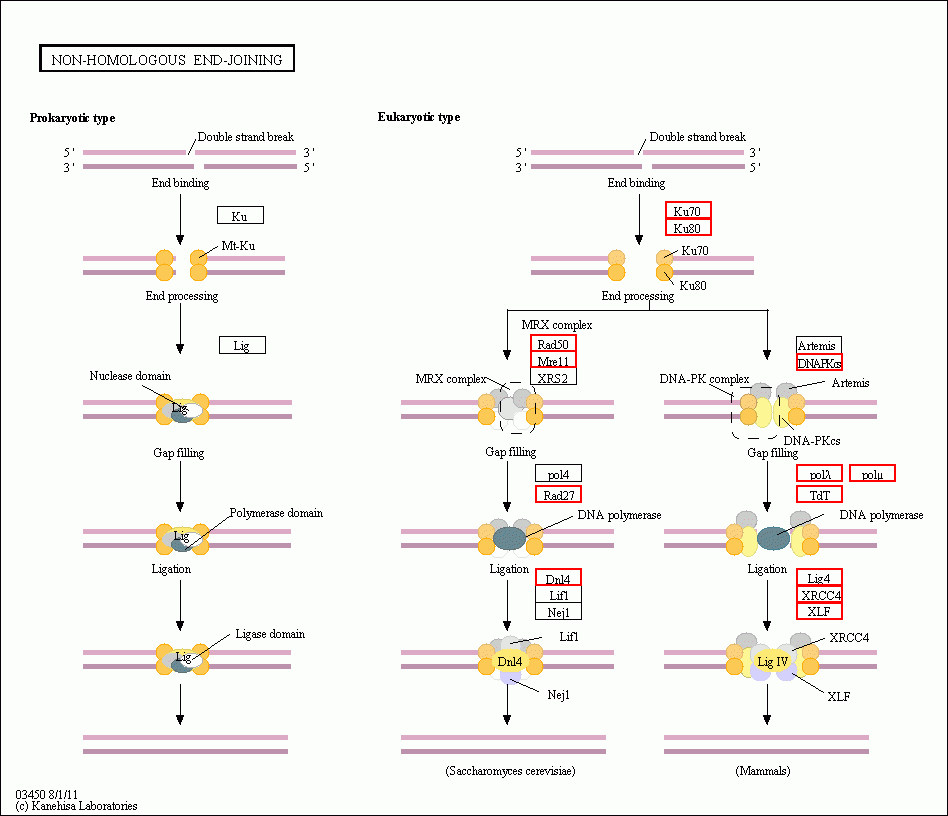

Supplement: Additional file 3: — Pathways found in the annotated portion of the transcriptomes. (ZIP 4950 kb) [file 12864_2015_1817_MOESM3_ESM.zip › map03450.png]

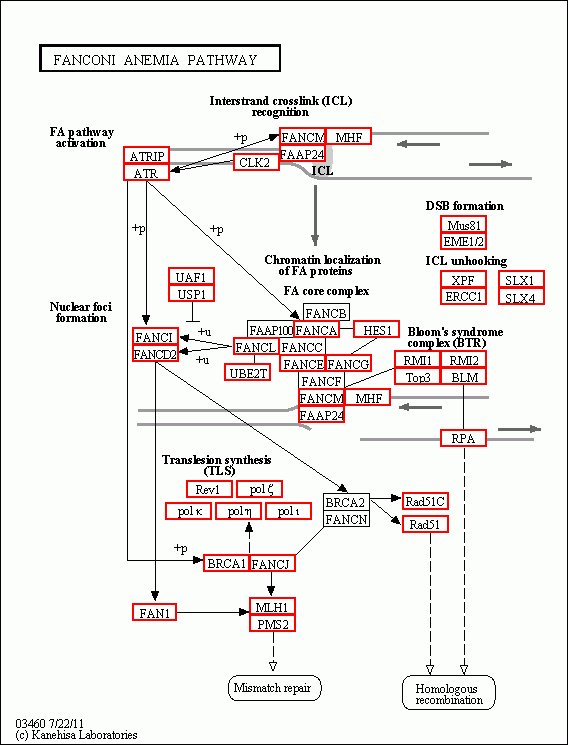

Supplement: Additional file 3: — Pathways found in the annotated portion of the transcriptomes. (ZIP 4950 kb) [file 12864_2015_1817_MOESM3_ESM.zip › map03460.png]

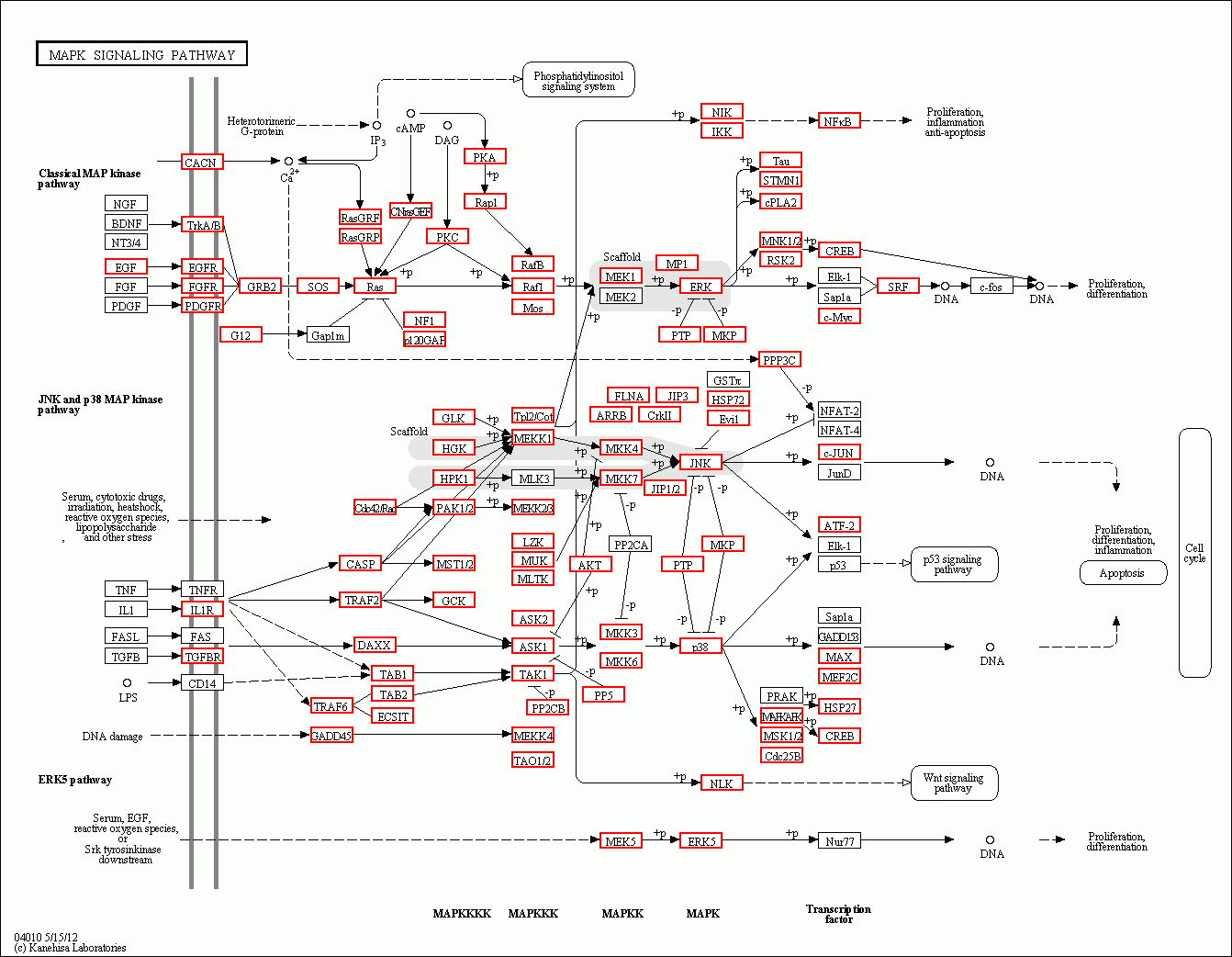

Supplement: Additional file 3: — Pathways found in the annotated portion of the transcriptomes. (ZIP 4950 kb) [file 12864_2015_1817_MOESM3_ESM.zip › map04010.png]

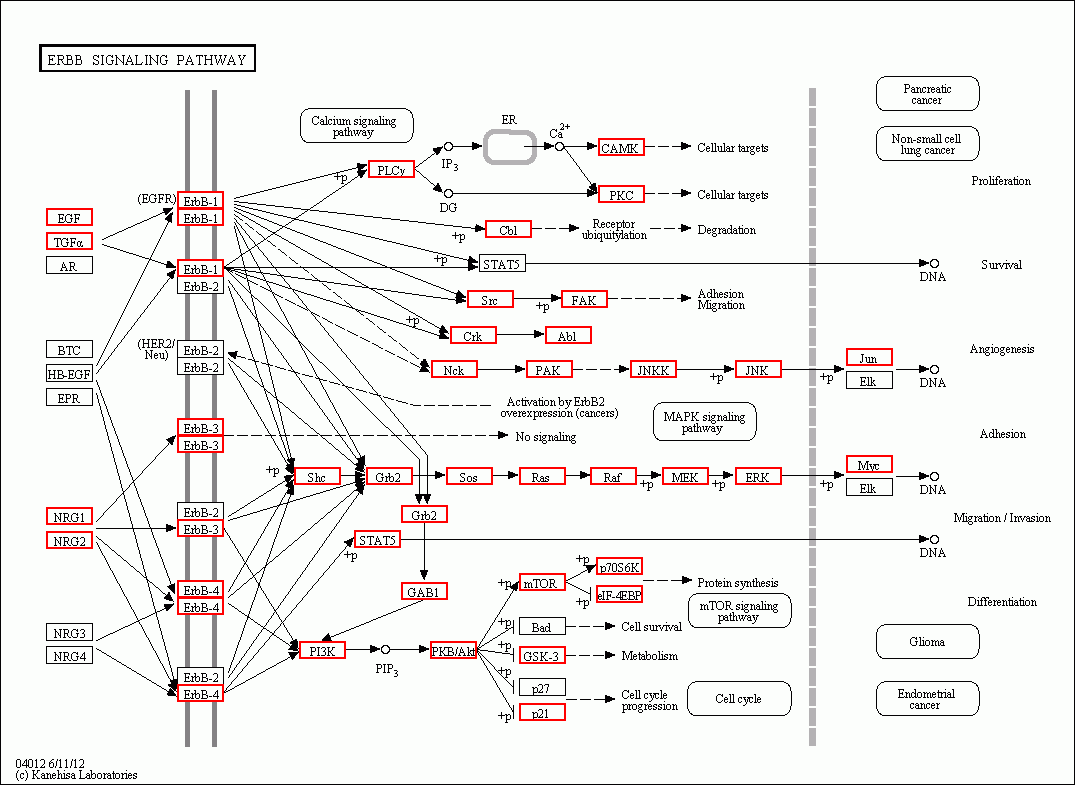

Supplement: Additional file 3: — Pathways found in the annotated portion of the transcriptomes. (ZIP 4950 kb) [file 12864_2015_1817_MOESM3_ESM.zip › map04012.png]

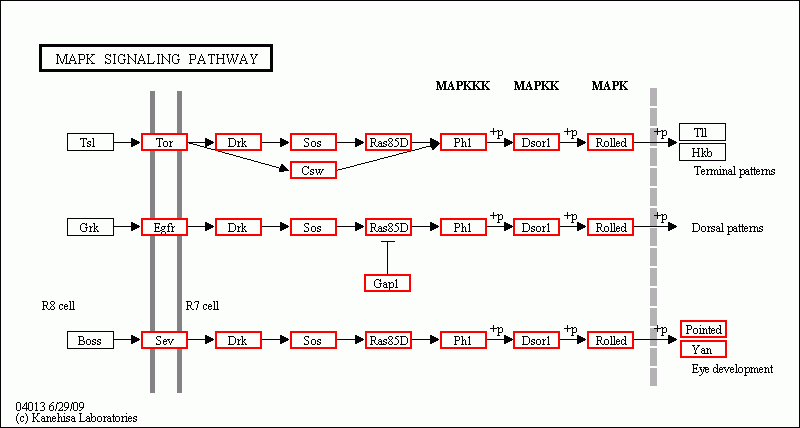

Supplement: Additional file 3: — Pathways found in the annotated portion of the transcriptomes. (ZIP 4950 kb) [file 12864_2015_1817_MOESM3_ESM.zip › map04013.png]

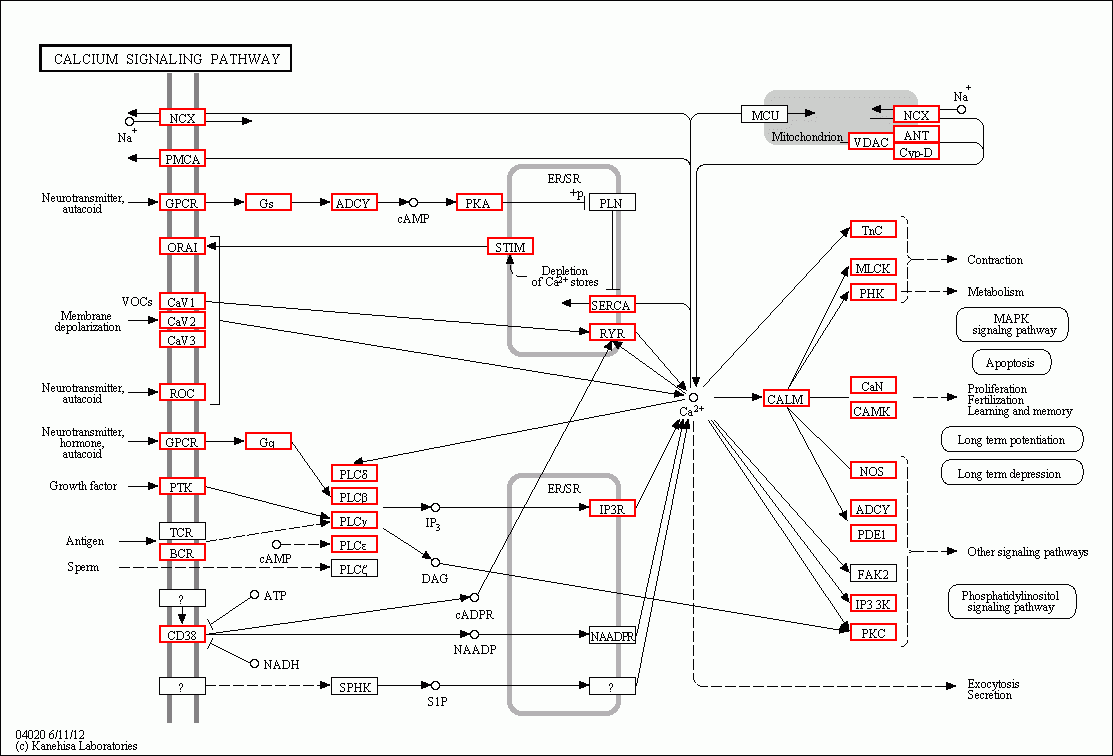

Supplement: Additional file 3: — Pathways found in the annotated portion of the transcriptomes. (ZIP 4950 kb) [file 12864_2015_1817_MOESM3_ESM.zip › map04020.png]

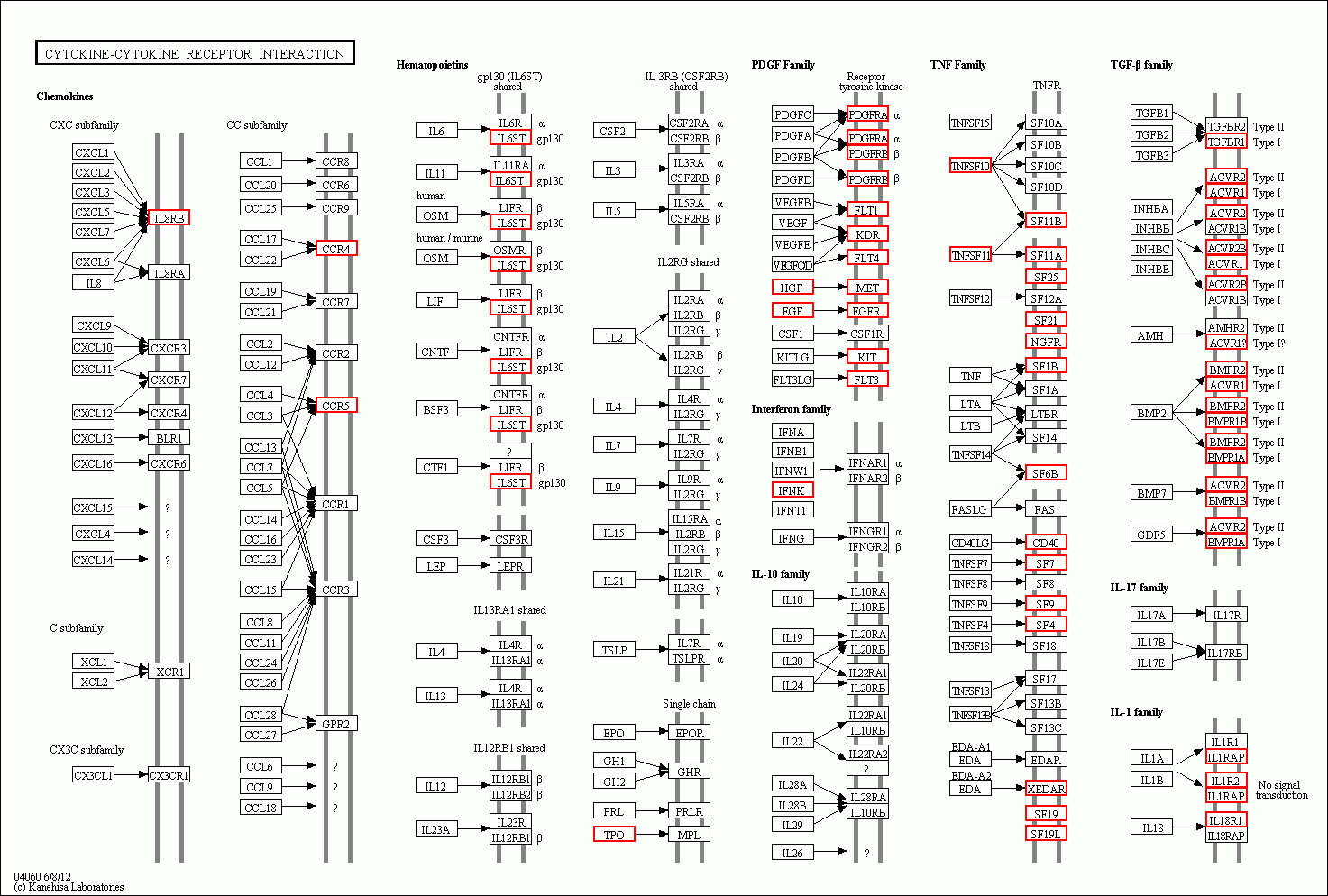

Supplement: Additional file 3: — Pathways found in the annotated portion of the transcriptomes. (ZIP 4950 kb) [file 12864_2015_1817_MOESM3_ESM.zip › map04060.png]

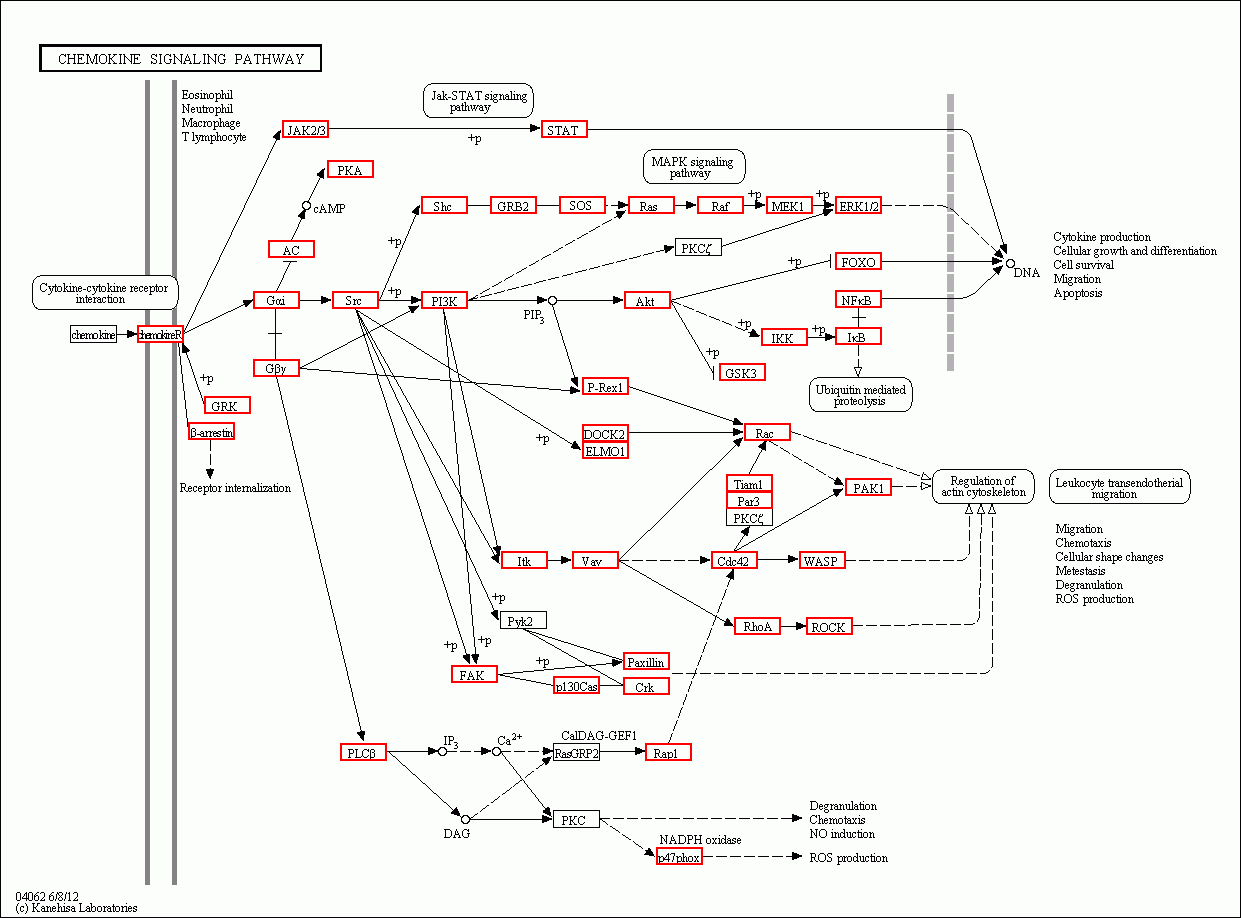

Supplement: Additional file 3: — Pathways found in the annotated portion of the transcriptomes. (ZIP 4950 kb) [file 12864_2015_1817_MOESM3_ESM.zip › map04062.png]

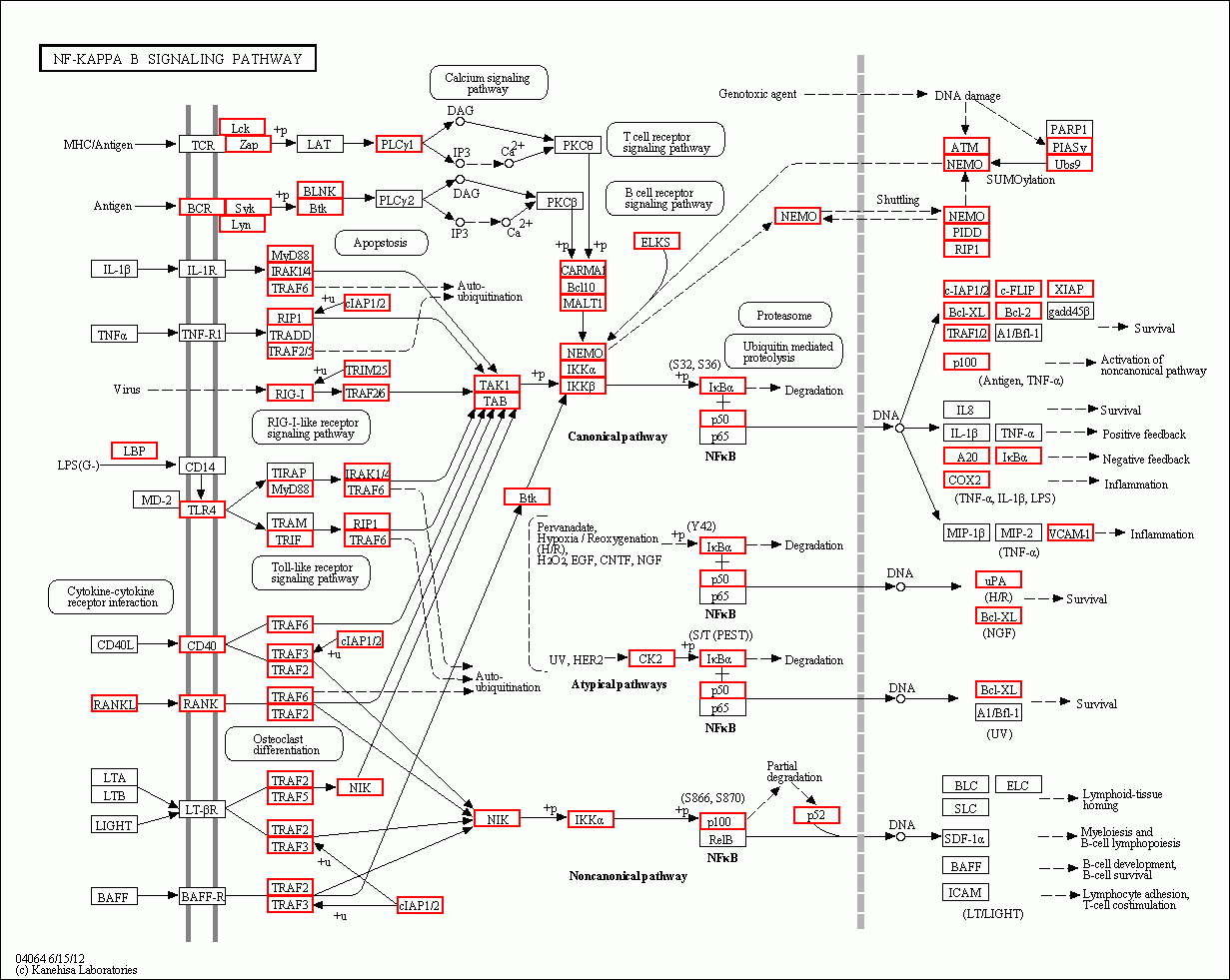

Supplement: Additional file 3: — Pathways found in the annotated portion of the transcriptomes. (ZIP 4950 kb) [file 12864_2015_1817_MOESM3_ESM.zip › map04064.png]

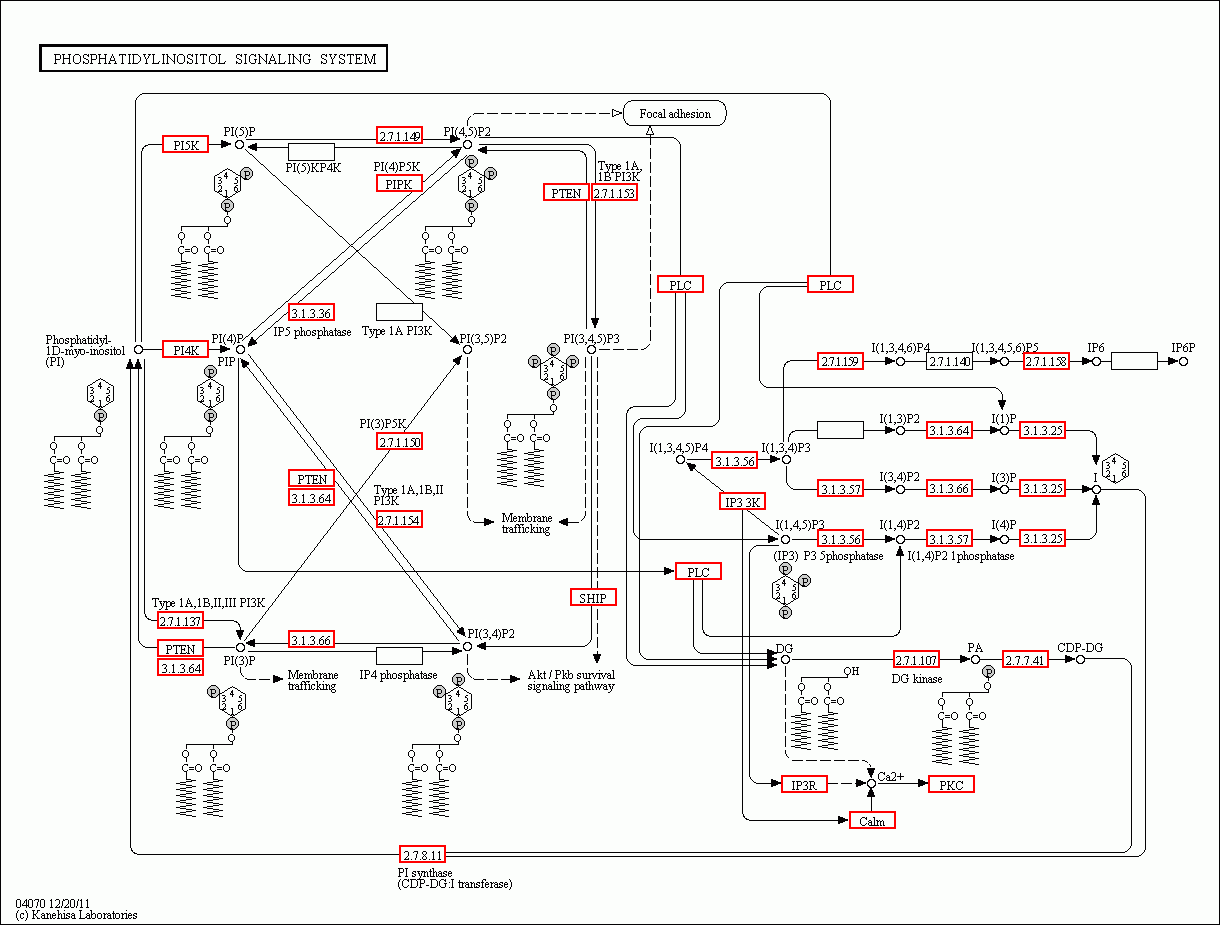

Supplement: Additional file 3: — Pathways found in the annotated portion of the transcriptomes. (ZIP 4950 kb) [file 12864_2015_1817_MOESM3_ESM.zip › map04070.png]

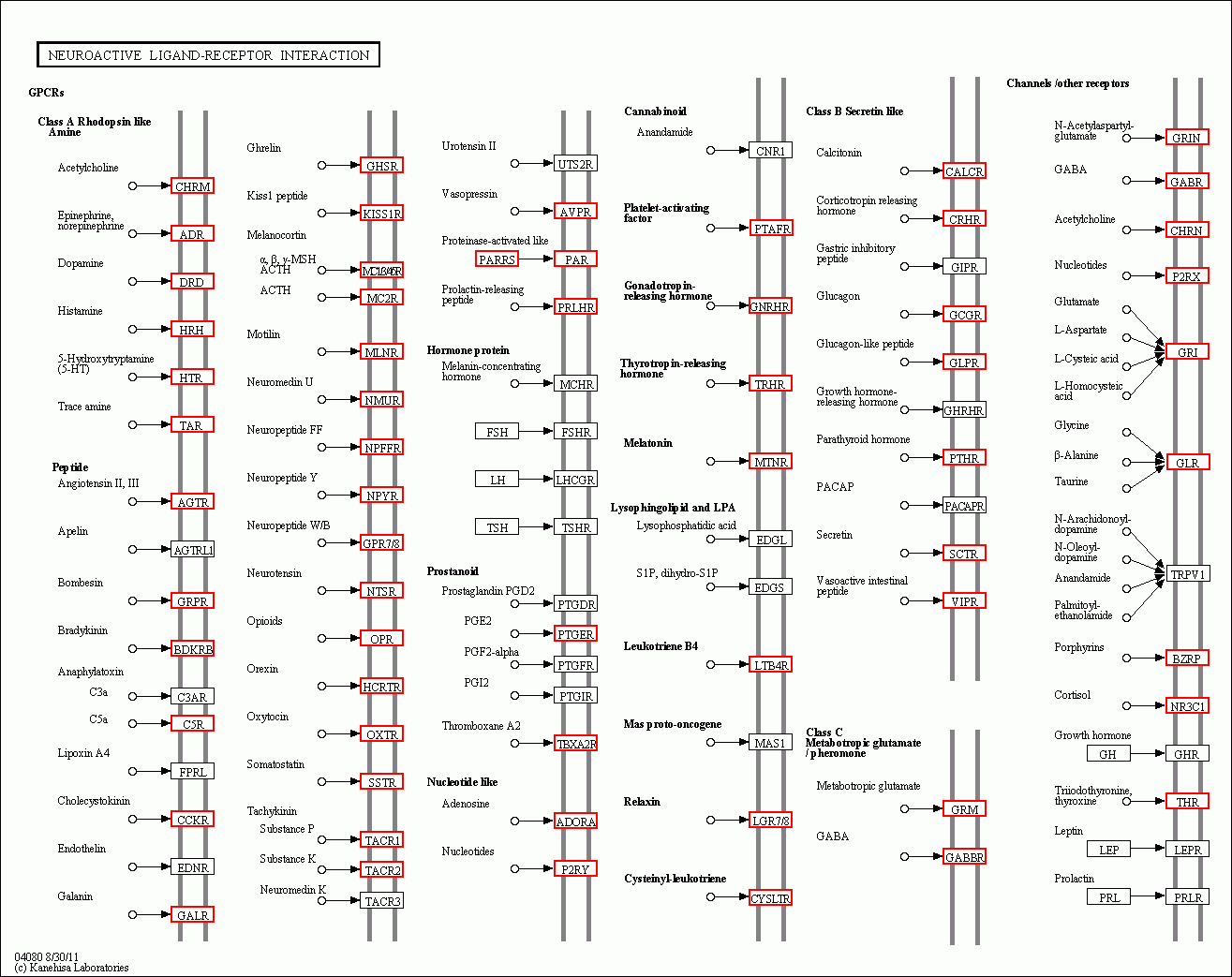

Supplement: Additional file 3: — Pathways found in the annotated portion of the transcriptomes. (ZIP 4950 kb) [file 12864_2015_1817_MOESM3_ESM.zip › map04080.png]

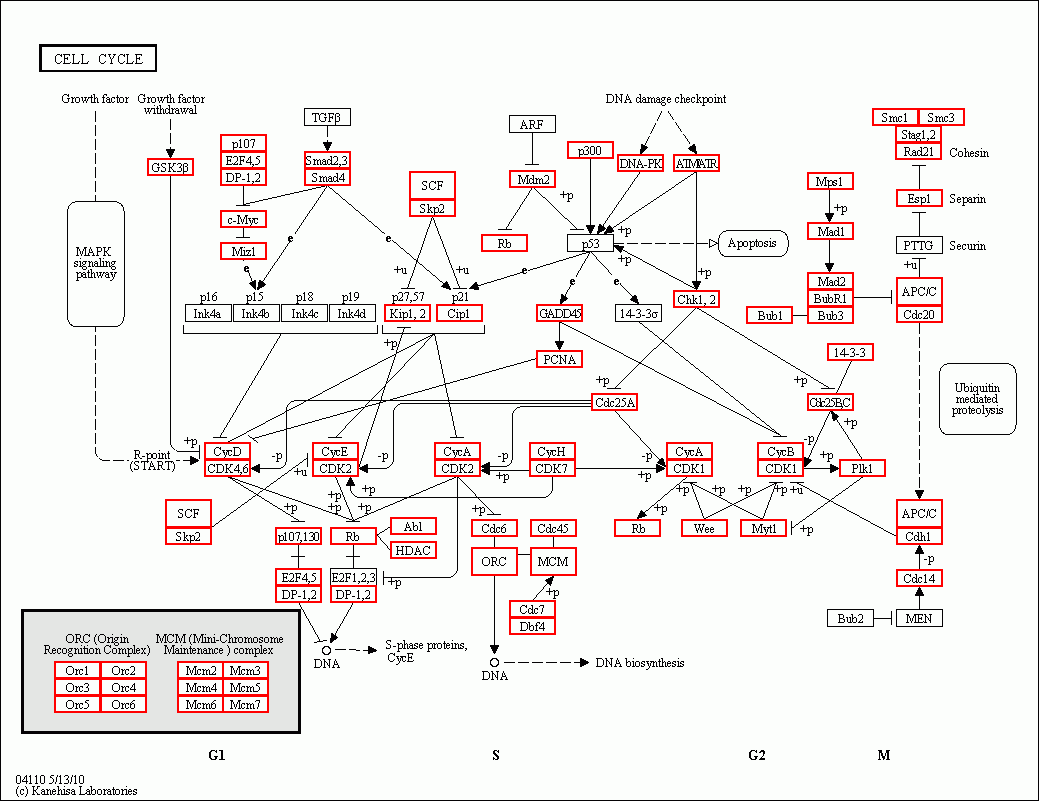

Supplement: Additional file 3: — Pathways found in the annotated portion of the transcriptomes. (ZIP 4950 kb) [file 12864_2015_1817_MOESM3_ESM.zip › map04110.png]

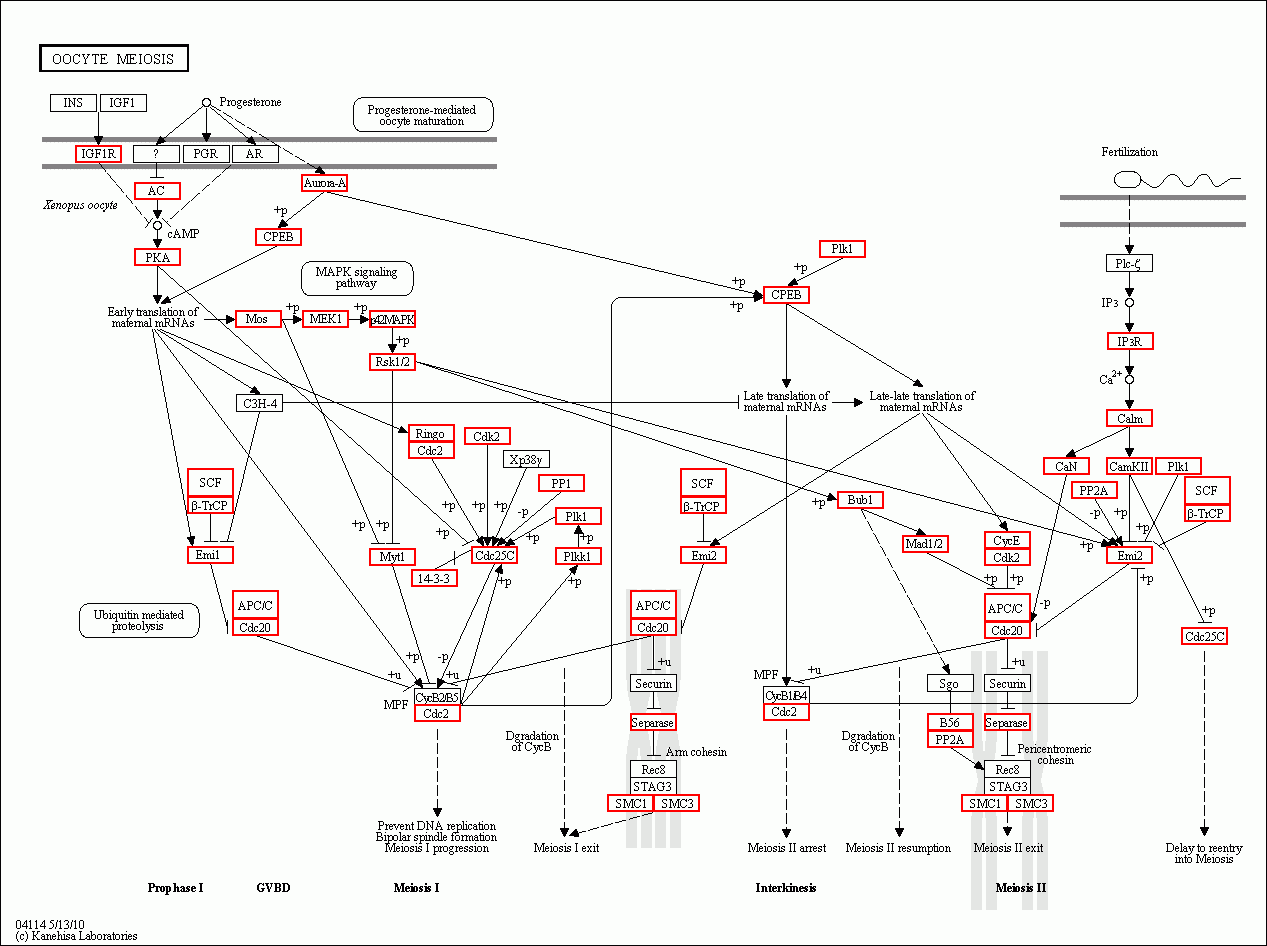

Supplement: Additional file 3: — Pathways found in the annotated portion of the transcriptomes. (ZIP 4950 kb) [file 12864_2015_1817_MOESM3_ESM.zip › map04114.png]

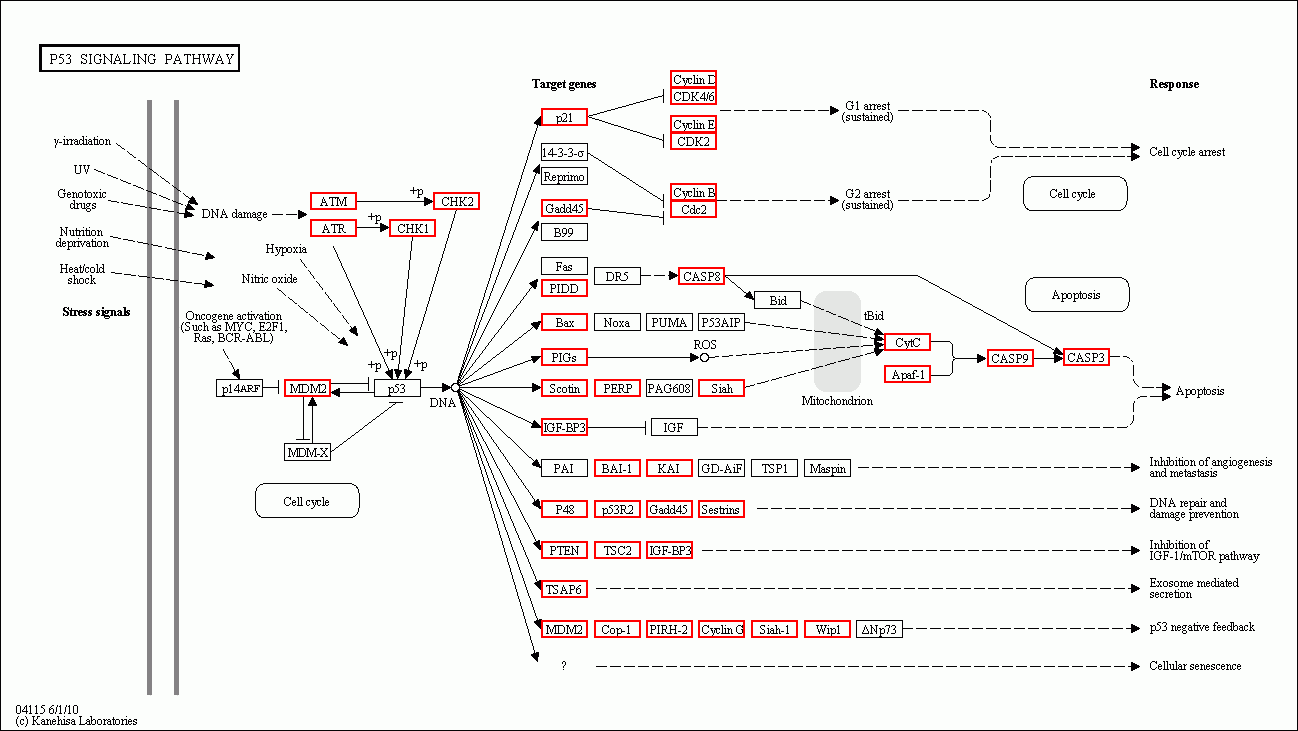

Supplement: Additional file 3: — Pathways found in the annotated portion of the transcriptomes. (ZIP 4950 kb) [file 12864_2015_1817_MOESM3_ESM.zip › map04115.png]

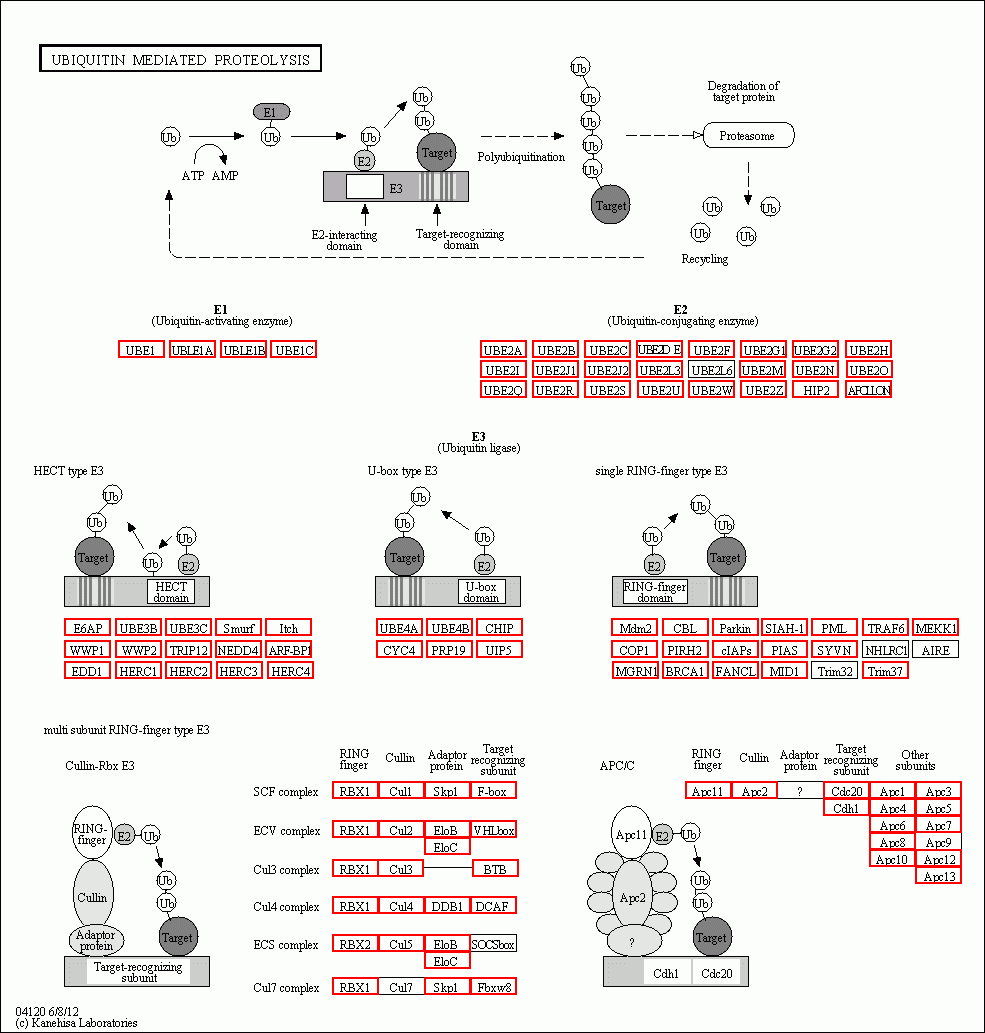

Supplement: Additional file 3: — Pathways found in the annotated portion of the transcriptomes. (ZIP 4950 kb) [file 12864_2015_1817_MOESM3_ESM.zip › map04120.png]

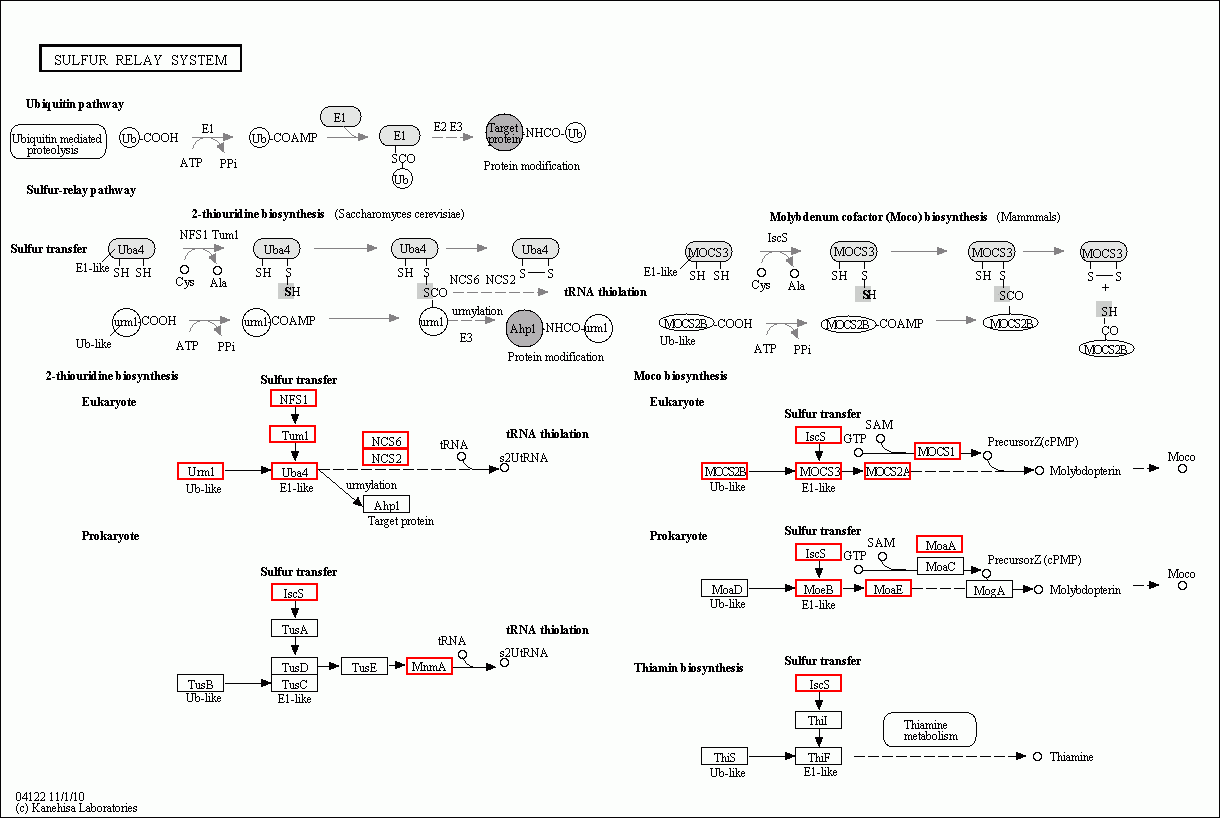

Supplement: Additional file 3: — Pathways found in the annotated portion of the transcriptomes. (ZIP 4950 kb) [file 12864_2015_1817_MOESM3_ESM.zip › map04122.png]

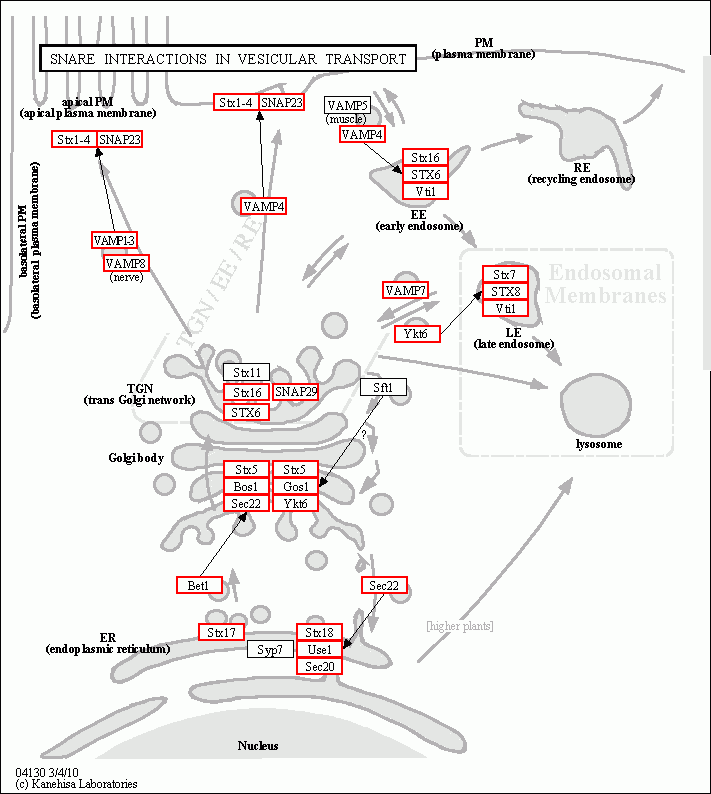

Supplement: Additional file 3: — Pathways found in the annotated portion of the transcriptomes. (ZIP 4950 kb) [file 12864_2015_1817_MOESM3_ESM.zip › map04130.png]

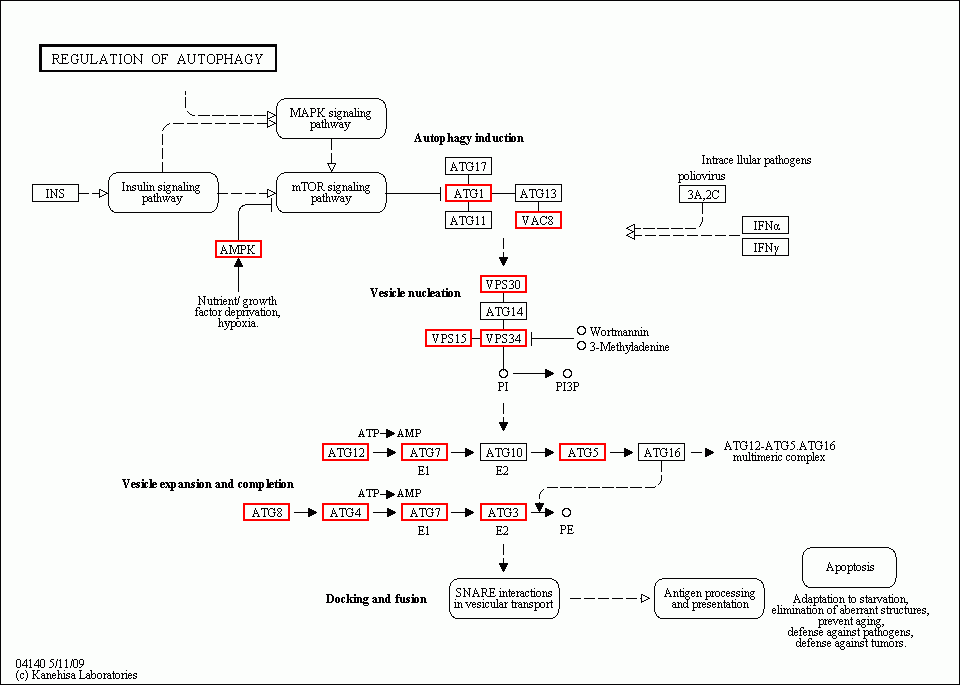

Supplement: Additional file 3: — Pathways found in the annotated portion of the transcriptomes. (ZIP 4950 kb) [file 12864_2015_1817_MOESM3_ESM.zip › map04140.png]

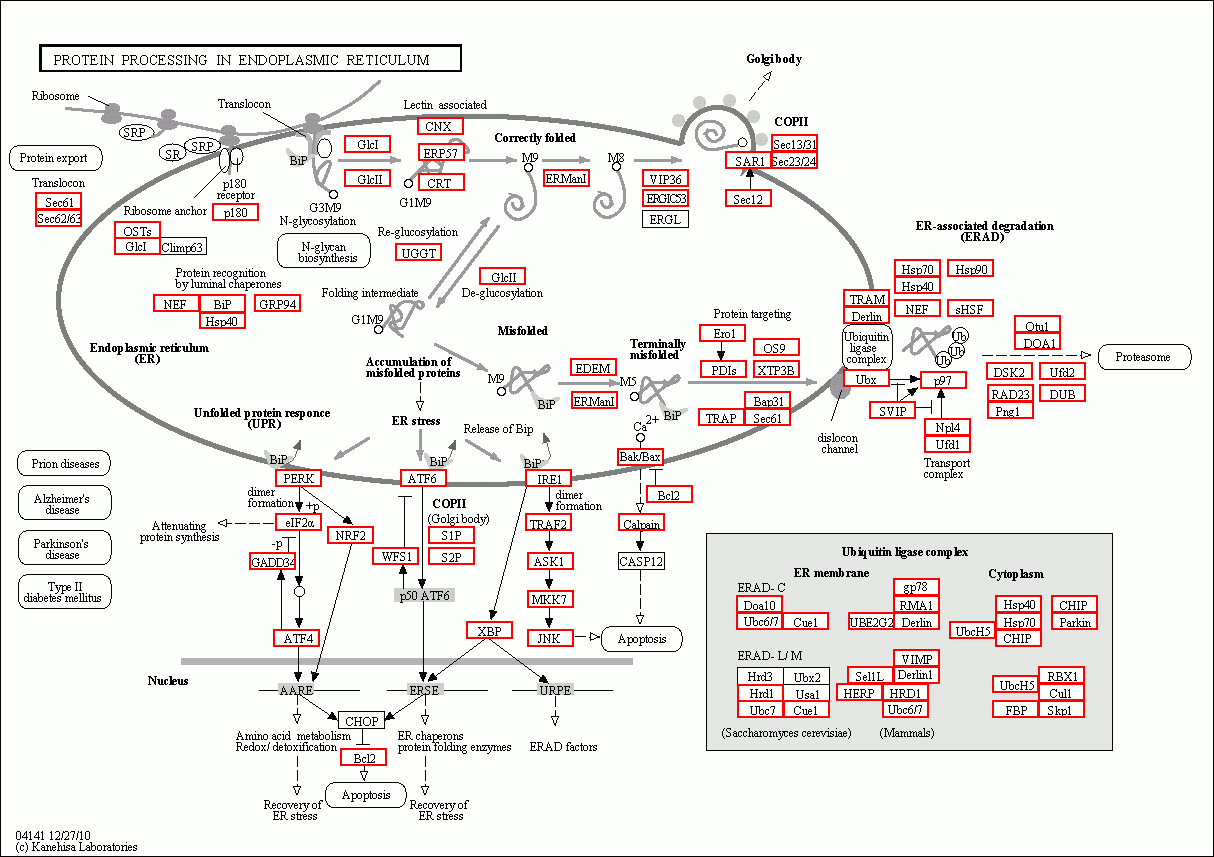

Supplement: Additional file 3: — Pathways found in the annotated portion of the transcriptomes. (ZIP 4950 kb) [file 12864_2015_1817_MOESM3_ESM.zip › map04141.png]

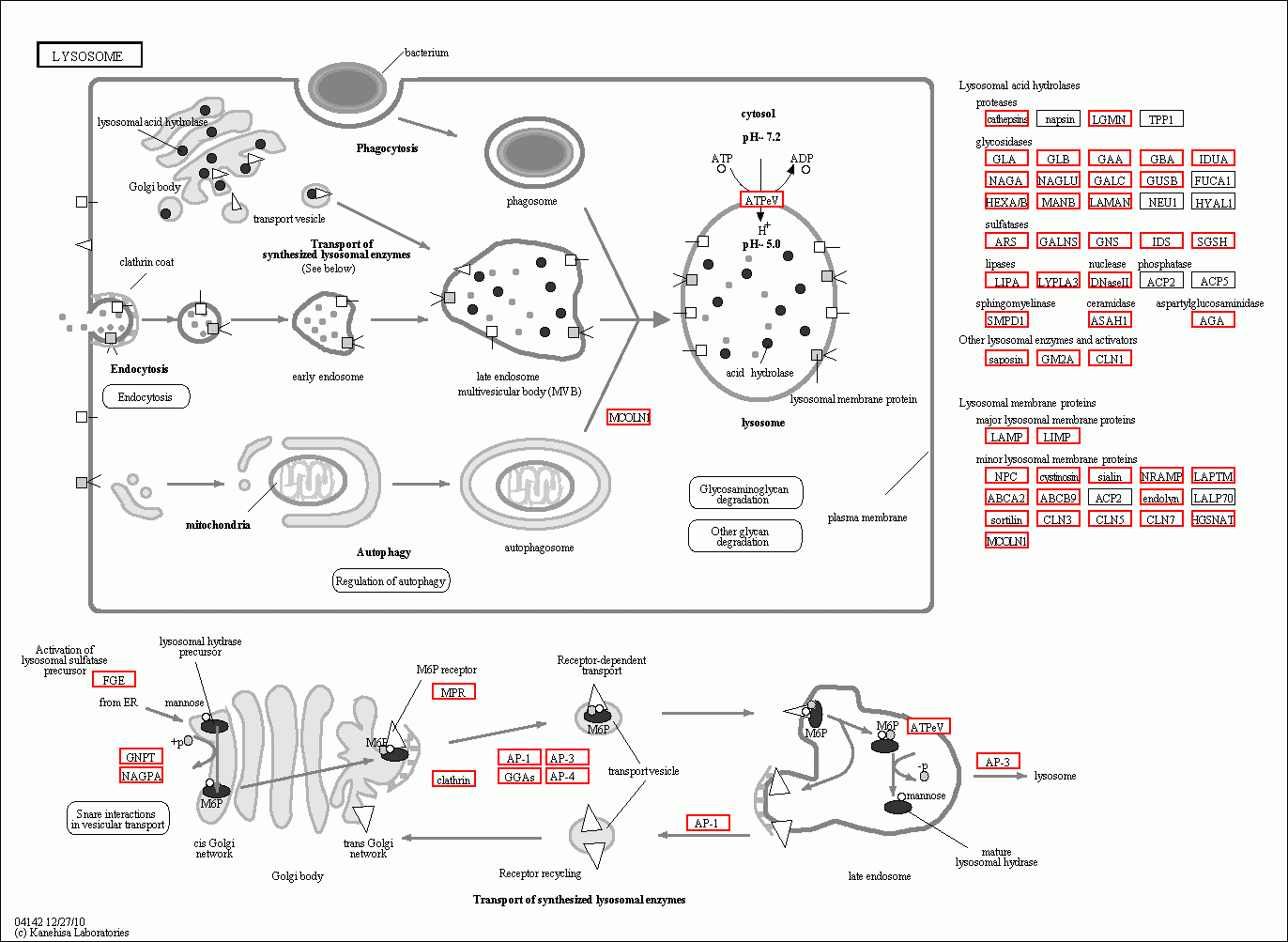

Supplement: Additional file 3: — Pathways found in the annotated portion of the transcriptomes. (ZIP 4950 kb) [file 12864_2015_1817_MOESM3_ESM.zip › map04142.png]

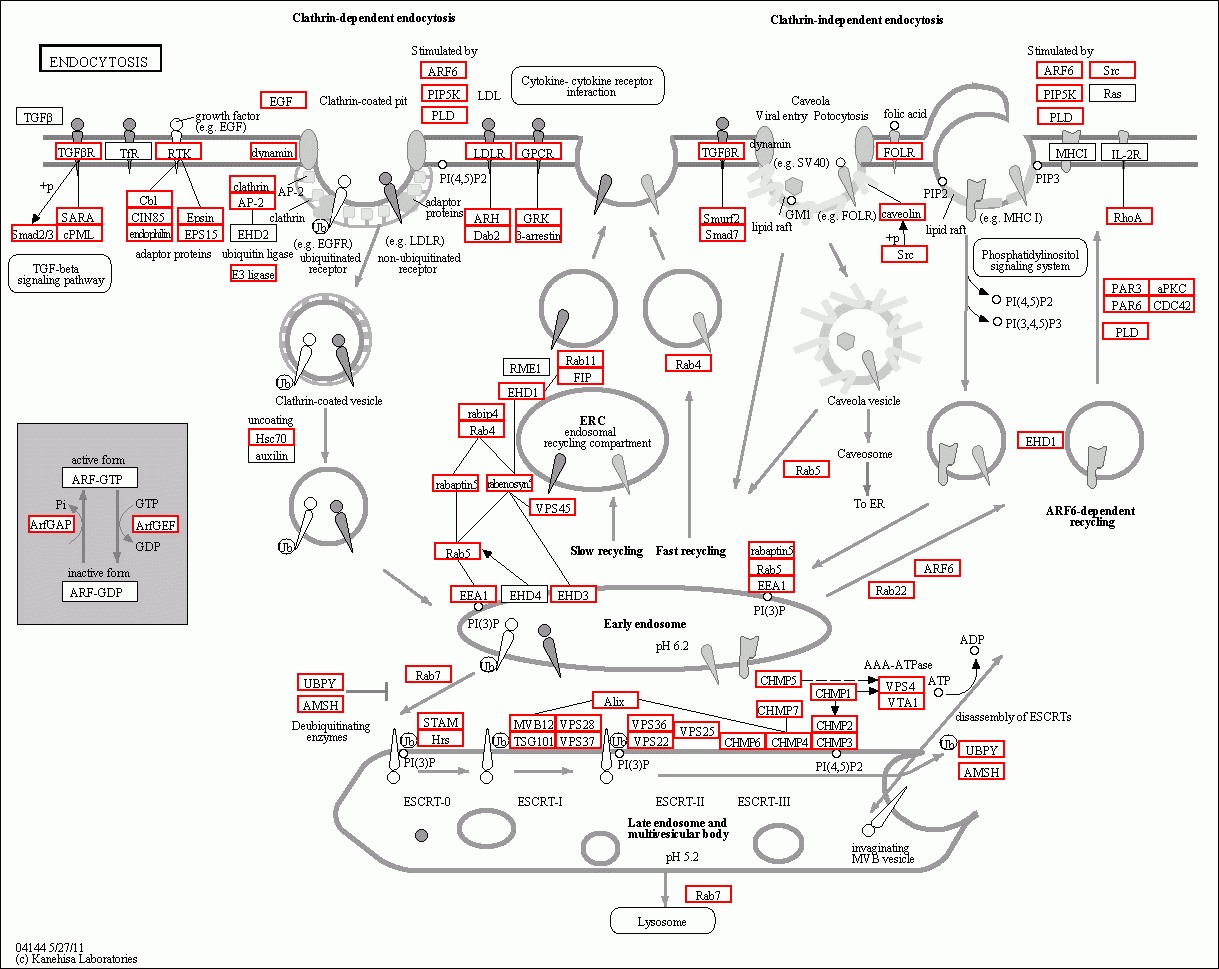

Supplement: Additional file 3: — Pathways found in the annotated portion of the transcriptomes. (ZIP 4950 kb) [file 12864_2015_1817_MOESM3_ESM.zip › map04144.png]

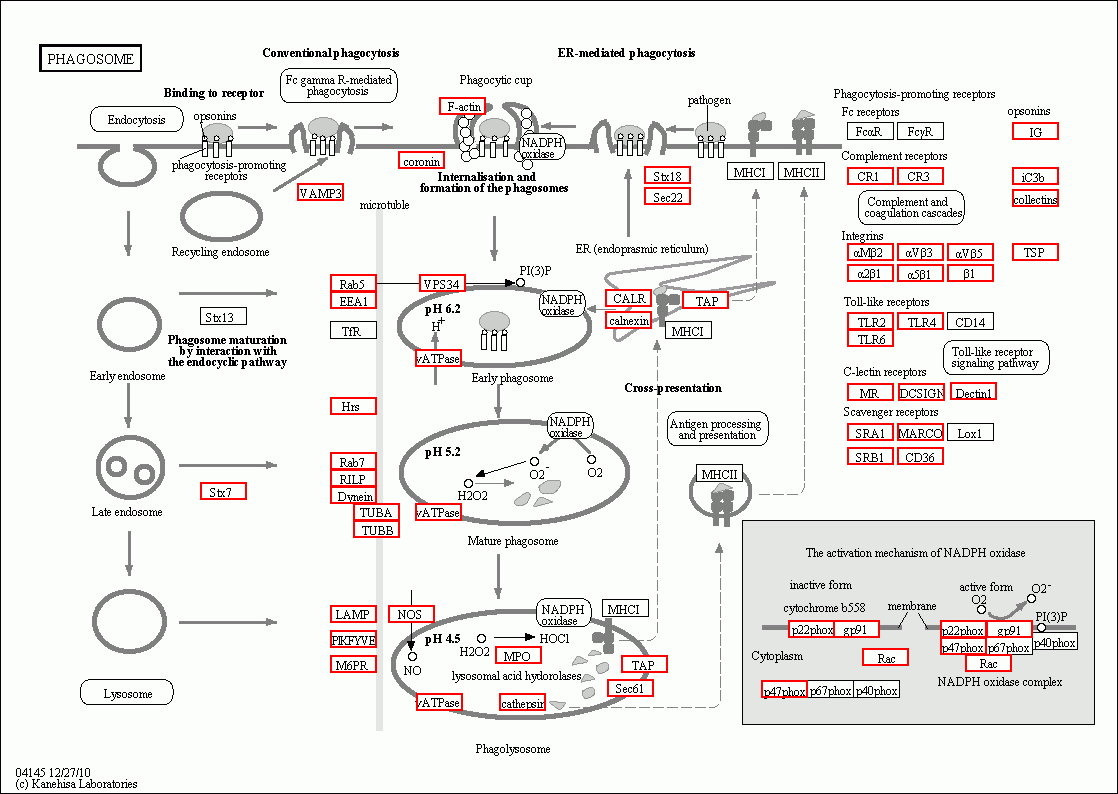

Supplement: Additional file 3: — Pathways found in the annotated portion of the transcriptomes. (ZIP 4950 kb) [file 12864_2015_1817_MOESM3_ESM.zip › map04145.png]

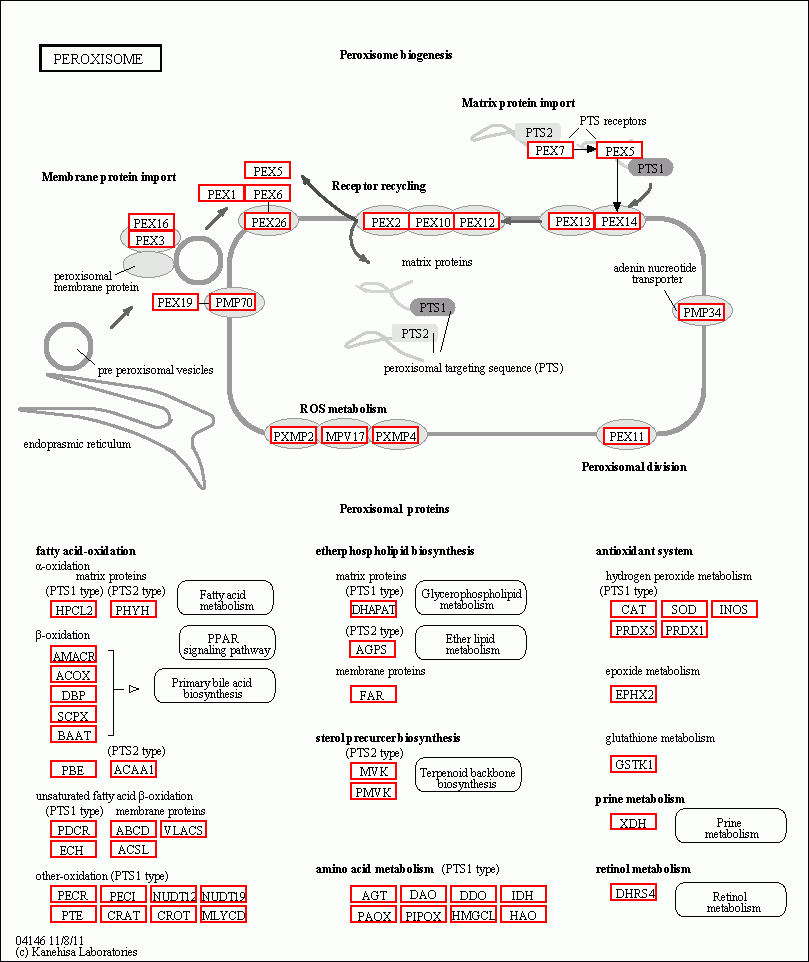

Supplement: Additional file 3: — Pathways found in the annotated portion of the transcriptomes. (ZIP 4950 kb) [file 12864_2015_1817_MOESM3_ESM.zip › map04146.png]

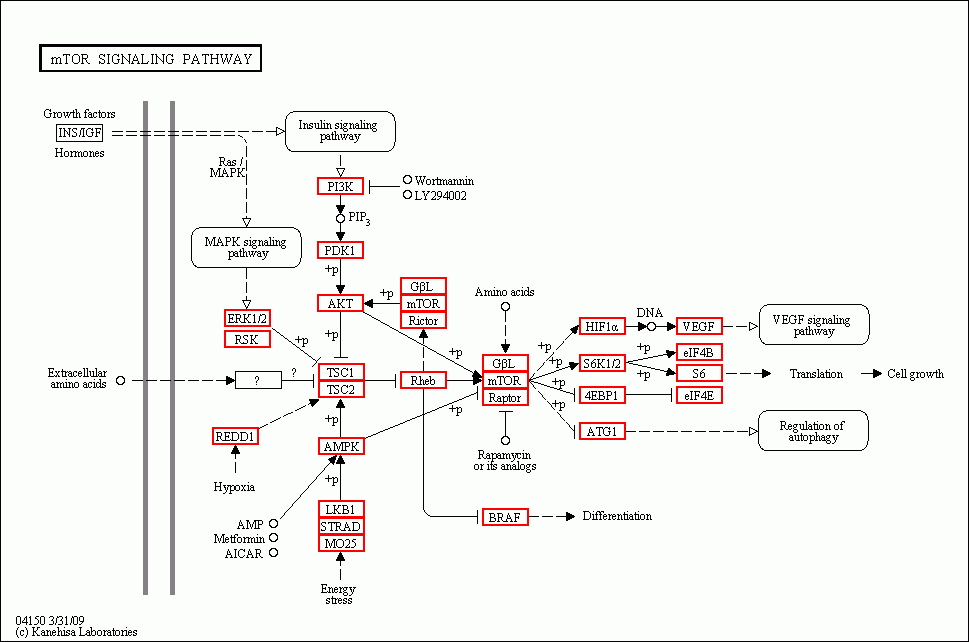

Supplement: Additional file 3: — Pathways found in the annotated portion of the transcriptomes. (ZIP 4950 kb) [file 12864_2015_1817_MOESM3_ESM.zip › map04150.png]

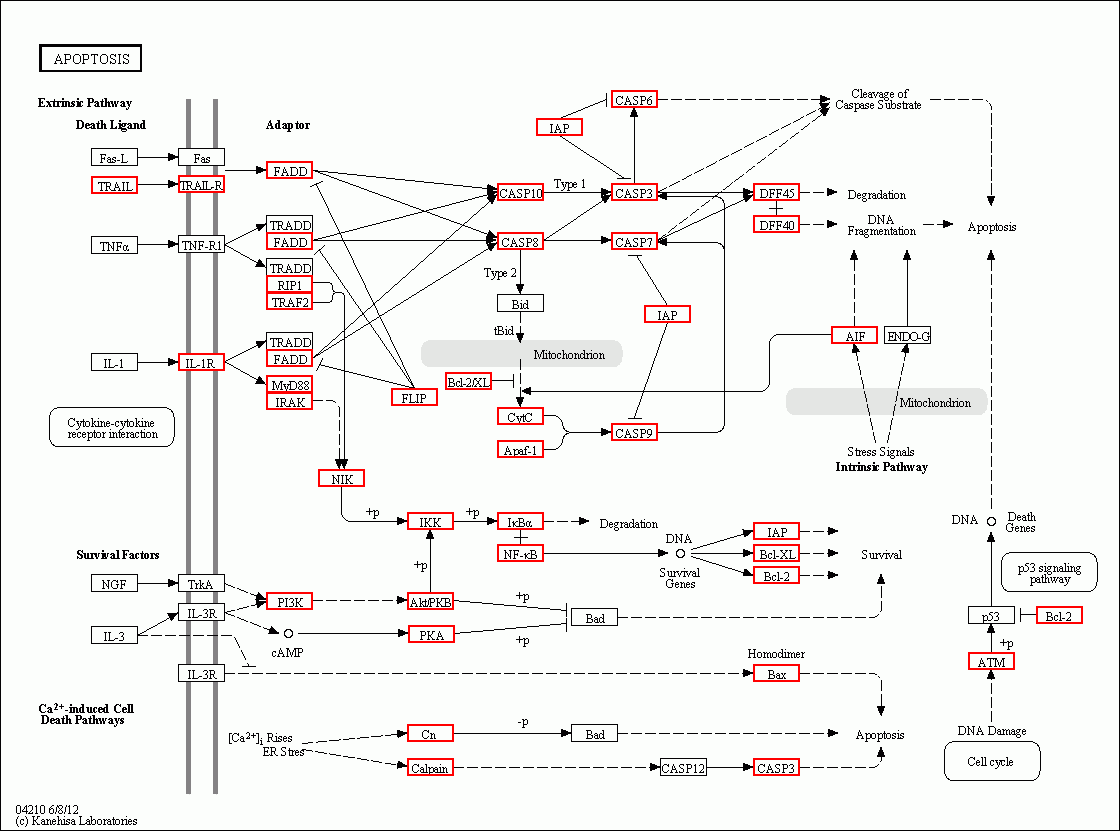

Supplement: Additional file 3: — Pathways found in the annotated portion of the transcriptomes. (ZIP 4950 kb) [file 12864_2015_1817_MOESM3_ESM.zip › map04210.png]

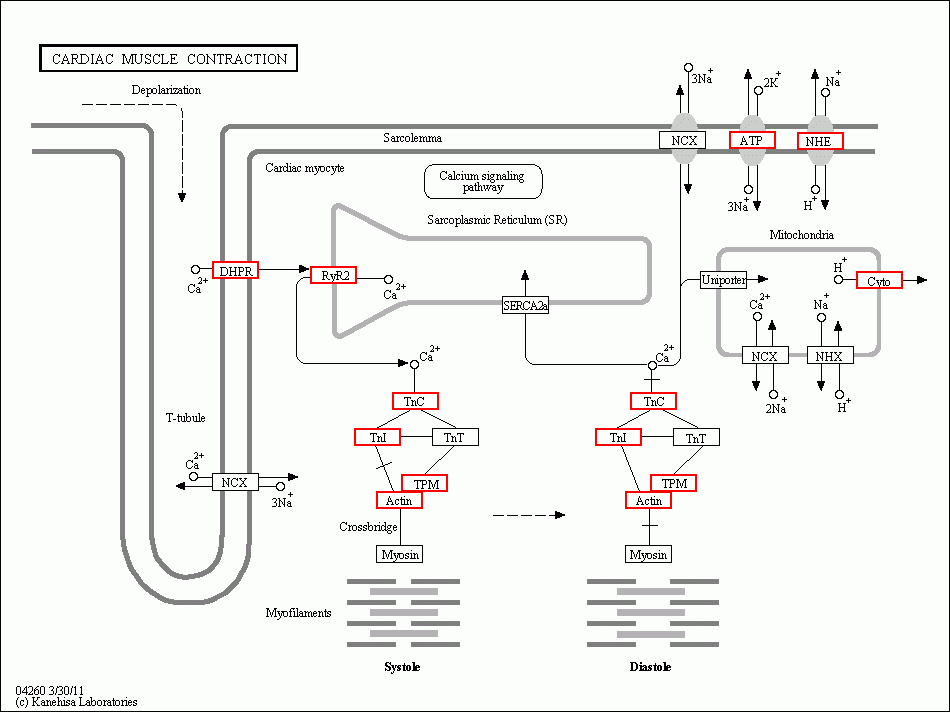

Supplement: Additional file 3: — Pathways found in the annotated portion of the transcriptomes. (ZIP 4950 kb) [file 12864_2015_1817_MOESM3_ESM.zip › map04260.png]

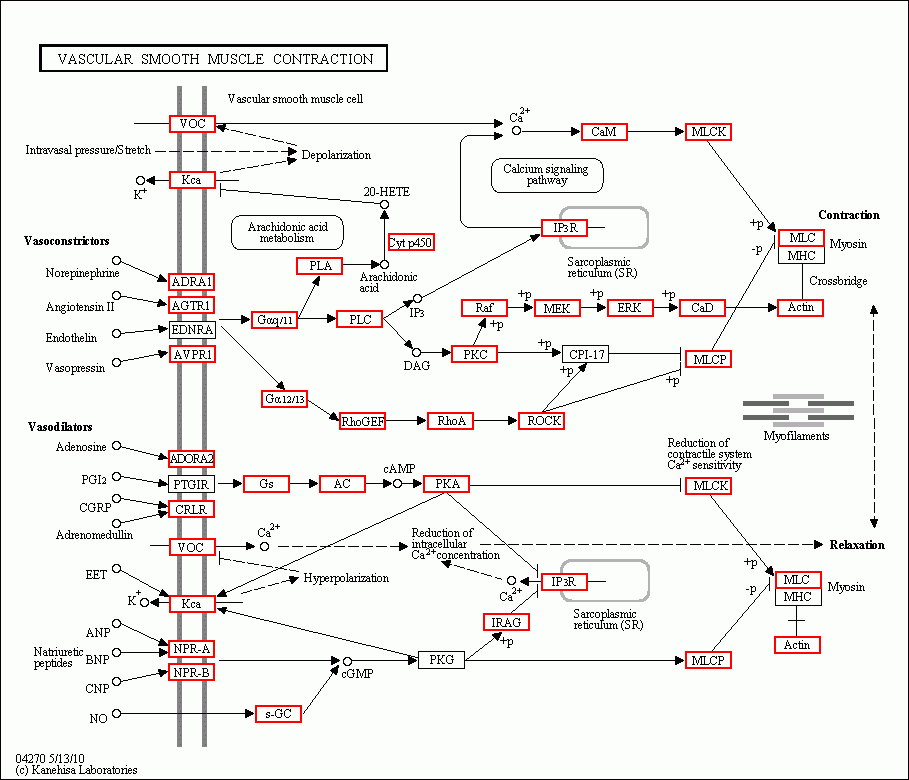

Supplement: Additional file 3: — Pathways found in the annotated portion of the transcriptomes. (ZIP 4950 kb) [file 12864_2015_1817_MOESM3_ESM.zip › map04270.png]

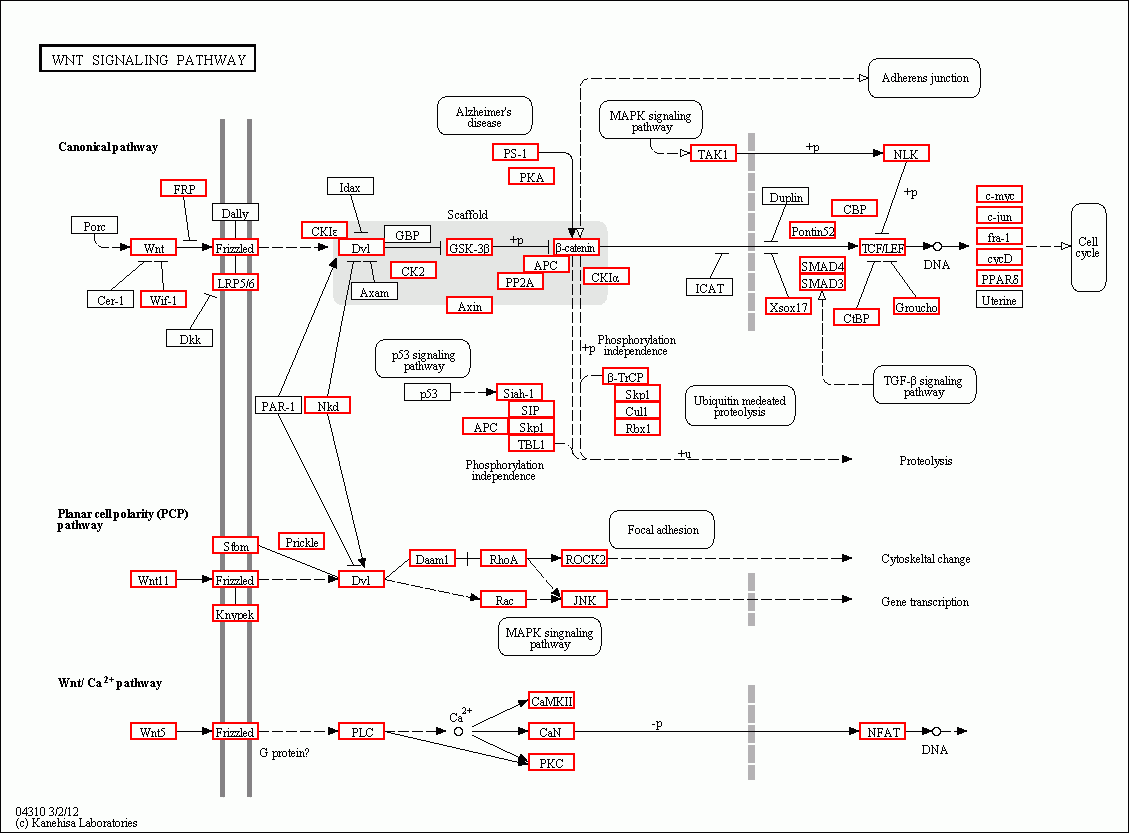

Supplement: Additional file 3: — Pathways found in the annotated portion of the transcriptomes. (ZIP 4950 kb) [file 12864_2015_1817_MOESM3_ESM.zip › map04310.png]

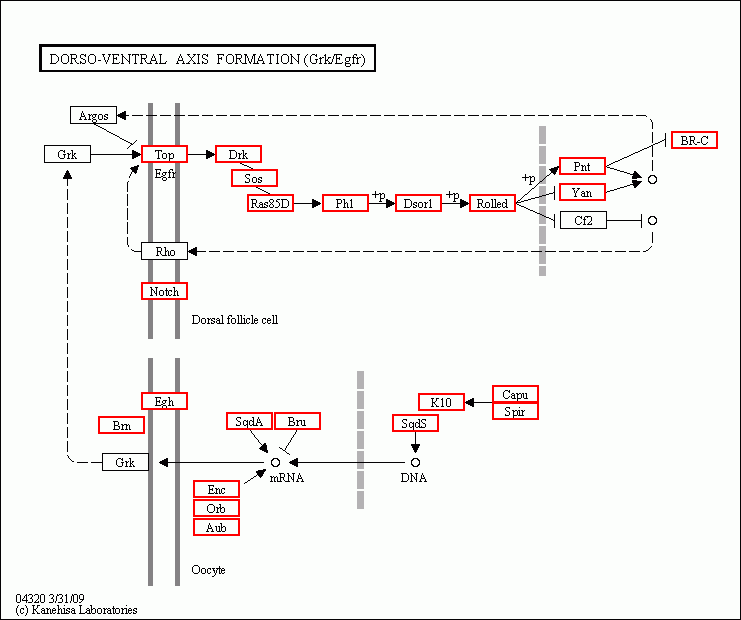

Supplement: Additional file 3: — Pathways found in the annotated portion of the transcriptomes. (ZIP 4950 kb) [file 12864_2015_1817_MOESM3_ESM.zip › map04320.png]

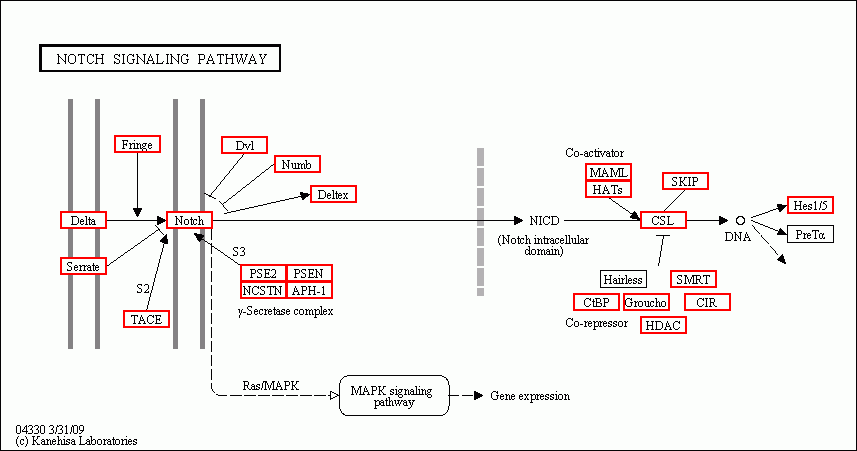

Supplement: Additional file 3: — Pathways found in the annotated portion of the transcriptomes. (ZIP 4950 kb) [file 12864_2015_1817_MOESM3_ESM.zip › map04330.png]

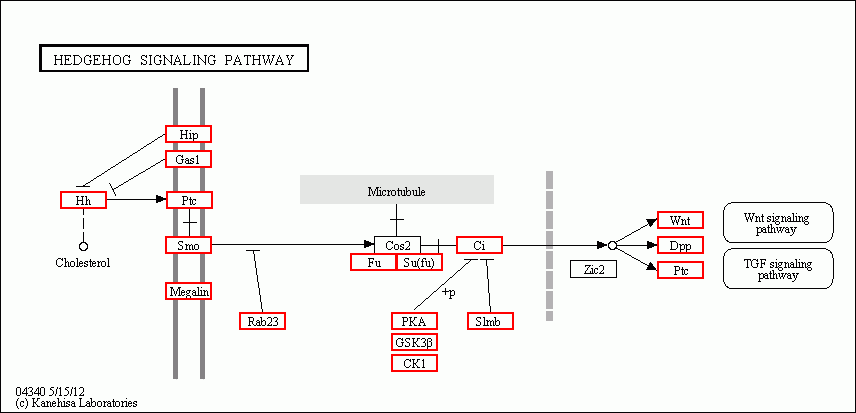

Supplement: Additional file 3: — Pathways found in the annotated portion of the transcriptomes. (ZIP 4950 kb) [file 12864_2015_1817_MOESM3_ESM.zip › map04340.png]

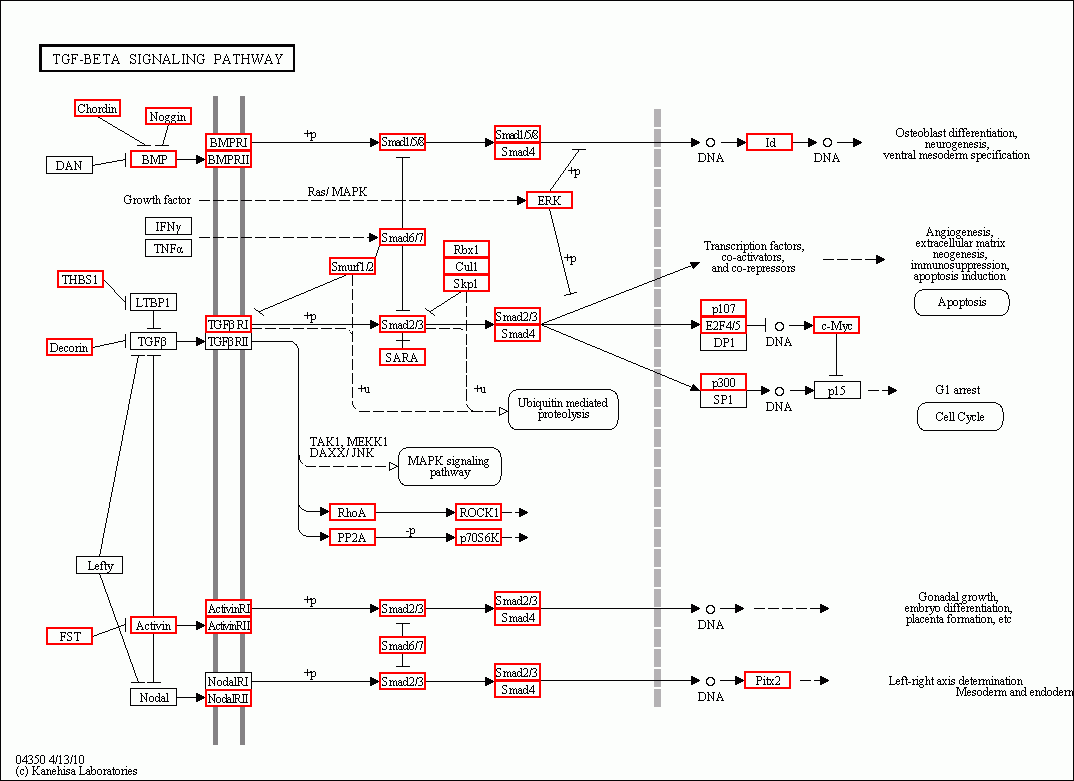

Supplement: Additional file 3: — Pathways found in the annotated portion of the transcriptomes. (ZIP 4950 kb) [file 12864_2015_1817_MOESM3_ESM.zip › map04350.png]

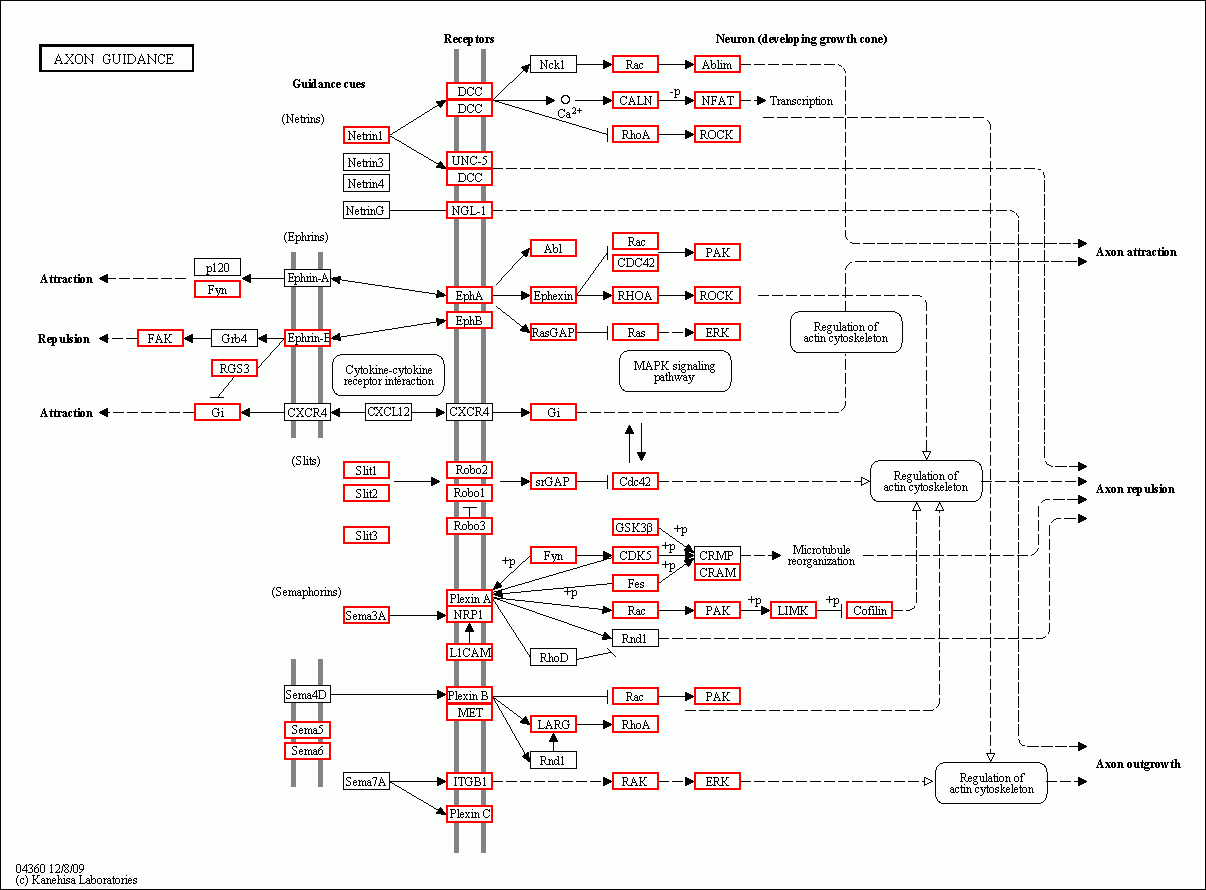

Supplement: Additional file 3: — Pathways found in the annotated portion of the transcriptomes. (ZIP 4950 kb) [file 12864_2015_1817_MOESM3_ESM.zip › map04360.png]

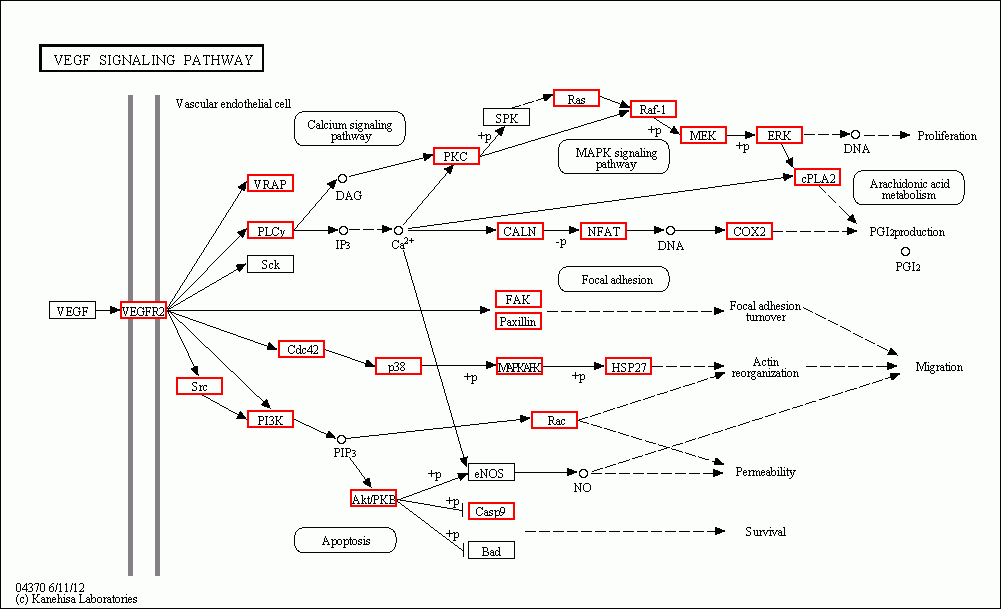

Supplement: Additional file 3: — Pathways found in the annotated portion of the transcriptomes. (ZIP 4950 kb) [file 12864_2015_1817_MOESM3_ESM.zip › map04370.png]

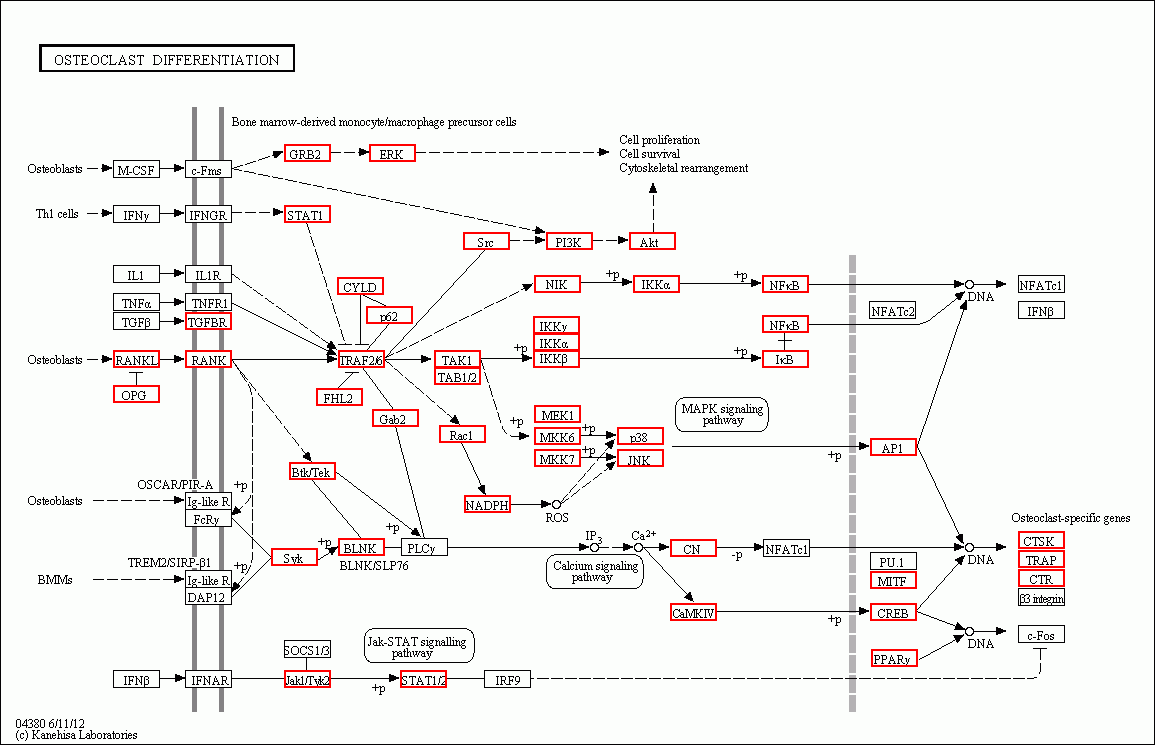

Supplement: Additional file 3: — Pathways found in the annotated portion of the transcriptomes. (ZIP 4950 kb) [file 12864_2015_1817_MOESM3_ESM.zip › map04380.png]

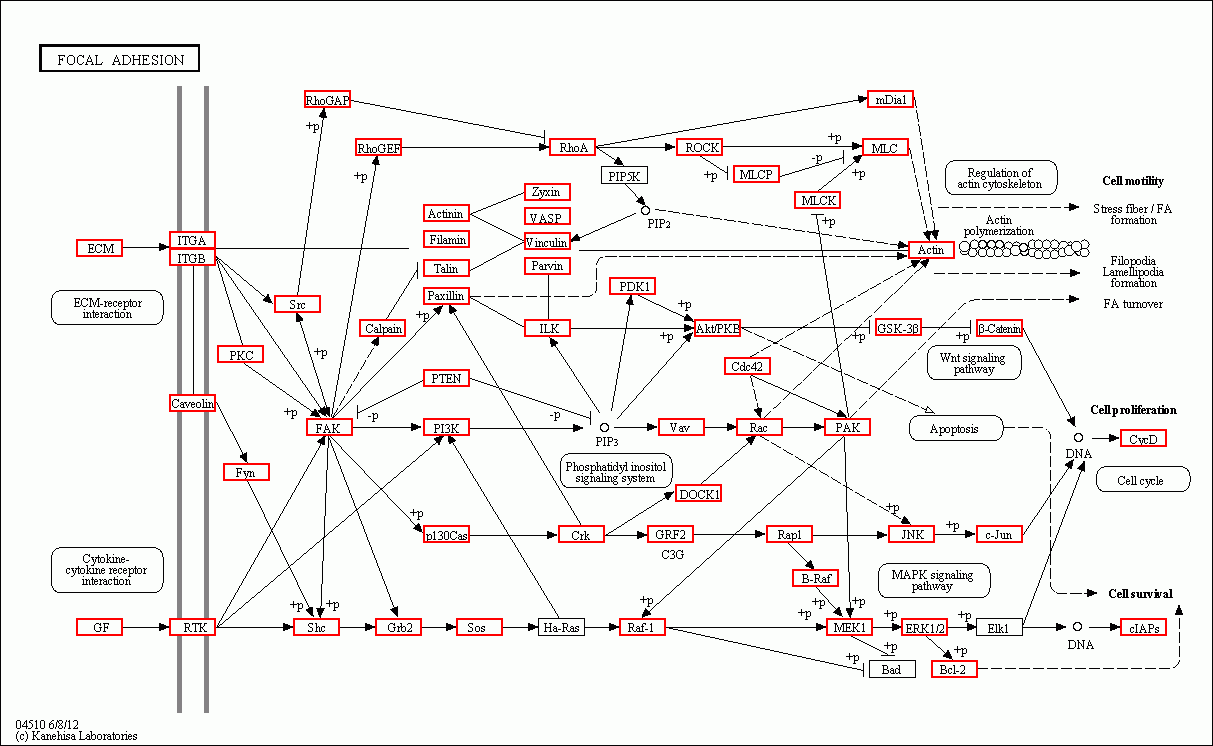

Supplement: Additional file 3: — Pathways found in the annotated portion of the transcriptomes. (ZIP 4950 kb) [file 12864_2015_1817_MOESM3_ESM.zip › map04510.png]

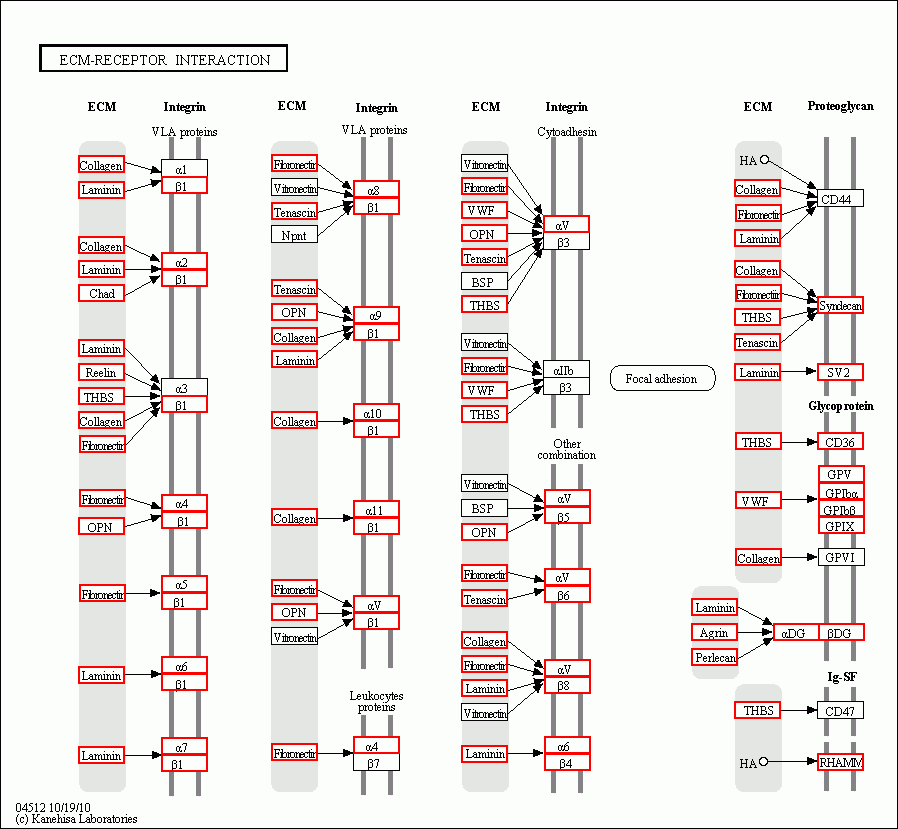

Supplement: Additional file 3: — Pathways found in the annotated portion of the transcriptomes. (ZIP 4950 kb) [file 12864_2015_1817_MOESM3_ESM.zip › map04512.png]

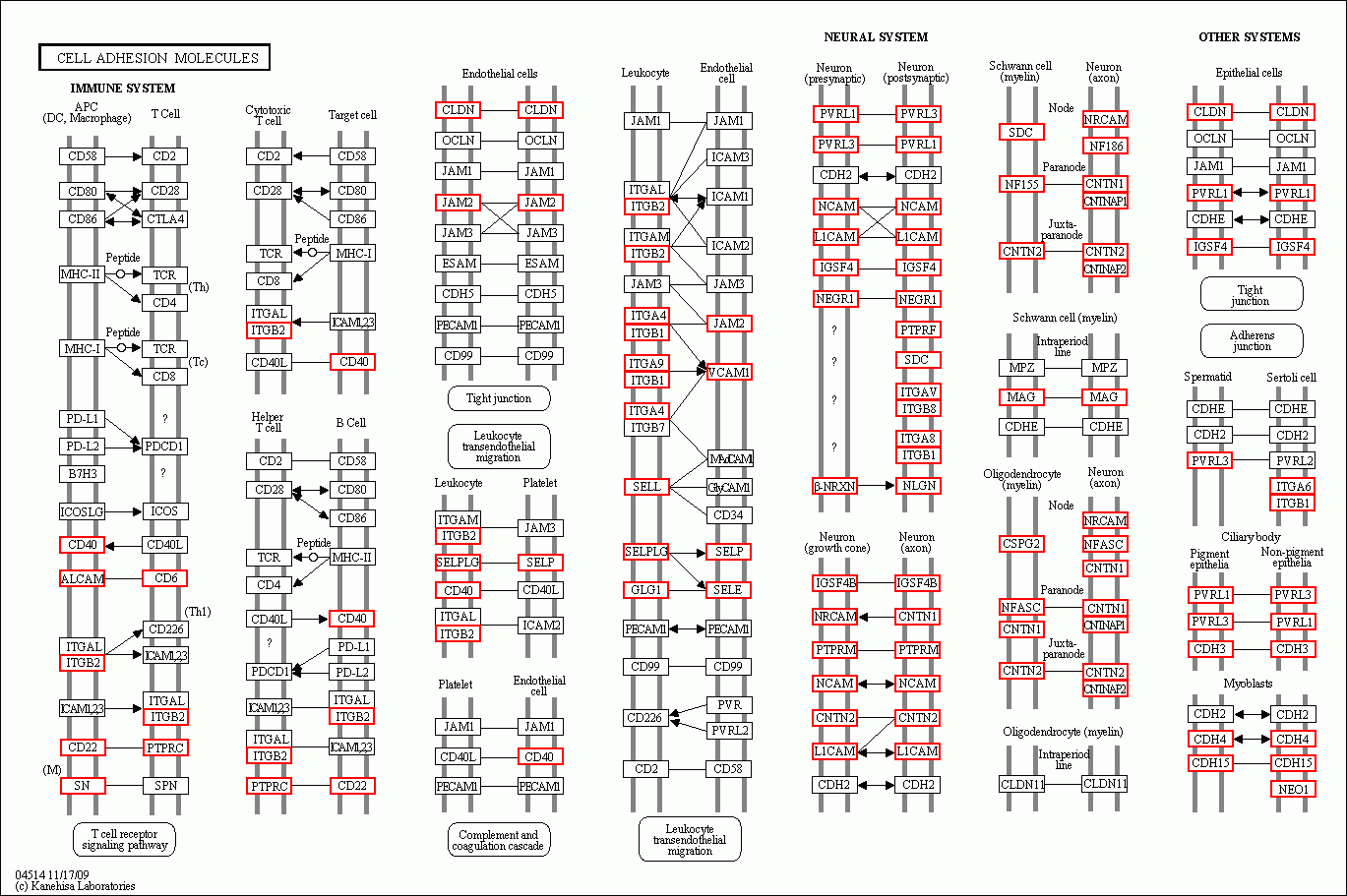

Supplement: Additional file 3: — Pathways found in the annotated portion of the transcriptomes. (ZIP 4950 kb) [file 12864_2015_1817_MOESM3_ESM.zip › map04514.png]

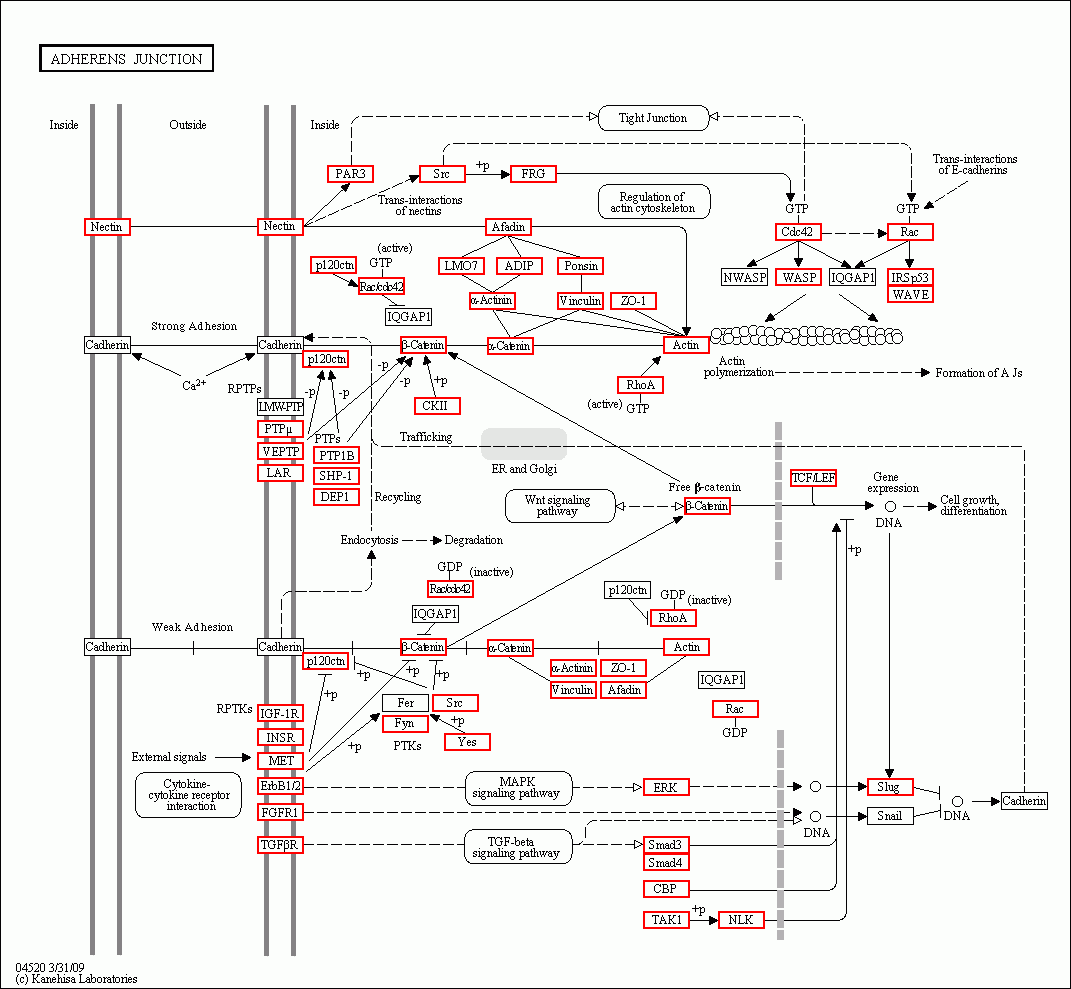

Supplement: Additional file 3: — Pathways found in the annotated portion of the transcriptomes. (ZIP 4950 kb) [file 12864_2015_1817_MOESM3_ESM.zip › map04520.png]

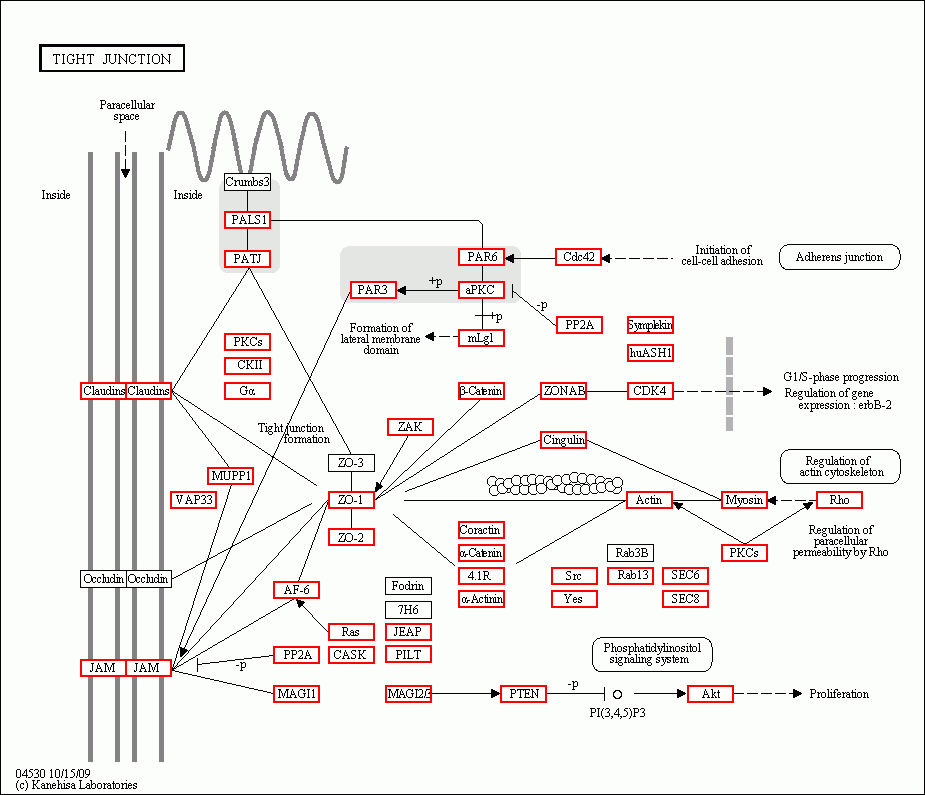

Supplement: Additional file 3: — Pathways found in the annotated portion of the transcriptomes. (ZIP 4950 kb) [file 12864_2015_1817_MOESM3_ESM.zip › map04530.png]

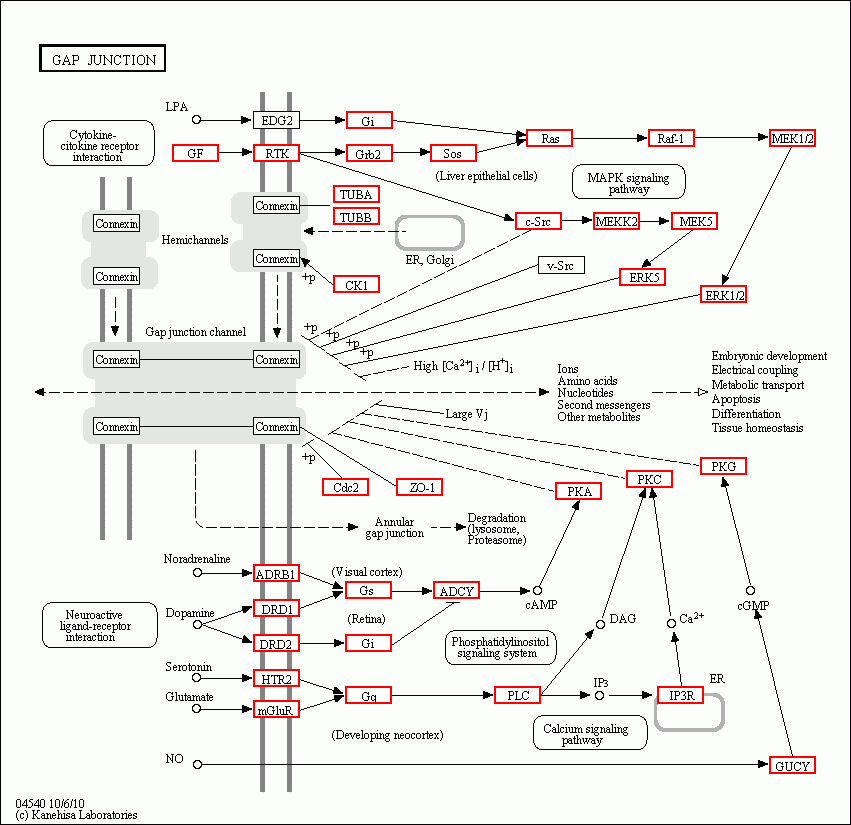

Supplement: Additional file 3: — Pathways found in the annotated portion of the transcriptomes. (ZIP 4950 kb) [file 12864_2015_1817_MOESM3_ESM.zip › map04540.png]

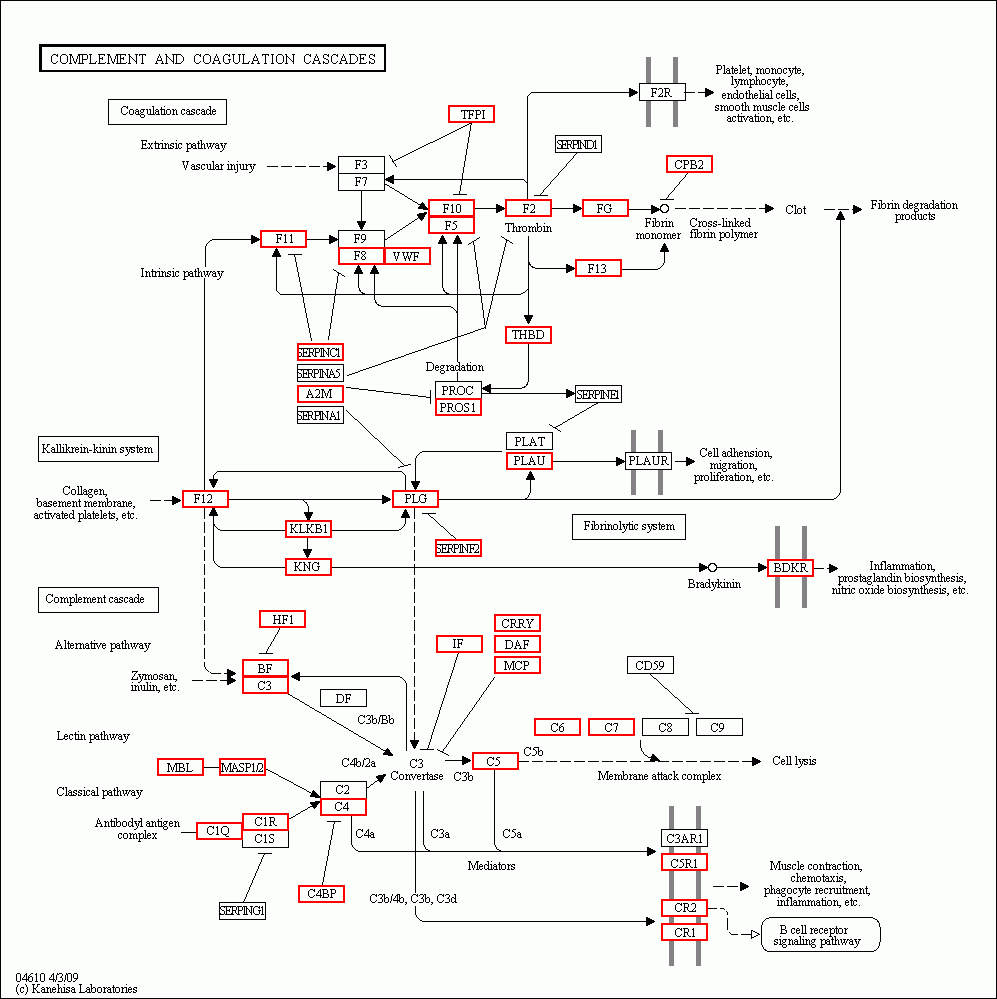

Supplement: Additional file 3: — Pathways found in the annotated portion of the transcriptomes. (ZIP 4950 kb) [file 12864_2015_1817_MOESM3_ESM.zip › map04610.png]

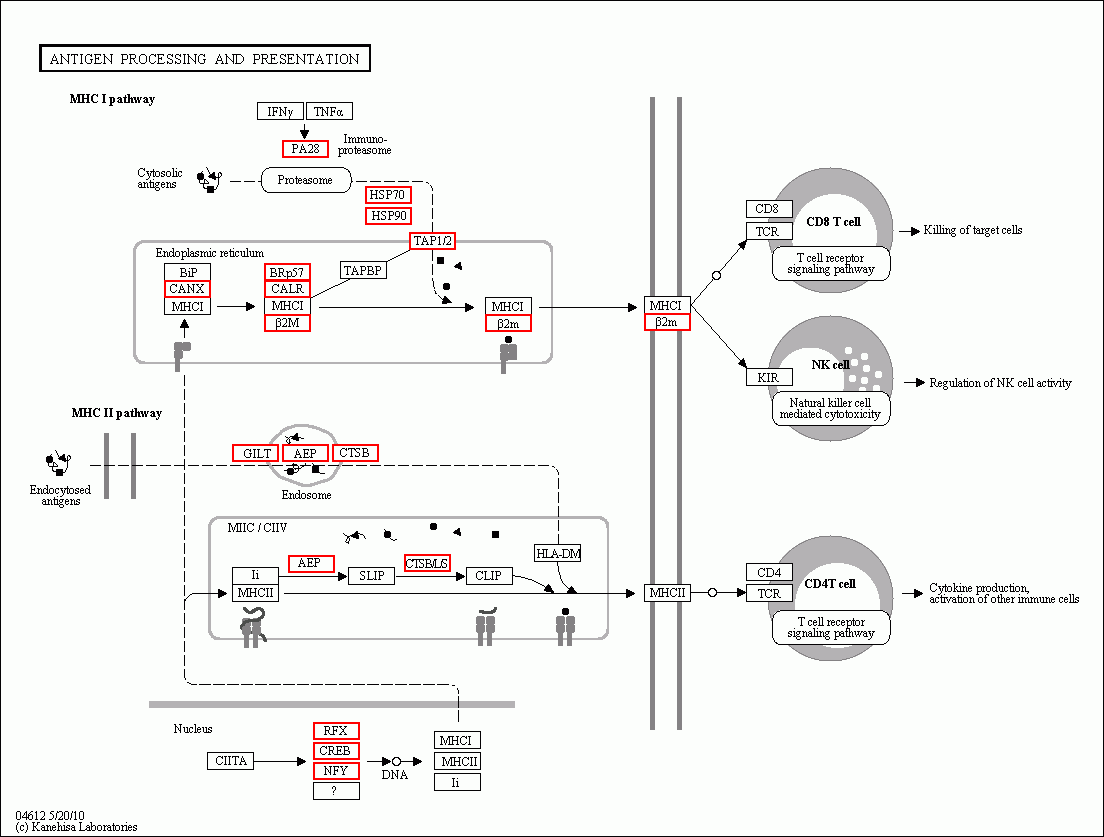

Supplement: Additional file 3: — Pathways found in the annotated portion of the transcriptomes. (ZIP 4950 kb) [file 12864_2015_1817_MOESM3_ESM.zip › map04612.png]

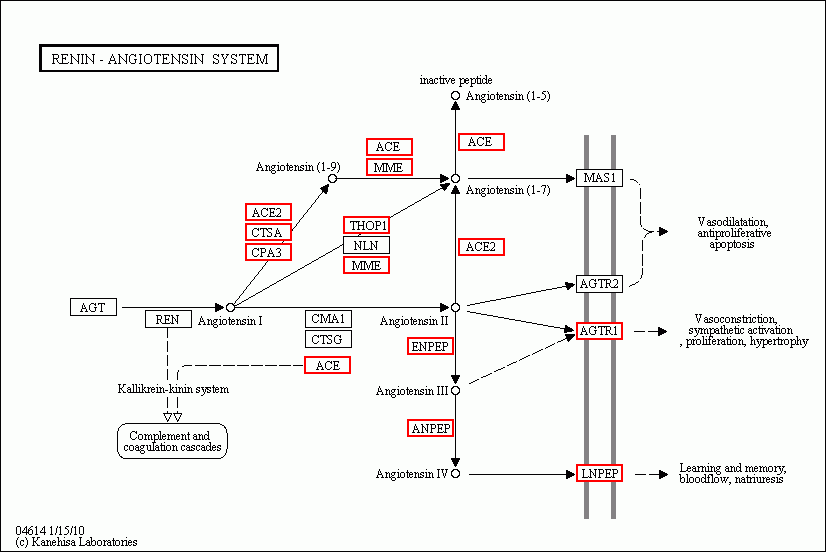

Supplement: Additional file 3: — Pathways found in the annotated portion of the transcriptomes. (ZIP 4950 kb) [file 12864_2015_1817_MOESM3_ESM.zip › map04614.png]

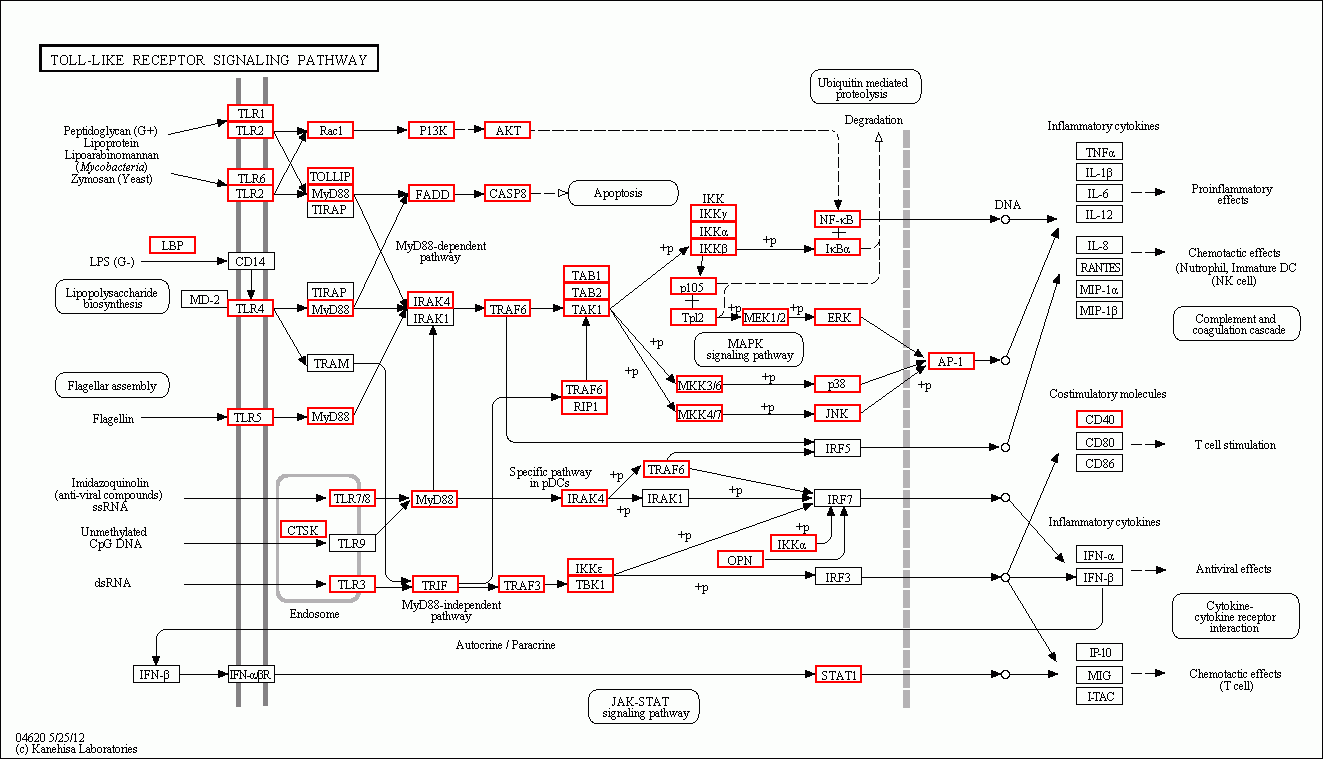

Supplement: Additional file 3: — Pathways found in the annotated portion of the transcriptomes. (ZIP 4950 kb) [file 12864_2015_1817_MOESM3_ESM.zip › map04620.png]

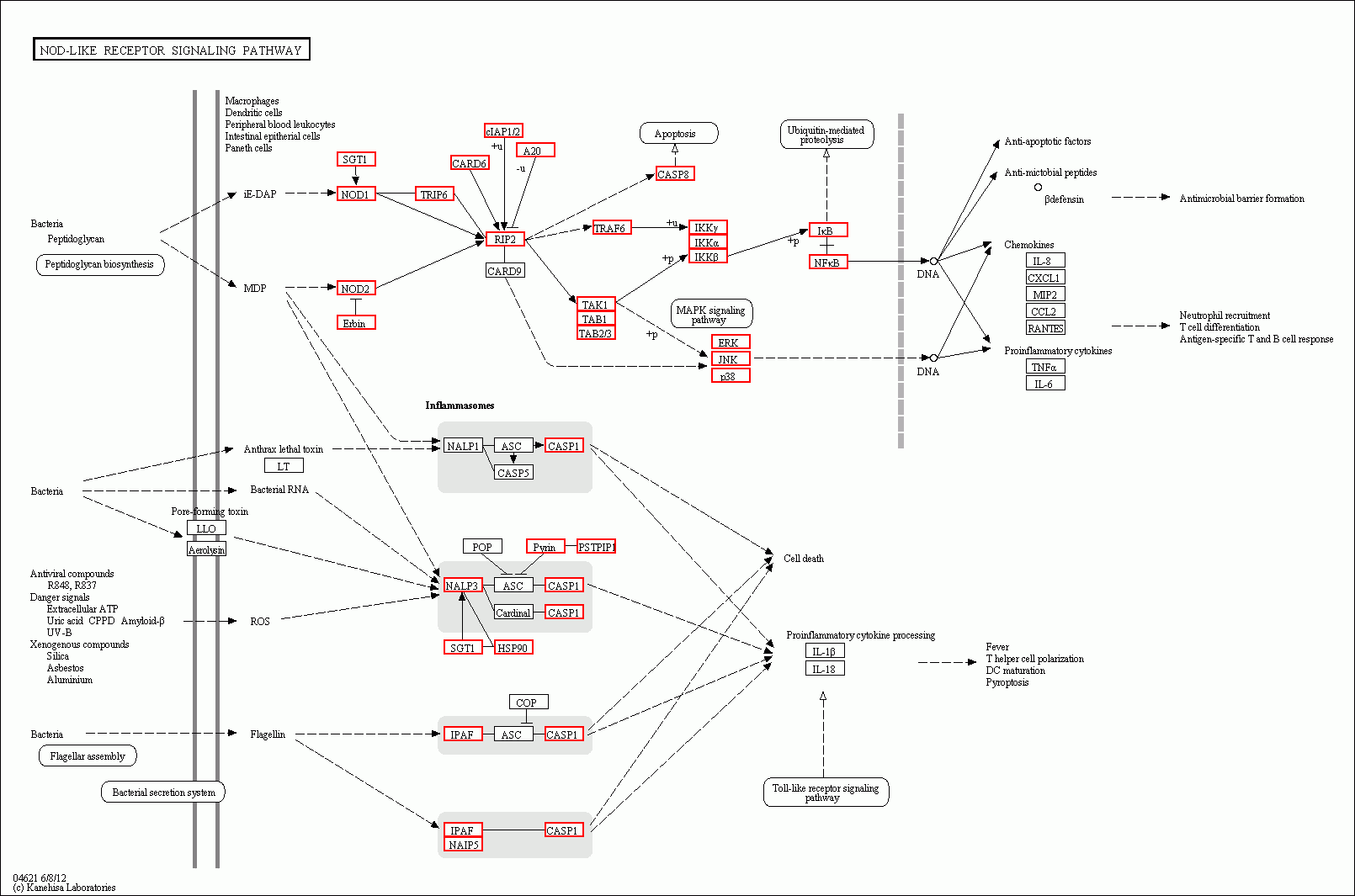

Supplement: Additional file 3: — Pathways found in the annotated portion of the transcriptomes. (ZIP 4950 kb) [file 12864_2015_1817_MOESM3_ESM.zip › map04621.png]

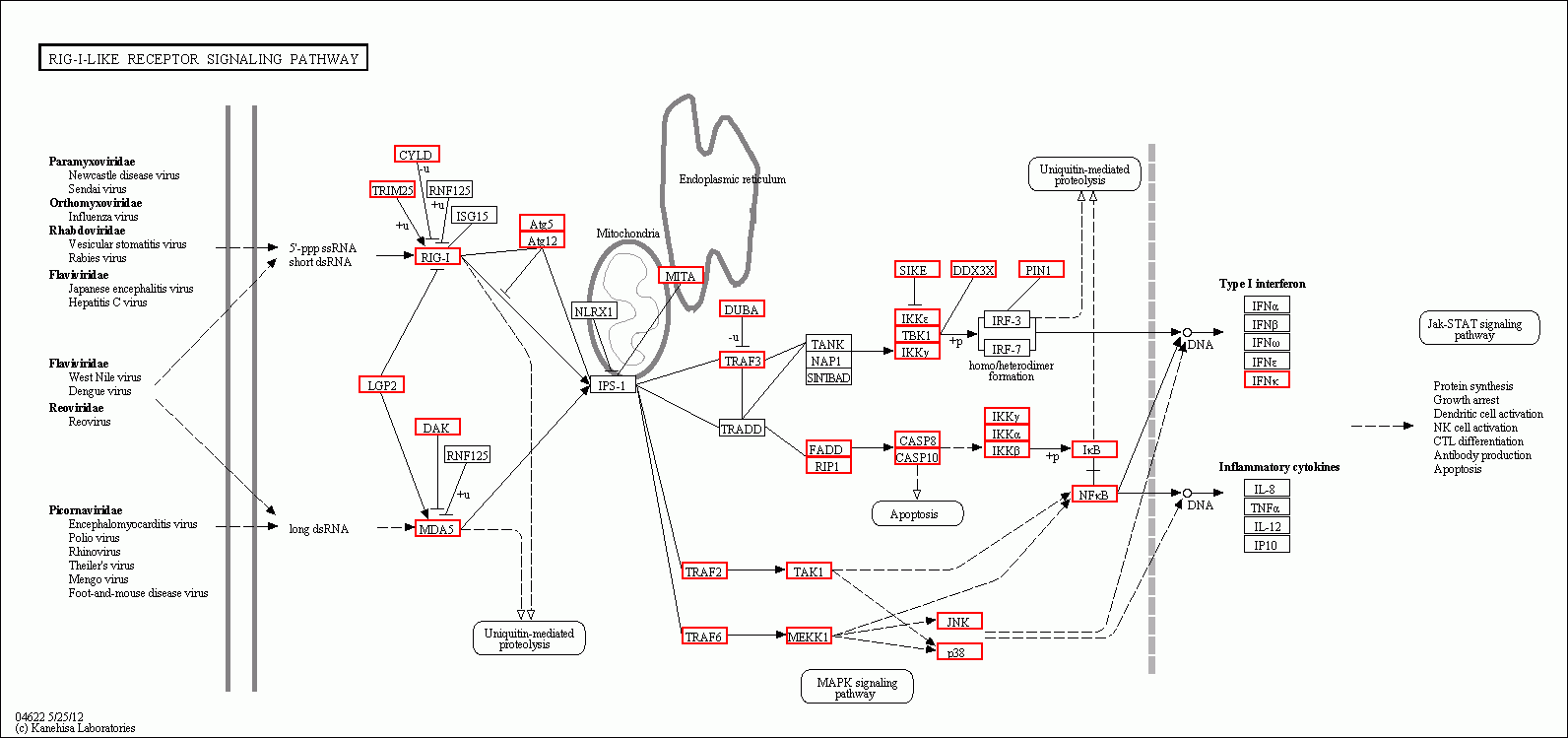

Supplement: Additional file 3: — Pathways found in the annotated portion of the transcriptomes. (ZIP 4950 kb) [file 12864_2015_1817_MOESM3_ESM.zip › map04622.png]

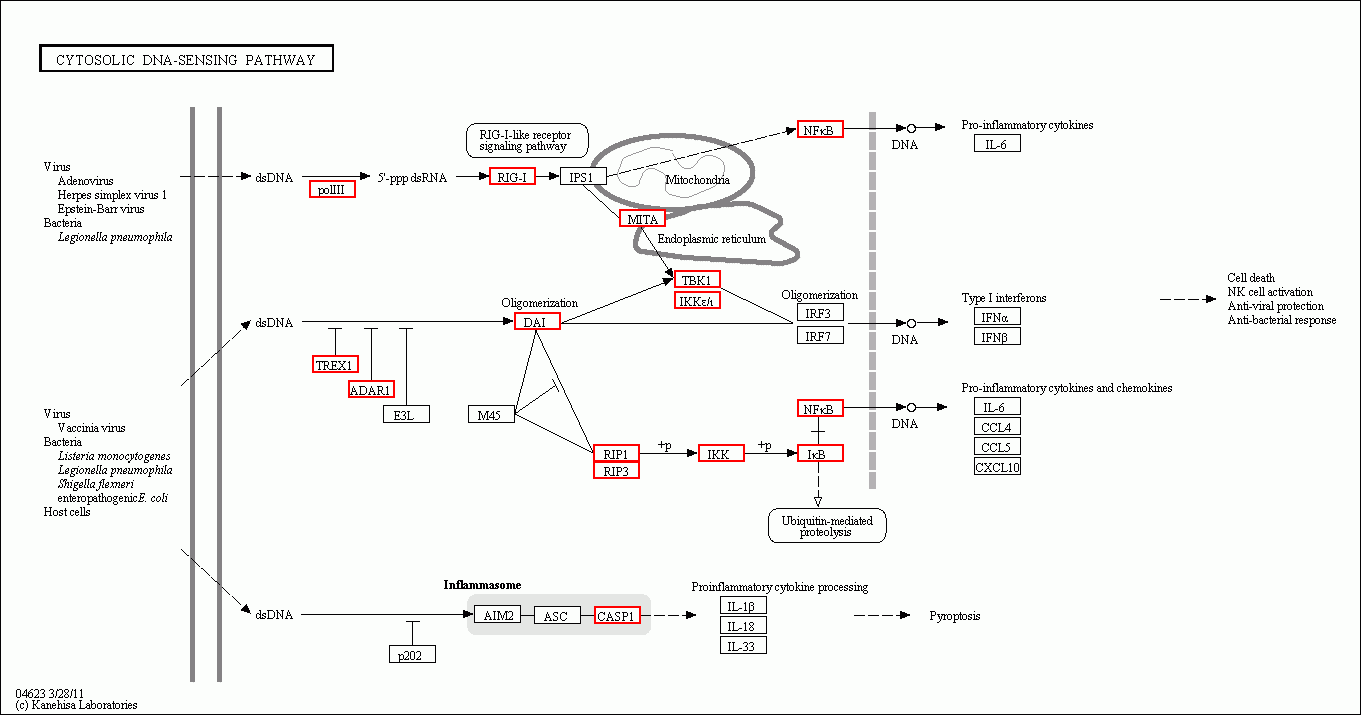

Supplement: Additional file 3: — Pathways found in the annotated portion of the transcriptomes. (ZIP 4950 kb) [file 12864_2015_1817_MOESM3_ESM.zip › map04623.png]

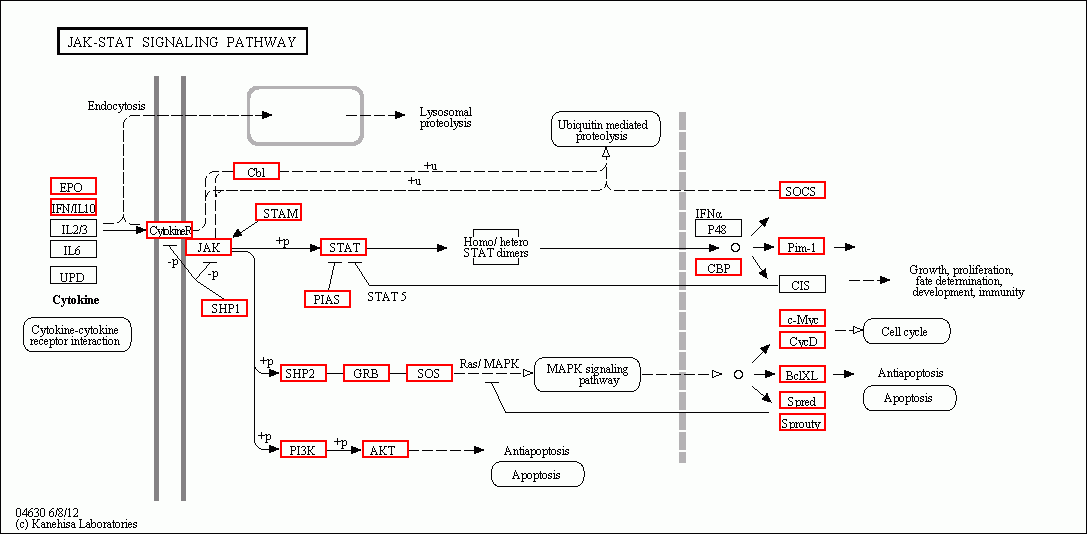

Supplement: Additional file 3: — Pathways found in the annotated portion of the transcriptomes. (ZIP 4950 kb) [file 12864_2015_1817_MOESM3_ESM.zip › map04630.png]

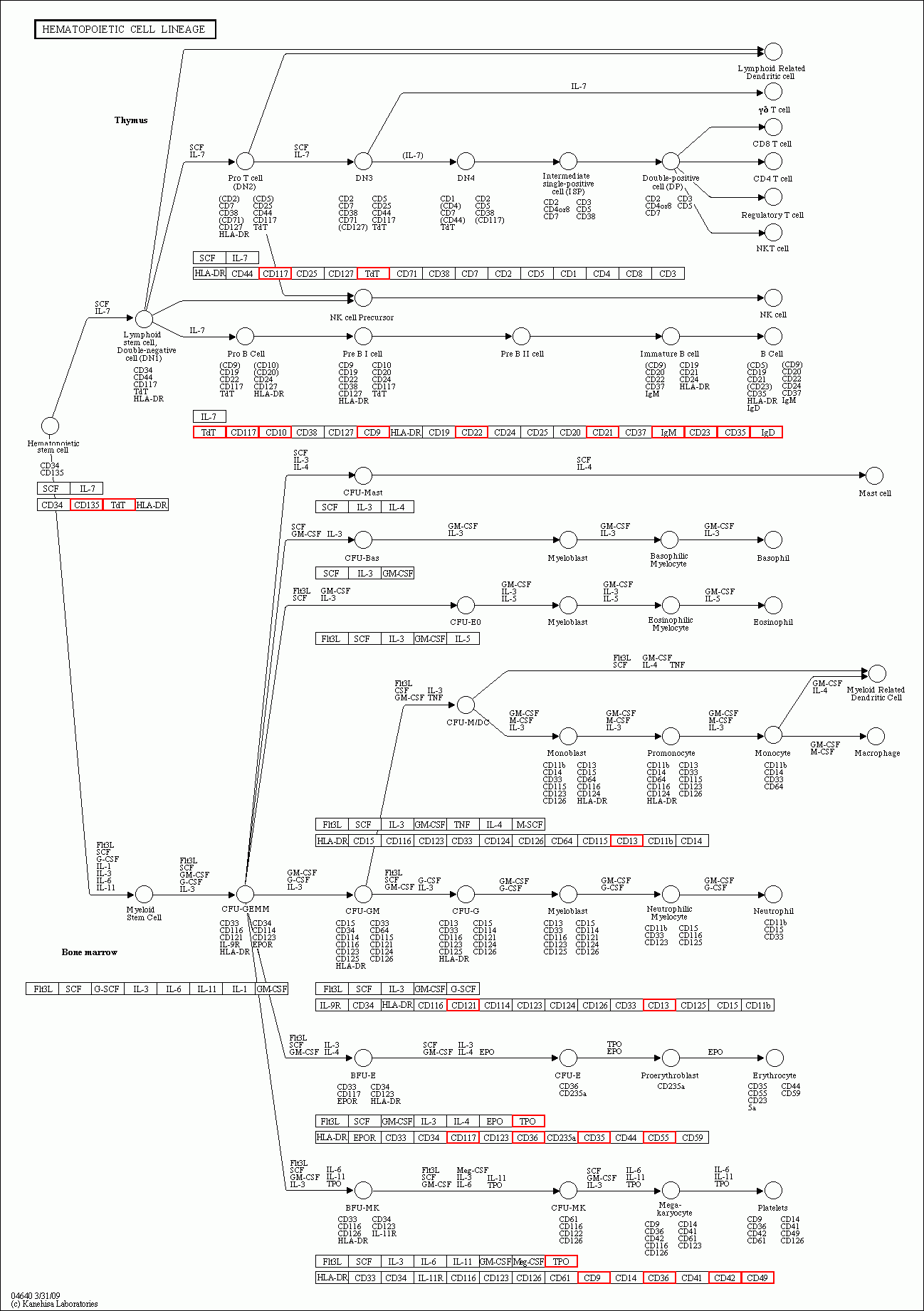

Supplement: Additional file 3: — Pathways found in the annotated portion of the transcriptomes. (ZIP 4950 kb) [file 12864_2015_1817_MOESM3_ESM.zip › map04640.png]

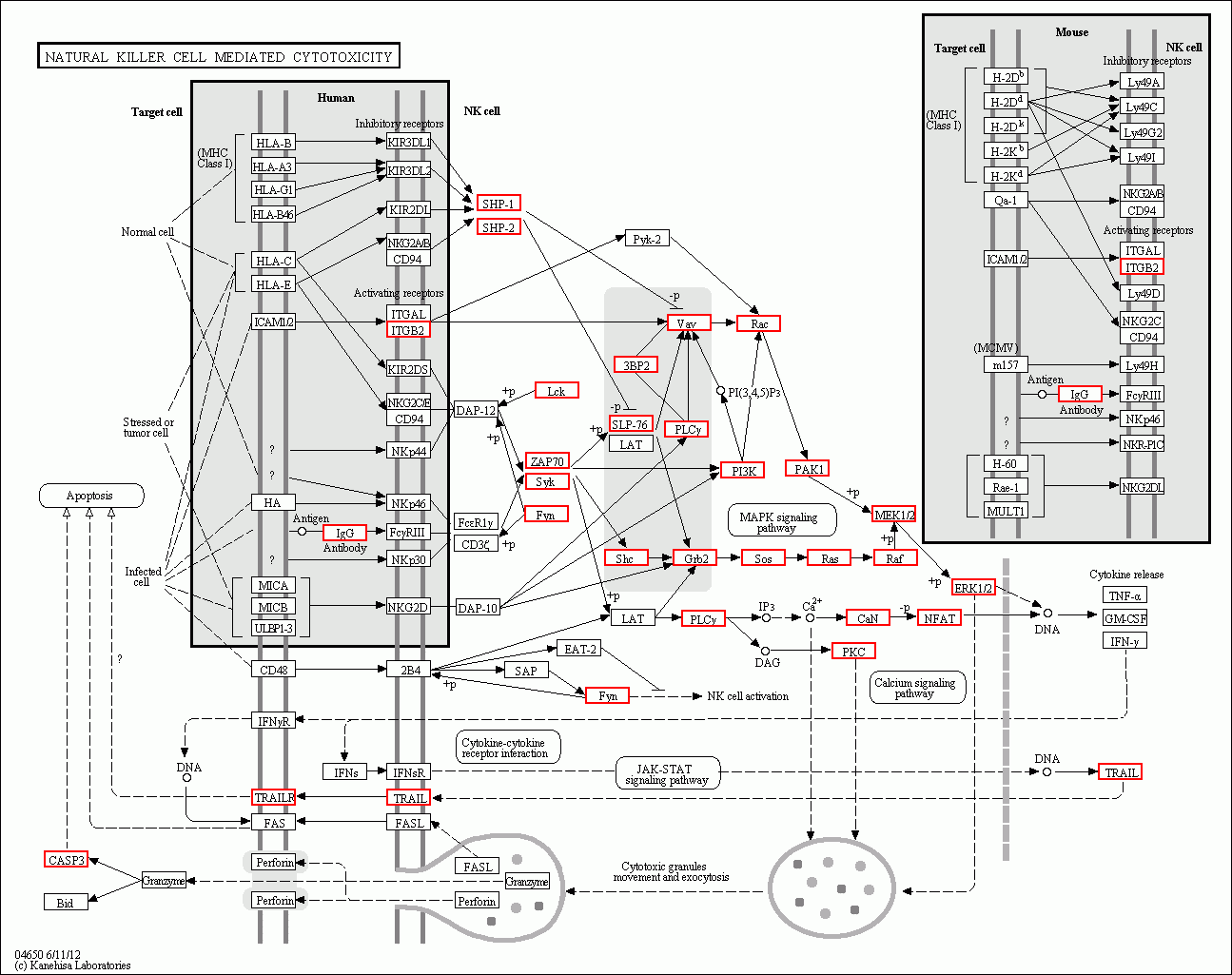

Supplement: Additional file 3: — Pathways found in the annotated portion of the transcriptomes. (ZIP 4950 kb) [file 12864_2015_1817_MOESM3_ESM.zip › map04650.png]
